# Recommendations on screening for primary prevention of fragility fractures

**DOI:** 10.1503/cmaj.221219

**Published:** 2023-05-08

**Authors:** Guylène Thériault, Heather Limburg, Scott Klarenbach, Donna L. Reynolds, John J. Riva, Brett D. Thombs, Laure A. Tessier, Roland Grad, Brenda J. Wilson

**Affiliations:** Departments of Family Medicine (Theriault, Grad) and Psychiatry (Thombs), McGill University, Montréal, Que.; Public Health Agency of Canada (Limburg, Tessier), Ottawa, Ont.; Department of Medicine (Klarenbach), University of Alberta, Edmonton, Alta.; Department of Family and Community Medicine (Reynolds), University of Toronto, Toronto, Ont.; Department of Family Medicine (Riva), McMaster University, Hamilton, Ont.; Department of Medicine (Wilson), Memorial University, St. John’s, NL.

## Abstract

**Background:**

Fragility fractures are a major health concern for older adults and can result in disability, admission to hospital and long-term care, and reduced quality of life. This Canadian Task Force on Preventive Health Care (task force) guideline provides evidence-based recommendations on screening to prevent fragility fractures in community-dwelling individuals aged 40 years and older who are not currently on preventive pharmacotherapy.

**Methods:**

We commissioned systematic reviews on benefits and harms of screening, predictive accuracy of risk assessment tools, patient acceptability and benefits of treatment. We analyzed treatment harms via a rapid overview of reviews. We further examined patient values and preferences via focus groups and engaged stakeholders at key points throughout the project. We used the Grading of Recommendations, Assessment, Development and Evaluation (GRADE) approach to determine the certainty of evidence for each outcome and strength of recommendations, and adhered to Appraisal of Guidelines for Research and Evaluation (AGREE), Guidelines International Network and Guidance for Reporting Involvement of Patients and the Public (GRIPP-2) reporting guidance.

**Recommendations:**

We recommend “risk assessment–first” screening for prevention of fragility fractures in females aged 65 years and older, with initial application of the Canadian clinical Fracture Risk Assessment Tool (FRAX) without bone mineral density (BMD). The FRAX result should be used to facilitate shared decision-making about the possible benefits and harms of preventive pharmacotherapy. After this discussion, if preventive pharmacotherapy is being considered, clinicians should request BMD measurement using dual-energy x-ray absorptiometry (DXA) of the femoral neck, and re-estimate fracture risk by adding the BMD T-score into FRAX (conditional recommendation, low-certainty evidence). We recommend against screening females aged 40–64 years and males aged 40 years and older (strong recommendation, very low-certainty evidence). These recommendations apply to community-dwelling individuals who are not currently on pharmacotherapy to prevent fragility fractures.

**Interpretation:**

Risk assessment–first screening for females aged 65 years and older facilitates shared decision-making and allows patients to consider preventive pharmacotherapy within their individual risk context (before BMD). Recommendations against screening males and younger females emphasize the importance of good clinical practice, where clinicians are alert to changes in health that may indicate the patient has experienced or is at higher risk of fragility fracture.

**Key messages for the public**

- Females aged 65 years and older may be able to reduce their risk of fracture through screening and preventive treatment.
- Screening is not recommended for females younger than 65 years, or for males.
- For further information, please visit www.canadiantaskforce.ca or talk to your primary care provider.

Fragility fractures result from a minor impact that would not damage normal bone.^1^–^3^ These fractures occur because of weakened bone structure (low bone mass or low mineral density, often referred to as osteoporosis).^2^–^4^ The most common fragility fracture sites are the hip, spine, humerus and wrist, also called major osteoporotic fractures (MOFs).^4^–^6^ Individuals with previous fragility fractures are at very high risk of another fragility fracture.^7^,^8^ Some disorders (e.g., diabetes and other endocrine disorders, rheumatoid arthritis, end-stage renal disease) or medications (e.g., chronic glucocorticoids) negatively affect bone density (often referred to as secondary osteoporosis) and increase the risk of fragility fracture.^9^,^10^ Other common risk factors include parental hip fracture, female sex (“female” and “male” refer to biological attributes at birth),^11^ older age, postmenopausal status, lower body weight, previous falls, smoking and alcohol use disorder.^12^–^15^

In 2016, the hip fracture rate in Canada was 168 factures per 100 000 population (aged 65–79 yr) and 1045 per 100 000 (aged ≥ 80 yr).^16^ In 2010/11, fragility fractures cost an estimated $4.6 billion in Canada.^17^ Consequences include disability, chronic pain, admission to hospital and long-term care, and increased mortality. 12,18–20 Quality of life can be substantially reduced, with deficits in mobility and self-care.^12^,^18^,^19^,^21^

Screening involves administration of a test or instrument to all individuals in a particular setting to identify who may benefit from further interventions. Usual care for preventing fragility fractures may vary, but typically includes monitoring of changes in physical health, surveillance of medication and counselling about exercise, fall prevention and nutrition.^22^,^23^

Screening to prevent fragility fractures typically includes risk assessment or measurement of bone mineral density (BMD), or both. Fracture risk assessment tools (e.g., Fracture Risk Assessment Tool [FRAX] with or without BMD testing,^24^ or the Canadian Association of Radiologists/Osteoporosis Canada tool [CAROC], which requires BMD testing^25^) estimate the percentage risk or risk category for hip fracture or MOF over 5 to 10 years.^26^ Measurement of BMD involves dual-energy x-ray absorptiometry (DXA) of the femoral neck (with or without spine or vertebral fracture assessment) and provides a T-score (based on standard reference values) for risk assessment.^12^,^13^,^27^,^28^

“Risk assessment–first” screening includes fracture risk estimation (e.g., FRAX without BMD), followed (if indicated) by BMD.^6^ Risk is then re-estimated by adding the BMD T-score to the calculation. “Bone mineral density test–first” screening starts with BMD, usually followed by risk assessment. For both strategies, preventive pharmacotherapy may be offered to those identified as being at high fracture risk.

First-line pharmacotherapy for fragility fracture prevention includes bisphosphonates (alendronate, risedronate or zoledronic acid) or, rarely, denosumab (e.g., if bisphosphonates are contraindicated). 12,29 Other interventions — such as exercise, smoking cessation, fall prevention strategies and adequate calcium and vitamin D intake — may also reduce risk.^4^,^12^ Screening and preventive pharmacotherapy may, however, lead to unintended consequences (e.g., labelling, stigma, adverse drug effects).^4^,^30^,^31^

This Canadian Task Force on Preventive Health Care (task force) guideline provides evidence-based recommendations on screening, focusing on the primary prevention of fragility fractures through pharmacotherapy. A separate task force guideline on falls prevention is under way.^32^

## Scope

The target population for this guideline is community-dwelling adults aged 40 years and older who are not currently on pharmacotherapy to prevent fragility fractures. Recommendations regarding treatment and nonpharmacologic prevention are not included. This guideline is intended to inform primary care practitioners, defined as health professionals who provide accessible, continued, comprehensive, coordinated care, who are a patient’s first health system contact.^33^

## Recommendations

*We recommend risk assessment–first screening to prevent fragility fractures in females aged 65 years and older, as follows (conditional recommendation, low-certainty evidence):*

- *FRAX: Apply the Canadian clinical FRAX risk assessment tool (without BMD). Use the 10-year absolute risk of MOFs to facilitate shared decision-making about the possible benefits and harms of preventive pharmacotherapy.*
- *BMD + FRAX: After this discussion, if preventive pharmacotherapy is considered, request BMD measurement using DXA of the femoral neck. Then re-estimate fracture risk by adding the BMD T-score into FRAX.*

*We recommend against screening females aged 40–64 years and males aged 40 years and older to prevent fragility fractures (strong recommendation, very low-certainty evidence).*

These recommendations apply to community-dwelling individuals who are not currently on pharmacotherapy to prevent fragility fractures.

The grading of recommendations is described in Box 1, with a summary of the recommendations in Box 2.

**Box 1: Grading of recommendations**

Recommendations are graded according to the Grading of Recommendations Assessment, Development and Evaluation (GRADE) system.^34^,^35^ Whether a recommendation is strong or conditional* will depend on considerations such as certainty in estimated effects of an intervention, including magnitude, as well as estimates of how patients value and prioritize outcomes, variability of these estimates and wise use of resources.

Evidence is graded as high-, moderate-, low- or very low-certainty, based on how likely further research is to change the confidence of the Canadian Task Force on Preventive Health Care (task force) in the estimate of effect.

**Strong recommendations**

- Strong recommendations are those for which the task force is confident that the desirable effects of an intervention outweigh its undesirable effects (strong recommendation for an intervention) or that the undesirable effects of an intervention outweigh its desirable effects (strong recommendation against an intervention). A strong recommendation implies that most people will be best served by the recommended course of action.
- Strong recommendations are typically based on high-certainty evidence (i.e., high confidence in the estimate of the effect of an intervention). Strong recommendations may recommend in favour of an intervention (when there is high confidence of net benefit) or against an intervention (when there is high confidence of net harm). However, there are circumstances in which a strong recommendation may be considered based on low- or very low-certainty evidence, or when there is absence of evidence or low-certainty evidence of benefit.^36^
- When there is an absence of evidence to provide confidence that there is benefit from implementing a new prevention service or when a conclusion of possible benefit requires a high level of speculation on linkages of uncertain evidence, but there is high certainty that some patients would be harmed or scarce health care resources expended, the task force may make a strong recommendation against service implementation.^37^ This is consistent with the GRADE approach, in which strong recommendations are sometimes made with low-certainty evidence combined with high certainty of harm or resource implications, and with the value that the task force places on using scarce primary care resources wisely.^37^

**Conditional recommendations**

- Conditional recommendations are those for which the desirable effects probably outweigh the undesirable effects (conditional recommendation in favour of an intervention) or undesirable effects probably outweigh the desirable effects (conditional recommendation against an intervention) but appreciable uncertainty exists. Conditional recommendations are made when the certainty of evidence is lower, when the margin between desirable and undesirable consequences is small and the balance depends on patient values and preferences, or when there is high variability in the values and preferences of patients. Conditional recommendations may also be applied when the balance of cost and benefits is ambiguous, key stakeholders differ about the acceptability or feasibility of the implementation, or the effects on health equity are unclear.
- In certain cases where a conditional recommendation for an intervention is made, clinicians are encouraged to engage in shared decision-making, to recognize that different choices will be appropriate for individual patients, and to help each person arrive at a management decision consistent with their values and preferences. Clinicians should recognize that different choices will be appropriate for different patients and that decisions must be consistent with each patient’s values and preferences. Knowledge translation tools are available on the task force website (www.canadiantaskforce.ca) to facilitate decisions that are evidence informed and aligned with an individual’s priorities.

*The task force previously used the term “weak recommendation,” but has replaced this with the term “conditional recommendation,” to improve understanding and facilitate implementation of guidance, based on feedback from clinician knowledge users. One reason for this change was the value that the task force places on shared decision-making, together with a need to better clarify when implementation of a recommendation depends on circumstances such as patient values, resource availability or other contextual considerations. Conditional recommendations based on patient values and preferences require clinicians to recognize that different choices will be appropriate for different patients and those decisions must be consistent with each patient’s values and preferences.

**Box 2: Summary of recommendations for clinicians and policy-makers**

*The Canadian Task Force on Preventive Health Care (task force) recommends “risk assessment–first” screening to prevent fragility fractures in females aged ≥ 65 years, as follows (conditional recommendation; low-certainty evidence):*

- *Apply the Canadian clinical FRAX* * *(without BMD). Use the 10-year absolute risk of major osteoporotic fracture to facilitate shared decision-making about the possible benefits and harms of preventive pharmacotherapy.*
- *After discussion, if preventive pharmacotherapy is considered, request BMD measurement using DXA of the femoral neck. Then re-estimate fracture risk by adding the BMD T-score into FRAX.*

*We recommend against screening females aged 40–64 years and males aged ≥ 40 years to prevent fragility fractures (strong recommendation; very low-certainty evidence).*

These recommendations apply to community-dwelling individuals who are not currently on pharmacotherapy to prevent fragility fractures. In the opinion of the task force, the benefit (reduction in hip fractures and clinical fragility fractures) of screening for females aged ≥ 65 years outweighs the risk of overdiagnosis (of high risk) and adverse events from potential medication.

There was no evidence for screening to prevent fragility fractures in males aged 40–64 years and females aged 55–64 years. For males aged ≥ 65 years and women aged 45–54 years, there was no evidence establishing a benefit (evidence was very uncertain). Additionally, screening to prevent fragility fractures in these populations is not standard practice in Canada. This resulted in a strong recommendation against screening when considering the harms and substantial resource use.

Although some observational studies suggest screening intervals based on age, baseline BMD or absolute fracture risk, repeating BMD at 3–8 years did not improve fracture risk prediction.^13^,^38^–^41^ It is unknown how often to rescreen eligible females, but rescreening within 8 years does not appear useful.

Note: BMD = bone mineral density, DXA = dual-energy x-ray absorptiometry, FRAX = Fracture Risk Assessment Tool.*The Canadian clinical FRAX risk assessment tool^21^ is available at https://frax.shef.ac.uk/FRAX/tool.aspx?country=19 and the task force Fragility Fracture Decision Aid is available at https://frax.canadiantaskforce.ca/.

We conducted 4 systematic reviews and 1 rapid overview of reviews for this guideline (with meta-analyses where appropriate). 42,43 The first systematic review^42^ (on harms and benefits of screening) found 4 randomized controlled trials (RCTs) (3 in females aged ≥ 65 yr and 1 in younger females),^5^,^6^,^44^,^45^ 1 clinical controlled trial (i.e., quasi-randomized)^46^ evaluating screening interventions to prevent fragility fractures, and 1 RCT comparing BMD testing–first versus risk assessment–first screening.^47^ In 2 RCTs,^5^,^44^ participants were self-selected based on willingness to complete a risk assessment independently (a subgroup that may differ from the general population). Another RCT^45^ and the clinical controlled trial^46^ included all eligible individuals (based on sex and age criteria). A final RCT^6^ included analysis for both “self-selected” and “all eligible” population analyses. All studies recruited via mailed invitations, which differs from the typically opportunistic screening setting in Canada.^48^

The second systematic review examined risk prediction tool calibration with 32 validation cohort studies.^42^ The third systematic review included 27 RCTs on treatment benefits.^42^ The fourth systematic review found 1 study examining values and preferences of screening^49^ and 11 studies on acceptability of initiating treatment.^42^ A rapid overview of reviews on treatment harms included 10 systematic reviews.^42^

### Benefits of screening

#### Trials of screening interventions

A meta-analysis^42^ of 3 RCTs and 1 clinical controlled trial (*n* = 43 736, 3–5-yr follow-up)^5^,^6^,^44^,^46^ found that among “self-selected” females aged 65 years and older, risk assessment–first screening (as described above) probably reduces hip fractures with 6.2 fewer per 1000 screened (95% confidence interval [CI] 2.8 to 9.0 fewer; number needed to screen [NNS] = 161) (moderate-certainty evidence). Screening also probably reduces all clinical fragility fractures (including MOFs) with 5.9 fewer per 1000 (95% CI 0.8 to 10.9 fewer; NNS = 169) (moderate-certainty evidence). These were re-estimated using Canadian fracture rates (10-yr follow-up from 1995–2005),^50^ resulting in 4.0 fewer (95% CI 1.8 to 5.8 fewer) hip fractures and 11.8 fewer (95% CI 1.7 to 21.8 fewer) clinical fragility fractures per 1000 screened, respectively (Appendix 1, available at www.cmaj.ca/lookup/doi/10.1503/cmaj.221219/tab-related-content).^42^

There was little to no difference in all-cause mortality among “self-selected” females aged 65 years and older (0.0 fewer per 1000, 95% CI 7.1 fewer to 5.3 more) (moderate-certainty evidence) 5,44,46 and all eligible females aged 65 years and older (3.5 fewer per 1000, 95% CI 9.4 fewer to 3.5 more) (low-certainty evidence).^6^ Screening may make little to no difference to health-related quality of life (5-yr follow-up) for “self-selected” females aged 65 years and older.^44^ There was no evidence for fracture-related mortality or functionality and disability.

Among “all eligible” (i.e., all participants regardless of FRAX or questionnaire completion) females aged 65 years and older,^6^ screening may not reduce hip (0.3 fewer per 1000, 95% CI 4.2 fewer to 3.9 more) or clinical fragility fractures (1.0 fewer per 1000, 95% CI 8.0 fewer to 6.0 more) (low-certainty evidence). One study^47^ comparing BMD testing–first versus risk assessment–first screening provided very low-certainty evidence and therefore did not confirm one strategy over the other.

In males aged 65 years and older,^46^ evidence was very uncertain for hip fractures and therefore did not establish a benefit. Evidence for females aged 45–54 years^45^ was very uncertain for hip and clinical fragility fractures and therefore did not establish a benefit. No evidence was found for females aged 55–64 years, males aged 40–64 years or on screening intervals.

#### Efficacy of treatment

Bisphosphonates as a drug class may reduce hip and clinical vertebral fractures and probably reduce all clinical fragility fractures in postmenopausal females (low-to moderate-certainty evidence).^42^ Denosumab probably reduces clinical vertebral and all clinical fragility fractures in postmenopausal females (moderate-certainty evidence) but may not reduce hip fractures (low-certainty evidence).^42^ Evidence for males showed that zoledronic acid may not reduce hip or clinical fragility fractures, and was very uncertain for denosumab (Appendix 2, available at www.cmaj.ca/lookup/doi/10.1503/cmaj.221219/tab-related-content).

### Harms of screening

#### Trials of screening interventions

In screening to prevent fragility fractures, overdiagnosis occurs when individuals are correctly classified or labelled as being at high risk of fracture but would never have known this nor experienced a fracture and may therefore undergo further assessments or preventive pharmacotherapy without benefit.^42^ Two trials included data used to calculate overdiagnosis of high risk (Appendix 3, available at www.cmaj.ca/lookup/doi/10.1503/cmaj.221219/tab-related-content).^5^,^44^ Among “self-selected” females (aged ≥ 65 yr) who were screened, 11.8% (using 10-yr hip fracture risk)^44^ and 19.3% (using 10-yr MOF risk)^5^ would be over-diagnosed as high risk (low-certainty evidence).^42^

#### Adverse effects of treatment

Analysis of treatment harms (v. placebo) found that alendronate and denosumab probably increase nonserious gastrointestinal adverse events (e.g., nausea, diarrhea, reflux) (moderate-certainty evidence) (Appendix 4, available at www.cmaj.ca/lookup/doi/10.1503/cmaj.221219/tab-related-content).^42^ Denosumab probably increases rash and eczema (moderate-certainty evidence) and infections (moderate-certainty evidence).^42^ Zoledronic acid probably increases several nonserious adverse events (e.g., headache, influenza-like symptoms, arthritis and arthralgia) (moderate-certainty evidence).^42^ Alendronate and bisphosphonates (as a drug class) may increase rare (i.e., < 5 events per 10 000) but serious harms of atypical femoral fracture and osteonecrosis of the jaw (low-certainty evidence).^42^ No other serious or nonserious adverse events were associated with treatment.

### Accuracy of risk assessment tools

After preplanned subgroup analyses (sex, age, risk of bias, baseline risk) to investigate high heterogeneity across FRAX studies, we used calibration estimates (i.e., observed to expected fracture ratio) from studies not at high risk of bias (Appendix 5, available at www.cmaj.ca/lookup/doi/10.1503/cmaj.221219/tab-related-content).^42^ In addition to having consistent findings, these studies used Canadian FRAX (considered highly applicable).

Clinical FRAX (without BMD) may be well calibrated (threshold 0.8–1.2) to predict 10-year hip fractures (1.13, 95% CI 0.74–1.72) (low-certainty evidence) and is probably well calibrated for clinical fragility fractures (1.10, 95% CI 1.01–1.20) (moderate-certainty evidence). FRAX with BMD is probably well calibrated to predict 10-year clinical fragility fractures (1.16, 95% CI 1.12–1.20) (moderate-certainty evidence) but may be poorly calibrated (1.31, 95% CI 0.91–2.13) to predict 10-year hip fractures (low-certainty evidence).

Although CAROC may be well calibrated to predict categories (low, medium, high) of clinical fragility fracture risk (low-certainty evidence), it does not permit risk assessment–first screening.

Results from other tools (i.e., Garvan Fracture Risk Calculator, 51 QFracture,^52^ Fracture and Immobilization Score [FRISC]^53^ and Fracture Risk Calculator [FRC]^54^) were very uncertain.^42^

### Patient values and preferences

We conducted surveys and focus groups to rate outcomes and determine acceptability among patients for this guideline (Appendix 6, available at www.cmaj.ca/lookup/doi/10.1503/cmaj.221219/tab-related-content). We asked participants to rate their willingness to screen on a scale of 1–9, where 1 = “not at all,” 5 = “neutral” and 9 = “very much.” Participants with known low BMD or previous fragility fractures were more willing to screen (median 9) than others (median 7) (overall interquartile range 6.5–9). We also asked a separate focus group to review patient key messages and provide feedback on an example of a decision aid. Participants provided feedback that easily accessible, simplified, patient-centred tools could help with understanding and shared decision-making (Appendix 7, available at www.cmaj.ca/lookup/doi/10.1503/cmaj.221219/tab-related-content).

Our systematic review found that females aged 50–65 years appear interested in screening (1 study),^49^ but there is likely heterogeneity in treatment acceptance when informed of treatment outcomes (11 studies).^42^ The high willingness to screen but low treatment acceptability suggests uncertainty. Decision aids may improve understanding of potential benefits and harms of preventive treatment^55^–^57^ and shared decision-making could better align screening and treatment with patient preferences.^58^–^60^

### Resource use

We did not conduct a systematic review of cost-effectiveness studies. However, cost-effectiveness analyses conducted on included RCTs showed that risk assessment–first screening (to reduce hip fractures) among “self-selected” females aged 65 years and older^44^ may be cost-effective (low-certainty evidence)^61^,^62^ (Appendix 8, available at www.cmaj.ca/lookup/doi/10.1503/cmaj.221219/tab-related-content).

A recommendation against screening males aged 40 years and older and females aged 40–64 years may reduce resource use where no health gains are expected. A risk assessment–first approach to screening females aged 65 years and older may reduce unnecessary BMD testing.

### Feasibility, acceptability and equity

Risk assessment–first screening may be acceptable given an increased emphasis on shared decision-making.^60^,^63^,^64^ However, access to family physicians and BMD testing varies by age, region (rural and remote v. urban areas), socioeconomic status and race or ethnicity.^65^–^67^ In 2015, 11% of Canadian females aged 65–79 years received a BMD.^16^ In 2009, 68% of Canadian females aged 65 years and older self-reported having received a BMD in their lifetime.^68^ Screening among women aged 50–64 years and men aged 40 years and older is likely occurring in Canada, with self-reported data from 2009 showing that 35% and 10%, respectively, had had at least 1 BMD.^68^ More recent age-standardized data in males aged 40 years and older show that 8.7% were screened in 2018/19^69^ and 15% of Ontario males aged 68–70 years (who had never been screened before) were screened in 2017/18.^70^

### Rationale

In the judgment of the task force, for females aged 65 years and older, the reduction in hip and clinical fragility fractures outweighs potential risks of overdiagnosis of high risk, nonserious adverse events and rare serious adverse events. This recommendation is conditional owing to low-certainty evidence and indirectness of trial populations (i.e., mostly in self-selected participants).

We recommend risk assessment–first screening, based on methods used in the RCTs, accuracy of Canadian FRAX and reported patient values. CAROC does not allow risk calculation without BMD and was not used in screening trials.

We recommend shared decision-making, based on patient acceptability and varying clinical FRAX thresholds for BMD access in the trials.^5^,^6^,^44^ This allows patients to consider preventive pharmacotherapy within their individual risk context (before BMD). Decision aids outlining individual fracture risk and possible treatment effectiveness may be useful.

Evidence regarding benefits for females aged 45–54 years and males aged 65 years and older was very uncertain. There was no evidence for females aged 55–64 years and males aged 40–64 years. Screening males is not standard practice in Canada and there has been low participation in screening^68^,^69^,^70^–^72^ despite existing Canadian recommendations to screen males aged 65 years and older.^12^ Considering the risk of overdiagnosis (of high risk) and risks of adverse events from potential medication, we recommend against screening females aged 40–64 years and males aged 40 years and older. There was no direct evidence establishing a benefit in these groups and there was low to moderate certainty for harms. The task force places a high value on not expending system-wide resources on interventions with no established benefit; hence, these recommendations are strong. This is consistent with the Grading of Recommendations, Assessment, Development and Evaluation (GRADE) approach,^36^,^37^ where strong recommendations can be based on very low-certainty evidence if there is evidence of harm or high resource implications.

## Methods

The task force is an independent panel of clinicians and methodologists who develop clinical practice guidelines supporting primary care providers in delivering preventive health care (www.canadiantaskforce.ca). A 6-member working group (G.T., R.G., S.K., D.L.R., J.J.R., B.D.T.) developed these recommendations with input from other task force members and scientific support from Public Health Agency of Canada (PHAC) staff.

We adhered to Appraisal of Guidelines for Research and Evaluation (AGREE), Guidelines International Network (GIN) and Guidance for Reporting Involvement of Patients and the Public (GRIPP-2) reporting guidance.^73^–^75^

The task force chose to develop guidance for screening to prevent fragility fractures because of the incidence and morbidity associated with these outcomes. New trial evidence, variation in practice and uncertain patient values and preferences added to the importance of this topic. The analytical framework and key questions are available in Appendix 9 (at www.cmaj.ca/lookup/doi/10.1503/cmaj.221219/tab-related-content).

Recommendations in this guideline were informed by systematic reviews^42^ on benefits and harms of screening, predictive accuracy of risk assessment tools, patient acceptability and benefits of treatment. We analyzed treatment harms via a rapid overview of reviews. The reviews^42^ and the protocol^43^ for the 4 systematic reviews and 1 rapid overview of reviews are available at www.canadiantaskforce.ca/guidelines/upcoming-guidelines/fragility-fractures/.

The Evidence Review and Synthesis Centre at the University of Alberta conducted the systematic reviews. Peer-reviewed databases (MEDLINE, Embase and Cochrane Library for all key questions; PsycINFO for acceptability of screening question) were searched from 2016 to July 4–8, 2019, for screening and predictive accuracy; from 2016 to Mar. 2, 2020, for treatment benefits (both updating evidence from an existing systematic review^13^); from 1995 to July 5, 2019, for acceptability; and from 2015 to June 24, 2020, for reviews on treatment harms.^42^ Full-search updates were conducted in June 2021 for predictive accuracy, patient acceptability and treatment harms (denosumab only), and in April 2022 for benefits and harms of screening.

Studies were included in the systematic and rapid reviews based on prespecified criteria.^43^ Potential benefits of screening include a decrease in hip fractures, clinical fragility fractures, fracture-related mortality, all-cause mortality and disability, and an improvement in functionality and quality of life. Potential harms included serious adverse events, nonserious adverse events, discontinuation because of adverse events and overdiagnosis of high risk (Appendix 9).

The working group rated the importance of outcomes following the GRADE approach.^34^ Outcomes rated as critical or important by patients (see Patient engagement section below) or task force members were included.

We used the GRADE approach to determine the certainty of evidence for each outcome and the strength of recommendations (Box 1).^34^ Appendix 10 (available at www.cmaj.ca/lookup/doi/10.1503/cmaj.221219/tab-related-content) provides the GRADE evidence-to-decision framework, which the task force reviewed and approved. The entire task force approved the recommendations.

More information about task force methods is available at https://canadiantaskforce.ca/methods/.

### Patient engagement

We conducted patient engagement through the Knowledge Translation group at St. Michael’s Hospital, Toronto. Phase 1 included 4 males and 21 females (aged ≥ 40 yr) recruited via advertisements on public advertisement websites and communications by related organizations. We conducted 4 surveys via 4 online focus groups (*n* = 25) between Aug. 29 and Oct. 5, 2018, to rate outcomes on a scale of 1–9, where 1–3 = not important, 4–6 = important and 7–9 = critical. Outcomes rated as critical or important were considered for inclusion in the systematic reviews. Participant characteristics, methodology and findings are available in Appendix 6.

In phase 2, 3 males and 3 females recruited from the Task Force Public Advisory Network (Appendix 11, available at www.cmaj.ca/lookup/doi/10.1503/cmaj.221219/tab-related-content) attended an online education session on this guideline (Oct. 28, 2021). In a subsequent session (Nov. 10, 2021), they provided feedback on key messages and opinions on interactive decision aids. Participant characteristics, methodology and findings are available in Appendix 7.

### External and content expert review

The protocol,^43^ systematic reviews^42^ and draft guideline were reviewed by stakeholders, peer reviewers and clinical experts. Clinical experts addressed technical and clinical considerations, by participating in working group meetings, examining supporting documents and reviewing the guideline. They were external to the task force and did not have input into nor did they vote on recommendations. Clinical and content experts who have assisted the task force in the guideline process may or may not agree with the task force recommendations.

### Management of competing interests

Task force funding is provided by PHAC. The task force follows GIN principles for managing competing interests.^76^,^77^ The oversight committee for evaluating and adjudicating competing interests included the task force chair (R.G., B.J.W.) during completion of this guideline and vice-chair (S.K.) and the director of PHAC’s Global Health and Guidelines Division.^77^ The task force does not consider the funding body’s views in developing recommendations.

Task force members disclose financial and other relevant interests when new topics are selected and at in-person meetings (3 per year). Disclosures are available at https://canadiantaskforce.ca/about/members/. Clinical experts disclose relevant interests initially and annually thereafter. We did not judge any disclosures to represent competing interests that precluded participation of task force members or clinical experts.

## Implementation

Considerations for implementation include a transition to risk assessment–first screening (if not currently performed) and a decrease in screening females aged 40–64 years and males (particularly in provinces with programs targeting males aged ≥ 65 yr).^78^,^79^ An interactive decision aid has been developed to help with implementation of risk assessment–first screening (https://frax.canadiantaskforce.ca). In those in whom screening is not recommended, knowledge translation should emphasize the lack of evidence of benefit and the potential harms.

Data underpinning the Canadian FRAX algorithm are limited for some racial and ethnic groups and should be interpreted cautiously. 80,81 Country-specific versions of FRAX are available (https://frax.shef.ac.uk/FRAX) as well as adjustments for Black, Hispanic and Asian populations in the United States FRAX; however, some concerns have been raised about the use of race- or ethnicity-based algorithms.^82^,^83^

We found no RCTs on screening intervals or age limits. Although some observational studies suggest intervals based on age, baseline BMD or absolute fracture risk, repeating BMD at 3–8 years did not improve fracture risk prediction.^13^,^38^–^41^ It is unknown how often to rescreen eligible females; however, rescreening within 8 years does not appear useful. Comorbidities and life expectancy should be considered for age limits and rescreening.

These recommendations emphasize the importance of good clinical practice, where clinicians are alert to changes in physical health and patient well-being. Clinicians should also be aware of the importance of secondary prevention (i.e., after fracture) and manage patients accordingly.

Clinical FRAX (without BMD) can be used in rural and remote areas where access to BMD is limited, as there was no meaningful difference in calibration compared with FRAX and BMD.^42^ However, no RCTs examined the use of risk assessment without adding BMD at some point, and therefore it is unknown whether risk assessment without BMD would lead to similar outcomes.

### Monitoring and evaluation

We suggest that implementation of risk assessment–first screening and uptake be evaluated. Screening rates among target and nontarget populations should be monitored. The task force will perform user testing and track interactive usage of its decision aid. It will also monitor and update the recommendations if new evidence becomes available.

## Other guidelines

Both the task force and Osteoporosis Canada recommend screening females aged 65–70 years and older.^12^,^84^ However, we recommend risk assessment–first, while Osteoporosis Canada recommends BMD testing first, followed by risk assessment.^12^,^84^ The 2010 Osteoporosis Canada guideline recommends informal risk assessment–first screening (based on a list of risk factors) for males and females aged 50–64 years (information was unavailable for its upcoming guideline).^12^ We recommend against screening males aged 40 years and older, whereas Osteoporosis Canada recommends BMD testing–first screening for males aged 65–70 years and older^12^,^84^ and the US Preventive Services Task Force in 2018 found insufficient evidence to make recommendations for males.^85^ Guidelines from the United Kingdom recommend risk assessment–first screening by age (females aged ≥ 65 yr, males aged ≥ 75 yr)^86^ or risk factors (i.e., case finding).^15^,^87^,^88^ Most North American guidelines recommend BMD testing–first screening in males and females aged 65–70 years and older, and informal or formal risk assessment–first screening for those aged 50–64 years^22^,^29^,^89^–^91^ (Table 1).

**Table 1:** Recommendations on screening to prevent fragility fractures

Organization	Recommendation
Society of Obstetricians and Gynaecologists of Canada, 2022^89^	All adults aged ≥ 65 yr should be screened for increased fracture risk by clinical evaluation and BMD assessment. Community-based screening in older females may be effective in reducing the incidence of hip fracture (conditional, moderate). In postmenopausal females < 65 yr, evaluate fracture risk clinically without BMD assessment (FRAX without BMD). A BMD assessment should be considered for patients with diseases or drugs associated with an increased risk of fracture or in the presence of a previous fragility fracture (conditional, low). If the FRAX score for MOF without BMD is > 10%, a BMD assessment should also be considered.
National Osteoporosis Guideline Group UK, 2022^15^	A FRAX assessment should be performed in any postmenopausal female, or male aged ≥ 50 yr, with a clinical risk factor for fragility fracture, to guide BMD measurement and prompt timely referral or drug treatment or both, where indicated (strong recommendation). Males and females with intermediate fracture risk (i.e., between the upper and lower assessment thresholds) should be referred for BMD measurement, if practical. Thereafter, fracture probability should be reassessed using FRAX (strong recommendation). Vertebral fracture assessment is indicated in postmenopausal females, and males aged ≥ 50 yr, if there is a history of ≥ 4 cm height loss, kyphosis, recent or current long-term oral glucocorticoid therapy, a BMD T-score ≤ −2.5 at either the spine or hip, or in cases of acute-onset back pain with risk factors for osteoporosis (strong recommendation).
The Bone Health and Osteoporosis Foundation (formerly the National Osteoporosis Foundation) (USA), 2022^22^	BMD testing should be performed in the following: Females aged ≥ 65 yr and men aged ≥ 70 yr Postmenopausal females and males aged 50–69 yr, based on risk profile Postmenopausal females and males aged ≥ 50 yr with history of adult-age fracture DXA facilities that employ accepted quality assurance measures The same facility and on the same densitometry device for each test whenever possible To detect subclinical vertebral fractures, vertebral fracture imaging (radiograph or DXA vertebral fracture assessment) should be performed in the following: Females aged ≥ 65 yr if T-score is ≤ −1.0 at the femoral neck Females aged ≥ 70 yr and males aged ≥ 80 yr if T-score is ≤ −1.0 at the lumbar spine, total hip or femoral neck Males aged 70–79 yr if T-score is ≤ −1.5 at the lumbar spine, total hip or femoral neck Postmenopausal females and males aged ≥ 50 yr with specific risk factors
The American College of Obstetricians and Gynecologists, 2021^90^	Screening for osteoporosis is recommended in postmenopausal patients ≥ 65 yr with BMD testing to prevent osteoporotic fractures (strong recommendation, high-quality evidence). Screening for osteoporosis with BMD testing is recommended to prevent osteoporotic fractures in postmenopausal patients < 65 yr who are at increased risk of osteoporosis, as determined by a formal clinical risk assessment tool (strong recommendation, high-quality evidence).
Scottish Intercollegiate Guidelines Network, 2021 update of 2015 report^87^	People aged ≥ 50 yr with a history of fragility fractures should be offered DXA scanning to evaluate the need for anti-osteoporosis therapy. Fracture risk assessment should be carried out, preferably using QFracture, before DXA in patients with clinical risk factors for osteoporosis and in whom anti-osteoporosis treatment is being considered. Measurement of BMD by DXA at the spine and hip should be carried out after fracture risk assessment in patients in whom anti-osteoporosis treatment is being considered. Population-based screening for fracture risk and an offer of treatment for those at high risk of fracture is not recommended as a means of reducing MOFs.
American Association of Clinical Endocrinologists and American College of Endocrinology, 2020^29^	Postmenopausal osteoporosis guidelines: Evaluate all postmenopausal females aged ≥ 50 yr for osteoporosis risk (grade B; BEL 1, downgraded owing to gaps in evidence). A detailed history, physical examination and clinical fracture risk assessment with FRAX or other fracture risk assessment tool should be included in the initial evaluation for osteoporosis (grade B; BEL 1). AACE recommends BMD testing for females aged ≥ 65 yr and younger postmenopausal females at increased risk for bone loss and fracture, based on analysis of fracture risk.
UK National Screening Committee, 2019^88^	Screening for osteoporosis is not recommended in postmenopausal females.
US Preventive Services Task Force, 2018^85^	Screening for osteoporosis with bone measurement testing is recommended to prevent osteoporotic fractures in females aged ≥ 65 yr (B recommendation). Screening for osteoporosis with bone measurement testing is recommended to prevent osteoporotic fractures in postmenopausal females aged < 65 yr at increased risk of osteoporosis, as determined by a formal clinical risk assessment tool (B recommendation). The USPSTF concludes that the current evidence is insufficient to assess the balance of benefits and harms of screening for osteoporosis to prevent osteoporotic fractures in males (I statement).
National Institute for Health and Care Excellence, 2017^86^	Assessment of fracture risk should be considered: In all females aged ≥ 65 yr and all males aged ≥ 75 yr In females aged < 65 yr and males aged < 75 yr in the presence of risk factors Either FRAX (without a BMD value if a DXA scan has not previously been undertaken) or QFracture, within the tools’ allowed age ranges, should be used to estimate 10-yr predicted absolute fracture risk when assessing risk of fracture. Above the upper age limits defined by the tools, people should be considered to be at high risk. BMD should not be routinely measured to assess fracture risk without previous assessment using FRAX (without a BMD value) or QFracture. After risk assessment with FRAX (without a BMD value) or QFracture, measuring BMD with DXA should be considered in people whose fracture risk is in the region of an intervention threshold for a proposed treatment, and absolute risk recalculated using FRAX with the BMD value.
American College of Radiology, 2016^91^	BMD screening should be carried out for: All females aged ≥ 65 yr and males aged ≥ 70 yr (asymptomatic screening) Females aged < 65 yr who have additional risk for osteoporosis, based on medical history and other findings Females aged < 65 yr or males aged < 70 yr who have additional risk factors People of any age with bone mass osteopenia or fragility fractures on imaging studies such as radiographs, computed tomography or magnetic resonance imaging People aged ≥ 50 yr who develop a wrist, hip, spine or proximal humerus fracture with minimal or no trauma, excluding pathologic fractures People of any age who develop ≥ 1 insufficiency fractures People being considered for pharmacologic therapy for osteoporosis
Osteoporosis Canada, 2010^12^*	People aged ≥ 65 yr should have a BMD test. Menopausal females, and males aged 50–64 yr with clinical risk factors for fracture, should have a BMD test. Height should be measured annually, and the presence of vertebral fractures should be assessed (grade A). History of falls in the past year should be assessed. If there has been such a fall, a multifactorial risk assessment should be conducted, including the ability to get out of a chair without using arms (grade A).

Note: AACE = American Association of Clinical Endocrinologists, ACOG = American College of Obstetricians and Gynecologists, BEL = best evidence level, BMD = bone mineral density, DXA = dual-energy x-ray absorptiometry, FRAX = Fracture Risk Assessment Tool, MOF = major osteoporotic fracture, NSC = National Screening Committee, USPSTF = US Preventive Services Task Force.

* The upcoming 2023 Osteoporosis Canada guideline was unavailable for review. However, a 2020 analysis supporting the upcoming guideline^84^ suggested the following for males and females: “BMD testing is indicated at age 70 if no additional FRAX clinical risk factors are present, or at age 65 if one or more clinical risk factors exists.”

## Gaps in knowledge

High-quality trials examining benefits and harms of screening in males and younger females are needed. Evidence on screening frequency and age to stop screening is lacking. Studies of emerging harms (i.e., rebound fractures after preventive pharmacotherapy is stopped) are limited or have very low-certainty evidence. Given differing fracture rates among populations,^92^–^94^ more work is required to refine screening tools for risk of fragility fracture to ensure that health care inequities are not perpetuated.

## Limitations

There were limited and very low-certainty data for females (40–64 yr) and males (≥ 40 yr). In females aged 65 years and older, applicability of the “self-selected” population, higher socioeconomic status of the participants and the mailed invitation recruitment was a concern, resulting in down-rating for indirectness in GRADE.^42^

Treatment harms were studied via a rapid overview of systematic reviews, which may exclude recent RCTs. However, a prepublication search update (June 2021), targeted to emerging harms, found no new RCTs.

Additionally, limited data exist on regional and socioeconomic factors or implications for transgender populations.

## Conclusion

We recommend risk assessment–first screening for females aged 65 years and older with the Canadian clinical FRAX (without BMD) to facilitate shared decision-making about possible benefits and harms of preventive pharmacotherapy. If pharmacotherapy is being considered, we recommend adding BMD to FRAX to re-estimate risk (conditional recommendation, low-certainty evidence). We recommend against screening females aged 40–64 years and males aged 40 years and older because of a lack of evidence establishing a benefit, potential harms, and increased health care resource use (strong recommendation, very low-certainty evidence).

## Supplementary Material

Appendix 1: Benefits of screening to prevent fragility fractures

Appendix 10: GRADE Evidence to Decision Framework

Appendix 11: Task Force Public Advisors Network (TFPAN) Protocol

Appendix 2: Benefits of pharmacologic treatments

Appendix 3: Calculations for overdiagnosis

Appendix 4: Harms (pharmacological) of screening to prevent fragility fractures

Appendix 5: Accuracy of risk asesment tools (calibrated for Canada)

Appendix 6: Patient preferences for screening to prevent fragility factures: Data summary

Appendix 7: Fragility Fractures Community Jury: Data Summary Report

Appendix 8: Cost-effectivenes Analyses of 2-step Screening via SCOOP RCT

Appendix 9: Analytical Framework
