# Supplementary material for: Recommendations on screening for primary prevention of fragility fractures
Source: CMAJ. 2023 May 8;195(18):E639–49. doi: 10.1503/cmaj.221219 (PMC10166624; doi:10.1503/cmaj.221219)
Supplement: Appendix 1: Benefits of screening to prevent fragility fractures [file 221219-guide-1-at.pdf]

Appendix 1: Benefits of screening to prevent fragility fractures

Outcome	Study approach; Population	Included studies; Sample size; Follow-up	Anticipated absolute effects*			Hazard ratio (95% CI)	Certainty
			Assumed population risk*	Risk with screening (95% CI)	Absolute difference (95% CI)		
**Hip fractures**	Offer-to-screen in "self-selected" population; Females ≥65 years	3 RCTs + 1 CCT (1–4); n=43,736; Follow-up: 3-5 years	Control event rate (study data)			0.80 (0.71 to 0.91)	Moderate to High[c]
			31 per 1000	24.8 per 1000 (22.0 to 28.2)	6.2 fewer per 1000 (9.0 fewer to 2.8 fewer)		
			General population risk				
			20 per 1000	16.0 per 1000 (14.2 to 18.2)	4.0 fewer per 1000 (5.8 fewer to 1.8 fewer)		
	"All eligible" / offer-to-screen; Females ≥65 years	1 RCT (2); n=34,229; Follow-up: 5 years	Control event rate (study data)			0.99 (0.88 to 1.11)	Low[a-c]
			35 per 1000	34.7 per 1000 (30.8 to 38.9)	0.3 fewer in 1000 (4.2 fewer to 3.9 more)		
			General population risk				
			20 per 1000	19.8 per 1000 (17.6 to 22.2)	0.2 fewer in 1000 (2.4 fewer to 2.2 more)		
	"All eligible" / offer-to-screen; Females 45-54 years	1 RCT (5); n=2,797; Follow-up: 9 years	Control event rate (study data)			0.95 (0.19 to 4.71)	Very Low[a-d]
			2 per 1000	1.9 per 1000 (0.4 to 9.42)	0.1 fewer in 1000 (1.6 fewer to 7.4 more)		
			General population risk				
			8 per 1000	7.6 per 1000 (1.5 to 37.7)	0.4 fewer in 1000 (6.5 fewer to 29.7 more)		
	Acceptors of screening; Females 45-54 years	1 RCT (5); n=2,604; Follow-up: 9 years	Control event rate (study data)			0.37 (0.04 to 3.52)	Very Low[a-d]
			2 per 1000	0.7 per 1000 (0.1 to 7.0)	1.3 fewer per 1000 (1.9 fewer to 5.0 more)		
			General population risk				
			8 per 1000	3.0 per 1000 (0.3 to 28.2)	5.0 fewer per 1000 (7.7 fewer to 20.2 more)		
	Offer-to-screen in "self-selected" population; Males ≥65 years	1 CCT (4); n=1,380; Follow-up: 4.9 years	Control event rate (study data)			0.68 (0.32 to 1.43)	Very Low to Low[a-d]
			30 per 1000	20.4 per 1000 (9.6 to 42.9)	9.6 fewer per 1000 (20.4 fewer to 12.9 more)		
			General population risk				
			16 per 1000	10.9 per 1000 (5.1 to 22.9)	5.1 fewer per 1000 (10.9 fewer to 6.9 more)		
**All clinical fragility fractures**	Offer-to-screen in "self-selected" population; Females ≥65 years	3 RCTs (1–3); n=42,009; Follow-up: 3-5 years	Control event rate (study data)			0.93 (0.87 to 0.99)	Moderate[c]
			84 per 1000	78.1 per 1000 (73.1 to 83.2)	5.9 fewer per 1000 (10.9 fewer to 0.8 fewer)		
			General population risk				
			168 per 1000	156.2 (146.2 to 166.3)	11.8 fewer per 1000 (21.8 fewer to 1.7 fewer)		
	"All eligible" / offer-to-screen; Females ≥65 years	1 RCT (2); n=34,229; Follow-up: 5 years	Control event rate (study data)			0.99 (0.92 to 1.06)	Low[a-c]
			100 per 1,000	99.0 per 1000 (92.0 to 106.0)	1.0 fewer per 1,000 (8.0 fewer to 6.0 more)		
			General population risk				
			168 per 1,000	166.3 per 1,000 (154.6 to 178.1)	1.7 fewer per 1,000 (13.4 fewer to 10.1 more)		
	"All eligible" / offer-to-screen;	1 RCT (5); n=2,797;	Control event rate (study data)			1.01 (0.68 to 1.50)	Very Low[a-d]
			34 per 1,000	34.3 per 1000 (23.1 to 51.0)	0.3 more per 1,000 (10.9 fewer to 17.0 more)		
			General population risk				

Appendix 1, as supplied by the authors. Appendix to: Thériault G, Limburg H, Klarenbach S, et al. Recommendations on screening for primary prevention of fragility fractures. *CMAJ* 2023. doi: 10.1503/cmaj.221219. Copyright © 2023 The Author(s) or their employer(s). To receive this resource in an accessible format, please contact us at cmajgroup@cmaj.ca.

Outcome	Study approach; Population	Included studies; Sample size; Follow-up	Anticipated absolute effects*			Hazard ratio (95% CI)	Certainty
			Assumed population risk*	Risk with screening (95% CI)	Absolute difference (95% CI)		
	Females 45-54 years	Follow-up: 9 years	67 per 1,000	67.7 per 1000 (45.6 to 100.5)	0.7 more per 1,000 (21.4 fewer to 33.5 more)		
	Acceptors of screening; Females 45-54 years	1 RCT (5); 2,604; Follow-up: 9 years	Control event rate (study data)			0.73 (0.46 to 1.14)	Very Low[a-d]
			34 per 1,000	24.8 per 1,000 (15.6 to 38.8)	9.2 fewer per 1,000 (18.4 fewer to 4.8 more)		
			General population risk				
			67 per 1,000	48.9 per 1,000 (30.8 to 76.4)	18.1 fewer per 1,000 (36.2 fewer to 9.4 more)		

CCT: clinical controlled trial; CI: confidence interval; RCT: randomized controlled trial
*The absolute effect (and its 95% CI) without screening (i.e., assumed population risk) is based on the estimated risk in the comparison group; the risk with screening is based on applying the relative effect (hazard ratio) of the intervention (and its 95% CI) to the assumed population risk. Study data refers to the median control events rates across trials. The assumed population risk for the general risk (Canadian) population are estimated from Prior et al., 2015 based on 10 year follow-up (6)

a=risk of bias; b=inconsistency; c=indirectness; d=imprecision

References

1. Merlijn T, Swart KM, Schoor NM, Heymans MW, Zwaard BC, Heijden AA, et al. The Effect of a Screening and Treatment Program for the Prevention of Fractures in Older Women: A Randomized Pragmatic Trial. J Bone Miner Res. 2019 Nov;34(11).
2. Rubin KH, Rothmann MJ, Holmberg T, Hoiberg M, Moller S, Barkmann R, et al. Effectiveness of a two-step population-based osteoporosis screening program using FRAX: the randomized Risk-stratified Osteoporosis Strategy Evaluation (ROSE) study. Osteoporos Int. 2018 Mar;29(3):567–78.
3. Shepstone L, Lenaghan E, Cooper C, Clarke S, Fong-Soe-Khioe R, Fordham R, et al. Screening in the community to reduce fractures in older women (SCOOP): a randomised controlled trial. Lancet. 2018;391(10122):741–7.
4. Kern LM, Powe NR, Levine MA, Fitzpatrick AL, Harris TB, Robbins J, et al. Association between screening for osteoporosis and the incidence of hip fracture. Ann Intern Med. 2005 Feb;142(3):173–81.
5. Barr RJ, Stewart A, Torgerson DJ, Reid DM. Population screening for osteoporosis risk: a randomised control trial of medication use and fracture risk. Osteoporos Int. 2010 Apr;21(4):561–8.
6. Prior JC, Langsetmo L, Lentle BC, Berger C, Goltzman D, Kovacs CS, et al. Ten-year incident osteoporosis-related fractures in the population-based Canadian Multicentre Osteoporosis Study - comparing site and age-specific risks in women and men. Bone. 2015 Feb;71:237–43.

Appendix 1, as supplied by the authors. Appendix to: Thériault G, Limburg H, Klarenbach S, et al. Recommendations on screening for primary prevention of fragility fractures. *CMAJ* 2023. doi: 10.1503/cmaj.221219. Copyright © 2023 The Author(s) or their employer(s). To receive this resource in an accessible format, please contact us at cmajgroup@cmaj.ca.
