# Supplementary material for: Recommendations on screening for primary prevention of fragility fractures
Source: CMAJ. 2023 May 8;195(18):E639–49. doi: 10.1503/cmaj.221219 (PMC10166624; doi:10.1503/cmaj.221219)
Supplement: Appendix 10: GRADE Evidence to Decision Framework [file 221219-guide-10-at.pdf]

Appendix 10: GRADE Evidence to Decision Framework

Question: Should we screen adults ≥40 years to prevent fragility fractures?

**POPULATION:**	Adults ≥40 years old (community dwelling)	**BACKGROUND:**<br><br>Fragility fractures are broken bones that result from a minor fall or normal activity that usually should not cause a fracture in healthy adults (1). These fractures occur due to weakened bone structure often referred to as osteoporosis (2). The most common sites of fragility fractures are the hip, spine and wrist (2). As people age, old bone cells may not be replaced by new cells as quickly resulting in brittle or fragile bones (3). This gradual loss of bone density and strength increases the risk of fracture. Risk factors for fragility fracture include low bone density, female sex[1], older age, lower body weight, prior fragility fracture, parental history of hip fracture, a history of falls, chronic use of certain medications (e.g., glucocorticoids), smoking, higher levels of alcohol use, and living with diabetes and/or rheumatoid arthritis (4–8). Post-menopausal females are at a greater risk due to additional bone loss associated with menopause (9).<br><br>The annual rate of hip fractures among Canadians was 168 per 100,000 (age 65-79 years) and 1,045 per 100,000 (age 80+ years) in 2016 (10). The annual rate for any type of fracture was 843 and 2,642 per 100,000 (ages 65-79 and 80+ years respectively) (10). In the 2010/2011 fiscal year, among Canadians ≥50 years, there were 131,443 fragility fractures associated with 64,884 acute care admissions and 983,074 hospitalized days (11). The cost of fragility fractures was estimated at $4.6 billion
**INTERVENTION:**	**KQ1**: Screening to prevent fragility fractures (BMD-first or risk assessment-first screening strategies)<br><br>**KQ2**: Validated fracture risk assessment tool (with or without BMD)<br><br>**KQ3**: Alendronate, risedronate, zoledronic acid, denosumab, any bisphosphonate<br><br>**KQ4:** N/A	
**COMPARISON:**	**KQ1a**: No screening; KQ1b: other screening strategies<br><br>**KQ2**: N/A<br><br>**KQ3**: No treatment or placebo<br><br>**KQ4**: N/A	
**MAIN OUTCOMES:**	<u>**Critical:**</u><br>**KQ1/3:** <u>Benefits</u>: Reductions in hip fractures, all clinical fragility fractures and fracture-related	

[1] The terms "female" and "male" are referring to sex (i.e., biological attributes, particularly the reproductive or sexual anatomy at birth), unless otherwise indicated.

Appendix 10, as supplied by the authors. Appendix to: Thériault G, Limburg H, Klarenbach S, et al. Recommendations on screening for primary prevention of fragility fractures. *CMAJ* 2023. doi: 10.1503/cmaj.221219. Copyright © 2023 The Author(s) or their employer(s). To receive this resource in an accessible format, please contact us at cmajgroup@cmaj.ca.

	mortality. Increased functionality and disability, quality of life or wellbeing.<br>Harms: Serious adverse events (including all serious cardiovascular events; serious cardiac rhythm disturbances (e.g. atrial fibrillation or ventricular arrhythmia); serious gastrointestinal (GI) events (excluding cancers); GI cancer; atypical femoral fractures; osteonecrosis of the jaw; rebound fractures.<br><br>**Important:**<br>**KQ1/3:** Benefits: Reduction in all-cause mortality<br>Harms: Overdiagnosis (KQ1 only), discontinuation due to adverse events, non-serious adverse events (including any adverse events or adverse (drug) reactions; any non-serious adverse events)<br><br>**KQ2:** Calibration for 5 and 10 year fracture risk of hip and all clinical fractures<br>**KQ4:** Acceptability of screening and/or treatment, willingness or intention to screen or initiate treatment, magnitude of benefit to make screening and/or treatment acceptable	including acute, rehabilitation and long-term care as well as prescription drug cost, wage loss and home care (11).<br><br>The World Health Organization (WHO) suggests that BMD T-score of less than -2.5 (2.5 or more standard deviations below the reference mean) is a significant risk factor for fragility fractures and refers to it as osteoporosis (12). The prevalence of low bone density (<-2.5) in Canada among aged 65-79 years was 17.9 % and 23.6% for those aged 80+ years (10). Among Canadians over 40 years of age, females were four times more likely to report having osteoporosis than males (13).<br><br>A common screening tool for the risk of fragility fracture is the measurement of bone mineral density (BMD) at the femoral neck (hip) using DXA (dual energy X-ray absorptiometry) (4). The absolute fracture risk can be estimated (with or without BMD) using a risk assessment tool (e.g. FRAX, QFracture (UK Fracture risk assessment), Garvan (Australian Fracture risk assessment), CAROC (Canadian risk assessment)) which provides a percentage risk or risk category for hip or osteoporotic fracture in the next 10 years (14). The goal of screening is to allow clinicians to identify those at risk for fragility fractures and provide appropriate treatment. In 2015, 7.1% of Canadians reported receiving a BMD test (10).<br><br>Pharmacological treatment to prevent fragility fractures includes first-line therapy for post-menopausal females of bisphosphates (alendronate, risedronate or zoledronic acid) or denosumab (15). Recommended first line therapy for males includes alendronate, risedronate and zoledronic acid (4). Adequate intake of calcium and
**SETTING:**	Primary care in Canada	

Appendix 10, as supplied by the authors. Appendix to: Thériault G, Limburg H, Klarenbach S, et al. Recommendations on screening for primary prevention of fragility fractures. *CMAJ* 2023. doi: 10.1503/cmaj.221219. Copyright © 2023 The Author(s) or their employer(s). To receive this resource in an accessible format, please contact us at cmajgroup@cmaj.ca.

**PERSPECTIVE:**	Population	vitamin D along with exercise, smoking cessation and fall prevention strategies are also recommended following a diagnosis of elevated fracture risk (2,4).<br><br>The consequences of fragility fractures include significant morbidity due to disability and chronic pain as well as hospitalization and long-term care admission (4,16,17). Quality of life can be significantly impacted following a fragility fracture, with lower health utility index scores and considerable deficits on mobility and self-care indicators (4,16–18). Fragility fractures also result in a higher mortality particularly among elderly patients with significant comorbidities (19). In fact, patients with bone density have approximately 1.5 times the mortality risk for each standard deviation (T-score) decrease in BMD (20). Since the risk of low bone density increases with age, comorbidities such as diabetes and hypertension are common and increase the risk of mortality following a fracture. However, hip fracture is associated with a 10-45% mortality rate within the first year and an estimated 26-28% of deaths among vertebral fracture patients can be attributed to the fracture alone (20). The increased mortality rates associated with low bone density is also affected by the location of the fragility fracture (e.g. hip), patient sex and ethnicity (20).<br><br>Screening to prevent fragility fractures may itself cause unintended consequences associated with the screening test, diagnosis and/or treatment. Overdiagnosis can also occur with the identification of high risk in individuals that if not screened would never have known they were at risk and would never experience a fracture.

Assessment

Appendix 10, as supplied by the authors. Appendix to: Thériault G, Limburg H, Klarenbach S, et al. Recommendations on screening for primary prevention of fragility fractures. *CMAJ* 2023. doi: 10.1503/cmaj.221219. Copyright © 2023 The Author(s) or their employer(s). To receive this resource in an accessible format, please contact us at cmajgroup@cmaj.ca.

	JUDGEMENT	RESEARCH EVIDENCE	ADDITIONAL CONSIDERATIONS
PROBLEM	**Is the problem a priority?**<br><br>○No<br>○Probably no<br>○Probably yes<br>**X** Yes<br><br>○Varies<br>○Don't know	Screening for the risk of fragility fractures was judged by the Task Force to be a priority problem. This is based on incidence of fragility fractures in Canada and the associated consequences (reduced quality of life, increased morbidity and mortality). There may also be variations in practice and uncertain patient values and preferences.<br><br>**Number of people affected (burden)**<br>• The annual rate of hip fractures among Canadians was 168 per 100,000 (age 65-79 years) and 1,045 per 100,000 (age 80+ years) in 2016 (10).<br>• The annual rate for any type of fracture was 843 and 2,642 per 100,000 (ages 65-79 and 80+ years respectively) (10).<br>• The cost of fragility fractures (fiscal year 2010/2011) was estimated at $4.6 billion including acute, rehabilitation and long-term care as well as prescription drug cost, wage loss and home care (11).<br>**Potential Consequences**<br>• Fewer than 50 % of Canadians who experience a hip fracture will have a full recovery, and many are permanently disabled (21).<br>• Approximately 25 % of patients will need a nursing home or assisted living care for a year or more after a hip fracture (21).<br>• Mortality was significantly increased among Canadian females 1 year post hip fracture (HR=3.0, 95%CI 1.0-8.7) and post vertebral fracture (HR=3.7, 95%CI 1.1-12.8) (19).<br><br>**Uncertainty for practice**<br>• Among females aged 50–64 years, 23.1% of females were identified for BMD testing under the USPSTF guidelines and 52.3% under the Osteoporosis Canada guidelines. Osteoporosis Canada also recommends screening males >=65 years with BMD however (4), the USPSTF found insufficient evidence to make a recommendation for males (6).<br>• Canadian and American guidelines recommend a BMD-first screening approach for females over age 65 while European guidelines recommend a risk assessment-first screening (FRAX or QFracture followed by BMD) (4,6,7,22).	

Appendix 10, as supplied by the authors. Appendix to: Thériault G, Limburg H, Klarenbach S, et al. Recommendations on screening for primary prevention of fragility fractures. *CMAJ* 2023. doi: 10.1503/cmaj.221219. Copyright © 2023 The Author(s) or their employer(s). To receive this resource in an accessible format, please contact us at cmajgroup@cmaj.ca.

		There is also considerable uncertainty in patient values and preferences (particularly around acceptability of treatment), as there are no comprehensive reviews available.	

Appendix 10, as supplied by the authors. Appendix to: Thériault G, Limburg H, Klarenbach S, et al. Recommendations on screening for primary prevention of fragility fractures. *CMAJ* 2023. doi: 10.1503/cmaj.221219. Copyright © 2023 The Author(s) or their employer(s). To receive this resource in an accessible format, please contact us at cmajgroup@cmaj.ca.

<table>
<tr>
<td>DESIRABLE EFFECTS</td>
<td>How substantial are the desirable anticipated effects?

○ Trivial
X Small (Females ≥65 years)
○Moderate
○Large

○Varies
X Don't know (Males ≥40 years and females 40-64 years)</td>
<td>KQ1a: What are the <u>benefits</u> of screening compared with no screening to prevent fragility fractures and related morbidity and mortality in primary care for adults ≥ 40 years?

<u>SUMMARY: JUDGEMENT OF BENEFITS</u>

All Eligible: (Offer-to-screen in general population, females 45-54 years)
Screening <u>may not reduce all-cause mortality</u>. The evidence for hip and clinical fragility fractures is very uncertain.

All Eligible (Offer-to-screen to females ≥65 years in the general population)
Screening <u>may not reduce hip fractures, clinical fragility fractures, or all-cause mortality</u>.

“Selected Populations” (Offer-to-screen to females ≥65 years willing to independently complete a mailed fracture risk questionnaire, females ≥65 years)
Screening <u>probably reduces hip fractures</u> and <u>probably slightly reduces clinical fragility fractures</u>. It <u>probably does not reduce all-cause mortality and there may be little-to-no difference in quality of life.</u>

“Selected Populations” (Offer-to-screen to males ≥65 years willing to complete a cardiovascular health study and attend a follow-up visit): The evidence for hip fragility fractures is very uncertain.

No studies reported on fracture-related mortality, functionality or disability.
No studies reported on men 40-64 years.</td>
<td><u>Definitions:</u>
Fracture risk assessment tools (e.g. FRAX, CAROC)
- Provide a 10-year probability of fracture based on clinical risk factors with or without bone mineral density.

Bone mineral densitometry (BMD)
- Also called “dual-energy x-ray absorptiometry” (DXA or DEXA)
- Includes BMD of either the hip and/or spine
- Provides a T-score of a patient’s BMD (standard deviation from the average healthy adult BMD)

BMD-first screening:
All patients go <u>directly to BMD</u> without initial fracture risk assessment (may also include post</td>
</tr>
</table>

Appendix 10, as supplied by the authors. Appendix to: Thériault G, Limburg H, Klarenbach S, et al. Recommendations on screening for primary prevention of fragility fractures. *CMAJ* 2023. doi: 10.1503/cmaj.221219. Copyright © 2023 The Author(s) or their employer(s). To receive this resource in an accessible format, please contact us at cmajgroup@cmaj.ca.

EVIDENCE TABLES

Table 1.1: Hip fractures

- The evidence about all eligible / offer-to-screen populations (females 45-54 years) is very uncertain.
- **Screening may not reduce hip fracture in *all eligible / offer-to-screen populations* (females 68-80 years).**
- The evidence about acceptors of screening (females 45-54 years) is very uncertain.
- **Screening probably reduces hip fractures in *selected populations*[2] (females ≥65 years; 4.0-6.2 fewer per 1000, NNS=250). (3-5 year follow-up)**
- The evidence for offer-to-screen in *selected*[3] *populations* (males≥65 years) is very uncertain.

Study approach	Population<br><br>Studies[4]; Sample size	Anticipated absolute effects			Relative HR (95% CI)	Certainty	Judgement
		Assumed population risk*	Risk with screening (95% CI)	Absolute difference (95% CI)			
All eligible / offer-to-screen	Females<br><br>45-54 y;<br><br>1 RCT (23) (APOSS); 2,797<br><br>Follow-up: 9 years	Control event rate (study data)			0.95 (0.19 to 4.71)	Very Low[a-d]	Very uncertain
		2 per 1000	1.9 per 1000 (0.4 to 9.42)	0.1 fewer in 1000 (1.6 fewer to 7.4 more)			
		General population risk*					
		8 per 1000	7.6 per 1000 (1.5 to 37.7)	0.4 fewer in 1000 (6.5			

BMD fracture risk assessment (e.g. FRAX+BMD) to calculate treatment eligibility)

Risk assessment-first screening
- Fracture risk assessment (e.g. FRAX), followed by BMD only if necessary (e.g. based on threshold or shared decision making). Fracture risk should then be re-estimated using FRAX + BMD to calculate treatment eligibility.

All clinical fragility fractures (includes sub-outcomes of
(a) Fractures reported in study as "clinical fractures"
(b) Add up non-vertebral + clinical vertebral
(c) (*Note: Only used for KQ3a and*

[2] Females ≥65 years willing to independently complete a mailed fracture risk questionnaire
[3] Males ≥65 years who were study participants in the CHS - Cardiovascular Health Study (i.e. willing to complete the CHS and attend a follow-up visit)
[4] See Appendix for details on individual studies

Appendix 10, as supplied by the authors. Appendix to: Thériault G, Limburg H, Klarenbach S, et al. Recommendations on screening for primary prevention of fragility fractures. *CMAJ* 2023. doi: 10.1503/cmaj.221219. Copyright © 2023 The Author(s) or their employer(s). To receive this resource in an accessible format, please contact us at cmajgroup@cmaj.ca.

				fewer to 29.7 more)			
	Females<br><br>68-80 y;<br><br>1 RCT (ROSE) (24); 34,229<br><br>Follow-up: 5 years	Control event rate (study data)			0.99 (0.88 to 1.11)	Low[a-c]	<u>**May not reduce**</u>
		35 per 1000	34.7 per 1000 (30.8 to 38.9)	0.3 fewer in 1000 (4.2 fewer to 3.9 more)			
		General population risk*					
		20 per 1000	19.8 per 1000 (17.6 to 22.2)	0.2 fewer in 1000 (2.4 fewer to 2.2 more)			
Acceptors of screening	Females<br><br>45-54 y;<br><br>1 RCT (23) (APOSS); 2,604<br><br>Follow-up: 9 years	Control event rate (study data)			0.37 (0.04 to 3.52)	Very Low[a-d]	Very uncertain
		2 per 1000	0.7 per 1000 (0.1 to 7.0)	1.3 fewer per 1000 (1.9 fewer to 5.0 more)			
		General population risk*					
		8 per 1000	3.0 per 1000 (0.3 to 28.2)	5.0 fewer per 1000 (7.7 fewer to 20.2 more)			
Offer-to-screen in <u>selected population</u>[2]	Females ≥65 y;<br><br>3 RCTs + 1 CCT (SALT, SCOOP,	Control event rate (study data)			0.80 (0.71 to 0.91)	Moderate to High[c]	<u>**Probably reduces**</u>
		31 per 1000	24.8 per 1000 (22.0 to 28.2)	**6.2 fewer per 1000 (9.0 fewer to 2.8 fewer)**			
		General population risk*					

sensitivity analysis showed no effect on the conclusions). All fractures (reported in study as "all fractures" or add up non-vertebral + any vertebral (clinical, morphologic or unstated)

Major osteoporotic fracture (MOF)
In the literature the term MOF (major osteoporotic fracture) is often defined as a fracture of the hip, spine (clinical), wrist or humerus.

The 10 years fracture risk for females in Canada: 6.7% 35-44 years; 8.3% 45-54 years; 13.9% 55-64 years; 31.8% 75-84 years (Canadian multicenter osteoporosis study 2015) (27)

A similar systematic review of SCOOP, ROSE and SALT (98)

Appendix 10, as supplied by the authors. Appendix to: Thériault G, Limburg H, Klarenbach S, et al. Recommendations on screening for primary prevention of fragility fractures. *CMAJ* 2023. doi: 10.1503/cmaj.221219. Copyright © 2023 The Author(s) or their employer(s). To receive this resource in an accessible format, please contact us at cmajgroup@cmaj.ca.

	ROSE + Kern) (24–27); 43,736<br><br>Follow-up: 3-5 years	20 per 1000	16.0 per 1000 (14.2 to 18.2)	**4.0 fewer per 1000 (5.8 fewer to 1.8 fewer)**			
Offer-to-screen in <u>selected population</u>[3]	Males ≥65 y;<br><br>1 CCT (Kern) (27); 1,380<br><br>Follow-up: 4.9 years	Control event rate (study data)			0.68 (0.32 to 1.43)	Very Low to Low[a-d]	Very uncertain
		30 per 1000	20.4 per 1000 (9.6 to 42.9)	9.6 fewer per 1000 (20.4 fewer to 12.9 more)			
		General population risk*					
		16 per 1000	10.9 per 1000 (5.1 to 22.9)	5.1 fewer per 1000 (10.9 fewer to 6.9 more)			

CCT: clinical controlled trial; CI: confidence interval; RCT: randomized controlled trial; y: years
a=risk of bias; b=inconsistency; c=indirectness; d=imprecision
*The effects without screening for the general risk population are estimated from Prior et al., 2015, based on 10 year follow-up (28)

Table 1.2: Clinical fragility fractures

- The evidence for all eligible / offer-to-screen (females 45-54 years) is very uncertain.
- **Screening <u>may not reduce</u> clinical fragility fractures in *all eligible / offer-to-screen*** (females 68-80 years).
- **Screening <u>probably slightly reduces</u> clinical fragility fractures in *selected populations*[5] (females ≥65 years; 5.9-11.8 fewer per 1000, NNS=85).** (3-5 year follow-up)

found "a significant reduction of (major) osteoporotic fractures and hip fractures after screening using fracture risk assessment and bone densitometry compared with usual care." Reduction of osteoporotic fractures (HR = 0.95, 95% confidence interval (CI) = 0.89–1.00), Major osteoporotic fractures (HR = 0.91; 95%CI = 0.84–0.98), and hip fractures (HR = 0.80; 95%CI = 0.71–0.91), Numbers needed to screen to prevent one fracture were 247 and 272 for osteoporotic fractures and hip fractures, respectively (corresponding to 113 and 124 performed bone densitometry

[5] Females ≥65 years willing to independently complete a mailed fracture risk questionnaire

Appendix 10, as supplied by the authors. Appendix to: Thériault G, Limburg H, Klarenbach S, et al. Recommendations on screening for primary prevention of fragility fractures. *CMAJ* 2023. doi: 10.1503/cmaj.221219. Copyright © 2023 The Author(s) or their employer(s). To receive this resource in an accessible format, please contact us at cmajgroup@cmaj.ca.

- The evidence for acceptors of screening (females 45-54 years) is very uncertain.

Study approach	Population<br>Studies; Sample size	Anticipated absolute effects: Assumed population risk*	Anticipated absolute effects: Risk with screening (95% CI)	Anticipated absolute effects: Absolute difference (95% CI)	Relative HR (95% CI)	Certainty	Judgement
All eligible / offer-to-screen	Females<br>45-54 y;<br>1 RCT (APOSS) (23); 2,797<br>Follow-up: 9 years	Control event rate (study data)			1.01 (0.68 to 1.50)	Very Low[a-d]	Very uncertain
		34 per 1,000	34.3 per 1000 (23.1 to 51.0)	0.3 more per 1,000 (10.9 fewer to 17.0 more)			
		General population risk*					
		67 per 1,000	67.7 per 1000 (45.6 to 100.5)	0.7 more per 1,000 (21.4 fewer to 33.5 more)			
	Females<br>68-80 y;<br>1 RCT (ROSE) (24); 34,229<br>Follow-up: 5 years	Control event rate (study data)			0.99 (0.92 to 1.06)	Low[a-c]	**May not reduce**
		100 per 1,000	99.0 per 1000 (92.0 to 106.0)	1.0 fewer per 1,000 (8.0 fewer to 6.0 more)			
		General population risk*					
		168 per 1,000	166.3 per 1,000 (154.6 to 178.1)	1.7 fewer per 1,000 (13.4 fewer to 10.1 more)			

examinations, and 25 and 28 persons being treated).

Appendix 10, as supplied by the authors. Appendix to: Thériault G, Limburg H, Klarenbach S, et al. Recommendations on screening for primary prevention of fragility fractures. *CMAJ* 2023. doi: 10.1503/cmaj.221219. Copyright © 2023 The Author(s) or their employer(s). To receive this resource in an accessible format, please contact us at cmajgroup@cmaj.ca.

Acceptors of screening	Females<br><br>45-54 y;<br><br>1 RCT (APOSS) (23); 2,604<br><br>Follow-up: 9 years	Control event rate (study data)			0.73 (0.46 to 1.14)	Very Low[a-d]	Very uncertain
		34 per 1,000	24.8 per 1,000 (15.6 to 38.8)	9.2 fewer per 1,000 (18.4 fewer to 4.8 more)			
		General population risk*					
		67 per 1,000	48.9 per 1,000 (30.8 to 76.4)	18.1 fewer per 1,000 (36.2 fewer to 9.4 more)			
Offer-to-screen in <u>selected population[5]</u>	Females<br><br>≥65 y;<br><br>3 RCTs (SALT, SCOOP, ROSE) (24–26); 42,009<br><br>Follow-up: 3-5 years	Control event rate (study data)			0.93 (0.87 to 0.99)	Moderate[c]	**<u>Probably slightly reduces</u>**
		84 per 1000	78.1 per 1000 (73.1 to 83.2)	**5.9 fewer per 1000 (10.9 fewer to 0.8 fewer)**			
		General population risk*					
		168 per 1000	156.2 (146.2 to 166.3)	**11.8 fewer per 1000 (21.8 fewer to 1.7 fewer)**			

CCT: clinical controlled trial; CI: confidence interval; RCT: randomized controlled trial; y: years
a=risk of bias; b=inconsistency; c=indirectness; d=imprecision
*The effects without screening for the general risk population are estimated from Prior et al., 2015, based on 10 year follow-up (28)

Table 1.3: All cause-mortality

Appendix 10, as supplied by the authors. Appendix to: Thériault G, Limburg H, Klarenbach S, et al. Recommendations on screening for primary prevention of fragility fractures. *CMAJ* 2023. doi: 10.1503/cmaj.221219. Copyright © 2023 The Author(s) or their employer(s). To receive this resource in an accessible format, please contact us at cmajgroup@cmaj.ca.

- The evidence about all eligible / offer-to-screen (females 45-54 years) using the assumed population risk from the study data is very uncertain.
- **Screening <u>may not reduce</u> all-cause mortality in females 45-54 years (*all eligible / offer-to-screen*)** based on the assumed population risk found in the general population
- **Screening <u>probably does not reduce</u> all-cause mortality for *offer-to-screen in selected populations*[6]** (females ≥65 years) (3-5 year follow-up).

Study approach	Population<br>Studies; Sample size	Anticipated absolute effects			Relative HR (95% CI)	Certainty	Judgement
		Assumed population risk*	Risk with screening (95% CI)	Absolute difference (95% CI)			
All eligible / offer-to-screen	Females<br>45-54 y;<br>1 RCT (APOSS) (23); 4,800<br>Follow-up: 9 years	Control event rate (study data)			0.99 (0.72 to 1.35)	Very Low to Low[b,d]	Very uncertain
		33 per 1000	32.7 per 1,000 (23.8 to 44.6)	0.3 fewer per 1,000 (9.2 fewer to 11.6 more)			
		General population risk*				Low to Moderate[b,d]	**<u>May not reduce</u>**
		3 per 1,000	3.0 per 1,000 (2.2 to 4.1)	No difference per 1,000 (0.8 fewer to 1.1 more)			
	Females<br>68-80 y;	Control event rate			0.97 (0.92-1.03)	Low[b,d]	**<u>May not reduce</u>**
		118 per 1000	114.5 per 1000 (108.6 to 121.5)	3.5 fewer per 1000 (9.4 fewer to 3.5 more)			
		General population risk*					

[6] Females ≥65 years willing to independently complete a mailed fracture risk questionnaire

Appendix 10, as supplied by the authors. Appendix to: Thériault G, Limburg H, Klarenbach S, et al. Recommendations on screening for primary prevention of fragility fractures. *CMAJ* 2023. doi: 10.1503/cmaj.221219. Copyright © 2023 The Author(s) or their employer(s). To receive this resource in an accessible format, please contact us at cmajgroup@cmaj.ca.

	1 RCT (ROSE) (24); 34,299<br><br>Follow-up: 5 years	57 per 1000	55.3 per 1000 (52.4 to 58.7)	1.7 fewer per 1000 (4.6 fewer to 1.7 more)			
Offer-to-screen in selected population[7]	Females ≥65 y;<br><br>2 RCTs + 1 CCT (SALT, SCOOP + Kern) (24–27); 59,740<br><br>Follow-up: 3-5 years	Control event rate (study data)			1.00 (0.92 to 1.09)	Moderate[d]	**<u>Probably does not reduce</u>**
		89 per 1000	89.0 per 1000 (81.9 to 94.3)	No difference in 1000 (7.1 fewer to 5.3 more)			
		General population risk*					
		57 per 1000	57.0 per 1000 (52.4 to 62.1)	No difference in 1000 (4.6 fewer to 5.1 more)			

CCT: clinical controlled trial; CI: confidence interval; RCT: randomized controlled trial; y: years
a=risk of bias; b=inconsistency; c=indirectness; d=imprecision
*The effects without screening for the general risk population are estimated from Prior et al., 2015, based on 10 year follow-up (28).

Table 1.4: Quality of life or wellbeing

- The evidence about all eligible / offer-to-screen females 45-54 years is very uncertain.
- **Screening <u>may make little to no difference</u> on self-rated health or health related quality of life for *offer-to-screen in selected populations*[7] (females 70-85 y) (3-5 year follow-up).**

[7] Females ≥65 years willing to independently complete a mailed fracture risk questionnaire

Appendix 10, as supplied by the authors. Appendix to: Thériault G, Limburg H, Klarenbach S, et al. Recommendations on screening for primary prevention of fragility fractures. *CMAJ* 2023. doi: 10.1503/cmaj.221219. Copyright © 2023 The Author(s) or their employer(s). To receive this resource in an accessible format, please contact us at cmajgroup@cmaj.ca.

Outcome	Study approach	Population<br><br>Studies; Sample size	Findings	Certainty	Judgement
**Wellbeing – self-rated health**	All eligible / offer-to-screen	Females 45-54 y;<br><br>1 RCT (APOSS) (23); 2,797<br><br>Follow-up: 9 years	Good or very good: 69.2% of screened, 68.0% controls<br><br>Not so good or poor: 12.8% of screened, 14.3% controls	Very Low to Low[a-c]	Very uncertain
	Offer-to-screen in selected population[7]	Females 70-85 y;<br><br>1 RCT (SCOOP) (26); 10,661<br><br>Follow-up: 3 years	Mental health: MD -0.30, 95% CI -0.86 to 0.26<br><br>Physical health: MD 0.30, 95% CI -0.21 to 0.81	Low to Moderate[a-c]	**May be little to no difference**
**Wellbeing – health-related quality of life**	All eligible / offer-to-screen	Females 45-54 y;<br><br>1 RCT (APOSS) (23); 1,217<br><br>Follow-up: 2 years	General health perception, mean (SD): 69.7 (21.7) in screened, 69.8 (20.8) in controls	Very Low to Low[a,b]	Very uncertain

Appendix 10, as supplied by the authors. Appendix to: Thériault G, Limburg H, Klarenbach S, et al. Recommendations on screening for primary prevention of fragility fractures. *CMAJ* 2023. doi: 10.1503/cmaj.221219. Copyright © 2023 The Author(s) or their employer(s). To receive this resource in an accessible format, please contact us at cmajgroup@cmaj.ca.

	Offer-to-screen in selected population[7]	Females 70-85 y;<br><br>1 RCT (SCOOP) (26); 10,661<br><br>Follow-up: 3 years	MD 0, 95% CI -0.07 to 0.07	Low to Moderate[a, b]	**May be little to no difference**

CCT: clinical controlled trial; CI: confidence interval; RCT: randomized controlled trial; MD: mean difference; y: years
a=risk of bias; b=inconsistency; c=indirectness; d=imprecision

KQ1b: Does the effectiveness of screening to prevent fragility fractures vary by screening program type (i.e., BMD-first vs Risk assessment-first) or risk assessment tool (e.g., FRAX vs CAROC)?

Appendix 10, as supplied by the authors. Appendix to: Thériault G, Limburg H, Klarenbach S, et al. Recommendations on screening for primary prevention of fragility fractures. *CMAJ* 2023. doi: 10.1503/cmaj.221219. Copyright © 2023 The Author(s) or their employer(s). To receive this resource in an accessible format, please contact us at cmajgroup@cmaj.ca.

SUMMARY: JUDGEMENT OF BENEFITS (*COMPARING SCREENING STRATEGIES*)

There was no evidence comparing the most common risk assessment tools used in Canada (i.e. FRAX and CAROC).

Evidence for SOF or SCORE tools + BMD (risk assessment-first) vs BMD alone (BMD-first) was very uncertain

EVIDENCE TABLES

Table 2.1: Hip and clinical fragility fracture outcomes by screening program type:

Outcome	Study approach	Population<br><br>Studies; Sample size	Anticipated absolute effects* (95% CI)			Relative HR (95% CI)	Certainty	Judgement
			Assumed population risk	Risk with BMD-first	Absolute difference			
**BMD-first vs. Risk assessment-first (SOF- or SCORE-based tool + BMD) screening**								
**Hip fractures**	All eligible / offer-to-screen	Females 60-80 y;	12 per 1,000	8.4 per 1,000 (5.0 to 14.1)	3.6 fewer per 1,000 (7.0 fewer to 2.1 more)	0.70 (0.42 to 1.18)	Very Low[a,b,d]	Very uncertain

Appendix 10, as supplied by the authors. Appendix to: Thériault G, Limburg H, Klarenbach S, et al. Recommendations on screening for primary prevention of fragility fractures. *CMAJ* 2023. doi: 10.1503/cmaj.221219. Copyright © 2023 The Author(s) or their employer(s). To receive this resource in an accessible format, please contact us at cmajgroup@cmaj.ca.

Follow-up: mean 2.3 years		1 RCT (OPRA) (29); 9,268						
	Acceptors of screening	Females 60-80 y; 1 RCT (OPRA) (29); 3,167	9 per 1,000	3.4 per 1,000 (0.6 to 19.3)	5.6 fewer per 1,000 (8.4 fewer to 10,3 more)	0.38 (0.07 to 2.14)	Very Low[,b,d]	Very uncertain
**Clinical fragility fractures**<br><br>Follow-up: mean 2.3 years	All eligible / offer-to-screen	Females 60-80 y; 1 RCT (OPRA) (29); 9,268	96 per 1,000	75.8 per 1,000 (63.4 to 90.2)	20.2 fewer per 1,000 (32.6 fewer to 5.8 fewer)	0.79 (0.66 to 0.94)	Very Low[a-c]	Very uncertain
	Acceptors of screening	Females 60-80 y; 1 RCT (OPRA) (29); 3,167	98 per 1,000	89.1 per 1,000 (63.7 to 126.4)	8.9 fewer per 1,000 (34.3 fewer to 28.4 more)	0.91 (0.65 to 1.29)	Very Low[a-d]	Very uncertain
**BMD-first vs. Risk assessment-first (SCORE-based tool + BMD) screening**								
**Hip fractures**	All eligible / offer-to-screen	Females 60-80 y;	9 per 1,000	8.5 per 1,000 (4.3 to 16.6)	0.5 fewer per 1,000	0.94 (0.48 to 1.84)	Very Low[a,b,d]	Very uncertain

Appendix 10, as supplied by the authors. Appendix to: Thériault G, Limburg H, Klarenbach S, et al. Recommendations on screening for primary prevention of fragility fractures. *CMAJ* 2023. doi: 10.1503/cmaj.221219. Copyright © 2023 The Author(s) or their employer(s). To receive this resource in an accessible format, please contact us at cmajgroup@cmaj.ca.

Follow-up: mean 2.3 years		1 RCT (OPRA) (29); 3,926			(4.7 fewer to 7.6 more)			
	Acceptors of screening	Females 60-80 y; 1 RCT (OPRA) (29); 991	8 per 1,000	3.2 per 1000 (0.5 to 22.2)	4.8 fewer per 1,000 (7.5 fewer to 14.2 more)	0.40 (0.06 to 2.78)	Very Low[a-d]	Very uncertain
**Clinical fragility fractures** Follow-up: mean 2.3 years	All eligible / offer-to-screen	Females 60-80 y; 1 RCT (OPRA) (29); 3,926	99 per 1,000	74.3 per 1,000 (59.4 to 91.1)	24.7 fewer per 1,000 (39.6 fewer to 7.9 fewer)	0.75 (0.60 to 0.92)	Low[a-c]	Very uncertain
	Acceptors of screening	Females 60-80 y; 1 RCT (OPRA) (29); 3,167	116 per 1,000	89.3 per 1000 (59.2 to 133.4)	26.7 fewer per 1,000 (56.8 fewer to 17.4 more)	0.77 (0.51 to 1.15)	Very Low[a-d]	Very uncertain
**BMD-first vs. Risk assessment-first (SOF-based tool + BMD) screening**								
**Hip fractures**	All eligible / offer-to-screen	Females 60-80 y;	13 per 1,000	8.3 per 1000 (4.9 to 14.2)	4.7 fewer per 1,000	0.64 (0.38 to 1.09)	Very Low[a,b,d]	Very uncertain

Appendix 10, as supplied by the authors. Appendix to: Thériault G, Limburg H, Klarenbach S, et al. Recommendations on screening for primary prevention of fragility fractures. *CMAJ* 2023. doi: 10.1503/cmaj.221219. Copyright © 2023 The Author(s) or their employer(s). To receive this resource in an accessible format, please contact us at cmajgroup@cmaj.ca.

Follow-up: mean 2.3 years		1 RCT (OPRA) (29); 7,328			(8.1 fewer to 1.2 more)			
	Acceptors of screening	Females 60-80 y; 1 RCT (OPRA) (29); 2,591	9 per 1,000	3.3 per 1000 (0.5 to 19.1)	5.7 fewer per 1,000 (8.5 fewer to 10.1 more)	0.37 (0.06 to 2.12)	Very Low[a,b,d]	Very uncertain
**Clinical fragility fractures** Follow-up: mean 2.3 years	All eligible / offer-to-screen	Females 60-80 y; 1 RCT (OPRA) (29); 7,328	92 per 1,000	74.5 per 1000 (61.6 to 89.2)	17.5 fewer in 1,000 (30.4 fewer to 2.8 fewer)	0.81 (0.67 to 0.97)	Very Low[a-c]	Very uncertain
	Acceptors of screening	Females 60-80 y; 1 RCT (OPRA) (29); 2,591	93 per 1,000	89.2 per 1000 (63.2 to 127.4)	3.8 fewer per 1,000 (29.8 fewer to 34.4 more)	0.96 (0.68 to 1.37)	Very Low[a-d]	Very uncertain
**Risk assessment-first (SCORE-based tool + BMD) vs. another risk assessment-first (SOF-based tool + BMD) screening**								
**Hip fractures**	All eligible / offer-to-screen	Females 60-80 y;	13 per 1,000	8.8 per 1000 (5.2 per 1000	4.2 fewer per 1,000	0.68 (0.40 to 1.15)	Very Low[a,b,d]	Very uncertain

Appendix 10, as supplied by the authors. Appendix to: Thériault G, Limburg H, Klarenbach S, et al. Recommendations on screening for primary prevention of fragility fractures. *CMAJ* 2023. doi: 10.1503/cmaj.221219. Copyright © 2023 The Author(s) or their employer(s). To receive this resource in an accessible format, please contact us at cmajgroup@cmaj.ca.

Follow-up: mean 2.3 years		1 RCT (OPRA) (29); 7,282		to 15.0 per 1000)	(7.8 fewer to 2.0 more)			
	Acceptors of screening	Females 60-80 y; 1 RCT (OPRA) (29); 2,752	9 per 1,000	8.2 per 1000 (3.0 to 22.7)	0.8 fewer per 1000 (6.0 fewer to 13.7 more)	0.91 (0.33 to 2.52)	Very Low[a,b,d]	Very uncertain
**Clinical fragility fractures** Follow-up: mean 2.3 years	All eligible / offer-to-screen	Females 60-80 y; 1 RCT (OPRA) (29); 7,282	92 per 1,000	99.4 per 1000 (84.6 to 117.8)	7.4 more per 1000 (7.4 fewer to 25.8 more)	1.08 (0.92 to 1.28)	Very Low[a-d]	Very uncertain
	Acceptors of screening	Females 60-80 y; 1 RCT (OPRA) (29); 2,752	93 per 1,000	116.3 per 1000 (88.4 to 153.5)	23.3 more per 1000 (4.6 fewer to 60.5 more)	1.25 (0.95 to 1.65)	Very Low[a-d]	Very uncertain

BMD: bone mineral density; CCT: clinical controlled trial; CI: confidence interval; RCT: randomized controlled trial; SCORE: Simple calculated osteoporosis risk estimation; SOF: Study of osteoporotic fractures (calculation); y: years
a=risk of bias; b=inconsistency; c=indirectness; d=imprecision
* The absolute effect (and its 95% CI) with risk assessment-first screening (i.e. baseline rate) is based on the estimated risk in the risk assessment-first screening group; the effect with BMD-first screening is based on applying the relative effect of the intervention (and its 95% CI) to the effect in the BMD-first group.

Appendix 10, as supplied by the authors. Appendix to: Thériault G, Limburg H, Klarenbach S, et al. Recommendations on screening for primary prevention of fragility fractures. *CMAJ* 2023. doi: 10.1503/cmaj.221219. Copyright © 2023 The Author(s) or their employer(s). To receive this resource in an accessible format, please contact us at cmajgroup@cmaj.ca.

		**KQ2: How accurate are screening tests at predicting fractures among adults ≥ 40 years?**	

Appendix 10, as supplied by the authors. Appendix to: Thériault G, Limburg H, Klarenbach S, et al. Recommendations on screening for primary prevention of fragility fractures. *CMAJ* 2023. doi: 10.1503/cmaj.221219. Copyright © 2023 The Author(s) or their employer(s). To receive this resource in an accessible format, please contact us at cmajgroup@cmaj.ca.

JUDGEMENT – ACCURACY OF RISK ASSESSMENT TOOLS

FRAX without BMD (lower risk of bias studies, calibrated for Canada)
- May be well-calibrated to predict 10-year hip fractures.
- Is probably well-calibrated to predict 10-year clinical fragility fractures
- May be poorly calibrated to predict 5-year hip fractures
- May be well-calibrated to predict 5-year clinical fragility fractures (most applicable to females)

FRAX with BMD (lower risk of bias studies, calibrated for Canada)
- May perform poorly to predict 10-year hip fractures.
- Is probably well calibrated to predict 10-year clinical fragility fractures
- May be well calibrated to predict 5-year hip or clinical fragility fractures (most applicable to females)

CAROC may be adequately calibrated to predict category of clinical fragility fracture risk.

Evidence for Garvan +/- BMD to predict the 10-year risk of hip or clinical fragility fractures is very uncertain. Garvan alone may underestimate the 5-year risk of hip fractures.

Evidence on QFracture is very uncertain for 10-year risk of hip and clinical fragility fracture. QFracture may underestimate 5-year hip fracture risk.

Evidence on FRISC, FRC and FRC+BMD is very uncertain.

Appendix 10, as supplied by the authors. Appendix to: Thériault G, Limburg H, Klarenbach S, et al. Recommendations on screening for primary prevention of fragility fractures. *CMAJ* 2023. doi: 10.1503/cmaj.221219. Copyright © 2023 The Author(s) or their employer(s). To receive this resource in an accessible format, please contact us at cmajgroup@cmaj.ca.

EVIDENCE TABLES

Table 3.1 Calibration of FRAX

FRAX without BMD (high risk of bias studies)

- Evidence from high risk of bias studies (not calibrated for Canada) was very uncertain for the conclusion of poor performance to predict 10-year hip fracture
- Evidence from high risk of bias studies (n=1/12 calibrated for Canada) was very uncertain for the conclusion of poor performance to predict 10-year clinical fragility fracture
- Evidence from high risk of bias studies (not calibrated for Canada) was very uncertain for the conclusion of poor performance to predict 5-year[8] hip fracture

FRAX without BMD (lower risk of bias studies)

- FRAX without BMD (lower risk of bias studies, calibrated for Canada) may be well-calibrated to predict 10-year hip fracture.
- FRAX without BMD (lower risk of bias studies, calibrated for Canada) is probably well-calibrated to predict 10-year clinical fragility fracture.
- FRAX without BMD (lower risk of bias studies, calibrated for Canada) may be poorly-calibrated to predict 5-year hip fracture[8].
- FRAX without BMD (lower risk of bias studies, calibrated for Canada) may be well-calibrated to predict 5-year clinical fragility fracture (females only)[8].

FRAX with BMD (high risk of bias studies)

- Evidence from high risk of bias studies (not calibrated for Canada) was very uncertain for the conclusion of poor performance to predict 10-year hip for clinical fragility fracture

FRAX with BMD (lower risk of bias studies)

- FRAX with BMD (lower risk of bias studies, calibrated for Canada) may perform poorly to predict 10-year hip fracture.
- FRAX without BMD (lower risk of bias studies, calibrated for Canada) is probably well-calibrated to predict 10-year clinical fragility fracture.
- FRAX without BMD (lower risk of bias studies, calibrated for Canada) may be well-calibrated to predict 5-year hip (females)[8].
- FRAX without BMD (lower risk of bias studies, calibrated for Canada) may be well-calibrated to predict clinical fragility fracture (females only)[8].

[8] FRAX is intended for 10-year hip or major osteoporotic fracture only.

Appendix 10, as supplied by the authors. Appendix to: Thériault G, Limburg H, Klarenbach S, et al. Recommendations on screening for primary prevention of fragility fractures. *CMAJ* 2023. doi: 10.1503/cmaj.221219. Copyright © 2023 The Author(s) or their employer(s). To receive this resource in an accessible format, please contact us at cmajgroup@cmaj.ca.

Outcome[*] <br>Studies; sample size	Findings	Certainty[†]	What does the evidence say?	Discrimination[‡] (pooled AUC, 95% CI) (5)
**FRAX without BMD (high risk of bias studies)**				
10-y hip fractures <br>13 cohort; 343,755 <br>(30–42)	None of the FRAX tools in this analysis were calibrated for Canada. Most studies show poor calibration and are inconsistent. Most often, the tool over- (n=4 studies, 4 comparisons; O:E estimates from 0.26 to 0.72) or underestimated (n=5 studies, 7 comparisons; O:E 1.21 to 3.87) the observed fracture risk. Inconsistency was not well explained by subgroup analyses.	Very Low[a-d]	Very uncertain for the conclusion of poor performance.	All studies, regardless of risk of bias: <br>F: 0.76 (0.72-0.81) <br>M: 0.73 (0.68-0.77)
10-y clinical fragility fractures <br>12 cohort; 190,116 <br>(30,31,41,43–46,32–34,36–40)	Only one of the 12 studies used the FRAX tool calibrated for Canada. Most studies show poor calibration and are inconsistent. Most often, the tool underestimated (n=7 studies, 8 comparisons; O:E 1.33 to 3.34) the observed fracture risk. Inconsistency was not well explained by subgroup analyses.	Very Low[a-d]	Very uncertain for the conclusion of poor performance.	All studies, regardless of risk of bias: <br>F: 0.67 (0.65-0.68) <br>M: 0.62 (0.61-0.64)
5-y hip fractures[§] <br>1 cohort; 1,054,815 (47)	A single study that did not use a FRAX tool calibrated to Canada showed underestimation of the observed 5-year risk of hip fracture (O:E 1.74, 95% CI 1.72-1.76).	Very Low[a,b,c]	Very uncertain for the conclusion of poor performance.	NR
5-y clinical fragility fractures	A single study of a FRAX tool calibrated to Canada showed overestimation of the observed 5-year risk of clinical fragility	Very Low[d]	Very uncertain for the conclusion of poor performance.	NR

Appendix 10, as supplied by the authors. Appendix to: Thériault G, Limburg H, Klarenbach S, et al. Recommendations on screening for primary prevention of fragility fractures. *CMAJ* 2023. doi: 10.1503/cmaj.221219. Copyright © 2023 The Author(s) or their employer(s). To receive this resource in an accessible format, please contact us at cmajgroup@cmaj.ca.

1 cohort; 9,393 (48)	fracture (O:E 0.75, 95% CI 0.68-0.89)				
**FRAX without BMD (lower risk of bias studies)**					
10-y hip fractures<br><br>3 cohort; 67,611 (43,44,46)	All studies used the FRAX tool calibrated for Canada. The pooled O:E showed acceptable calibration with some underestimation of the observed fracture risk, and a wide confidence interval **(pooled O:E 1.13, 95% CI 0.74-1.72, I^2=89.2%).**	Low[b,d]	**May be well calibrated.**	See above.	
10-y clinical fragility fractures<br><br>3 cohort; 67,611 (43,44,46)	All studies used the FRAX tool calibrated for Canada. The pooled O:E showed acceptable calibration with some underestimation of the observed fracture risk **(O:E 1.10, 95% CI 1.01-1.20, I^2=50.4%).**	Moderate[b,d]	**Probably well calibrated.**	See above.	There was a discordance between the effect size seen in the screening (e.g. 6.2 fewer per 1,000 (hip fractures) and treatment trials (e.g. 2.9-5.3 fewer in 1,000 hip fractures). This may be due to older population in screening trials.<br><br>See Balance of Effects section for comments on differences in population risk for hip and clinical fragility fracture compared to the Canadian general population.
5-y hip fractures[8]<br><br>1 cohort; 68,730 (62,275 F, 6,445 M)<br><br>(44)	A single study, which used the FRAX tool calibrated for Canada, showed large overestimation of the observed 5-year risk of hip fracture in females **(O:E 0.68, 95% CI 0.62-0.73)** and imprecise underestimation in males **(O:E 0.82, 95% CI 0.60- 1.03).**	Low[a,b,d]	**May be poorly calibrated.**	NR	
5-y clinical fragility fractures[8]<br><br>1 cohort; 68,730 (62,275 F, 6,445 M) (44)	A single study, which used the FRAX tool calibrated for Canada, found acceptable calibration in females **(O:E 0.93, 95% CI 0.89-0.96)**. The tool imprecisely underestimated the observed fracture risk in males **(O:E 1.23, 95% CI 1.08-1.38).**	Low[a,b,d]	**May be well calibrated (most applicable to females).**	NR	
**FRAX + BMD (high risk of bias studies)**					

Appendix 10, as supplied by the authors. Appendix to: Thériault G, Limburg H, Klarenbach S, et al. Recommendations on screening for primary prevention of fragility fractures. *CMAJ* 2023. doi: 10.1503/cmaj.221219. Copyright © 2023 The Author(s) or their employer(s). To receive this resource in an accessible format, please contact us at cmajgroup@cmaj.ca.

10-y hip fractures<br><br>13 cohort; 138,606 (30,31,42,49,50, 32,33,36–41)	None of the FRAX tools in this analysis were calibrated for Canada. Most studies show poor calibration and are inconsistent. Most often, the tool either over- (n = 4 studies, 6 comparisons; O:E range from 0.24 to 0.68) or underestimated (n = 8 studies, 10 comparisons; O:E 1.30 to 3.33) the observed fracture risk. Inconsistency was not well explained by subgroup analyses.	Very Low[a,c,d]	Very uncertain for the conclusion of poor performance.	All studies, regardless of risk of bias:<br><br>F: 0.79 (0.76-0.81)<br><br>M: 0.76 (0.72-0.80)
10-y clinical fragility fractures<br><br>16 cohort; 49,235 (30,31,49–54,32,33,36–41)	None of the FRAX tools in this analysis were calibrated for Canada. Most studies show poor calibration and are inconsistent. Most often (10 studies, 12 comparisons; O:E 1.11 to 3.90), the tool underestimated the observed fracture risk. Inconsistency was not well explained by subgroup analyses.	Very Low[a-d]	Very uncertain for the conclusion of poor performance.	All studies, regardless of risk of bias:<br><br>F: 0.70 (0.68-0.71)<br><br>M: 0.67 (0.66-0.68)
**FRAX + BMD (lower risk of bias studies)**				
10-y hip fractures<br><br>3 cohort; 61,156<br><br>(43,44,46)	All studies used the FRAX tool calibrated for Canada. The pooled O:E showed underestimation of the observed risk with a high level of inconsistency (O:E 1.31, 95% CI 0.91-2.13, I^2 = 92.7%); two comparisons showed acceptable calibration while two others showed substantial underestimation of the observed fracture risk.	Low[b,d]	**May perform poorly.**	See above.
10-y clinical fragility fractures<br><br>3 cohort; 61,156 (43,44,46)	All studies used the FRAX tool calibrated for Canada. The pooled O:E showed acceptable calibration with some underestimation of the observed risk **(O:E 1.16, 95% CI 1.12-1.20, I^2 = 0%).**	Moderate[b,d]	**Probably well calibrated.**	See above.

Appendix 10, as supplied by the authors. Appendix to: Thériault G, Limburg H, Klarenbach S, et al. Recommendations on screening for primary prevention of fragility fractures. *CMAJ* 2023. doi: 10.1503/cmaj.221219. Copyright © 2023 The Author(s) or their employer(s). To receive this resource in an accessible format, please contact us at cmajgroup@cmaj.ca.

5-y hip fractures[8]<br><br>1 cohort; 68,730 (62,275 F, 6,445 M) (44)	A single study, which used the FRAX tool calibrated for Canada, showed acceptable calibration with some overestimation in females **(O:E 0.88, 95% CI 0.81-0.95)** and males **(O:E 0.88, 95% CI 0.65-1.10).**	Low[,b,d]	**May be well calibrated (most applicable to females.**	NR
5-y clinical fragility fractures[8]<br><br>1 cohort; 68,730 (62,275 F, 6,445 M) (44)	A single, which used the FRAX tool calibrated for Canada, study provided inconsistent findings, showing acceptable calibration in females **(O:E 1.00, 95% CI 0.97-1.04).** The tool imprecisely underestimated the observed fracture risk in males **(O:E 1.22, 95% CI 1.07, 1.37).**	Low[a,b,d]	**May be well calibrated (most applicable to females).**	NR

BMD=bone mineral density; CI=confidence interval; F=female; M=male; O:E ratio=ratio of observed to expected (predicted) events

a=risk of bias; b=inconsistency; c=indirectness; d=imprecision

*Rows for 5-year fractures have been omitted from the table when no studies were located that reported on this outcome.

† When our assessment of the certainty of evidence fell between levels, we assigned the level that best represented our actual certainty.

‡Extracted directly from the 2018 USPSTF systematic review by Viswanathan et al. (5).

Table 3.2 Calibration of CAROC

- CAROC: may be adequately calibrated to predict category of clinical fragility fracture risk (one study only)

**CAROC (includes BMD)**				
10-y hip fractures	No studies reported this outcome.	Not applicable		NR
10-y clinical fragility fractures<br><br>1 cohort; 34,060 (55)	One study did not report an O:E ratio. Observed fracture risk (95% CI) was 6.4 (6.0-6.8)% in the low risk (<10%) group, 13.8 (13.1-14.5)% in the moderate risk group (10-20%), and 23.8 (22.5-25.0)% in the high risk group (>20%).	Low[a-c]	**May be adequately calibrated to predict a category of risk.**	NR

Appendix 10, as supplied by the authors. Appendix to: Thériault G, Limburg H, Klarenbach S, et al. Recommendations on screening for primary prevention of fragility fractures. *CMAJ* 2023. doi: 10.1503/cmaj.221219. Copyright © 2023 The Author(s) or their employer(s). To receive this resource in an accessible format, please contact us at cmajgroup@cmaj.ca.

BMD=bone mineral density; CI=confidence interval; F=female; M=male; O:E ratio=ratio of observed to expected (predicted) events a=risk of bias; b=inconsistency; c=indirectness; d=imprecision

*Rows for 5-year fractures have been omitted from the table when no studies were located that reported on this outcome.

† When our assessment of the certainty of evidence fell between levels, we assigned the level that best represented our actual certainty.

‡Extracted directly from the 2018 USPSTF systematic review by Viswanathan et al. (5).

Table 3.3 Calibration of Garvan, QFracture, FRISC and FRC

- Evidence on using Garvan +/- BMD to predict the 10-year risk of hip or clinical fragility fractures is very uncertain.
- Garvan alone may underestimate the 5-year risk of hip fractures.
- Evidence on QFracture is very uncertain for 10-year risk of hip and clinical fragility fracture. QFracture may underestimate 5-year hip fracture risk.
- Evidence on FRISC (Fracture and Immobilization Score (includes BMD)), FRC (Fracture Risk Calculator) and FRC+BMD is very uncertain.

**Garvan alone (no BMD)**				
10-y hip fractures<br><br>2 cohort; 67,923 (46,56)	In one study, the tool substantially underestimated the observed fracture risk (O:E 3.63, 95% CI 3.31-3.97). A second study reported only the Hosmer-Lemeshow test (p<0.0001), indicating poor calibration.	Very Low[a-c]	Very uncertain for the conclusion of poor performance	F: 0.68 (NR)<br><br>F: 0.65 (NR)
10-y clinical fragility fractures 1 cohort; 5,063 (56)	In one study, the Hosmer-Lemeshow test was significant (p=0.01014), indicating poor calibration.	Very Low[a,b]	Very uncertain for the conclusion of poor performance	F: 0.66 (0.61-0.72)<br><br>M: NR
5-y hip fractures<br><br>1 cohort; 1,054,815 (47)	In one study, the tool substantially underestimated the observed fracture risk (O:E 2.17, 95% CI 2.16-2.17).	Low[a-c]	**May underestimate by 116 to 117%**	NR

Appendix 10, as supplied by the authors. Appendix to: Thériault G, Limburg H, Klarenbach S, et al. Recommendations on screening for primary prevention of fragility fractures. *CMAJ* 2023. doi: 10.1503/cmaj.221219. Copyright © 2023 The Author(s) or their employer(s). To receive this resource in an accessible format, please contact us at cmajgroup@cmaj.ca.

**Garvan + BMD**				
10-y hip fractures<br><br>5 cohort; 11,869<br><br>(31,40,56–58)	Most studies show poor calibration and are inconsistent. Most often, the tool overestimated fracture risk to an important magnitude, though the degree of overestimation is highly variable (n = 3 studies, 4 comparisons, O:E 0.10 to 0.66).<br><br>Inconsistency was not well explained by subgroup analyses.	Very Low[a,b,d]	Very uncertain for the conclusion of poor performance	F: 0.73 (0.66-0.79)<br><br>M: 0.79 (NR)
10-y clinical fragility fractures<br><br>5 cohort; 11,733 (31,40,56–58)	Most studies show poor calibration and are inconsistent. Most often, the tool over- (n = 2 studies, 2 comparisons; O:E 0.34 to 0.74) or underestimated (n = 1 study, 1 comparison; O:E 1.65) the observed fracture risk. One study reported only the Hosmer-Lemeshow test (p=0.0001), indicating poor calibration.<br><br>Inconsistency was not well explained by subgroup analyses.	Very Low[a,b,d]	Very uncertain for the conclusion of poor performance	F: 0.68 (0.64-0.71)<br><br>M: 0.75 (NR)
**QFracture (no BMD)**				
10-y hip fractures<br><br>1 cohort; 5,200 (56)	In one study, the O:E was not reported. The Hosmer-Lemeshow test was significant (p<0.0001), indicating poor calibration.	Very Low[a,b,d]	Very uncertain for the conclusion of poor performance	NR
10-y clinical fragility fractures<br><br>1 cohort; 5,063 (56)	In one study, the O:E was not reported. The Hosmer-Lemeshow test was significant (p=0.0001), indicating poor calibration	Very Low[a,b,d]	Very uncertain for the conclusion of poor performance	NR

Appendix 10, as supplied by the authors. Appendix to: Thériault G, Limburg H, Klarenbach S, et al. Recommendations on screening for primary prevention of fragility fractures. *CMAJ* 2023. doi: 10.1503/cmaj.221219. Copyright © 2023 The Author(s) or their employer(s). To receive this resource in an accessible format, please contact us at cmajgroup@cmaj.ca.

5-y hip fractures<br><br>1 cohort; 1,054,815 (47)	In one study, the tool underestimated the observed fracture risk (O:E 1.42, 95% CI 1.41-1.42).	Low[a-c]	**May underestimate by 40 to 42%**	NR
**Fracture and Immobilization Score (FRISC; includes BMD)**				
10-y hip fractures	No studies reported this outcome.	Not applicable		NR
10-y clinical fragility fractures 1 cohort; 400 (52)	In one study, FRISC was imprecise for overestimation of the 10- year risk of clinical fragility fracture (O:E 0.74, 95% CI 0.59-0.93)	Very Low[a,b,d]	Very uncertain for the conclusion of poor performance	F: 0.73 (NR)
**Fracture Risk Calculator (FRC) alone (no BMD)**				
10-y hip fractures 2 cohort; 100,382<br><br>(59,60)	The evidence from 2 cohort studies (n=100,382) is very uncertain.	Very Low[a-d]	Very uncertain for the conclusion of poor performance	F: 0.83 (0.82-0.84)<br><br>M: 0.71 (NR)
10-y clinical fragility fractures 1 cohort; 5,893 (59)	The evidence from 1 cohort study (n=5,893) is very uncertain.	Very Low[a-d]	Very uncertain for the conclusion of poor performance	F: NR<br><br>M: 0.66 (NR)
**FRC + BMD**				
10-y hip fractures 2 cohort; 100,382<br><br>(59,60)	The evidence from 2 cohort studies (n=100,382) is very uncertain.	Very Low[a-d]	Very uncertain for the conclusion of poor performance	F: 0.85 (0.84-0.86)<br><br>M: 0.79 (NR)

Appendix 10, as supplied by the authors. Appendix to: Thériault G, Limburg H, Klarenbach S, et al. Recommendations on screening for primary prevention of fragility fractures. *CMAJ* 2023. doi: 10.1503/cmaj.221219. Copyright © 2023 The Author(s) or their employer(s). To receive this resource in an accessible format, please contact us at cmajgroup@cmaj.ca.

10-y clinical fragility fractures 1 cohort; 5,893 (59)	The evidence from 1 cohort study (n=5,893) is very uncertain.	Very Low[a-d]	Very uncertain for the conclusion of poor performance	F: NR<br><br>M: 0.70 (NR)

BMD=bone mineral density; CI=confidence interval; F=female; M=male; O:E ratio=ratio of observed to expected (predicted) events

a=risk of bias; b=inconsistency; c=indirectness; d=imprecision

[*]Rows for 5-year fractures have been omitted from the table when no studies were located that reported on this outcome.

[†] When our assessment of the certainty of evidence fell between levels, we assigned the level that best represented our actual certainty.

[‡]Extracted directly from the 2018 USPSTF systematic review by Viswanathan et al. (5).

KQ3a. What are the benefits of pharmacologic treatments to prevent fragility fractures among adults ≥40 years?

Appendix 10, as supplied by the authors. Appendix to: Thériault G, Limburg H, Klarenbach S, et al. Recommendations on screening for primary prevention of fragility fractures. *CMAJ* 2023. doi: 10.1503/cmaj.221219. Copyright © 2023 The Author(s) or their employer(s). To receive this resource in an accessible format, please contact us at cmajgroup@cmaj.ca.

JUDGEMENT – BENEFITS OF TREATMENT

Bisphosphonates (as a class) and risedronate may reduce hip fractures in post-menopausal females.

Bisphosphonates (as a class), alendronate, zoledronic acid and denosumab probably reduce clinical fragility fractures in post-menopausal females. Risedronate may reduce clinical fragility fractures in post-menopausal females.

Bisphosphonates (as a class) and zoledronic acid may reduce clinical vertebral fractures in post-menopausal females.

Bisphosphonates as a class may not reduce the risk of all-cause mortality compared to placebo over 1 to 6 years of follow-up (post-menopausal females). Evidence for individual bisphosphonates is very uncertain for all-cause mortality.

Evidence for males (zoledronic acid or denosumab) is very uncertain.

EVIDENCE TABLES

Table 4.1 Benefits of treatment

Bisphosphonates (as a class, post-menopausal females)

Appendix 10, as supplied by the authors. Appendix to: Thériault G, Limburg H, Klarenbach S, et al. Recommendations on screening for primary prevention of fragility fractures. *CMAJ* 2023. doi: 10.1503/cmaj.221219. Copyright © 2023 The Author(s) or their employer(s). To receive this resource in an accessible format, please contact us at cmajgroup@cmaj.ca.

		- Treatment with **bisphosphonates** as a class **may reduce the risk of hip fractures** (2.9-5.3 fewer in 1,000), **probably reduces the risk of clinical fragility fractures** (11.1-33.6 fewer in 1,000) and it **may reduce clinical vertebral fractures** (10.0-12.8 fewer in 1,000). Treatment with bisphosphonates may not reduce all-cause mortality.<br><br>Alendronate (post-menopausal females)<br>- Alendronate may not reduce hip fractures.<br>- **Alendronate probably reduces clinical fragility fractures** (14.7-28.4 fewer in 1,000). Evidence for clinical vertebral fractures and all-cause mortality was uncertain.<br><br>Risedronate (post-menopausal females)<br>- **Risedronate may reduce hip fractures** (5.3-7.9 fewer in 1,000) **and clinical fragility fractures** (7.8-28.4 fewer in 1000). Evidence for clinical vertebral fractures and all-cause mortality was uncertain.<br><br>Zoledronic acid (post-menopausal females)<br>- Zoledronic acid may not reduce hip fractures.<br>- **Zoledronic acid probably reduced clinical vertebral fractures** (20.1-62.6 fewer in 1,000) and **may reduce clinical vertebral fractures** (14.9-18.7 fewer in 1000). Evidence for all-cause mortality was uncertain.<br><br>Zoledronic acid (men)<br>- Zoledronic acid may not reduce hip fractures and clinical fragility fractures.<br>- The evidence for all-cause mortality was very uncertain<br><br>Denosumab (post-menopausal females)<br>- Denosumab may not reduce hip fractures<br>- **Denosumab probably reduces the risk of clinical fragility fractures** (12.2-51.5 fewer in 1,000) **and clinical vertebral fractures** (16.2-18.2 fewer in 1,000).<br>- Denosumab probably does not reduce all-cause mortality and probably does not change health-related quality of life.<br><br>Denosumab (men)<br>- The evidence for hip, clinical fragility and clinical vertebral fractures, and all-cause mortality was very uncertain.	

Appendix 10, as supplied by the authors. Appendix to: Thériault G, Limburg H, Klarenbach S, et al. Recommendations on screening for primary prevention of fragility fractures. *CMAJ* 2023. doi: 10.1503/cmaj.221219. Copyright © 2023 The Author(s) or their employer(s). To receive this resource in an accessible format, please contact us at cmajgroup@cmaj.ca.

Outcome & Study approach	Studies; sample size; follow-up	Anticipated absolute effects (95% CI)			Relative OR (95% CI)	Certainty	Judgement
		Assumed population risk*	Risk with treatment	Absolute difference			
**Bisphosphonates (alendronate, risedronate or zoledronic acid) vs placebo (postmenopausal females)**							
Hip fractures<br><br>Intention to treat	14 RCT; 21,038<br><br>Follow-up: 12-72 months<br><br>(61–76)	Study data:<br><br>11 in 1000	8.1 per 1,000 (6.4 t 10.1)	**2.9 fewer in 1000 (4.6 fewer to 0.9 fewer)**	0.73 (0.58 to 0.92)	Low[a,c]	**May reduce**
		General F ≥65 y:<br><br>20 in 1000	14.7 per 1,000 (11.7 to 18.4)	**5.3 fewer in 1000 (8.3 fewer to 1.6 fewer)**			
Clinical fragility fractures<br><br>Intention to treat or exposed to ≥1 dose	19 RCT; 22,482<br><br>Follow-up: 12-72 months<br><br>(61,63,73–82,64,83–85,65–71)	Study data:<br><br>58 in 1000	46.9 per 1,000 (43.0 to 51.4)	**11.1 fewer in 1000 (15.0 fewer to 6.6 fewer)**	0.80 (0.73 to 0.88)	Moderate[a,c]	**Probably reduces**
		General F ≥65 y:<br><br>202 in 1000	168.4 per 1,000 (156.0 to 182.2)	**33.6 fewer in 1000 (46.0 fewer to 19.8 fewer)**			
Clinical vertebral fractures; Intention to treat or exposed to ≥1 dose	11 RCT; 8,921<br><br>Follow-up: 12-72 months<br><br>(61,62,80,82,85,63–69,72)	Study data:<br><br>21 in 1000	11.0 per 1000 (7.0 to 17.1)	**10.0 fewer in 1000 (14.0 fewer to 3.9 fewer)**	0.52 (0.33, 0.81)	Low[a,b,d]	**May reduce**
		General F ≥65 y:<br><br>27 in 1000	14.2 per 1000 (9.1 to 22.0)	**12.8 fewer in 1000 (17.9 fewer to 5.0 fewer)**	0.52 (0.33, 0.81)		

Appendix 10, as supplied by the authors. Appendix to: Thériault G, Limburg H, Klarenbach S, et al. Recommendations on screening for primary prevention of fragility fractures. *CMAJ* 2023. doi: 10.1503/cmaj.221219. Copyright © 2023 The Author(s) or their employer(s). To receive this resource in an accessible format, please contact us at cmajgroup@cmaj.ca.

All-cause mortality<br><br>Intention to treat or exposed to ≥1 dose	8 RCT; 8,542<br><br>Follow-up: 12-72 months<br><br>(62–64,67,68,72,81, 84,86)	Study data:<br><br>30 in 1000	24.0 per 1,000 (17.6 to 32.9)	5.5 fewer in 1000<br><br>(11.9 fewer to 3.4 more)	0.81 (0.59 to 1.12)	Low[a-c]	**May not reduce**
		General F >65 y:<br><br>57 in 1000	46.7per 1,000 (34.4 to 63.4)	10.3 fewer in 1000<br><br>(22.6 fewer to 6.4 more)			
**Alendronate vs placebo (post-menopausal females)**							
Hip fractures<br><br>Intention to treat	7 RCT; 9,226 post-menopausal<br><br>Females<br><br>Follow-up: 12-48 months (61,62,68–73)	Study data:<br><br>8 in 1000	5.9 per 1000 (3.5 to 9.9)	2.1 fewer in 1000<br><br>(4.5 fewer to 1.9 more)	0.73 (0.43, 1.24)	Low[b,c,d]	**May not reduce**
		General F ≥65 y:<br><br>20 in 1000	5.9 per 1000 (3.5 to 9.9)	5.3 fewer in 1000<br><br>(11.3 fewer to 4.7 more)	0.73 (0.43, 1.24)		
Clinical fragility fractures<br><br>Intention to treat	8 RCT; 8,854 post-menopausal females<br><br>Follow-up: 12-48 months<br><br>(61,68,81,69–71,73,77–80)	Study data:<br><br>96 in 1000	79.3 per 1000 (69.5 to 91.4)	**14.7 fewer in 1000 (24.5 fewer to 2.6 fewer)**	0.83 (0.72, 0.97)	Moderate[b,c]	**Probably reduces**
		General F ≥65 y:<br><br>202 in 1000	173.6 per 1000 (154.2 to 197.1)	**28.4 fewer in 1000 (47.8 fewer to 4.9 fewer)**	0.83 (0.72, 0.97)		

Appendix 10, as supplied by the authors. Appendix to: Thériault G, Limburg H, Klarenbach S, et al. Recommendations on screening for primary prevention of fragility fractures. *CMAJ* 2023. doi: 10.1503/cmaj.221219. Copyright © 2023 The Author(s) or their employer(s). To receive this resource in an accessible format, please contact us at cmajgroup@cmaj.ca.

Clinical vertebral fractures	The evidence from (n=6,324, follow-up: 12-48 months) is very uncertain is very uncertain (61,62,68,69,72,80).					Very Low[a-d]	Very uncertain
All-cause mortality	The evidence from 4 RCTs (n=5,272, follow-up: 12-48 months) is very uncertain (62,68,72,81,86).					Very Low[b,c]	Very uncertain
**Risedronate vs placebo (post-menopausal females)**							
Hip fractures<br><br>Intention to treat	4 RCT; 9,672 post-menopausal females<br><br>Follow-up: 12-36 months (63,74–76)	Study data:<br><br>30 in 1000	22.1 per 1000 (17.0 to 28.5)	**7.9 fewer in 1000**<br><br>**(13.0 fewer to 1.5 fewer)**	0.73 (0.56 to 0.95)	Low[b,c]	**May reduce**
		General F ≥65 y:<br><br>20 in 1000	14.7 per 1000 (11.3 to 19.0)	**5.3 fewer in 1000**<br><br>**(8.7 fewer to 1.0 fewer)**	0.73 (0.56 to 0.95)		
Clinical fragility fractures<br><br>Intention to treat or exposed to ≥1 dose	7 RCT; 10,572 post-menopausal females<br><br>Follow-up: 12-36 months (63,74–76,78,82,83)	Study data:<br><br>48 in 1000	40.2 per 1000 (35.5 to 45.7)	**7.8 fewer in 1000 (12.5 fewer to 2.3 fewer)**	0.83 (0.73, 0.95)	Low[a-c]	**May reduce**
		General F ≥65 y:<br><br>202 in 1000	173.6 per 1000 (156.0 to 193.9)	**28.4 fewer in 1000 (46.0 fewer to 8.1 fewer)**	0.83 (0.73, 0.95)		
Clinical vertebral fractures	The evidence from 2 RCTs (n=230, follow-up: 12-24 months) is very uncertain (63,82).					Very Low[a,c,d]	Very uncertain
All-cause mortality	The evidence from 1 RCT (n=170, follow-up: 12 months) is very uncertain (63).					Very Low[a,b,d]	Very uncertain

Appendix 10, as supplied by the authors. Appendix to: Thériault G, Limburg H, Klarenbach S, et al. Recommendations on screening for primary prevention of fragility fractures. *CMAJ* 2023. doi: 10.1503/cmaj.221219. Copyright © 2023 The Author(s) or their employer(s). To receive this resource in an accessible format, please contact us at cmajgroup@cmaj.ca.

**Zoledronic acid vs placebo (post-menopausal females)**							
Hip fractures<br><br>Intention to treat	3 RCT; 2,200<br><br>Follow-up: 12-72 months<br><br>(64–67)	Study data:<br><br>12 in 1000	8.3 per 1,000 (3.5 to 19.4)	3.7 fewer in 1000<br><br>(8.5 fewer to 7.4 more)	0.69 (0.29 to 1.63)	Low[b,c]	May not reduce
		General F ≥65 y:<br><br>20 in 1000	13.9 per 1,000 (5.9 to 32.2)	6.1 fewer in 1000<br><br>(14.1 fewer to 12.2 more)			
Clinical fragility fractures<br><br>Intention to treat	5 RCT; 3,218<br>Follow-up: 12-72 months<br><br>(64–67,84,85)	Study data:<br><br>50 in 1000	37.9 per 1,000 (30.4 to 48.1)	20.1 fewer in 1000<br>(27.6 fewer to 9.9 fewer)	0.64 (0.51 to 0.82)	Moderate[b]	Probably reduces
		General F ≥65 y:<br><br>202 in 1000	139.4 per 1,000 (114.3 to 171.9)	62.6 fewer in 1000<br>(87.7 fewer to 30.1 fewer)			
Clinical vertebral fractures<br><br>Intention to treat	4 RCT; 2,367<br>Follow-up: 12-72 months (64–67,85)	Study data:<br><br>34 in 1000	15.3 per 1,000 (8.4 to 27.4)	18.7 fewer in 1000<br>(25.6 fewer to 6.6 fewer)	0.44 (0.24 to 0.8)	Low[a,b,d]	May reduce
		General F ≥65 y:<br><br>27 in 1000	12.1 per 1,000 (6.6 to 21.7)	14.9 fewer in 1000<br>(20.4 fewer to 5.3 fewer)			
All-cause mortality	The evidence from 3 RCTs (n=2,656, follow-up=12-72 months) is very uncertain (64,67,84)					Very Low[a,b,c,d]	Very uncertain
**Zoledronic acid vs placebo (men)**							

Appendix 10, as supplied by the authors. Appendix to: Thériault G, Limburg H, Klarenbach S, et al. Recommendations on screening for primary prevention of fragility fractures. *CMAJ* 2023. doi: 10.1503/cmaj.221219. Copyright © 2023 The Author(s) or their employer(s). To receive this resource in an accessible format, please contact us at cmajgroup@cmaj.ca.

Hip fractures<br><br>Intention to treat	1 RCT; 1,199<br>Follow-up: 24 months<br><br>(87)	Study data:<br><br>2 in 1000	4.2 per 1,000 (0.4 to 44.0)	2.2 more in 1000<br><br>(1.6 fewer to 42.0 more)	2.08 (0.19 to 22.98)	Low[b,c]	May not reduce
		General M ≥65 y:<br><br>16 in 1000	32.7 per 1,000 (3.1 to 272.0)	16.7 more in 1000<br><br>(12.9 fewer to 256.0 more)			
Clinical fragility fractures<br><br>Intention to treat	1 RCT; 1,199<br>Follow-up: 24 months<br><br>(87)	Study data:<br><br>18 in 1000	10.3 per 1,000 (3.8 to 27.5)	7.7 fewer in 1000<br><br>(14.2 fewer to 9.5 more)	0.57 (0.21 to 1.54)	Low[b,d]	May not reduce
		General M ≥65 y:<br><br>105 in 1000	62.7 per 1,000 (24.0 to 153.0)	42.3 fewer in 1000<br><br>(81.0 fewer to 48.0 more)			
Clinical vertebral fractures	No study reported on this outcome.						
All-cause mortality	The evidence from 1 RCT (n=1,199, follow-up=24 months) is very uncertain (87).					Very Low[a,b,d]	Very uncertain
**Denosumab vs placebo (post-menopausal females)**							
Hip fractures<br><br>Intention to treat	3 RCT; 8,542<br><br>Follow-up: 6-36 months	Study data:<br><br>11 in 1000	7.1 per 1,000 (4.3 to 11.2)	3.9 fewer in 1000<br><br>(6.7 fewer to 0.2 more)	0.64 (0.39 to 1.02)	Low[b-d]	**May not reduce**

Appendix 10, as supplied by the authors. Appendix to: Thériault G, Limburg H, Klarenbach S, et al. Recommendations on screening for primary prevention of fragility fractures. *CMAJ* 2023. doi: 10.1503/cmaj.221219. Copyright © 2023 The Author(s) or their employer(s). To receive this resource in an accessible format, please contact us at cmajgroup@cmaj.ca.

			(88–92)	General F ≥65 y: 20 in 1000	12.9 per 1,000 (7.9 to 20.4)	7.1 fewer in 1000 (12.1 fewer to 0.4 more)			
		Clinical fragility fractures<br>Intention to treat or exposed to ≥1 dose	5 RCT; 9,231<br>Follow-up: 12-36 months<br>(81,86,88–91,93)	Study data: 42 in 1000	29.8 per 1,000 (25.2 to 34.7)	**12.2 fewer in 1000 (16.8 fewer to 7.3 fewer)**	0.70 (0.59 to 0.82)	Moderate[b]	**Probably reduces**
				General F ≥65 y: 202 in 1000	150.5 per 1,000 (129.9 to 171.9)	**51.5 fewer in 1000 (72.1 fewer to 30.1 fewer)**			
		Clinical vertebral fractures<br>Intention to treat or exposed to ≥1 dose	3 RCT; 8,397 Follow-up: 6-36 months<br>(88,91,93–95)	Study data: 24 in 1000	7.8 per 1,000 (5.1 to 11.9)	**16.2 fewer in 1000 (18.9 fewer to 12.1 fewer)**	0.32 (0.21 to 0.49)	Moderate[b]	**Probably reduces**
				General F ≥65 y: 27 in 1000	8.8 per 1,000 (5.8 to 13.4)	**18.2 fewer in 1000 (21.2 fewer to 13.6 fewer)**			
		All-cause mortality<br>Intention to treat or exposed to ≥1 dose	5 RCT; 9,185<br>Follow-up: 6-36 months<br>(81,86,88–91,93,95)	Study data: 23 in 1000	18.3 per 1,000 (13.5 to 24.8)	4.7 fewer in 1000 (9.5 fewer to 1.8 more)	0.79 (0.58 to 1.08)	Moderate[b,d]	**Probably does not reduce**
				General F >65 y: 57 in 1000	45.6 per 1,000 (33.9 to 61.3)	11.4 fewer in 1000 (23.1 fewer to 4.3 more)			

Appendix 10, as supplied by the authors. Appendix to: Thériault G, Limburg H, Klarenbach S, et al. Recommendations on screening for primary prevention of fragility fractures. *CMAJ* 2023. doi: 10.1503/cmaj.221219. Copyright © 2023 The Author(s) or their employer(s). To receive this resource in an accessible format, please contact us at cmajgroup@cmaj.ca.

Health-related quality of life (OPAQ-SV; 0-100; higher = better) after 3-y of treatment	1 RCT; 6,481<br><br>Follow-up: 36 months<br><br>(96)	Change from baseline: physical function (-1.3 vs. -1.2), emotional status (-1.4 vs. -1.6), and back pain (4.1 vs. 4.3) for denosumab vs. placebo.				Moderate[b,c]	**Probably does not change**
**Denosumab vs placebo (men)**							
Hip fractures<br><br>Intention to treat	1 RCT; 242<br><br>Follow-up: 12 months<br><br>(97)	Study data:<br><br>0 in 1000	0.0 per 1,000 (0 to 0)	No difference in 1000	1.00 (0.02 to 50.80)	Very low[a,b,d]	Very uncertain
		General M ≥65 y: 16 in 1000	16.0 per 1,000 (0.3 to 452.4)	No difference in 1000<br><br>(15.7 fewer to 436.4 more)			
Clinical fragility fractures<br><br>Intention to treat	1 RCT; 242<br><br>Follow-up: 12 months<br><br>(97)	Study data:<br><br>16 in 1000	8.6 per 1,000 (0.7 to 88.2)	8.4 fewer in 1000<br><br>(16.3 fewer to 71.2 more)	0.50 (0.04 to 5.59)	Very low[a,b,d]	Very uncertain
		General M ≥65 y:<br><br>105 in 1000	55.4 per 1,000 (4.7 to 396.1)	49.6 fewer in 1000<br><br>(100.3 fewer to 291.1 more)			
Clinical vertebral fractures<br><br>Intention to treat	1 RCT; 242<br><br>Follow-up: 12 months<br><br>(97)	Study data:<br><br>0 in 1000	0.0 per 1,000 (0.0 to 0.0)	No difference in 1000	1.00 (0.02 to 50.80)	Very low[a,b,d]	Very uncertain
		General M ≥65 y:<br><br>10 in 1000	10.0 per 1,000 (0.2 to 339.1)	No difference in 1000<br><br>(9.8 fewer to 329.1 more)			

Appendix 10, as supplied by the authors. Appendix to: Thériault G, Limburg H, Klarenbach S, et al. Recommendations on screening for primary prevention of fragility fractures. *CMAJ* 2023. doi: 10.1503/cmaj.221219. Copyright © 2023 The Author(s) or their employer(s). To receive this resource in an accessible format, please contact us at cmajgroup@cmaj.ca.

		All-cause mortality<br><br>Exposed to ≥1 dose	1 RCT; 240<br><br>Follow-up: 12 months<br><br>(97)	Study data:<br><br>8 in 1000	8.0 per 1,000 (0.5 to 115.4)	No difference in 1000<br><br>(7.5 fewer to 107.4 more)	1.00 (0.06 to 16.17)	Very low[a,b,d]	Very uncertain
				General M >65 y:<br><br>76 in 1000	76.0 per 1,000 (4.9 to 570.8)	No difference in 1000<br><br>(71.1 fewer to 494.8 more)			

CI=confidence interval; RCT=randomized controlled trial; NA=not applicable; OPAQ-SV=Osteoporosis Assessment Questionnaire-Short Version; y=years

a=risk of bias; b=inconsistency; c=indirectness; d=imprecision

[*] The effects without screening for the general risk population are estimated from Prior et al. based on 10 year follow-up (28). Data for the general population <65 years is not included in the summary table.

UNDESIRABLE EFFECTS	**How substantial are the undesirable anticipated effects?**<br><br>○Large<br>**X** Small<br>○Moderate<br>○Trivial<br><br>○Varies<br>○Don't know	**KQ1a: What are the harms of screening compared with no screening to prevent fragility fractures and related morbidity and mortality in primary care for adults ≥ 40 years?**	

Appendix 10, as supplied by the authors. Appendix to: Thériault G, Limburg H, Klarenbach S, et al. Recommendations on screening for primary prevention of fragility fractures. *CMAJ* 2023. doi: 10.1503/cmaj.221219. Copyright © 2023 The Author(s) or their employer(s). To receive this resource in an accessible format, please contact us at cmajgroup@cmaj.ca.

JUDGEMENT – HARMS OF SCREENING

Selected Populations[9]: (Offer-to-screen, females ≥65 years)
The evidence for serious adverse events was very uncertain.
(See KQ3b for further harms of treatment)
Overdiagnosis: Screening resulted in 11.8% (using 10-year hip fracture risk) and 19.3% (using 10-year MOF[10] risk) being identified as high risk that would never have known they were at risk and would never have experienced a fracture. This overdiagnosis may result in labelling, anxiety and/or unnecessary treatment.

Selected Populations[9]: (Acceptors of screening, females 70-85 years)
Overdiagnosis: Screening resulted in 24.1% being overdiagnosed using 10-year hip fracture risk.

EVIDENCE TABLES

Table 5.1 Harms of Screening (Serious adverse events)

- The evidence about serious adverse events in offer-to-screen in selected populations (females 70-85 y) is very uncertain.

Appendix 10, as supplied by the authors. Appendix to: Thériault G, Limburg H, Klarenbach S, et al. Recommendations on screening for primary prevention of fragility fractures. *CMAJ* 2023. doi: 10.1503/cmaj.221219. Copyright © 2023 The Author(s) or their employer(s). To receive this resource in an accessible format, please contact us at cmajgroup@cmaj.ca.

Outcome	Study approach	Population<br><br>Studies; Sample size	Findings	Certainty	Judgement
**Serious adverse events**	Offer-to-screen in selected[9] population<br><br>Follow-up: 5 years	Females 70-85 y;<br><br>1 RCT (SCOOP)(26); 12,483	No serious adverse events reported	Very Low to Low[a,b,d]	Very uncertain

RCT: randomized controlled trial; y: years
a=risk of bias; b=inconsistency; c=indirectness; d=imprecision

Table 5.2 Harms of Screening (Overdiagnosis)

- For those ***offered screening in selected populations*[9], 11.8%** (females 70-85 y, using 10-year hip fracture risk) and **19.3%** (females 65-90 y, using 10-year MOF[10] risk may be identified as high risk that would never have known they were at risk and would never experienced a fracture. For those who ***accepted screening in selected populations*[9], 29%** (females 70-85 y, using 10-year hip fracture risk) may be identified as high risk that would never have known they were at risk and would never experienced a fracture.

Outcome	Study approach	Population<br><br>Studies; Sample size	Findings	Certainty
**Overdiagnosis**	Offer-to-screen in selected[9] populations	Females 70-85 y;<br><br>1 RCT (SCOOP) (26); 6,233	14.4 x (100-17.9) /100 = **11.8% overdiagnosed** (using 10-year hip fracture risk)	Low[c]
		Females 65-90 y;<br><br>1 RCT (SALT) (25); 5,575	25.4 x (100-23.9) / 100 = **19.3% overdiagnosed**	Low[c]

[9] Females ≥65 years willing to independently complete a mailed fracture risk questionnaire.
[10] MOF=Major osteoporotic fracture.

Appendix 10, as supplied by the authors. Appendix to: Thériault G, Limburg H, Klarenbach S, et al. Recommendations on screening for primary prevention of fragility fractures. *CMAJ* 2023. doi: 10.1503/cmaj.221219. Copyright © 2023 The Author(s) or their employer(s). To receive this resource in an accessible format, please contact us at cmajgroup@cmaj.ca.

			(using 10-year MOF risk)	
	Acceptors of screening in selected[9] populations	Females 70-85 y;<br><br>1 RCT (SCOOP) (26); 2,750	29.3 x (100-17.9) / 100 = **24.1% overdiagnosed**<br><br>(using 10-year hip fracture risk)	Low[c]

RCT: randomized controlled trial; y: years
a=risk of bias; b=inconsistency; c=indirectness; d=imprecision

KQ3b. What are the harms of pharmacologic treatments to prevent fragility fractures among adults ≥40 years?

Appendix 10, as supplied by the authors. Appendix to: Thériault G, Limburg H, Klarenbach S, et al. Recommendations on screening for primary prevention of fragility fractures. *CMAJ* 2023. doi: 10.1503/cmaj.221219. Copyright © 2023 The Author(s) or their employer(s). To receive this resource in an accessible format, please contact us at cmajgroup@cmaj.ca.

JUDGEMENT – HARMS OF TREAMENT

Serious harms

Bisphosphonates (as a class) and alendronate may increase rare but serious harms of subtrochanteric atypical femoral fracture and osteonecrosis of the jaw.

The risk of 'any serious adverse event' (composite outcome) is probably not increased with risedronate and zoledronic acid and may not be increased with alendronate.

The risk of certain serious gastrointestinal (GI) adverse events (perforations, ulcers, and bleeds; serious esophageal) may not be increased with alendronate.

The risk of stroke and myocardial infarction probably does not increase with bisphosphonates (as a class); and the risk of other serious cardiovascular events may not increase with alendronate, zoledronic acid and denosumab.

Non-serious harms

Alendronate and denosumab probably increase non-serious GI adverse events.

Zoledronic acid probably increases any non-serious adverse event (AE), pyrexia, headache, influenza-like symptoms, arthritis and arthralgia, myalgia and may increase the composite measure of arthralgia, myalgia, pyrexia, chills, & influenza-like symptoms.

Denosumab probably increases rash/eczema and infections and may increase eczema alone.

Appendix 10, as supplied by the authors. Appendix to: Thériault G, Limburg H, Klarenbach S, et al. Recommendations on screening for primary prevention of fragility fractures. *CMAJ* 2023. doi: 10.1503/cmaj.221219. Copyright © 2023 The Author(s) or their employer(s). To receive this resource in an accessible format, please contact us at cmajgroup@cmaj.ca.

EVIDENCE TABLES

Table 6.1 Harms of Treatment (Alendronate)

- Alendronate may increase subtrochanteric atypical femoral fractures (0.06-0.08 more per 1,000) and osteonecrosis of the jaw (0.22 more per 1,000).
- Alendronate may not increase the composite "any serious AE", the composite "GI perforations, ulcers, or bleeds", serious esophageal AEs or atrial fibrillation.
- Alendronate probably increases non-serious GI events (16.3 more per 1,000) but probably does not increase discontinuation due to AE and may not increase the composite measure of any non-serious AE.
- Evidence for serious GI (any), GI cancer, serious cardiovascular AE and atypical femoral fractures (any, with treatment >3 years) was uncertain.

Outcome	Studies; sample Size	Assumed pop. risk*	Absolute effects (95% CI)	Relative RR unless otherwise stated (95% CI)	Certainty	Judgement
**Alendronate vs. placebo or no treatment**						
**Serious adverse events**						
Atypical femoral fractures (subtrochanteric) (99)	1 cohort; 220,360	0.06 per 1000	**0.08 more per 1000 (0.05 more to 0.14 more)**	**HR** 2.41 (1.78 to 3.27)	Low[a,b,e]	**May increase**
Atypical femoral fractures (femoral shaft) (99)	1 cohort; 220,360	0.03 per 1000	**0.06 more per 1000 (0.03 more to 0.10 more)**	**HR** 2.90 (1.97 to 4.26)	Low[a,b,e]	**May increase**
Osteonecrosis of the jaw (99)	1 cohort; 220,360	0.1 per 1000	**0.22 more per 1000 (0.04 more to 0.59 more)**	**HR** 3.15 (1.44 to 6.87)	Low[a,b,e]	
Any serious AE) (5,100)	5 RCT; 1,955	106 per 1000	5.7 fewer per 1000 (31.9 fewer to 29.4 more)	0.95 (0.71 to 1.27)	Low[a,b]	**May not increase**
GI perforations, ulcers, bleeds (2,101,102)	10 RCT; 137 Events	NR	Cannot be calculated; NS difference	0.89 (0.63 to 1.25)	Low[a,b]	

Appendix 10, as supplied by the authors. Appendix to: Thériault G, Limburg H, Klarenbach S, et al. Recommendations on screening for primary prevention of fragility fractures. *CMAJ* 2023. doi: 10.1503/cmaj.221219. Copyright © 2023 The Author(s) or their employer(s). To receive this resource in an accessible format, please contact us at cmajgroup@cmaj.ca.

Serious esophageal AE (100–102)	5 RCT; 499,062	NR	Cannot be calculated; NS difference	1.39 (0.75 to 2.65)	Low[a,b,d]	
Atrial fibrillation (100–102)	1 RCT; NR	14 per 1000	3.6 more per 1000 (0.6 fewer to 9.0 more)	**OR** 1.26 (0.96 to 1.66)[†]	Low[a,b]	
	1 SR of 32 RCT; 17,291		2.2 more per 1000 (1.8 fewer to 7.7 more)			
<u>Very uncertain:</u> Serious GI AEs (any) [a,b,d] (2,101,102), GI cancer (colorectal[b,], gastric[d], esophageal[b,d], liver[b], pancreatic[d], oral[b,d], bile duct[b,d], small intestinal[b,d]) (103), serious cardiovascular AE (acute coronary syndrome[a,b,d], cerebrovascular death[ra,b,d], thromboembolic events[a,b,d]) (2,101,102), and atypical femoral fractures (any[b,d], with long term treatment [>3 years][a,b]) (99).<br><u>No evidence:</u> serious stroke, pulmonary embolism.						
**Non-serious adverse events and discontinuation due to AE**						
Non-serious GI AE (2,101,102)	50 RCT; 22,549	589 per 1000	**16.3 more per 1000 (2.4 more to 31.3 more)**	**OR** 1.07 (1.01 to 1.14)[‡]	Moderate[a]	**<u>Probably increases</u>**
Discontinuation due to AE (5,100)	9 RCT; 9,160	68 per 1000	1.4 fewer per 1000 (10.0 fewer to 8.3 more)	0.98 (0.85 to 1.12)	Moderate[a]	**<u>Probably does not increase</u>**
Any non-serious AE (104)	5 RCT; 4,720	815 per 1000	16.3 fewer per 1000 (81.5 fewer to 48.9 more)	0.98 (0.90 to 1.06)	Low[a,b]	**<u>May not increase</u>**
Very uncertain: Influenza-like symptoms[a,b,d] (104), and musculoskeletal (arthritis and arthralgia[a,b,d]; myalgia, cramps, and limb pain[a,b,d]) AEs (2,101,102).						

AE=adverse event; GI=gastrointestinal; NR=not reported; NS=not statistically significant; RCT=randomized controlled trial; SR=systematic review

*The control event rate is the median rate in the control group for studies in the analysis. These were extracted directly from the systematic reviews when possible. Otherwise, we extracted these data from the included primary studies when there were ≤5 in the analysis or used the 5 largest studies from larger analyses to calculate the control event rate.

a=risk of bias; b=inconsistency; c=indirectness; d=imprecision; e=Large magnitude of effect (+1)

[†]The absolute effect (and its 95% CI) without treatment (i.e. baseline rate) is based on the estimated risk in the comparison group; the effect with treatment is based on applying the relative effect of the intervention (and its 95% CI) to the effect without treatment. When an OR is presented, we used the following formula recommended in the Cochrane manual: intervention risk per 1000 = 1000 x (OR x ACR / 1 – ACR + (OR x ACR))

[‡]Odds ratio derived from exact logistic regression meta-analysis (101).

Table 6.2 Harms of Treatment (Risedronate)

Appendix 10, as supplied by the authors. Appendix to: Thériault G, Limburg H, Klarenbach S, et al. Recommendations on screening for primary prevention of fragility fractures. *CMAJ* 2023. doi: 10.1503/cmaj.221219. Copyright © 2023 The Author(s) or their employer(s). To receive this resource in an accessible format, please contact us at cmajgroup@cmaj.ca.

- Risedronate probably does not increase any serious AE, any non-serious AE, any non-serious GI AE or discontinuation due to AEs.
- Evidence for influenza-like symptoms, pharyngitis, and arthritis or arthralgia was very uncertain.

Outcome	Studies; sample size	Assumed pop. risk*	Absolute effects (95% CI)	Relative RR unless otherwise stated (95% CI)	Certainty	Judgement
**Risedronate vs. placebo**						
**Serious adverse events**						
Any serious AE (5,100)	5 RCT; 7,195	11 per 1000	2.6 fewer per 1000 (10.2 fewer to 5.7 more)	0.98 (0.91 to 1.05)	Moderate[a]	**Probably does not increase**
Very uncertain: serious GI AEs (all[a,c]; GI perforations, ulcers bleeds[a,b,d]; serious esophageal AE[a,b,d]) (2,101,102), GI cancer[a,b,d] (2,101,102), acute coronary syndrome[a,b,d], cerebrovascular death[a,b,d], pulmonary embolism[a,b,d] (2,101,102), atrial fibrillation[a,b,d] (2,101,102).<br>No evidence: serious stroke, thromboembolic events (2,101,102), atypical femoral fractures, or osteonecrosis of the jaw.						
**Non-serious adverse events and discontinuation due to AE**						
Any non-serious AE (104)	6 RCT; 9,575	915 in 1000	45.8 fewer in 1000 (146.4 fewer to 73.2 more)	0.95 (0.84 to 1.08)	Moderate[a]	**Probably does not increase**
Non-serious GI AE (2,101,102)	21 RCT; 3,474 Events	223 in 1000	5.2 more in 1000 (8.8 fewer to 20.3 more)	**OR** 1.03 (0.95 to 1.12)[‡]	Moderate[a]	
Discontinuation due to AE (5,100)	5 RCT; 7,159	111 in 1000	1.0 fewer in 1000 (11.8 fewer to 10.9 more)	0.99 (0.89 to 1.10)	Moderate[a]	
Very uncertain: influenza-like symptoms[a,b,d] (104), pharyngitis[a,b,d] (104), and arthritis and arthralgia[a,b,d] (2,101,102).<br>No evidence: myalgia, cramps, and limb pain (2,101,102)						

AE=adverse event; GI=gastrointestinal; NR=not reported; NS=not statistically significant; RCT=randomized controlled trial; SR=systematic review

* The control event rate is the median rate in the control group for studies in the analysis. These were extracted directly from the systematic reviews when possible. Otherwise, we extracted these data from the included primary studies when there were ≤5 in the analysis or used the 5 largest studies from larger analyses to calculate the control event rate.

a=risk of bias; b=inconsistency; c=indirectness; d=imprecision; e=Large magnitude of effect (+1)

‡Odds ratio derived from exact logistic regression meta-analysis (101).

Appendix 10, as supplied by the authors. Appendix to: Thériault G, Limburg H, Klarenbach S, et al. Recommendations on screening for primary prevention of fragility fractures. *CMAJ* 2023. doi: 10.1503/cmaj.221219. Copyright © 2023 The Author(s) or their employer(s). To receive this resource in an accessible format, please contact us at cmajgroup@cmaj.ca.

Table 6.3 Harms of Treatment (Zoledronic acid)

- Zoledronic acid probably does not increase any serious AE and may not increase acute coronary syndrome, serious stroke, or non-serious GI AEs.
- Evidence for cerebrovascular death, atrial fibrillation, atypical femoral fractures and osteonecrosis of the jaw was very uncertain.
- Zoledronic acid probably increases the composite of "any non-serious AE", pyrexia, headache, influenza-like symptoms, arthritis and arthralgia, myalgia, the composite of arthralgia, myalgia, pyrexia, chills and flu-like symptoms, and chills
- Zoledronic acid may not increase non-serious GI AEs
- The evidence for discontinuation due to AEs is very uncertain

Outcome	Studies; sample size	Assumed pop. risk*	Absolute effects (95% CI)	Relative RR unless otherwise stated (95% CI)	Certainty	Judgement
**Zoledronic acid vs. placebo**						
**Serious adverse events**						
Any serious AE (5,100)	3 RCT; 1,950	114 in 1000	0.9 fewer in 1000 (19.8 fewer to 21.8 more)	0.99 (0.83 to 1.19)	Moderate[a]	**Probably does not increase**
Acute coronary syndrome (2,101,102)	2 RCT; NR	NR	Cannot be calculated; NS difference	**OR** 0.82 (0.55 to 1.21)‡	Low[a,b,d]	**May not increase**
Serious stroke (2,101,102)	2 RCT; NR	NR	Cannot be calculated; NS difference	**OR** 1.13 (0.90 to 1.42)‡	Low[a,b,d]	
Very uncertain: cerebrovascular death[a,b,d] (2,101,102), atrial fibrillation[a,d] (2,101,102), atypical femoral fractures[a,b,d] (2,101,102), osteonecrosis of the jaw[a,b,d] (2,101,102).<br>No evidence: serious GI AE (any; GI perforations, ulcers, bleeds; serious esophageal AE), GI cancer, pulmonary embolism, thromboembolic events.						
**Non-serious adverse events and discontinuation due to AE**						
Any non-serious AE (104)	6 RCT; 9,575	915 in 1000	**51.8 more per 1000 (no difference to 112.2 more)**	1.06 (1.00 to 1.13)	Moderate[a]	**Probably increases**
Pyrexia (104)	5 RCT; 11,823	38 in 1000	**127.7 more in 1000 (34.6 more to 337.4 more)**	4.36 (1.91 to 9.88)	Moderate[a]	
Headache (104)	4 RCT; 9,712	53 in 1000	**60.4 more in 1000 (19.1 more to 126.7 more)**	2.14 (1.36 to 3.39)	Moderate[a]	

Appendix 10, as supplied by the authors. Appendix to: Thériault G, Limburg H, Klarenbach S, et al. Recommendations on screening for primary prevention of fragility fractures. *CMAJ* 2023. doi: 10.1503/cmaj.221219. Copyright © 2023 The Author(s) or their employer(s). To receive this resource in an accessible format, please contact us at cmajgroup@cmaj.ca.

Influenza-like symptoms (2,101,102)	5 RCT; 10,695	44 in 1000	**142.5 more in 1000 (105.5 more to 188.4 more)**	**OR** 4.98 (3.82 to 6.58)[‡]	Moderate[a]	
Arthritis and arthralgia (2,101,102)	6 RCT; 11,171	145 in 1000	**178.5 more in 1000 (137.4 more to 224.1 more)**	**OR** 2.82 (2.32 to 3.45)[‡]	Moderate[a]	
Myalgia (2,101,102)	5 RCT; 11,065	17 in 1000	**70.7 more in 1000 (54.6 more to 90.8 more)**	**OR** 5.56 (4.46 to 6.99)[‡]	Moderate[a]	
Arthralgia, myalgia, pyrexia, chills, & influenza-like symptoms (2,101,102)	6 RCT; 11,676	219 in 1000	**422.8 more in 1000 (398.6 more to 446.3 more)**	**OR** 6.39 (5.76 to 7.09)[‡]	Low[a,c]	**May increase**
Chills (104)	2 RCT; 799	12 in 1000	**33.7 more in 1000 (3.0 more to 127.2 more)**	3.81 (1.25 to 11.6)	Low[a,b,d]	
Non-serious GI AE (2,101,102)	3 RCT; 840	79 in 1000	30.9 more in 1000 (11.8 fewer to 97.6 more)	**OR** 1.44 (0.84 to 2.50)[‡]	Low[a,b,d]	**May not increase**
Very uncertain: discontinuation due to AE[a,b,d] (5,100).						

AE=adverse event; GI=gastrointestinal; NR=not reported; NS=not statistically significant; RCT=randomized controlled trial;
*The control event rate is the median rate in the control group for studies in the analysis. These were extracted directly from the systematic reviews when possible. Otherwise, we extracted these data from the included primary studies when there were ≤5 in the analysis or used the 5 largest studies from larger analyses to calculate the control event rate.
a=risk of bias; b=inconsistency; c=indirectness; d=imprecision; e=Large magnitude of effect (+1)
[‡]Odds ratio derived from exact logistic regression meta-analysis (101).

Table 6.4 Harms of Treatment (Bisphosphonates as a class)

- Bisphosphonates as a class may increase atypical femoral fractures (any, with long-term treatment, >3 years = 11 (7 to 14) in 10,000 in-years, subtrochanteric = 0.2-1.1 more per 1000) and osteonecrosis of the jaw (0.3-43.0 in 1000).
- Bisphosphonates probably do not increase stroke or myocardial infarction (MI) and may not increase the composite of nonfatal stroke, MI or death from vascular cause or cardiovascular mortality.
- The evidence for esophageal cancer and atrial fibrillation was very uncertain.

Outcome	Studies; sample size	Assumed pop. risk*	Absolute effects (95% CI)	Relative RR unless otherwise stated	Certainty	Judgement

Appendix 10, as supplied by the authors. Appendix to: Thériault G, Limburg H, Klarenbach S, et al. Recommendations on screening for primary prevention of fragility fractures. *CMAJ* 2023. doi: 10.1503/cmaj.221219. Copyright © 2023 The Author(s) or their employer(s). To receive this resource in an accessible format, please contact us at cmajgroup@cmaj.ca.

				(95% CI)		
**Bisphosphonate vs. placebo or no treatment**						
**Serious adverse events**						
Atypical femoral fracture (any, with long-term treatment, >3 years) (99)	1 cohort; ~2.8 mill	0.3 in 1000†	**11 (7 to 14) in 10,000 in-years**	**OR** 126 (55 to 288)	Low[a,e]	**May increase**
	1 case-control; 1,368		**NA**	**OR** 93 (66 to 132) for >5 years of use		
	1 case-control; 290		**NA**	**OR** 25.65 (10.74 to 61.28)		
Atypical femoral fracture (subtrochanteric) (2,101,102)	3 RCT; NR	0.3 in 1000†	**1.0 more in 1000 (2.6 fewer to 41.1 more)**	1.33 (0.14 to 14.7)	Low[b]	
	1 SR of 11 observational; NR		**0.2 more in 1000 (0.1 more to 0.4 more)**	1.70 (1.22 to 2.37)		
	Pooled: safety databases; NR		**1.1 more in 1000 (0.7 more to 1.5 more)**	4.51 (3.44 to 5.92)		
Osteonecrosis of the jaw (2,101,102)	Case series, SRs; NR	NR	**Inconsistent, 0.3 to 43.0 in 1000**		Low[b]	
Stroke (105)	2 RCT; 9,825	33 in 1000	2.0 more in 1000 (5.9 fewer to 11.6 more)	1.06 (0.82 to 1.35)	Moderate[d]	**Probably does not increase**
Myocardial infarction (105)	5 RCT; 10,4040	12 in 1000	2.2 fewer in 1000 (5.2 fewer to 2.0 more)	0.82 (0.57 to 1.17)	Moderate[d]	
Nonfatal stroke, MI, death - vascular cause (105)	12 RCT; 16,888	67 in 1000	3.4 fewer in 1000 (8.7 fewer to 3.4 more)	0.95 (0.87 to 1.05)	Low[a,c]	**May not increase**
Cardiovascular mortality (105)	5 RCT; 10,165	22 in 1000	2.6 fewer in 1000 (8.4 fewer to 5.1 more)	0.88 (0.62 to 1.23)	Low[a,d]	
Very uncertain: esophageal cancer[b] (2,101,102) and atrial fibrillation[b,d] (2,101,102). No evidence: effect of long-term bisphosphonates (>3 years) on the risk of osteonecrosis of the jaw.						

MI=myocardial infarction; NA=not applicable; NR=not reported; RCT=randomized controlled trial; SR=systematic review
*The control event rate is the median rate in the control group for studies in the analysis. These were extracted directly from the systematic reviews when possible. Otherwise, we extracted these data from the included primary studies when there were ≤5 in the analysis or used the 5 largest studies from larger analyses to calculate the control event rate.

Appendix 10, as supplied by the authors. Appendix to: Thériault G, Limburg H, Klarenbach S, et al. Recommendations on screening for primary prevention of fragility fractures. *CMAJ* 2023. doi: 10.1503/cmaj.221219. Copyright © 2023 The Author(s) or their employer(s). To receive this resource in an accessible format, please contact us at cmajgroup@cmaj.ca.

†The absolute effect (and its 95% CI) without treatment (i.e. baseline rate) is based on the estimated risk in the comparison group; the effect with treatment is based on applying the relative effect of the intervention (and its 95% CI) to the effect without treatment. When an OR is presented, we used the following formula recommended in the Cochrane manual: intervention risk per 1000 = 1000 x (OR x ACR / 1 – ACR + (OR x ACR))
a=risk of bias; b=inconsistency; c=indirectness; d=imprecision; e=Large magnitude of effect (+1)

Table 6.5 Harms of Treatment (Denosumab)

- Denosumab may not increase any serious AE, serious cardiac events, stroke, the composite of cardiovascular death, MI or stroke or the composite of cardiovascular death, MI, stroke or heart failure.
- Denosumab probably does not increase any non-serious AE and may not increase discontinuation due to AEs.
- Evidence for serious infections, venous thromboembolism, composite of stroke, atrial fibrillation, heart failure and coronary artery disease, atrial fibrillation, atypical femoral fractures and osteonecrosis of the jaw is very uncertain
- Denosumab probably increases non-serious GI AEs (64.5 more in 1,000), rash or eczema (15.8 more in 1,000) and infections (1.8 more in 1,000) and may increase eczema (13.8 more in 1,000).
- Denosumab probably does not increase any non-serious AEs and may not increase discontinuations due to AEs.
- Evidence for arthralgia, injection-site reactions and rash was very uncertain
- Evidence for rebound fractures associated with discontinuation was very uncertain for non-vertebral, clinical vertebral and multiple clinical vertebral fractures.

Outcome	Studies; sample size	Assumed pop. risk*	Absolute effects (95% CI)	Relative RR unless otherwise stated (95% CI)	Certainty	Judgement
**Denosumab vs. placebo**						
**Serious adverse events**						
Any serious AE (5,100)	4 RCT; 8,663	81 per 1000	9.8 more per 1000 (10.0 fewer to 35.2 more)	1.12 (0.88 to 1.44)	Low[d]	**May not increase**
Serious cardiac events (2,101,102)	3 RCT; NR	NR	Cannot be calculated; NS difference	**OR** 1.04 (0.87 to 1.25)‡	Low[a,b,d]	
Stroke (106)	2 RCT; 7,733	NR	Cannot be calculated; NS difference	1.4% vs. 1.4%	Low[a,b,d]	
Cardiovascular death + MI +	4 RCT; 9,066	NR	Cannot be calculated; NS	1.00 (0.82 to 1.23)	Low[a,c]	

Appendix 10, as supplied by the authors. Appendix to: Thériault G, Limburg H, Klarenbach S, et al. Recommendations on screening for primary prevention of fragility fractures. *CMAJ* 2023. doi: 10.1503/cmaj.221219. Copyright © 2023 The Author(s) or their employer(s). To receive this resource in an accessible format, please contact us at cmajgroup@cmaj.ca.

stroke (106)			difference			
Cardiovascular death + MI + stroke + heart failure (107)	4 RCT; 9,066	NR	Cannot be calculated; NS difference	0.99 (0.83 to 1.19)	Low[a,c]	
<u>Very uncertain</u>: serious infections[d] (5,100), venous thromboembolism[a,b,d] (108); composite of stroke, atrial fibrillation, heart failure, coronary artery disease[a,b,c,d] (106); atrial fibrillation[a,b,d] (2,101,102), atypical femoral fractures[a,b,d] (99,108), and osteonecrosis of the jaw[a,b,d] (99,108).<br><u>No evidence:</u> serious GI AE (any; GI perforations, ulcers, bleeds; serious esophageal) (2,101,102), GI cancer (2,101,102), thromboembolic events (2,101,102), cardiac death (2,101,102).						
**Non-serious adverse events and discontinuation due to AE**						
Non-serious GI AE (2,101,102)	3 RCT; 8,454	105 in 1000	**64.5 more in 1000 (26.4 more to 113.3 more)**	**OR** 1.74 (1.29 to 2.38)‡	Moderate[a]	<u>**Probably increases**</u>
Rash or eczema (5,100)	3 RCT; 8,454	17 in 1000	**15.8 more in 1000 (7.6 more to 27.0 more)**	**OR** 1.96 (1.46 to 2.66)‡	Moderate[a]	
Infections (2,101,102)	4 RCT; 8,691	7 in 1000	**1.8 more in 1000 (0.1 more to 4.0 more)**	1.26 (1.01 to 1.57)	Moderate[a]	
Eczema (5,100)	1 RCT; 7,762	17 in 1000	**13.8 more in 1000 (5.8 more to 24.5 more)**	1.81 (1.34 to 2.44)	Low[a,b]	<u>**May increase**</u>
Any non-serious AE (108)	5 RCT; 9,201	907 in 1000	No difference in 1000 (9.1 fewer to 9.1 more)	1.00 (0.99 to 1.01)	Moderate[a]	<u>**Probably does not increase**</u>
Discontinuation due to AE (5,100)	3 RCT; 8,451	21 in 1000	Cannot be calculated; NS difference	1.14 (0.85 to 1.52)	Low[a,b,d]	<u>**May not increase**</u>
<u>Very uncertain:</u> arthralgia[a,b,d] (107), injection-site reactions[a,b,d] (5,100), and rash[a,b,d] (5,100).<br><u>No evidence:</u> influenza-like symptoms.						
**Rebound fractures with discontinuation (discontinuation of denosumab vs. discontinuation of placebo)**						
<u>Very uncertain:</u> non-vertebral fractures[a,b,c,d] (107), clinical vertebral fractures[a,b,c,d] (107), and multiple clinical vertebral fractures[a,b,c,d] (107).<br><u>No evidence:</u> There was **no evidence located** to comment on the effect of discontinuing denosumab on the risk of hip fracture.						

AE=adverse event; GI=gastrointestinal; MI=myocardial infarction; NR=not reported; NS=not statistically significant; RCT=randomized controlled trial

*The control event rate is the median rate in the control group for studies in the analysis. These were extracted directly from the systematic reviews when possible. Otherwise, we extracted these data from the included primary studies when there were ≤5 in the analysis or used the 5 largest studies from larger analyses to calculate the control event rate.

Appendix 10, as supplied by the authors. Appendix to: Thériault G, Limburg H, Klarenbach S, et al. Recommendations on screening for primary prevention of fragility fractures. *CMAJ* 2023. doi: 10.1503/cmaj.221219. Copyright © 2023 The Author(s) or their employer(s). To receive this resource in an accessible format, please contact us at cmajgroup@cmaj.ca.

		†The absolute effect (and its 95% CI) without treatment (i.e. baseline rate) is based on the estimated risk in the comparison group; the effect with treatment is based on applying the relative effect of the intervention (and its 95% CI) to the effect without treatment. When an OR is presented, we used the following formula recommended in the Cochrane manual: intervention risk per 1000 = 1000 x (OR x ACR / 1 – ACR + (OR x ACR))<br>a=risk of bias; b=inconsistency; c=indirectness; d=imprecision; e=Large magnitude of effect (+1)<br><br>This review did not include all possible rare adverse events caused by treatment (e.g. hypocalcemia caused by denosumab). Guidelines providing recommendations for pharmaceutical treatment may discuss this in greater detail.<br><br>**Overall severity of undesirable anticipated effects**<br><br>The increase in adverse events was seen as small (i.e. very rare SAEs with wide confidence intervals, more common but non-serious AEs.<br><br>Although level of overdiagnosis was moderate (i.e. a large portion of the screened population will be overdiagnosed) the resulting harm (anxiety, labelling) was thought to be not as severe as being overdiagnosed with cancer (i.e. does not require surgery, chemo, etc.), resulting in an overall rating of “small” for undesirable effects.	

Appendix 10, as supplied by the authors. Appendix to: Thériault G, Limburg H, Klarenbach S, et al. Recommendations on screening for primary prevention of fragility fractures. *CMAJ* 2023. doi: 10.1503/cmaj.221219. Copyright © 2023 The Author(s) or their employer(s). To receive this resource in an accessible format, please contact us at cmajgroup@cmaj.ca.

CERTAINTY OF EVIDENCE	**What is the overall certainty of the evidence of effects?**<br><br>**X** Very low (males ≥40 and females 40-65 years)<br>**X** Low (females ≥65 years)<br>o Moderate<br>oHigh<br><br>oNo included studies	JUDGEMENT – CERTAINTY<br><br>When considering the benefits and harms of screening for the "selected population" (offer-to-screen among females ≥65 years willing to independently complete a mailed fracture risk questionnaire), the overall certainty of the evidence for an effect of screening to prevent fragility fractures was Low.<br><br>When considering the benefits and harms of screening from the general population (offer-to-screen among females ≥65 years), the overall certainty of the evidence for an effect of screening to prevent fragility fractures was Low.<br><br>When considering the benefits and harms of screening for the general population (females 40-64 or males ≥65 years), the overall certainty of the evidence for an effect of screening to prevent fragility fractures was Very low.<br><br>There was no evidence for males 40-64 years (Very low)<br><br>This resulted in a combined rating of<br>Females ≥65 years = Low certainty<br>Females 40-64 years = Very low certainty<br>Males ≥40 years = Very low certainty<br><br>**SCREENING VS NO SCREENING (KQ1a)**	

Appendix 10, as supplied by the authors. Appendix to: Thériault G, Limburg H, Klarenbach S, et al. Recommendations on screening for primary prevention of fragility fractures. *CMAJ* 2023. doi: 10.1503/cmaj.221219. Copyright © 2023 The Author(s) or their employer(s). To receive this resource in an accessible format, please contact us at cmajgroup@cmaj.ca.

Outcomes for "Selected population" (offer-to-screen among females ≥65 years willing to independently complete a mailed fracture risk questionnaire).

Outcome	Certainty
Hip fractures	Moderate
Clinical fragility fractures	Moderate
~~Fracture related mortality~~	~~No evidence (very low)~~
All-cause mortality	Moderate
~~Functionality and disability~~	~~No evidence (very low)~~
Quality of life or well-being	~~Very low to~~ moderate
Serious adverse events	~~Very low (KQ1) to~~ moderate (KQ3b)
Overdiagnosis	Low
~~Discontinuation due to adverse events~~	~~Very low to moderate (KQ3b)~~
Non-serious adverse events	Low to moderate (KQ3b)

- GRADE methods usually require that the overall rating be based on the lowest certainty outcome (109).
- An overall rating of "low certainty" was chosen despite the very-low certainty found on some outcomes. This follows the GRADE methodology where certain outcomes may cease to be considered critical if they would not change the strength or direction of the recommendation. Additionally, "If there is higher quality of evidence for some critical outcomes to support a decision, then one need not rate down quality of evidence because of lower confidence in estimates of effects on other critical outcomes that support the same recommendation" (109). Rationale for the re-classification of specific outcomes are listed below:
  - Fracture related-mortality was deemed not necessary as the higher certainty outcome of all-cause mortality did not show an effect.

Appendix 10, as supplied by the authors. Appendix to: Thériault G, Limburg H, Klarenbach S, et al. Recommendations on screening for primary prevention of fragility fractures. *CMAJ* 2023. doi: 10.1503/cmaj.221219. Copyright © 2023 The Author(s) or their employer(s). To receive this resource in an accessible format, please contact us at cmajgroup@cmaj.ca.

- Functionality and disability, and Quality of life or well-being were considered not necessary due to the higher certainty outcome of hip fracture (which would impact these outcomes). Additionally, there was one analysis with moderate certainty that showed no effect on quality of life.
- The outcome of serious adverse events (KQ1) was not relevant as this was studied with greater detail and follow-up in treatment trials from KQ3.
- The outcome of Discontinuation due to adverse events was deemed not necessary due to the higher certainty outcome evidence from serious and non-serious adverse events.

- **The overall rating is the lowest of the remaining outcome certainties = Low**

Outcomes for All eligible (offer-to-screen among the general population) Females ≥65 years

Outcome	Certainty
Hip fractures	Low
Clinical fragility fractures	Low
~~Fracture related mortality~~	~~No evidence (very low)~~
All-cause mortality	Low
~~Functionality and disability~~	~~No evidence (very low)~~
~~Quality of life or well-being~~	~~No evidence (very low)~~
Serious adverse events	Moderate (KQ3b)
~~Overdiagnosis~~	~~No evidence (very low)~~
~~Discontinuation due to adverse events~~	~~Very low to moderate (KQ3b)~~
Non-serious adverse events	Low to moderate (KQ3b)

- GRADE methods usually require that the overall rating be based on the lowest certainty outcome (109).

Appendix 10, as supplied by the authors. Appendix to: Thériault G, Limburg H, Klarenbach S, et al. Recommendations on screening for primary prevention of fragility fractures. *CMAJ* 2023. doi: 10.1503/cmaj.221219. Copyright © 2023 The Author(s) or their employer(s). To receive this resource in an accessible format, please contact us at cmajgroup@cmaj.ca.

- An overall rating of “low certainty” was chosen despite the very-low certainty found on some outcomes. This follows the GRADE methodology where certain outcomes may cease to be considered critical if they would not change the strength or direction of the recommendation. Additionally, “If there is higher quality of evidence for some critical outcomes to support a decision, then one need not rate down quality of evidence because of lower confidence in estimates of effects on other critical outcomes that support the same recommendation” (109). Rationale for the re-classification of specific outcomes are listed below:
    - Fracture related-mortality was deemed not necessary as the higher certainty outcome of all-cause mortality did not show an effect.
    - Functionality and disability, and Quality of life or well-being were considered not necessary due to the higher certainty outcome of hip fracture (which would impact these outcomes). Additionally, there was one analysis with moderate certainty that showed no effect on quality of life for selected populations (see above).
    - The outcome of serious adverse events (KQ1) was not relevant as this was studied with greater detail and follow-up in treatment trials from KQ3.
    - The outcome of overdiagnosis was deemed not necessary for this population due to the higher certainty outcome evidence from the “Selected population”.
    - The outcome of Discontinuation due to adverse events was deemed not necessary due to the higher certainty outcome evidence from serious and non-serious adverse events.
- **The overall rating is the lowest of the remaining outcome certainties = Low**

Outcomes for All eligible (general population) Females 40-64 years

Outcome	Certainty
Hip fractures	Very low
Clinical fragility fractures	Very low
Fracture related mortality	No evidence (very low)
All-cause mortality	Very low to Moderate
Functionality and disability	No evidence (very low)

Appendix 10, as supplied by the authors. Appendix to: Thériault G, Limburg H, Klarenbach S, et al. Recommendations on screening for primary prevention of fragility fractures. *CMAJ* 2023. doi: 10.1503/cmaj.221219. Copyright © 2023 The Author(s) or their employer(s). To receive this resource in an accessible format, please contact us at cmajgroup@cmaj.ca.

Quality of life or well-being	Very low to Low
Serious adverse events	No evidence (very low)
Overdiagnosis	No evidence (very low)
Discontinuation due to adverse events	Very low to moderate (KQ3b)
Non-serious adverse events	Low to moderate (KQ3b)

- **The overall rating is the lowest of the outcome certainties = Very Low**

Outcomes for All eligible (general population) Males ≥65 years

Outcome	Certainty
Hip fractures	Very low
Clinical fragility fractures	No evidence (very low)
Fracture related mortality	No evidence (very low)
All-cause mortality	No evidence (very low)
Functionality and disability	No evidence (very low)
Quality of life or well-being	No evidence (very low)
Serious adverse events	Moderate (KQ3b)
Overdiagnosis	No evidence (very low)
Discontinuation due to adverse events	Very low to moderate (KQ3b)
Non-serious adverse events	Low to moderate (KQ3b)

Appendix 10, as supplied by the authors. Appendix to: Thériault G, Limburg H, Klarenbach S, et al. Recommendations on screening for primary prevention of fragility fractures. *CMAJ* 2023. doi: 10.1503/cmaj.221219. Copyright © 2023 The Author(s) or their employer(s). To receive this resource in an accessible format, please contact us at cmajgroup@cmaj.ca.

		- The overall rating is the lowest of the outcome certainties = Very Low<br><br>**Outcomes for All eligible (general population) Males 40-64 years**<br>No evidence, therefore the overall rating is Very Low<br><br>**This resulted in a combined overall rating of:**<br>- **The overall certainty of the evidence for all females (selected or general population) ≥65 years = Low**<br>- **The overall certainty of the evidence for all females 40-64 years = Very low**<br>- **The overall certainty of the evidence for all males ≥40 years = Very low**	
VALUES	**Is there important uncertainty about or variability in how much people value the main outcomes?**<br><br>**X** Important uncertainty or variability<br>○Possibly important uncertainty or variability<br>○Probably no important uncertainty or variability	**KQ4: For patients ≥40 years, what is the acceptability (i.e., positive attitudes, intentions, willingness, uptake) of screening and/or initiating treatment to prevent fragility fractures when considering the possible benefits and harms from screening and/or treatment?**<br><br>**JUDGEMENT – VALUES**<br><br>**Younger (lower risk) females (age 50-65 years) have a high willingness to be screened.**<br><br>**There is large heterogeneity in the level of risk at which patients may be accepting of initiating treatment, and treatment effects appear to be overestimated by the patients.**<br><br>**There is low to moderate certainty in the evidence on patient acceptability of treatment indicating that a substantial proportion of people (possibly >50%) may not value the benefits as more important than the harms. The evidence is indirect to screening since most studies examined treatment decisions**	

Appendix 10, as supplied by the authors. Appendix to: Thériault G, Limburg H, Klarenbach S, et al. Recommendations on screening for primary prevention of fragility fractures. *CMAJ* 2023. doi: 10.1503/cmaj.221219. Copyright © 2023 The Author(s) or their employer(s). To receive this resource in an accessible format, please contact us at cmajgroup@cmaj.ca.

○No important uncertainty or variability

Table 7.1: Patient acceptability of screening and/or initiating treatment

Studies; sample size	Certainty*	What does the evidence say?
**Acceptance of screening**		
Females 50-65 y<br>1 observational; 258<br>(110)	Low[a-d]	Females aged 50-65 years (low risk) may have a high intention to be screened, and this intention may not be changed after reading a 1-page decision support sheet (1 study, n=258) (110).
**Acceptance of treatment with information**		
Adults (predominantly female) ≥50 y, mean 63-72 y, 2 observational, 2 RCT; 980<br>(111–114)	Low[a,c]	Patients' preference for treatment vs. no treatment may be highly variable (2 studies, n=287) (111,112). After receiving information on their personal fracture risk, relatively few (19 to 39%) patients may be willing to accept treatment (2 studies, n=593) (113,114).
**Acceptance of treatment with decision aids**		
Postmenopausal females ≥45 y, mean 62-69 y<br>4 observational (5 reports); ~324<br>(115–119)	Moderate[a,d]	Few (5-20%) postmenopausal females with osteoporosis or osteopenia who read decisions aids and are aware of their fracture risk are willing to initiate treatment (2 studies, n~240) (115–117). Somewhat more (41-44%) may be willing to start treatment when the decision aid is used during a clinical encounter or when they have had a previous fracture or are at higher fracture risk (32-45%; 1 study, n=208) (2 studies, n=84) (118,119). Overall, a minority of postmenopausal females at increased risk for fracture may accept treatment.
**Minimum acceptable benefit of treatment**		
Adults ≥50 y, mean 60-72 y, 3 observational; 741<br>(111,112,120)	Low[a,c]	About two-thirds (64%) of adults ≥50 years may have overly optimistic views of the benefits of treatment (1 study, n=354) (120); these views may be highly variable (3 studies, n=741) (111,112,120) Patients may require a reduction of 20 to 200 fractures per 1000 to consider 10 y of bisphosphonate treatment acceptable (1 study, n=354) (120).
**Level of risk at which treatment is acceptable**		
Adults (predominantly female), ≥45 y, 6 observational; 1091<br>(111,113–115,119,121)	Low[a,c]	Among adults ≥45 years (97% female; aware of personal risk) there is large heterogeneity in the level of risk at which treatment would be considered (111,113–115,119,121). Many (19 to 51%) are willing to accept treatment at low levels of fracture risk (5 to 20%), but a large proportion (44 to 68%) of high-risk females (≥3% hip or ≥20% osteoporotic fracture risk; ≥30% in one study) would choose not to be treated (3 studies, n=378) (113,115,119).

RCT=randomized controlled trial; y=years

Appendix 10, as supplied by the authors. Appendix to: Thériault G, Limburg H, Klarenbach S, et al. Recommendations on screening for primary prevention of fragility fractures. *CMAJ* 2023. doi: 10.1503/cmaj.221219. Copyright © 2023 The Author(s) or their employer(s). To receive this resource in an accessible format, please contact us at cmajgroup@cmaj.ca.

<table>
<tr><td rowspan="1"></td><td></td><td>*When our assessment of the certainty of evidence fell between levels, we assigned the level that best represented our actual
a=risk of bias; b=inconsistency; c=indirectness; d=imprecision; e=Large magnitude of effect (+1)</td><td></td></tr>
<tr><td>BALANCE OF EFFECTS</td><td>Does the balance between desirable and undesirable effects favor the intervention or the comparison?

X Favors the comparison (males ≥40 and females 40-65 years)
○ Probably favors the comparison
○Does not favor either the intervention or the comparison
X Probably</td><td>JUDGEMENT

The balance of benefits and harms may favour the intervention for females ≥65 years
The balance of benefits and harms favours the comparison for females 40-64 and males ≥40 years

<table>
<tr><th></th><th>Females ≥65 years (selected population)

Overall certainty: Low</th><th>Females ≥65 years (general population)

Overall certainty: Low</th><th>Females 40-64 years (general population)

Overall certainty: Very Low</th><th>Males ≥65 years

Overall certainty: Very Low</th><th>Males 40-64 years

Overall certainty: Very Low</th></tr>
<tr><td>Population</td><td>Females ≥65 years (SCOOP, SALT and ROSE RCTs + Kern CCT for hip fractures only) (24–27)[11]</td><td>Females 68-80 years (ROSE RCT) (24)</td><td>Females 45-54 years (APOSS (Barr) RCT) (23)</td><td>Males ≥65 years (Kern CCT) (27)</td><td>No evidence</td></tr>
<tr><td>Hip fractures</td><td>Probably reduces</td><td>May not reduce</td><td>Very uncertain</td><td>Very uncertain</td><td>No evidence</td></tr>
</table></td><td>Notes: Screening RCTs had a higher rate of hip fracture and lower rate of MOF and clinical fragility fracture than Canadian data (see footnote 11). Treatment RCTs had lower hip and MOF rates than Canadian data (see footnotes 12 -14)

The frequency of screening was not examined in this analysis, but may be a consideration for implementation

- Osteoporosis Canada recommends repeating BMD in 1–</td></tr>
</table>

[11] This is a population with a mean risk of hip and MOF of 3.1% and 8.4% respectively (higher for hip and lower for MOF than Canadian females (2.0% and 16.8% respectively).

Appendix 10, as supplied by the authors. Appendix to: Thériault G, Limburg H, Klarenbach S, et al. Recommendations on screening for primary prevention of fragility fractures. *CMAJ* 2023. doi: 10.1503/cmaj.221219. Copyright © 2023 The Author(s) or their employer(s). To receive this resource in an accessible format, please contact us at cmajgroup@cmaj.ca.

	favors the intervention (females ≥65 years)<br>○Favors the intervention<br><br>○Varies<br>○Don't know		Study data: 6.2 (2.8-9.0) fewer per 1000<br>General population: 4.0 (1.8-5.8) fewer per 1,000<br><br>Moderate to high certainty	Study data: 0.3 fewer (4.2 fewer to 3.9 more) per 1000<br>General population: 0.2 fewer (2.4 fewer to 2.2 more) fewer per 1,000<br>Low certainty				3 years to reassess risk (4).<br><br>- USPSTF states "limited evidence from 2 good-quality studies found no benefit in predicting fractures from repeating bone measurement testing 4 to 8 years after initial screening." (6)
			Indirect evidence[12]:<br>Bisphosphonates **may reduce** 2.9 (study data) and 5.3 (general population) fewer in 1,000.<br><br>Low certainty			Indirect evidence[13]:<br><br>Zoledronic acid **may not reduce**<br>Study data: 2.2 more (1.6 fewer to 42.0 more) per 1,000<br><br>General population: 16.7 more (12.9 fewer to 256.0 more) per 1,000<br><br>Low certainty		

[12] This is a population with a mean risk of hip and clinical fragility fracture of 1.1% and 5.8% respectively (lower for hip and MOF than Canadian females (2.0% and 20.2% respectively)).
[13] This is a population with a mean risk of hip of 0.4% (lower than Canadian males (3.3%)).

Appendix 10, as supplied by the authors. Appendix to: Thériault G, Limburg H, Klarenbach S, et al. Recommendations on screening for primary prevention of fragility fractures. *CMAJ* 2023. doi: 10.1503/cmaj.221219. Copyright © 2023 The Author(s) or their employer(s). To receive this resource in an accessible format, please contact us at cmajgroup@cmaj.ca.

Clinical fragility fractures	**Probably reduces**<br><br>Study data: 5.9 (0.8-10.9) fewer per 1000<br>General population: 11.8 (1.7-21.8) fewer per 1,000<br><br>Moderate to high certainty	**May not reduce**<br><br>Study data: 1.0 fewer (8.0 fewer to 6.0 more) per 1000<br>General population: 1.7 fewer (13.4 fewer to 10.1 more) per 1,000<br><br>Low certainty	**Very uncertain**	**No evidence**	**No evidence**
	Indirect evidence[14]<br>Bisphosphonates **probably reduces** 11.1 (study data) and 33.6 (general population) fewer in 1,000 and denosumab **probably reduces** 12.2 (study data) and 51.5 (general population) fewer in 1,000<br><br>Moderate certainty			Indirect evidence[15]**:**<br>Zoledronic acid **may not reduce**<br><br>Study data: 7.7 fewer (14.2 fewer to 9.5 more) per 1,000<br><br>General population: 42.3 fewer (81.0 fewer to 48.0 more) per 1,000<br><br>Low certainty	

[14] This is a population with a mean risk of clinical fragility fracture of 4.2% (lower than for Canadian females (20.2%)).
[15] This is a population with a mean risk of clinical fragility fracture of 1.0% (lower than Canadian males (6.3%)).

Appendix 10, as supplied by the authors. Appendix to: Thériault G, Limburg H, Klarenbach S, et al. Recommendations on screening for primary prevention of fragility fractures. *CMAJ* 2023. doi: 10.1503/cmaj.221219. Copyright © 2023 The Author(s) or their employer(s). To receive this resource in an accessible format, please contact us at cmajgroup@cmaj.ca.

All-cause mortality	**Probably does not reduce**<br><br>No difference in 1,000 (study and general population)<br><br>Moderate certainty	**May not reduce**<br><br>Study data:<br><br>3.5 fewer (9.4 fewer to 3.5 more) in 1000.<br><br>General population:<br><br>1.7 fewer (4.6 fewer to 1.7 more) in 1,000<br><br>Low certainty	**Very uncertain to may not reduce**<br><br>Study data:<br><br>0.3 fewer (9.2 fewer to 11.6 more) per 1,000<br><br>General population:<br><br>No difference (0.8 fewer to 1.1 more) in 1,000<br><br>Very low to moderate certainty	**No evidence**	**No evidence**
Quality of life/health related quality of life	**May be little to no difference**<br><br>Low to moderate certainty	**No evidence**	**Very uncertain**	**No evidence**	**No evidence**

Paradigmatic situations in which a strong recommendation may be warranted despite low or very

Appendix 10, as supplied by the authors. Appendix to: Thériault G, Limburg H, Klarenbach S, et al. Recommendations on screening for primary prevention of fragility fractures. *CMAJ* 2023. doi: 10.1503/cmaj.221219. Copyright © 2023 The Author(s) or their employer(s). To receive this resource in an accessible format, please contact us at cmajgroup@cmaj.ca.

Overdiagnosis	SCOOP: 11.8% (using hip fracture risk) (26); SALT: 19.8% (using MOF risk) (25)<br><br>Among acceptors = 24.1 (SCOOP) (26);<br><br>Low certainty	**No evidence**	**No evidence**	**No evidence**	**No evidence**
Serious adverse events	**Very uncertain** (SCOOP) (26);				
	<u>Indirect evidence</u>[16]<br><br>Bisphosphonates (as a class) and alendronate **may increase rare but serious harms of subtrochanteric atypical femoral fracture and osteonecrosis of the jaw**<br><br>(AFF = 0.06 to 1.1 more per 1,000)<br><br>(ONJ = 0.22 to 43 more per 1,000)<br><br>Low certainty				
	**No direct evidence**				

low confidence in effect estimates (122)

1. When low quality evidence suggests benefit in a life-threatening situation

2. When low quality evidence suggests benefit and high quality evidence suggests harm or a very high cost

3. When low quality evidence suggests equivalence of two alternatives, but high quality evidence of less harm for one of the competing alternatives

4. When high quality evidence suggests equivalence of two alternatives and low quality evidence suggests harm in one alternative

[16] Majority were older females, some older males (Alendronate)

Appendix 10, as supplied by the authors. Appendix to: Thériault G, Limburg H, Klarenbach S, et al. Recommendations on screening for primary prevention of fragility fractures. *CMAJ* 2023. doi: 10.1503/cmaj.221219. Copyright © 2023 The Author(s) or their employer(s). To receive this resource in an accessible format, please contact us at cmajgroup@cmaj.ca.

<table>
<tr>
<td></td>
<td></td>
<td>
<table>
<tr>
<td>Non-serious adverse events</td>
<td>Indirect evidence[17]

Alendronate and denosumab probably increase non-serious GI adverse events 16.3 to 64.5 more respectively per 1,000.

(moderate certainty).

Zoledronic acid probably increases pyrexia, headache, influenza-like symptoms, arthritis and arthralgia, myalgia. 422.8 more per 1,000

(low certainty)

Denosumab probably increases rash/eczema 15.8 more per 1,000 and infections 1.8 more per 1,000

(moderate certainty)</td>
</tr>
</table>

There is low certainty evidence that screening with a clinical risk assessment tool (i.e. FRAX) followed by a BMD scan (where indicated*) and re-calculation of FRAX with BMD risk, may reduce hip and clinical fragility fractures in females ≥65 years

There is low certainty evidence that screening may result in 11.8-19.8% being overdiagnosed as “at risk” and may increase rare but serious harms (i.e. osteonecrosis of the jaw, atypical femoral fractures) and probably increases non-serious adverse events (e.g. gastrointestinal (reflux, nausea), headache, influenza-like symptoms, rash)

BMD-first vs Risk assessment-first screening
- Direct evidence from a trial comparing SCORE or SOF + BMD vs BMD alone was very uncertain (29)
- SCOOP, SALT and ROSE all used risk assessment-first (FRAX+/-BMD) screening (24–26)
- FRAX without BMD may be well-calibrated to predict 10-year hip fractures and is probably well-calibrated to predict 10-year clinical fragility fractures
</td>
<td>5. When high quality evidence suggests modest benefits and low/very low quality evidence suggests possibility of catastrophic harm

Additional TF criteria:

“When there is an absence of evidence to provide confidence that there is benefit from implementing a new prevention service or when a conclusion of possible benefit requires a high level of speculation on linkages of uncertain evidence, but there is high certainty that some patients would be harmed or scarce health care resources expended, the task force may make a strong recommendation against service implementation” (123).</td>
</tr>
</table>

[17] Majority were older females, some older males (Zoledronic acid)

Appendix 10, as supplied by the authors. Appendix to: Thériault G, Limburg H, Klarenbach S, et al. Recommendations on screening for primary prevention of fragility fractures. *CMAJ* 2023. doi: 10.1503/cmaj.221219. Copyright © 2023 The Author(s) or their employer(s). To receive this resource in an accessible format, please contact us at cmajgroup@cmaj.ca.

- FRAX with BMD may perform poorly to predict 10-year hip fractures but is probably well calibrated to predict 10-year clinical fragility fractures
- CAROC[18] may be adequately calibrated to predict category of clinical fragility fracture risk. However, no screening trial has been conducted with CAROC.

RECOMMENDATIONS

FEMALES ≥65 YEARS

In females 65 years or older, we recommend screening with the Canadian FRAX risk assessment tool (without BMD) and using the 10-year absolute risk of Major Osteoporotic Fracture to facilitate shared decision-making about the possible benefits and harms of treatment. If the patient is considering preventive treatment, we recommend refining fracture risk by adding the BMD value in FRAX. (Conditional recommendation, low certainty evidence)

Considerations for implementation

- *The frequency of screening was not specifically examined as a key question for this analysis, but may be a consideration for implementation*
    - *No RCTs of different screening intervals were found during the SR for KQ1*
    - *We did not perform a systematic review of observational studies of different screening intervals*
    - *Evidence from an environmental scan of cohort studies found that repeated BMD scans at 3-8 years did not improve fracture risk prediction (females aged ≥50 years) (147-149)*
    - *A systematic review performed by the USPSTF in 2018 found that "Some observational and modeling studies have suggested screening intervals based on age, baseline BMD, and calculated projected time to transition to osteoporosis. However, limited evidence from 2 good-quality studies found no benefit in predicting fractures from repeating bone measurement testing 4 to 8 years after initial screening."* (5)

Rationale:

- *The small benefit (reduction in hip and clinical fragility fractures) outweighs the moderate risk of overdiagnosis, small increased risk of rare serious harms (osteonecrosis of the jaw, atypical femoral fractures) and small increase in non-serious AEs (e.g. GI AEs, arthralgia, influenza-like symptoms)*

[18] CAROC is a semi-quantitative method for estimating 10-year absolute risk of a MOF in postmenopausal females and males over age 50. Three zones (low: < 10%, moderate: 10-20, high: > 20%). Based on age, BMD, sex, previous fracture after age 40 and glucocorticoid use (142).

Appendix 10, as supplied by the authors. Appendix to: Thériault G, Limburg H, Klarenbach S, et al. Recommendations on screening for primary prevention of fragility fractures. *CMAJ* 2023. doi: 10.1503/cmaj.221219. Copyright © 2023 The Author(s) or their employer(s). To receive this resource in an accessible format, please contact us at cmajgroup@cmaj.ca.

- *The recommendation for a risk assessment-first screening process is based on the methods used in the trials (i.e. FRAX (without BMD) followed by BMD assessment if indicated and recalculation of FRAX with BMD)*
    - *Risk assessment-first screening limits the number of women who require BMD scans to only those at increased risk (based on FRAX results and shared decision making)*
    - *Additionally, evidence from KQ2 showed that Canadian FRAX (without BMD) may be well-calibrated to predict 10-year hip or clinical fragility fracture*
    - *The CAROC tool is also commonly used but was not recommended as part of the risk assessment-first screening process as it does not allow risk calculation without BMD*
- *The trials used a threshold for access to BMD and treatment, however, the WG recommends shared decision making at each step*
- *This recommendation is conditional based on the low certainty of the evidence, differences in benefit seen in "select" vs general populations and the variable patient values and preferences*

<u>FEMALES 40-65 YEARS</u>
In females 40-64 years, we recommend not screening. (Strong recommendation, very low certainty evidence)

Rationale:
- *The evidence about all eligible / offer-to-screen populations (females 45-54 years) is very uncertain.*
- *It is uncertain whether females 40-64 years would benefit from screening. However, there is high certainty that some patients may be harmed (e.g. overdiagnosis) and costs would increase*

- *This recommendation follows the TF criteria for making a strong recommendation based on very low certainty evidence:*
    - *"When there is an absence of evidence to provide confidence that there is benefit from implementing a new prevention service or when a conclusion of possible benefit requires a high level of speculation on linkages of uncertain evidence, but there is high certainty that some patients would be harmed or scarce health care resources expended, the task force may make a strong recommendation against service implementation" (see notes)*

<u>MALES ≥40 YEARS</u>
In males ≥40 years we recommend not screening. (Strong recommendation, very low certainty evidence)

Rationale:

Appendix 10, as supplied by the authors. Appendix to: Thériault G, Limburg H, Klarenbach S, et al. Recommendations on screening for primary prevention of fragility fractures. *CMAJ* 2023. doi: 10.1503/cmaj.221219. Copyright © 2023 The Author(s) or their employer(s). To receive this resource in an accessible format, please contact us at cmajgroup@cmaj.ca.

		- *The evidence about offer-to-screen in selected populations (males ≥65 years) is very uncertain.*<br>- *There was no evidence for males 40-64 years*<br>- *It is uncertain whether males ≥40 would benefit from screening. However, there is high certainty that some patients may be harmed (e.g. overdiagnosis) and costs would increase*<br><br>• *This recommendation follows the TF criteria for making a strong recommendation based on very low certainty evidence:*<br>– *"When there is an absence of evidence to provide confidence that there is benefit from implementing a new prevention service or when a conclusion of possible benefit requires a high level of speculation on linkages of uncertain evidence, but there is high certainty that some patients would be harmed or scarce health care resources expended, the task force may make a strong recommendation against service implementation" (see notes)*	

Appendix 10, as supplied by the authors. Appendix to: Thériault G, Limburg H, Klarenbach S, et al. Recommendations on screening for primary prevention of fragility fractures. *CMAJ* 2023. doi: 10.1503/cmaj.221219. Copyright © 2023 The Author(s) or their employer(s). To receive this resource in an accessible format, please contact us at cmajgroup@cmaj.ca.

.RESOURCES REQUIRED	**How large are the resource requirements (costs)?**<br><br>○Large costs<br>○Moderate costs<br>○Negligible costs and savings<br>**X** Moderate savings<br>○Large savings<br><br>○Varies<br>○Don't know	A systematic cost-effectiveness analysis was not conducted as part of the systematic review.<br><br>**JUDGEMENT – RESOURCES REQUIRED**<br><br>**There would be moderate cost savings with a strong recommendation against screening for men and younger females. Canadian data (2009) indicates that 35% of females 40-64 years and 10% of males ≥40 years (20% of males ≥65 years) self-reported receiving BMD scans (140). Additionally, there should be savings if the Task Force recommends for risk assessment-first screening among females ≥65 years (e.g. some BMD scans could be avoided based on FRAX score and shared decision making).**<br><br>**There may be additional costs if recommending for screening females ≥65 years as Canadian self-reported data (2009) found that 32% of females in this age group had never received a BMD test (140). However, as a conditional recommendation this would depend on the results of shared decision making.**<br><br>**A cost-effectiveness analysis of the SCOOP trial (5 year follow-up) showed that screening prevented fractures at a cost of £4,478 and £7,694 per fracture for MOF and hip fractures, respectively. It also improved QALY at an average incremental cost of £2,772 (124).**<br><br>**A Markov model of the SCOOP trial estimated long-term (mean=14 year) outcomes for screened vs unscreened individuals. Screening of 1,000 patients saved 9 hip fractures and 20 non-hip fractures. The screening arm also saved £286 in comparison with usual management arm (125).**	Estimated cost for BMD (DEXA) in Ontario (126):<br>(Billing schedule)<br>$47.75 for one site<br>$61.55 for two sites (hip and spine)<br>Associated costs<br>- Radiation technologist<br>- Radiologist<br>- Family doctor<br>- Medication<br><br>There may be significant patient costs of medication as some provincial drug coverage only provides restricted access to certain medications (e.g. denosumab, zoledronic acid).<br>Canada >=65 years medication coverage (127,128)<br>Alendronate (CAD$122-$182/ year) or Risedronate (CAD$130-$600 /year): Coverage varies by province (open access or restricted access)<br>Zoledronic acid (CAD$335/year):

Appendix 10, as supplied by the authors. Appendix to: Thériault G, Limburg H, Klarenbach S, et al. Recommendations on screening for primary prevention of fragility fractures. *CMAJ* 2023. doi: 10.1503/cmaj.221219. Copyright © 2023 The Author(s) or their employer(s). To receive this resource in an accessible format, please contact us at cmajgroup@cmaj.ca.

		**COST-EFFECTIVENESS ANALYSES OF THE SCOOP STUDY:**<br><br>2. Turner DA, Khioe RFS, Shepstone L, Lenaghan E, Cooper C, Gittoes N, et al. The Cost-Effectiveness of Screening in the Community to Reduce Osteoporotic Fractures in Older Women in the UK: Economic Evaluation of the SCOOP Study. J Bone Miner Res 2018 May;33(5):845-851. (124)<br>Perspective: A "within trial" economic analysis was undertaken on an "intention-to-treat" basis from the perspective of a national health payer, the UK National Health Service (NHS).<br>Methods: **Five-year time horizon** for cost (2013/14) per quality adjusted life year (QALY), osteoporosis-related fracture prevented (hip, vertebral, wrist), and hip fracture prevented. Point estimates from RCT for hip and osteoporotic fractures used. QALYs estimated (from AUC) from EQ-5D scores across all data points (at baseline, 6, 12 and annually; major imputation requited for 36% participants); tariffs from UK population from 10-year duration TTOs. Screening resources (BMD/DXA scans, calculation and clinical review of fracture risk, GP consultation; identifying women) were recorded as part of the SCOOP study and costed per study data or using NHS Reference costs 2013 to 2014 or unit costs of health and social care 2014. Resources and costs associated with fracture-related health care contacts (inpatient [elective or non-elective]; length of stay; short stays; and excess bed days), outpatient [by specialty & first or follow-up appointments & procedure costs], and accident and emergency (A&E) datasets using Health Resource Group codes were linked to NHS reference costs. Medication data were available for anti-osteoporosis medicines for the full period of follow-up for all study participants and were costed using prices from the British National Formulary. Sensitivity analysis using complete case analysis with patients completing all EQ5D data.<br>Summary: The screening arm had an average incremental **QALY gain of 0.0237** (95% CI -0.0034 to 0.00508) for the 5-year follow-up. The **cost per QALY gained was £2,772**. Cost-effectiveness acceptability curves indicated a 93% probability of the intervention being cost-effective at a threshold cost/QALY of ≤£20,000. The intervention arm prevented fractures at a cost of **£4,478 and £7,694 per fracture for osteoporosis-related hip fractures**, respectively. Complete case analysis had 2-3 times higher cost/QALYs (ICERs).<br><br>**Table 8.1 Cost-effectiveness results for cases vs controls in SCOOP** (130)	Coverage varies by province (restricted access or no coverage)<br>Denosumab (CAD$716/year) (restricted access in all provinces) (127,128)<br><br>In Ontario, the total cost of treatment for all hip fractures occurring in 2015/16 (in adults aged 66+) was estimated to be $255,773,130 based on direct utilization costs for the episode of care. The median cost per single episode of care was $25,015 for direct utilization costs (129).<br><br>Utilization of BMD varies by sex with 8.15% of females vs 4.81% of males aged 40+ reporting a BMD scan in 2015 (10).<br><br>22.9% of eligible adults (aged 68-70) in Ontario reported

	Usual management	Screening
**Mean costs, per patient (£)**		
Inpatient	531	482
A & E	162	160

Appendix 10, as supplied by the authors. Appendix to: Thériault G, Limburg H, Klarenbach S, et al. Recommendations on screening for primary prevention of fragility fractures. *CMAJ* 2023. doi: 10.1503/cmaj.221219. Copyright © 2023 The Author(s) or their employer(s). To receive this resource in an accessible format, please contact us at cmajgroup@cmaj.ca.

<table>
<tr>
<td></td>
<td></td>
<td>
<table>
<tr><td>Outpatient</td><td>191</td><td>201</td></tr>
<tr><td>Medicines</td><td>8</td><td>13</td></tr>
<tr><td>Non-SCOOP DXA</td><td>9</td><td>9</td></tr>
<tr><td>Cost of SCOOP interventions</td><td>-</td><td>104</td></tr>
<tr><td>Total costs</td><td>900</td><td>968</td></tr>
</table>

2. Söreskog E, Borgström F, Shepstone L, Clarke S, Cooper C, Harvey I, et al. Long-term cost-effectiveness of screening for fracture risk in a UK primary care setting: the SCOOP study. Osteoporos Int 2020 Aug;31(8):1499-1506. (125)
<u>Perspective</u>: Cost-effectiveness based on the NICE’s willingness-to-pay (WTP) threshold for recommending new treatment of £20,000–30,000 per QALY gained
<u>Methods:</u> Health economic Markov model (6-month cycles and 8 health states: wrist fracture, vertebral fracture, hip fracture, other osteoporotic fracture, post-vertebral fracture, post-hip fracture, dead and well [i.e. without fracture]; transition probabilities NR but cited studies) following a cohort from study participation until death or an age of 100 years (mean 14 year time horizon). Outcomes were cost per quality adjusted life year (QALY) and life year. Point estimates from RCT for various discrete fractures used (risk reductions at 5 years were used without any assumptions of longer term effects). Resource use and costs for drugs, administration, screening intervention as per above analysis from SCOOP RCT. Clinical costs (hospitalizations, nursing homes, outpatient) in the first and subsequent years after fracture were derived from two retrospective cohort studies that estimated fracture costs in postmenopausal females in the UK. Quality of life weights for each health state, in the first year after fracture and subsequent years (for hip and vertebral), respectively, were derived from the International Costs and Utilities Related to Osteoporotic fractures Study (ICUROS). Annual mortality in the general female population was obtained from the Office for National Statistics dataset. The relative risks of death in patients who had sustained a fracture compared with the general population were derived from a study by Jönsson et al. In agreement with previous health economic studies of osteoporotic treatments it was assumed that 30% of the excess mortality after a hip, vertebral, wrist and other osteoporotic fracture was related to the fracture event. Probabilistic and deterministic sensitivity analyses including if no effect on non-hip fractures and age.
<u>Summary:</u> Screening of 1000 patients saved 9 hip fractures and 20 non-hip fractures over the remaining lifetime (mean 14 years) compared with usual management. Per patient, the screening arm saved £286 and gained 0.015 QALYs and 0.002 life years in comparison with the usual management arm. 97% probability of cost-effective at
</td>
<td>ever being screened in 2017/18 (129).

13.4% of BMD scans in Ontario were performed on “low risk” adults aged ≥40 years in 2017/18 (129).</td>
</tr>
</table>

Appendix 10, as supplied by the authors. Appendix to: Thériault G, Limburg H, Klarenbach S, et al. Recommendations on screening for primary prevention of fragility fractures. *CMAJ* 2023. doi: 10.1503/cmaj.221219. Copyright © 2023 The Author(s) or their employer(s). To receive this resource in an accessible format, please contact us at cmajgroup@cmaj.ca.

WTP £20,000 and 98% at £30,000; cost-saving in 87% of simulations. Deterministic analyses all indicated cost-savings, except for at age 71 where screening became cost-neutral.

Table 8.2 Long-term cost-effectiveness results (Markov model) (125)

	Usual management	Screening	Screening vs. usual management
Mean costs, per patient (£)			
Hospitalisations	3059	2934	− 125
Nursing home	6056	5645	− 410
Outpatient	378	363	− 15
Total morbidity cost	9493	8942	− 551
Drugs	12	43	31
Treatment management	92	326	234
Total intervention cost	104	369	265
**Total cost**	**9596**	**9310**	**−286**

Appendix 10, as supplied by the authors. Appendix to: Thériault G, Limburg H, Klarenbach S, et al. Recommendations on screening for primary prevention of fragility fractures. *CMAJ* 2023. doi: 10.1503/cmaj.221219. Copyright © 2023 The Author(s) or their employer(s). To receive this resource in an accessible format, please contact us at cmajgroup@cmaj.ca.

CERTAINTY OF EVIDENCE OF REQUIRED RESOURCES	**What is the certainty of the evidence of resource requirements (costs)?**<br><br>○ Very low<br>**X** Low<br>○ Moderate<br>○High<br><br>○No included studies	A systematic cost-effectiveness analysis was not conducted as part of the systematic review.<br><br>**JUDGEMENT – CERTAINTY OF EVIDENCE OF RESOURCE REQUIREMENTS**<br><br>**There are serious limitations due to indirectness and risk of bias with the Turner et al., 2018 study.**<br><br>**There are serious concerns due to indirectness and some concerns with risk of bias with the Soreskog et al., 2020 study.**<br><br>**Overall for selected population, there is low certainty for being highly cost-effective (ICERs low) or cost-saving to the healthcare system (indirectness and risk of bias), but moderate certainty for likely meeting typical cost-effectiveness thresholds.**<br><br>2. The Cost-Effectiveness of Screening in the Community to Reduce Osteoporotic Fractures in Older Females in the UK: Economic Evaluation of the SCOOP Study (124)<br><br>Limitations:<br>• No sensitivity for uncertainty in hip fracture reductions (e.g. 95% CI) or lack of any effect on other fractures<br>• Large amount of missing EQ5D data. Missing cases had statistically significantly lower baseline EQ-5D, more incident fractures and higher fracture related healthcare costs. More missing EQ5D data possible for worse cases if fractured during data collection. Data collected q 6-12 months so acute changes from fractures not captured. Complete case analysis had 2-3 times higher cost/QALYs.<br>• Short time horizon and lack of effects from fractures on longer term and/or more reductions in fractures that may occur<br>• Estimates may be conservative from healthier sample (50% fewer deaths, more educated, higher SES) and some costs related to RCT (£44/enrolled for identifying pts)	Note: The cost-effectiveness threshold suggested by CADTH is $50,000 (130).

Appendix 10, as supplied by the authors. Appendix to: Thériault G, Limburg H, Klarenbach S, et al. Recommendations on screening for primary prevention of fragility fractures. *CMAJ* 2023. doi: 10.1503/cmaj.221219. Copyright © 2023 The Author(s) or their employer(s). To receive this resource in an accessible format, please contact us at cmajgroup@cmaj.ca.

		• No patient or societal costs (e.g. family carers) accounted for (if desiring societal perspective).<br>• Not costed for routine primary care contacts (e.g. for monitoring patients on meds or treating AEs) or admissions to nursing homes (may underestimate savings from fewer fractures; # admissions NR in publications)<br><br>Serious indirectness: Use of all vertebral fractures, but little effect in this study. Recruitment methods and education of GPs not typical but effect on the outcomes is unclear. Short-term time horizon. Does not account for possible larger reduction in non-hip fractures seen in other RCTs and our meta-analysis (which may improve cost-effectiveness).<br>Serious ROB from large missing data for utilities (for ICER on QALYs), not full spectrum of costs accounted for, and contamination of control groups possibly impacting risk reductions.<br><br>Note: Indirect to use of clinical FRAX only (number treated may differ) or using FRAX+BMD treatment thresholds that are not age dependent.<br><br>2. Söreskog E, Borgström F, Shepstone L, Clarke S, Cooper C, Harvey I, et al. Long-term cost-effectiveness of screening for fracture risk in a UK primary care setting: the SCOOP study. Osteoporos Int 2020 Aug;31(8):1499-1506. (125)<br><br>Limitations:<br>• QALYs using age and gender matched general UK population despite outcome data in a different population. Assuming one consistent utility score for all non-fractured states and minimum 6-month period for fracture states (some utilities may change before this).<br>• Quality of data used for transition probabilities unknown ("valid model").<br>• No sensitivity for uncertainty in hip fracture reductions.<br>• Not costed for routine primary care contacts (e.g. for monitoring patients on meds or treating AEs)<br>• No patient or societal costs (e.g. family carers) accounted for.<br><br>Serious indirectness: Indirect sources for utilities and transitions. Indirect from use of all vertebral fractures, but effects minimal. Recruitment methods and education of GPs not typical but effect on the outcomes is unclear. Does not account for possible larger reduction in non-hip fractures seen in other RCTs and our meta-analysis (which may improve cost-effectiveness).	

Appendix 10, as supplied by the authors. Appendix to: Thériault G, Limburg H, Klarenbach S, et al. Recommendations on screening for primary prevention of fragility fractures. *CMAJ* 2023. doi: 10.1503/cmaj.221219. Copyright © 2023 The Author(s) or their employer(s). To receive this resource in an accessible format, please contact us at cmajgroup@cmaj.ca.

		Some concern about ROB: from not full spectrum of costs accounted for, and contamination of control groups possibly impacting risk reductions.<br><br>Studies quite consistent, but overlap in data so not too surprising.<br><br>**Overall for selected population, would rate at low certainty for being highly cost-effective (ICERs low) or cost-saving to the healthcare system (indirectness and risk of bias), but moderate for likely meeting typical CE thresholds.**<br><br>Please see Cost-effectiveness analysis section for the full assessment	
COST EFFECTIVENESS	**Does the cost-effectiveness of the intervention favor the intervention or the comparison?**<br><br>○Favors the comparison<br>○Probably favors the comparison<br>○Does not favor either the intervention or the comparison<br>**X** Probably	**JUDGEMENT – COST-EFFECTIVENESS**<br><br>**Based on the two studies of SCOOP, it appears to favour screening to prevent fragility fractures (cost-saving to the health-care system). However, the certainty for this conclusion is low and is based only on one RCT.**<br><br>**When considering whether the cost to the healthcare system would meet typical cost-effectiveness thresholds the certainty was moderate.**	

Appendix 10, as supplied by the authors. Appendix to: Thériault G, Limburg H, Klarenbach S, et al. Recommendations on screening for primary prevention of fragility fractures. *CMAJ* 2023. doi: 10.1503/cmaj.221219. Copyright © 2023 The Author(s) or their employer(s). To receive this resource in an accessible format, please contact us at cmajgroup@cmaj.ca.

	favors the intervention<br>○Favors the intervention<br><br>○Varies<br>○No included studies		

Appendix 10, as supplied by the authors. Appendix to: Thériault G, Limburg H, Klarenbach S, et al. Recommendations on screening for primary prevention of fragility fractures. *CMAJ* 2023. doi: 10.1503/cmaj.221219. Copyright © 2023 The Author(s) or their employer(s). To receive this resource in an accessible format, please contact us at cmajgroup@cmaj.ca.

EQUITY	What would be the impact on health equity?		
	○Reduced<br>○Probably reduced<br>○Probably no impact<br>**X** Probably increased<br>○Increased<br><br>○ Varies<br>○ Don't know	**JUDGEMENT – EQUITY**<br><br>**A recommendation for risk assessment-first screening (females ≥65 years) may increase equity where access to BMD is limited. Risk assessment tools may help by removing unnecessary BMD for individuals at lower risk and increasing availability to those at the highest risk. However, it may decrease equity if the risk assessment tool is not calibrated for particular ethnic groups in Canada (133).**<br><br>**A recommendation against screening males (≥40 years) may increase equity, where access to BMD is limited, by removing those without multiple risk factors from BMD.**<br><br>**A recommendation against screening females (40-64 years) may increase equity where access to BMD is limited, by removing unnecessary BMD for individuals at lower risk.**<br><br>**There are issues of equity among racialized communities, those with lower SES or those in rural/remote regions that would remain unchanged (133,135).**<br><br>Significant inequities in access to BMD testing in Ontario have been described by Cadarette et al., (2007) due to age, health beliefs, education, income, use of preventive health services, region (rural/remote), and provider sex (131). Data from adults 50+ in Ontario (2017/2018) showed a 2-fold variation between the region with the highest age standardized rate of screening (10.0 per 100 in the Central[19] LHIN) and that with the lowest rate (4.2	

[19] Northern Toronto, Etobicoke, York and South Simcoe regions

Appendix 10, as supplied by the authors. Appendix to: Thériault G, Limburg H, Klarenbach S, et al. Recommendations on screening for primary prevention of fragility fractures. *CMAJ* 2023. doi: 10.1503/cmaj.221219. Copyright © 2023 The Author(s) or their employer(s). To receive this resource in an accessible format, please contact us at cmajgroup@cmaj.ca.

per 100 in the North-West[20] LHIN) (129). Data from Manitoba (2007) also found that females in the highest SES category had significantly higher BMD utilization rates regardless of age or morbidity (132). Concerns about equitable access to BMD were identified among Indigenous populations in Canada. Leslie et al., 2012 found that they were “one half to one tenth as likely to receive post-fracture BMD testing, osteoporosis treatment, or an osteoporosis diagnosis than the general population” (133–135).

The risk of fracture varies across Canada. Age-standardized annual hip fracture rates were lowest in Quebec (124.7 per 100,000) and highest in the Northwest Territories (188.3 per 100,000) (136). Access to a family doctor in Canada also varies by province. In 2013, 15% of Canadians reported having no regular family physician (range from 7.2% in NB to 24.6% in Quebec) (137). Figure 1 provides information on the variation in between provinces in terms of fragility fractures.

Figure 1: Canadian Fragility Fracture score-card (138)

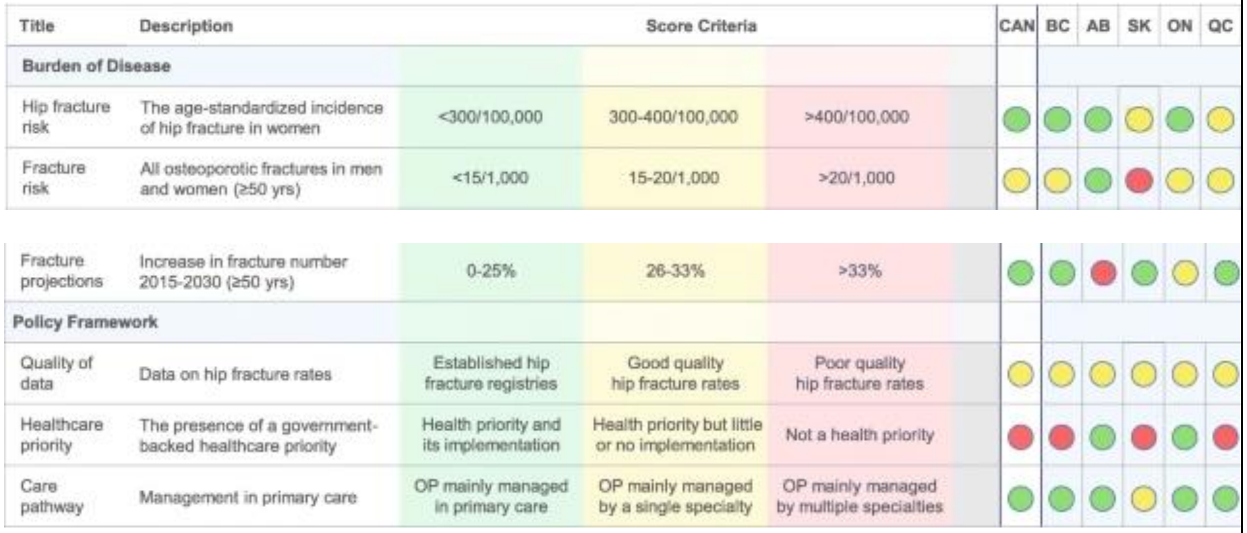

Title	Description	Score Criteria			CAN	BC	AB	SK	ON	QC
**Burden of Disease**										
Hip fracture risk	The age-standardized incidence of hip fracture in women	<300/100,000	300-400/100,000	>400/100,000	green	green	green	yellow	green	yellow
Fracture risk	All osteoporotic fractures in men and women (≥50 yrs)	<15/1,000	15-20/1,000	>20/1,000	yellow	yellow	green	red	yellow	yellow
Fracture projections	Increase in fracture number 2015-2030 (≥50 yrs)	0-25%	26-33%	>33%	green	green	red	green	yellow	green
**Policy Framework**										
Quality of data	Data on hip fracture rates	Established hip fracture registries	Good quality hip fracture rates	Poor quality hip fracture rates	yellow	yellow	yellow	yellow	yellow	yellow
Healthcare priority	The presence of a government-backed healthcare priority	Health priority and its implementation	Health priority but little or no implementation	Not a health priority	red	red	green	red	green	red
Care pathway	Management in primary care	OP mainly managed in primary care	OP mainly managed by a single specialty	OP mainly managed by multiple specialties	green	green	green	yellow	green	green

[20] Thunder Bay, Kenora, Rainy River and Northern regions

Appendix 10, as supplied by the authors. Appendix to: Thériault G, Limburg H, Klarenbach S, et al. Recommendations on screening for primary prevention of fragility fractures. *CMAJ* 2023. doi: 10.1503/cmaj.221219. Copyright © 2023 The Author(s) or their employer(s). To receive this resource in an accessible format, please contact us at cmajgroup@cmaj.ca.

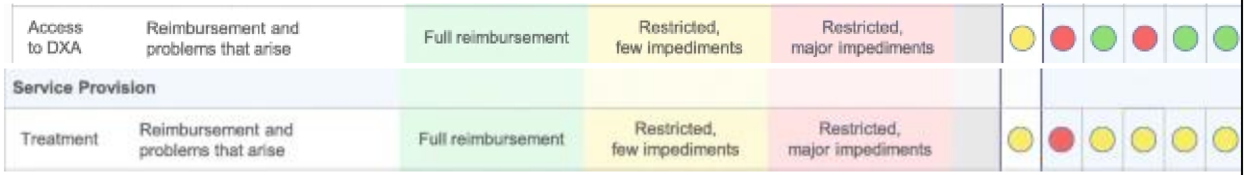

Access to DXA	Reimbursement and problems that arise	Full reimbursement	Restricted, few impediments	Restricted, major impediments	
**Service Provision**					
Treatment	Reimbursement and problems that arise	Full reimbursement	Restricted, few impediments	Restricted, major impediments	

ACCEPTABILITY	**Is the intervention acceptable to key stakeholders?**<br><br>○No<br>○Probably no<br>○ Probably yes<br>○Yes<br><br>**X** Varies<br>○Don’t know		

Appendix 10, as supplied by the authors. Appendix to: Thériault G, Limburg H, Klarenbach S, et al. Recommendations on screening for primary prevention of fragility fractures. *CMAJ* 2023. doi: 10.1503/cmaj.221219. Copyright © 2023 The Author(s) or their employer(s). To receive this resource in an accessible format, please contact us at cmajgroup@cmaj.ca.

JUDGEMENT – ACCEPTABILITY

A recommendation for risk assessment-first screening (females ≥65 years) may or may not be acceptable to primary care physicians depending on current practice. Physicians may not be routinely using a risk assessment tool first, but this should be acceptable in the context of shared-decision making. The additional burden of time (to perform the initial FRAX) should be offset by a reduction in BMD scan referrals.

This recommendation may or may not be acceptable to patients depending on their willingness to accept the accuracy of a risk assessment result without BMD. However, this is currently being used in many European countries (24–26).

A recommendation against screening (males ≥40 years) may or may not be acceptable. The current Osteoporosis Canada guideline (4) recommends screening males ≥65 years but data from the 2009 CCHS showed that only 20% of this group self-reported ever being screened (140).

A recommendation against screening (females 40-64 years) may be acceptable. This aligns with the Osteoporosis Canada guidelines (4), but 35% of females 40-64 years reported being screened in a 2009 Canadian survey (140). Additionally data from KQ4 showed that females aged 50-65 had a high willingness to be screened.

Appendix 10, as supplied by the authors. Appendix to: Thériault G, Limburg H, Klarenbach S, et al. Recommendations on screening for primary prevention of fragility fractures. *CMAJ* 2023. doi: 10.1503/cmaj.221219. Copyright © 2023 The Author(s) or their employer(s). To receive this resource in an accessible format, please contact us at cmajgroup@cmaj.ca.

Data on whether Canadian physicians commonly use a BMD-first or risk assessment-first approach to screening is limited. However, it appears that risk assessment tools (e.g. FRAX) are more commonly recommended for use when considering treatment (i.e. following BMD assessment) (145). Screening with BMD-first to prevent fragility fractures is also currently recommended by Osteoporosis Canada for males and females ≥65 years (4).

There also appears to be a lack of clarity around when to initiate screening. A 2016 study of Ontario family physicians found "a tendency for baseline BMD testing in healthy, postmenopausal women and a lack of clarity on the appropriate age for screening for men in particular." (139)

Data from KQ4 showed that females (age 50-65 years) have a high willingness to be screened with BMD but the acceptability of risk assessment tools as part of screening is unknown. Males are less likely to screen for risk of fracture than females and therefore a recommendation against screening males may be more acceptable. In 2015, 4.81% of males and 8.15% of females over 40 years reported receiving a BMD screening test (10).

The Canadian Community Health survey (2009) collected data on the percentage of individuals who self-reported ever having a BMD scan (by age group and sex). It showed that 7% of males and 35% of females aged 40-64 reported at least one previous BMD scan. For males and females 65-100 years this number was 20% and 68% respectively (140).

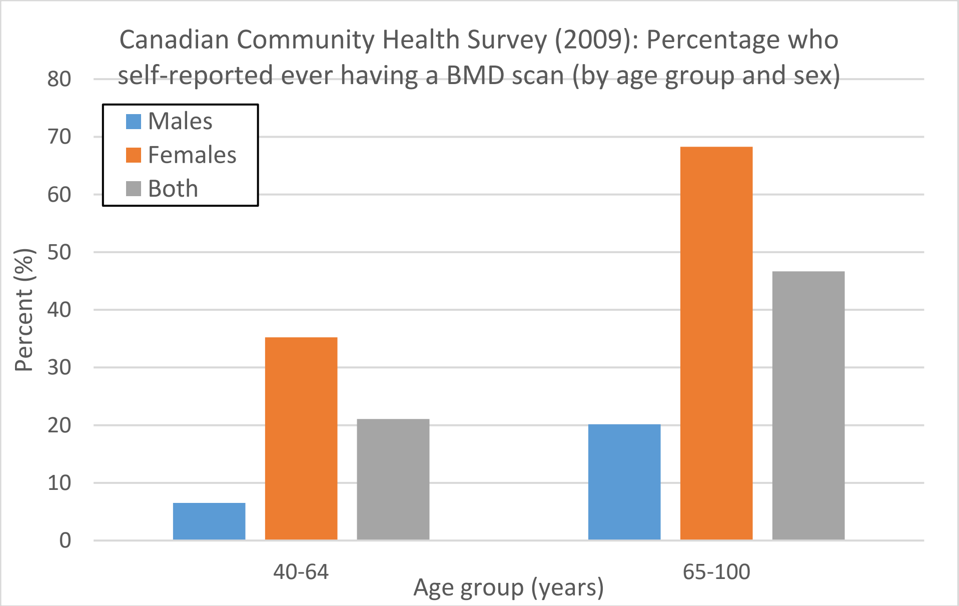

Appendix 10, as supplied by the authors. Appendix to: Thériault G, Limburg H, Klarenbach S, et al. Recommendations on screening for primary prevention of fragility fractures. *CMAJ* 2023. doi: 10.1503/cmaj.221219. Copyright © 2023 The Author(s) or their employer(s). To receive this resource in an accessible format, please contact us at cmajgroup@cmaj.ca.

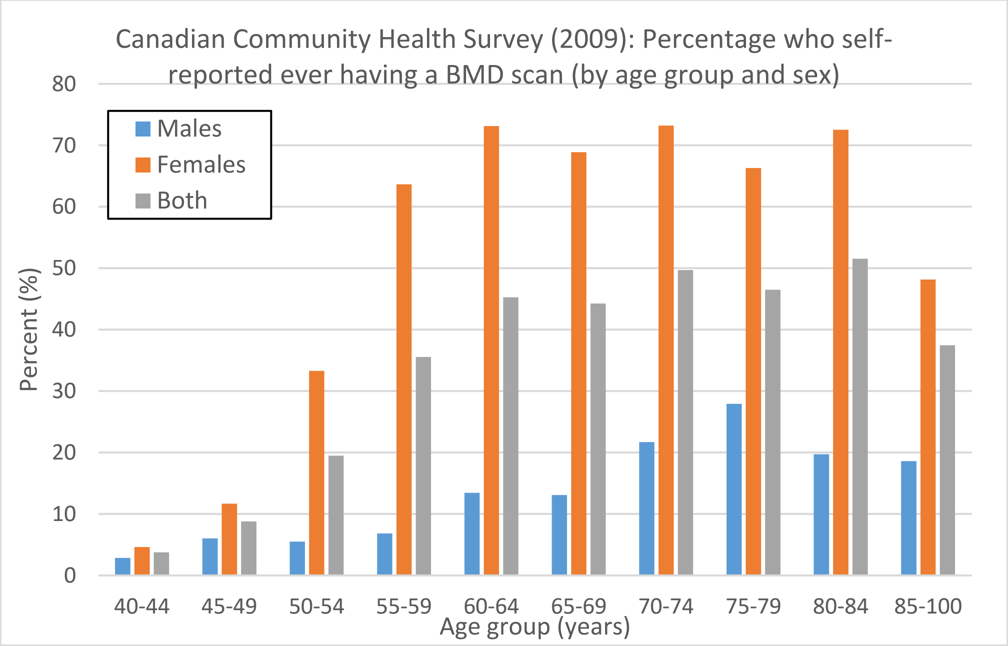

Data source: Canadian Community Health Survey, 2009 at Statistics Canada (145). Analysis by: Centre for Surveillance and Applied Research, Public Health Agency of Canada.

Appendix 10, as supplied by the authors. Appendix to: Thériault G, Limburg H, Klarenbach S, et al. Recommendations on screening for primary prevention of fragility fractures. *CMAJ* 2023. doi: 10.1503/cmaj.221219. Copyright © 2023 The Author(s) or their employer(s). To receive this resource in an accessible format, please contact us at cmajgroup@cmaj.ca.

FEASIBILITY	**Is the intervention feasible to implement?**<br><br>○No<br>○Probably no<br>○ Probably yes<br>**X** Yes<br><br>○Varies<br>○Don't know	**JUDGEMENT – FEASIBILITY**<br><br>**Primary care physicians**<br>**A recommendation for risk assessment-first screening (≥65 years) should be feasible in the context of patient-centred care.**<br><br>**A recommendation against screening males (≥40 years) would be feasible.**<br><br>**A recommendation against screening females (40-64 years) would be feasible.**	E.g. Ontario:<br>OHIP covers annual BMD tests for individuals at high risk for osteoporosis and future fractures. Individuals at low risk are eligible for a baseline BMD test and a second BMD test 36 months after the baseline. Third and subsequent BMD tests for low-risk individuals are insured by OHIP once every 60 months (141).

Appendix 10, as supplied by the authors. Appendix to: Thériault G, Limburg H, Klarenbach S, et al. Recommendations on screening for primary prevention of fragility fractures. *CMAJ* 2023. doi: 10.1503/cmaj.221219. Copyright © 2023 The Author(s) or their employer(s). To receive this resource in an accessible format, please contact us at cmajgroup@cmaj.ca.

Summary of judgements

	JUDGEMENT							IMPLICATIONS
**PROBLEM**	No	Probably no	Probably yes	**Yes**		Varies	Don't know	
**DESIRABLE EFFECTS**	Trivial	**Small**<br><br>**(Females ≥65 years)**	Moderate	Large		Varies	**Don't know**<br><br>**(Males ≥40 and females 40-64 years)**	Trivial (general population)<br>Small (Selected population)
**UNDESIRABLE EFFECTS**	Large	**Small**	Moderate	Trivial		Varies	Don't know	Small = Adverse events<br>Moderate= Overdiagnosis
**CERTAINTY OF EVIDENCE**	**Very low**<br><br>**(Males ≥40 and females 40-64 years)**	**Low**<br><br>**(Females ≥65 years)**	Moderate	High			No included studies	(See section on Strong recommendations based on low or very low certainty evidence)
**VALUES**	**Important uncertainty or variability**	Possibly important uncertainty or variability	Probably no important uncertainty or variability	No important uncertainty or variability				Screening is acceptable to patients but not necessarily treatment

Appendix 10, as supplied by the authors. Appendix to: Thériault G, Limburg H, Klarenbach S, et al. Recommendations on screening for primary prevention of fragility fractures. *CMAJ* 2023. doi: 10.1503/cmaj.221219. Copyright © 2023 The Author(s) or their employer(s). To receive this resource in an accessible format, please contact us at cmajgroup@cmaj.ca.

	JUDGEMENT							IMPLICATIONS
**BALANCE OF EFFECTS**	**Favors the comparison (Males ≥40 and females 40-64 years)**	Probably favors the comparison	Does not favor either the intervention or the comparison	**Probably favors the intervention (Females ≥65 years)**	Favors the intervention	Varies	Don't know	
**RESOURCES REQUIRED**	Large costs	Moderate costs	Negligible costs and savings	**Moderate savings**	Large savings	Varies	Don't know	
**CERTAINTY OF EVIDENCE OF REQUIRED RESOURCES**	Very low	**Low**	Moderate	High			No included studies	Certainty that it is cost-effective (cost savings)
**COST EFFECTIVENESS**	Favors the comparison	Probably favors the comparison	Does not favor either the intervention or the comparison	**Probably favors the intervention**	Favors the intervention	Varies	No included studies	
**EQUITY**	Reduced	Probably reduced	Probably no impact	**Probably increased**	Increased	Varies	Don't know	

Appendix 10, as supplied by the authors. Appendix to: Thériault G, Limburg H, Klarenbach S, et al. Recommendations on screening for primary prevention of fragility fractures. *CMAJ* 2023. doi: 10.1503/cmaj.221219. Copyright © 2023 The Author(s) or their employer(s). To receive this resource in an accessible format, please contact us at cmajgroup@cmaj.ca.

	JUDGEMENT							IMPLICATIONS
**ACCEPTABILITY**	No	Probably no	Probably yes	Yes		**Varies**	Don't know	Implications for KT
**FEASIBILITY**	No	Probably no	Probably yes	**Yes**		Varies	Don't know	

Conclusions

Should we screen patients to prevent fragility fractures?

**TYPE OF RECOMMENDATION**	**Strong recommendation against the intervention**<br><br>**X (Males ≥40 years and females 40-65 years)**	Conditional recommendation against the intervention<br><br>○	Conditional recommendation for either the intervention or the comparison<br><br>○	**Conditional recommendation for the intervention**<br><br>**X (Females ≥65 years)**	Strong recommendation for the intervention<br><br>○
**RECOMMENDATION**	**FEMALES ≥65 YEARS**<br><br>In females 65 years or older, we recommend screening with the Canadian FRAX risk assessment tool (without BMD) and using the 10-year absolute risk of Major Osteoporotic Fracture to facilitate shared decision-making about the possible benefits and harms of treatment. If the patient is considering preventive treatment, we recommend refining fracture risk by adding the BMD value in FRAX.				

Appendix 10, as supplied by the authors. Appendix to: Thériault G, Limburg H, Klarenbach S, et al. Recommendations on screening for primary prevention of fragility fractures. *CMAJ* 2023. doi: 10.1503/cmaj.221219. Copyright © 2023 The Author(s) or their employer(s). To receive this resource in an accessible format, please contact us at cmajgroup@cmaj.ca.

	(Conditional recommendation, low certainty evidence)<br><br>**<u>FEMALES 40-64 YEARS</u>**<br>In females 40-64 years, we recommend not screening. *(Strong recommendation, very low certainty evidence)*<br><br>**<u>MALES ≥40 YEARS</u>**<br>In males ≥40 years we recommend not screening. *(Strong recommendation, very low certainty evidence)*<br><br>*These recommendations apply to community-dwelling individuals who are not currently on pharmacotherapy to prevent fragility fractures.*
**JUSTIFICATION**	Females ≥65 years<br>- The small benefit (reduction in hip fractures and clinical fragility fractures) outweighs the moderate risk of overdiagnosis, small increased risk of rare harms (osteonecrosis of the jaw, atypical femoral fractures) and small to moderate increase in some non-serious AEs (e.g. GI AEs, arthralgia, myalgia, pyrexia, chills, & influenza-like symptoms)<br>- Recommendation for a risk assessment-first screening process is based on the methods used in the trials (i.e. risk assessment-first screening with various European FRAX followed by BMD if indicated and evidence from KQ2 showing Canadian FRAX to probably be well-calibrated to predict 5 or 10-year hip or clinical fragility fracture.<br>- There are also potential resource savings associated with a risk assessment-first screening process CAROC was not recommended as part of the risk assessment-first screening process as it does not allow risk calculation without BMD.<br><br>Females 40-64 years<br>- The evidence about all eligible / offer-to-screen populations (females 45-54 years) is very uncertain.<br>- Screening females 40-64 would result in increased costs for uncertain benefits

Appendix 10, as supplied by the authors. Appendix to: Thériault G, Limburg H, Klarenbach S, et al. Recommendations on screening for primary prevention of fragility fractures. *CMAJ* 2023. doi: 10.1503/cmaj.221219. Copyright © 2023 The Author(s) or their employer(s). To receive this resource in an accessible format, please contact us at cmajgroup@cmaj.ca.

	A conclusion of possible benefit requires a high level of speculation on linkages of uncertain evidence, but there is high certainty that some patients would be harmed or scarce health care resources expended (123).<br><br>Males ≥40 years<br>- The evidence about offer-to-screen in selected populations (males ≥65 years) is very uncertain.<br>- There was no evidence for males 40-64 years<br>- Screening males ≥65 years would result in Increased costs for uncertain benefits<br>A conclusion of possible benefit requires a high level of speculation on linkages of uncertain evidence, but there is high certainty that some patients would be harmed or scarce health care resources expended (123).
**SUBGROUP CONSIDERATIONS**	A priori subgroups of interest included age, sex, and menopausal status.<br><br>The data was stratified by age and sex with separate recommendations for <u>females aged ≥65 years, females aged 40-64 years and males ≥40 years (see above).</u><br><br>The evidence for males was also stratified by age with very low certainty data on males ≥65 years and no available data for males 40-64 years. As both age groups showed very uncertain results we combined them into one age category.<br><br>Data specific to menopausal status was not available and a subgroup analysis was not completed for this group.
**IMPLEMENTATION CONSIDERATIONS**	• Ethnicity<br>○ Data underpinning the Canadian FRAX may be sparse for certain ethnicities (e.g., black, Asian, Hispanic). Country-specific versions of FRAX are available (https://frax.shef.ac.uk/FRAX/) as well as adjustments for Black, Hispanic and Asian populations in the United States FRAX; however, there have been some concerns raised about the use of race- or ethnicity-based algorithms.

Appendix 10, as supplied by the authors. Appendix to: Thériault G, Limburg H, Klarenbach S, et al. Recommendations on screening for primary prevention of fragility fractures. *CMAJ* 2023. doi: 10.1503/cmaj.221219. Copyright © 2023 The Author(s) or their employer(s). To receive this resource in an accessible format, please contact us at cmajgroup@cmaj.ca.

	• Risk assessment-first screening<br>○ Implementation considerations include a transition to risk assessment-first screening where this is not currently performed (e.g. direct to BMD screening)<br>▪ Risk assessment-first screening should be acceptable in the context of shared-decision making. The additional burden of time (to perform the initial FRAX) should be offset by a reduction in BMD scan referrals.<br>○ Additionally, CAROC is used in some jurisdictions (instead of FRAX).<br>▪ Although CAROC may be adequately calibrated to predict category of clinical fragility fracture risk it cannot be used without BMD. Therefore, FRAX should be used as the initial risk assessment tool.<br><br>• Shared decision making between the physician and patient is extremely important due to the conditional recommendation and the variable patient values and preferences<br>○ This recommendation is conditional based on the low certainty of the evidence, differences in benefit seen in “select” vs general populations and the variable patient values and preferences<br>○ Patients should be informed of the consequences of screening (overdiagnosis, treatment AEs) and their willingness to undergo treatment should be considered as part of the criteria for FRAX with BMD.<br><br>• The frequency of screening was not specifically examined as a key question for this analysis, but may be a consideration for implementation<br>○ No RCTs of different screening intervals were found during the SR for KQ1<br>○ We did not perform a systematic review of observational studies of different screening intervals<br>○ Evidence from an environmental scan of cohort studies found that repeated BMD scans at 3-8 years did not improve fracture risk prediction (females aged ≥50 years) (147-149)

Appendix 10, as supplied by the authors. Appendix to: Thériault G, Limburg H, Klarenbach S, et al. Recommendations on screening for primary prevention of fragility fractures. *CMAJ* 2023. doi: 10.1503/cmaj.221219. Copyright © 2023 The Author(s) or their employer(s). To receive this resource in an accessible format, please contact us at cmajgroup@cmaj.ca.

	○ A systematic review performed by the USPSTF in 2018 found that "Some observational and modeling studies have suggested screening intervals based on age, baseline BMD, and calculated projected time to transition to osteoporosis. However, limited evidence from 2 good-quality studies found no benefit in predicting fractures from repeating bone measurement testing 4 to 8 years after initial screening."(5)
**MONITORING AND EVALUATION**	Evaluation of clinician uptake of a risk assessment-first screening program (i.e. initial screening with FRAX) will help determine uptake of the guideline.<br><br>Rates of screening among the target population (females ≥65 years) should be monitored to ensure adherence to the guideline.<br><br>Monitoring to ensure males and younger females are not being screened will help evaluate if the guideline is being followed.
**RESEARCH PRIORITIES**	There is a lack of trials on younger females (<65 years) or males (any age) for screening to reduce fragility fractures. Additionally, there is a need for evidence on the frequency of screening (i.e. screening intervals) and at what age to stop screening. Researchers are also encouraged to include a general population approach which doesn't limit the sample to only those who agree to complete a risk assessment tool.<br><br>Due to the important uncertainty and variability in patient values and preferences for treatment to prevent fragility fractures, more research into this area is needed. Specifically, decision aids or other knowledge translation tools are needed to help patients understand the true benefits and harms of screening and treatment.

Appendix 10, as supplied by the authors. Appendix to: Thériault G, Limburg H, Klarenbach S, et al. Recommendations on screening for primary prevention of fragility fractures. *CMAJ* 2023. doi: 10.1503/cmaj.221219. Copyright © 2023 The Author(s) or their employer(s). To receive this resource in an accessible format, please contact us at cmajgroup@cmaj.ca.

References

1. Hernlund E, Svedbom A, Ivergård M, Compston J, Cooper C, Stenmark J, et al. Osteoporosis in the European Union: medical management, epidemiology and economic burden. Arch Osteoporos. 2013 Dec 11;8(1–2).
2. Qaseem A, Forciea MA, McLean RM, Denberg TD. Treatment of low bone density or osteoporosis to prevent fractures in men and women: A clinical practice guideline update from the American college of physicians. Ann Intern Med. 2017 Jun;166(11):818–39.
3. Knudtson M. Osteoporosis: Background and Overview. J Nurse Pract. 2009;5(6):S4–12.
4. Papaioannou A, Morin S, Cheung AM, Atkinson S, Brown JP, Feldman S, et al. 2010 clinical practice guidelines for the diagnosis and management of osteoporosis in Canada: summary. Can Med Assoc J. 2010 Nov 23;182(17).
5. Viswanathan M, Reddy S, Berkman N, Cullen K, Middleton JC, Nicholson WK, et al. Screening to Prevent Osteoporotic Fractures: An Evidence Review for the U.S. Preventive Services Task Force. Evidence Synthesis No.162. Rockville, MD: Agency for Healthcare Research and Quality; 2018.
6. U.S. Preventive Services Task Force. Final Recommendation Statement Osteoporosis to Prevent Fractures: Screening [Internet]. Vol. 2018. 2018 [cited 2018 Jul 27]. Available from: https://www.uspreventiveservicestaskforce.org/Page/Document/RecommendationStatementFinal/osteoporosis-screening1.
7. The National Osteoporosis Guideline Group. NOGG 2017: Clinical guidelines for the prevention and treatment of osteoporosis [Internet]. 2017 [cited 2017 Nov 27]. Available from: https://www.sheffield.ac.uk/NOGG/NOGG Guideline 2017.pdf.
8. Pisani P, Renna MD, Conversano F, Casciaro E, Di Paola M, Quarta E, et al. Major osteoporotic fragility fractures: Risk factor updates and societal impact. World J Orthop. 2016 Mar;7(3):171–81.
9. The North American Menopause Society. Management of osteoporosis in postmenopausal women: 2010 position statement of The North American Menopause Society. Menopause. 2010;17(1):25–54.
10. Public Health Agency of Canada. Canadian Chronic Disease Surveillance System (CCDSS) [Internet]. Vol. 2021. 2019 [cited 2021 Feb 22]. Available from: https://health-infobase.canada.ca/ccdss/data-tool/Age?V=32&M=3&S=B&Y=2016
11. Hopkins RB, Burke N, Keyserlingk C Von, Leslie WD, Morin SN, Adachi JD, et al. The current economic burden of illness of osteoporosis in Canada. Osteoporos Int. 2016 Oct;27(10):3023–32.
12. Kanis JA, Melton LJ 3rd, Christiansen C, Johnston CC, Khaltaev N. The diagnosis of osteoporosis. J Bone Miner Res. 1994 Aug;9(8):1137–41.
13. Public Health Agency of Canada. Fast Facts from the 2009 Canadian Community Health Survey - Osteoporosis Rapid Response. Vol. 2017. 2010.
14. Kanis JA, Harvey NC, Johansson H, Oden A, McCloskey E V, Leslie WD. Overview of Fracture Prediction Tools. J Clin Densitom. 2017 Jul;20(3):444–50.

Appendix 10, as supplied by the authors. Appendix to: Thériault G, Limburg H, Klarenbach S, et al. Recommendations on screening for primary prevention of fragility fractures. *CMAJ* 2023. doi: 10.1503/cmaj.221219. Copyright © 2023 The Author(s) or their employer(s). To receive this resource in an accessible format, please contact us at cmajgroup@cmaj.ca.

15. Camacho PM, Petak SM, Binkley N, Diab DL, Eldeiry LS, Farooki A, et al. American Association of Clinical Endocrinologists/American College of Endorinology Clinical Practice Guidelines for the Diagnosis and Treatment of Postmenopausal Osteoporosis-2020 Update. Endocr Pract. 2020 May;26(Suppl 1):1–46.
16. Si L, Winzenberg TM, de Graaff B, Palmer AJ. A systematic review and meta-analysis of utility-based quality of life for osteoporosis-related conditions. Osteoporos Int. 2014 Aug;25(8):1987–97.
17. Dyer SM, Crotty M, Fairhall N, Magaziner J, Beaupre LA, Cameron ID, et al. A critical review of the long-term disability outcomes following hip fracture. BMC Geriatr. 2016 Nov;16:158.
18. Barcenilla-Wong AL, Chen JS, Cross MJ, March LM. The Impact of Fracture Incidence on Health Related Quality of Life among Community-Based Postmenopausal Women. J Osteoporos. 2015;2015:717914.
19. Ioannidis G, Papaioannou A, Hopman WM, Akhtar-Danesh N, Anastassiades T, Pickard L, et al. Relation between fractures and mortality: results from the Canadian Multicentre Osteoporosis Study. Can Med Assoc J. 2009 Sep 1;181(5).
20. Teng GG, Curtis JR, Saag KG. Mortality and osteoporotic fractures: is the link causal, and is it modifiable? Clin Exp Rheumatol. 2008;26(5):S125-37.
21. Health Canada. Seniors and Aging - Osteoporosis. Vol. 2021. 2007.
22. Kanis JA, McCloskey E V., Johansson H, Cooper C, Rizzoli R, Reginster J-Y. European guidance for the diagnosis and management of osteoporosis in postmenopausal women. Osteoporos Int. 2013 Jan 19;24(1):23–57.
23. Barr RJ, Stewart A, Torgerson DJ, Reid DM. Population screening for osteoporosis risk: a randomised control trial of medication use and fracture risk. Osteoporos Int. 2010 Apr;21(4):561–8.
24. Rubin KH, Rothmann MJ, Holmberg T, Hoiberg M, Moller S, Barkmann R, et al. Effectiveness of a two-step population-based osteoporosis screening program using FRAX: the randomized Risk-stratified Osteoporosis Strategy Evaluation (ROSE) study. Osteoporos Int. 2018 Mar;29(3):567–78.
25. Merlijn T, Swart KM, Schoor NM, Heymans MW, Zwaard BC, Heijden AA, et al. The Effect of a Screening and Treatment Program for the Prevention of Fractures in Older Women: A Randomized Pragmatic Trial. J Bone Miner Res. 2019 Nov;34(11).
26. Shepstone L, Lenaghan E, Cooper C, Clarke S, Fong-Soe-Khioe R, Fordham R, et al. Screening in the community to reduce fractures in older women (SCOOP): a randomised controlled trial. Lancet. 2018;391(10122):741–7.
27. Kern LM, Powe NR, Levine MA, Fitzpatrick AL, Harris TB, Robbins J, et al. Association between screening for osteoporosis and the incidence of hip fracture. Ann Intern Med. 2005 Feb;142(3):173–81.
28. Prior JC, Langsetmo L, Lentle BC, Berger C, Goltzman D, Kovacs CS, et al. Ten-year incident osteoporosis-related fractures in the population-based Canadian Multicentre Osteoporosis Study - comparing site and age-specific risks in women and men. Bone. 2015 Feb;71:237–43.
29. LaCroix AZ, Buist DSM, Brenneman SK, Abbott TA. Evaluation of Three Population-Based Strategies for Fracture Prevention. Med Care. 2005 Mar;43(3):293–302.
30. Azagra R, Zwart M, Encabo G, Aguyé A, Martin-Sánchez JC, Puchol-Ruiz N, et al. Rationale of the Spanish FRAX model in decision-making for predicting osteoporotic fractures: an update of FRIDEX cohort of Spanish women. BMC Musculoskelet Disord. 2016 Dec 17;17(1):262.

Appendix 10, as supplied by the authors. Appendix to: Thériault G, Limburg H, Klarenbach S, et al. Recommendations on screening for primary prevention of fragility fractures. *CMAJ* 2023. doi: 10.1503/cmaj.221219. Copyright © 2023 The Author(s) or their employer(s). To receive this resource in an accessible format, please contact us at cmajgroup@cmaj.ca.

31. Bolland MJ, Siu AT, Mason BH, Horne AM, Ames RW, Grey AB, et al. Evaluation of the FRAX and Garvan fracture risk calculators in older women. J Bone Miner Res. 2011 Feb;26(2):420–7.
32. Sornay-Rendu E, Munoz F, Delmas PD, Chapurlat RD. The FRAX tool in French women: How well does it describe the real incidence of fracture in the OFELY cohort?. J Bone Miner Res. 2010 Oct;25(10):2101–7.
33. Tamaki J, Iki M, Sato Y, Winzenrieth R, Kajita E, Kagamimori S, et al. Does Trabecular Bone Score (TBS) improve the predictive ability of FRAX R for major osteoporotic fractures according to the Japanese Population-Based Osteoporosis (JPOS) cohort study?. J Bone Miner Metab. 2019 Jan;37(1):161–70.
34. Yin MT, Shiau S, Rimland D, Gibert CL, Bedimo RJ, Rodriguez-Barradas MC, et al. Fracture Prediction With Modified-FRAX in Older HIV-Infected and Uninfected Men. J Acquir Immune Defic Syndr JAIDS. 2016 Aug;72(5):513–20.
35. Crandall CJ, Larson J, LaCroix A, Cauley JA, LeBoff MS, Li W, et al. Predicting Fracture Risk in Younger Postmenopausal Women: Comparison of the Garvan and FRAX Risk Calculators in the Women’s Health Initiative Study. J Gen Intern Med. 2019 Nov;34(2):235–42.
36. Czerwiński E, Borowy P, Kumorek A, Amarowicz J, Górkiewicz M, Milert A. Fracture Risk Prediction in Outpatients from Krakow Region Using FRAX Tool Versus Fracture Risk in 11-year Follow-up. Ortop Traumatol Rehabil. 2013 Oct 24;15(6):617–28.
37. Ettinger B, Ensrud KE, Blackwell T, Curtis JR, Lapidus JA, Orwoll ES. Performance of FRAX in a cohort of community-dwelling, ambulatory older men: the Osteoporotic Fractures in Men (MrOS) study. Osteoporos Int. 2013 Apr 21;24(4):1185–93.
38. Goldshtein I, Gerber Y, Ish-Shalom S, Leshno M. Fracture Risk Assessment With FRAX Using Real-World Data in a Population-Based Cohort From Israel. Am J Epidemiol. 2018 Jan 1;187(1):94–102.
39. Marques A, Lucas R, Simoes E, Verstappen SMM, Jacobs JWG, da Silva JAP. Do we need bone mineral density to estimate osteoporotic fracture risk? A 10-year prospective multicentre validation study. RMD Open. 2017;3(2):e000509.
40. Pluskiewicz W, Adamczyk P, Czekajło A, Grzeszczak W, Drozdzowska B. High fracture probability predicts fractures in a 4-year follow-up in women from the RAC-OST-POL study. Osteoporos Int. 2015 Dec 14;26(12):2811–20.
41. Premaor M, Parker RA, Cummings S, Ensrud K, Cauley JA, Lui L-Y, et al. Predictive value of FRAX for fracture in obese older women. J Bone Miner Res. 2013 Jan;28(1):188–95.
42. Pressman AR, Lo JC, Chandra M, Ettinger B. Methods for assessing fracture risk prediction models: experience with FRAX in a large integrated health care delivery system. J Clin Densitom. 2011 Oct;14(4):407–15.
43. Fraser L-A, Langsetmo L, Berger C, Ioannidis G, Goltzman D, Adachi JD, et al. Fracture prediction and calibration of a Canadian FRAX® tool: a population-based report from CaMos. Osteoporos Int. 2011 Mar 16;22(3):829–37.
44. Leslie WD, Majumdar SR, Morin SN, Lix LM, Johansson H, Oden A, et al. FRAX for fracture prediction shorter and longer than 10 years: the Manitoba BMD registry. Osteoporos Int. 2017 Sep 7;28(9):2557–64.
45. Li G, Thabane L, Papaioannou A, Adachi JD. Comparison between frailty index of deficit accumulation and fracture risk assessment tool (FRAX) in prediction of risk of fractures. Bone. 2015 Aug;77:107–14.
46. Crandall CJ, Schousboe JT, Morin SN, Lix LM, Leslie W. Performance of FRAX and FRAX-Based Treatment Thresholds in Women Aged 40 Years and Older: The Manitoba BMD Registry. J Bone Miner Res. 2019 Aug 17;34(8):1419–27.
47. Dagan N, Cohen-Stavi C, Leventer-Roberts M, Balicer RD. External validation and comparison of three prediction tools for risk of

Appendix 10, as supplied by the authors. Appendix to: Thériault G, Limburg H, Klarenbach S, et al. Recommendations on screening for primary prevention of fragility fractures. *CMAJ* 2023. doi: 10.1503/cmaj.221219. Copyright © 2023 The Author(s) or their employer(s). To receive this resource in an accessible format, please contact us at cmajgroup@cmaj.ca.

osteoporotic fractures using data from population based electronic health records: retrospective cohort study. BMJ. 2017 Jan 19;i6755.
48. Desbiens L, Sidibé A, Beaudoin C, Jean S, Mac-Way F. Comparison of Fracture Prediction Tools in Individuals Without and With Early Chronic Kidney Disease: A Population-Based Analysis of CARTaGENE. J Bone Miner Res. 2020 Jun 10;35(6):1048–57.
49. Holloway KL, Mohebbi M, Betson AG, Hans D, Hyde NK, Brennan-Olsen SL, et al. Prediction of major osteoporotic and hip fractures in Australian men using FRAX scores adjusted with trabecular bone score. Osteoporos Int. 2018 Jan 23;29(1):101–8.
50. Melton LJ 3rd, Atkinson EJ, Achenbach SJ, Kanis JA, Therneau TM, Johansson H, et al. Potential Extensions of the US FRAX Algorithm. J Osteoporos. 2012;2012:528790.
51. Iki M, Fujita Y, Tamaki J, Kouda K, Yura A, Sato Y, et al. Trabecular bone score may improve FRAX® prediction accuracy for major osteoporotic fractures in elderly Japanese men: the Fujiwara-kyo Osteoporosis Risk in Men (FORMEN) Cohort Study. Osteoporos Int. 2015 Jun 10;26(6):1841–8.
52. Tanaka S, Yoshimura N, Kuroda T, Hosoi T, Saito M, Shiraki M. The Fracture and Immobilization Score (FRISC) for risk assessment of osteoporotic fracture and immobilization in postmenopausal women–A joint analysis of the Nagano, Miyama, and Taiji Cohorts. Bone. 2010 Dec;47(6):1064–70.
53. Tebé Cordomí C, del Río LM, Di Gregorio S, Casas L, Estrada M-D, Kotzeva A, et al. Validation of the FRAX Predictive Model for Major Osteoporotic Fracture in a Historical Cohort of Spanish Women. J Clin Densitom. 2013 Apr;16(2):231–7.
54. Tremollieres FA, Pouilles J-M, Drewniak N, Laparra J, Ribot CA, Dargent-Molina P. Fracture risk prediction using BMD and clinical risk factors in early postmenopausal women: sensitivity of the WHO FRAX tool. J Bone Miner Res. 2010 May;25(5):1002–9.
55. Leslie WD, Majumdar SR, Lix LM, Josse RG, Johansson H, Oden A, et al. Direct comparison of FRAX(R) and a simplified fracture risk assessment tool in routine clinical practice: a registry-based cohort study. Osteoporos Int. 2016 Nov;27(9):2689–95.
56. Gourlay ML, Ritter VS, Fine JP, Overman RA, Schousboe JT, Cawthon PM, et al. Comparison of fracture risk assessment tools in older men without prior hip or spine fracture: the MrOS study. Arch Osteoporos. 2017 Oct;12(1):91.
57. Langsetmo L, Nguyen T V., Nguyen ND, Kovacs CS, Prior JC, Center JR, et al. Independent external validation of nomograms for predicting risk of low-trauma fracture and hip fracture. Can Med Assoc J. 2011 Feb 8;183(2):E107–14.
58. Reyes Dominguez A, Sosa Cabrera N, Saavedra Santana P, de Tejada Romero M, Jodar Gimeno E, Sosa Henriquez M. Assessment of the predictive capacity of the garvan calculator of 10 year risk of fracture in a Spanish population. Rev Osteoporos y Metab Miner. 2017;9(2):55–61.
59. Ettinger B, Liu H, Blackwell T, Hoffman AR, Ensrud KE, Orwoll ES, et al. Validation of FRC, a fracture risk assessment tool, in a cohort of older men: the Osteoporotic Fractures in Men (MrOS) Study. J Clin Densitom. 2012 Jul;15(3):334–42.
60. Lo JC, Pressman AR, Chandra M, Ettinger B. Fracture risk tool validation in an integrated healthcare delivery system. Am J Manag Care. 2011 Mar;17(3):188–94.
61. Ascott-Evans BH, Guañabens N, Kivinen S, Stuckey BGA, Magaril CH, Vandormael K, et al. Alendronate Prevents Loss of Bone Density Associated With Discontinuation of Hormone Replacement Therapy. Arch Intern Med. 2003 Apr 14;163(7):789.
62. Chesnut CH, McClung MR, Ensrud KE, Bell NH, Genant HK, Harris ST, et al. Alendronate treatment of the postmenopausal osteoporotic woman: Effect of multiple dosages on bone mass and bone remodeling. Am J Med. 1995 Aug;99(2):144–52.

Appendix 10, as supplied by the authors. Appendix to: Thériault G, Limburg H, Klarenbach S, et al. Recommendations on screening for primary prevention of fragility fractures. *CMAJ* 2023. doi: 10.1503/cmaj.221219. Copyright © 2023 The Author(s) or their employer(s). To receive this resource in an accessible format, please contact us at cmajgroup@cmaj.ca.

63. Valimaki M, Farreronsminguella J, Halse J, Kroger H, Maroni M, Mulder H, et al. Effects of risedronate 5 mg/d on bone mineral density and bone turnover markers in late-postmenopausal women with osteopenia: A multinational, 24-month, randomized, double-blind, placebo-controlled, parallel-group, phase III trial. Clin Ther. 2007 Sep;29(9):1937–49.
64. Grey A, Bolland M, Mihov B, Wong S, Horne A, Gamble G, et al. Duration of antiresorptive effects of low-dose zoledronate in osteopenic postmenopausal women: a randomized, placebo-controlled trial. J Bone Miner Res. 2014 Jan;29(1):166–72.
65. Grey A, Bolland MJ, Wattie D, Horne A, Gamble G, Reid IR. The Antiresorptive Effects of a Single Dose of Zoledronate Persist for Two Years: A Randomized, Placebo-Controlled Trial in Osteopenic Postmenopausal Women. J Clin Endocrinol Metab. 2009 Feb 1;94(2):538–44.
66. Grey A, Bolland M, Wong S, Horne A, Gamble G, Reid IR. Low-dose zoledronate in osteopenic postmenopausal women: a randomized controlled trial. J Clin Endocrinol Metab. 2012 Jan;97(1):286–92.
67. Reid IR, Horne AM, Mihov B, Stewart A, Garratt E, Wong S, et al. Fracture Prevention with Zoledronate in Older Women with Osteopenia. N Engl J Med. 2018 Nov;379(25):2407–16.
68. Cummings SR, Black DM, Thompson DE, Applegate WB, Barrett-Connor E, Musliner TA, et al. Effect of alendronate on risk of fracture in women with low bone density but without vertebral fractures: results from the Fracture Intervention Trial. JAMA. 1998 Dec;280(24):2077–82.
69. Hosking D, Chilvers CE, Christiansen C, Ravn P, Wasnich R, Ross P, et al. Prevention of bone loss with alendronate in postmenopausal women under 60 years of age. Early Postmenopausal Intervention Cohort Study Group. N Engl J Med. 1998 Feb;338(8):485–92.
70. Liberman UA, Weiss SR, Broll J, Minne HW, Quan H, Bell NH, et al. Effect of oral alendronate on bone mineral density and the incidence of fractures in postmenopausal osteoporosis. The Alendronate Phase III Osteoporosis Treatment Study Group. N Engl J Med. 1995 Nov;333(22):1437–43.
71. Pols HAP, Felsenberg D, Hanley DA, Štepán J, Muñoz-Torres M, Wilkin TJ, et al. Multinational, Placebo-Controlled, Randomized Trial of the Effects of Alendronate on Bone Density and Fracture Risk in Postmenopausal Women with Low Bone Mass: Results of the FOSIT Study. Osteoporos Int. 1999 Apr;9(5):461–8.
72. Yan Y, Wang W, Zhu H, Li M, Liu J, Luo B, et al. The efficacy and tolerability of once-weekly alendronate 70 mg on bone mineral density and bone turnover markers in postmenopausal Chinese women with osteoporosis. J Bone Miner Metab. 2009 Jul 3;27(4):471–8.
73. Tucci JR, Tonino RP, Emkey RD, Peverly CA, Kher U, Santora AC 2nd. Effect of three years of oral alendronate treatment in postmenopausal women with osteoporosis. Am J Med. 1996 Nov;101(5):488–501.
74. Yuming L, Zhongzhi Z, Xiuling D, Lulu C. Efficacy and safety of risedronate sodium in treatment of postmenopausal osteoporosis. J Huazhong Univ Sci Technol [Medical Sci. 2005 Sep;25(5):527–9.
75. McClung MR, Geusens P, Miller PD, Zippel H, Bensen WG, Roux C, et al. Effect of Risedronate on the Risk of Hip Fracture in Elderly Women. N Engl J Med. 2001 Feb;344(5):333–40.
76. Mortensen L, Charles P, Bekker PJ, Digennaro J, Johnston CC. Risedronate Increases Bone Mass in an Early Postmenopausal Population: Two Years of Treatment Plus One Year of Follow-Up1. J Clin Endocrinol Metab. 1998 Feb 1;83(2):396–402.
77. Bell NH, Bilezikian JP, Bone III HG, Kaur A, Maragoto A, Santora AC. Alendronate Increases Bone Mass and Reduces Bone Markers in Postmenopausal African-American Women. J Clin Endocrinol Metab. 2002 Jun 1;87(6):2792–7.

Appendix 10, as supplied by the authors. Appendix to: Thériault G, Limburg H, Klarenbach S, et al. Recommendations on screening for primary prevention of fragility fractures. *CMAJ* 2023. doi: 10.1503/cmaj.221219. Copyright © 2023 The Author(s) or their employer(s). To receive this resource in an accessible format, please contact us at cmajgroup@cmaj.ca.

78. Hosking D, Adami S, Felsenberg D, Andia JC, Välimäki M, Benhamou L, et al. Comparison of change in bone resorption and bone mineral density with once-weekly alendronate and daily risedronate: a randomised, placebo-controlled study. Curr Med Res Opin. 2003 Jan 22;19(5):383–94.
79. Donaldson MG, Palermo L, Ensrud KE, Hochberg MC, Schousboe JT, Cummings SR. Effect of alendronate for reducing fracture by FRAX score and femoral neck bone mineral density: The fracture intervention trial. J Bone Miner Res. 2012 Aug;27(8):1804–10.
80. Hochberg MC, Thompson DE, Black DM, Quandt SA, Cauley J, Geusens P, et al. Effect of alendronate on the age-specific incidence of symptomatic osteoporotic fractures. J Bone Miner Res. 2005 Jun;20(6):971–6.
81. McClung MR, Lewiecki EM, Cohen SB, Bolognese MA, Woodson GC, Moffett AH, et al. Denosumab in Postmenopausal Women with Low Bone Mineral Density. N Engl J Med. 2006 Feb 23;354(8):821–31.
82. Fogelman I, Ribot C, Smith R, Ethgen D, Sod E, Reginster for the bmd-mn Study Grou J-Y. Risedronate Reverses Bone Loss in Postmenopausal Women with Low Bone Mass: Results From a Multinational, Double-Blind, Placebo-Controlled Trial [1]. J Clin Endocrinol Metab. 2000 May;85(5):1895–900.
83. Hooper MJ, Ebeling PR, Roberts AP, Graham JJ, Nicholson GC, D'Emden M, et al. Risedronate prevents bone loss in early postmenopausal women: a prospective randomized, placebo-controlled trial. Climacteric. 2005 Sep;8(3):251–62.
84. McClung M, Miller P, Recknor C, Mesenbrink P, Bucci-Rechtweg C, Benhamou C-L. Zoledronic Acid for the Prevention of Bone Loss in Postmenopausal Women With Low Bone Mass. Obstet Gynecol. 2009 Nov;114(5):999–1007.
85. Reid IR, Brown JP, Burckhardt P, Horowitz Z, Richardson P, Trechsel U, et al. Intravenous Zoledronic Acid in Postmenopausal Women with Low Bone Mineral Density. N Engl J Med. 2002 Feb 28;346(9):653–61.
86. Lewiecki EM, Miller PD, McClung MR, Cohen SB, Bolognese MA, Liu Y, et al. Two-Year Treatment With Denosumab (AMG 162) in a Randomized Phase 2 Study of Postmenopausal Women With Low BMD. J Bone Miner Res. 2007 Aug 16;22(12):1832–41.
87. Boonen S, Reginster J-Y, Kaufman J-M, Lippuner K, Zanchetta J, Langdahl B, et al. Fracture Risk and Zoledronic Acid Therapy in Men with Osteoporosis. N Engl J Med. 2012 Nov;367(18):1714–23.
88. Cummings SR, Martin JS, McClung MR, Siris ES, Eastell R, Reid IR, et al. Denosumab for prevention of fractures in postmenopausal women with osteoporosis. N Engl J Med. 2009 Aug;361(8):756–65.
89. Nct. Denosumab China Phase III Study [Internet]. Vol. 2021. 2016 [cited 2021 Mar 1]. Available from: https://www.cochranelibrary.com/central/doi/10.1002/central/CN-01479832/full and https://clinicaltrialsgov/show/NCT02014467
90. Zhu HM, Tang T, Cheng Q, He L, Li PQ, Xue QY, et al. Efficacy and Safety of Denosumab in Chinese Postmenopausal Women with Osteoporosis at Increased Risk of Fracture: Results From a 12-Month, Randomized, Double-blind, Placebo-controlled Phase III Study. Vol. 31, J Bone Miner Res. 2016. p. S160.
91. Pitale S, Thomas M, Rathi G, Deshmukh V, Kumar P, Reddy S, et al. A randomized placebo-controlled trial of the efficacy of denosumab in Indian postmenopausal women with osteoporosis. Indian J Endocrinol Metab. 2015 Jan;19(1):148–54.
92. Boonen S, Adachi JD, Man Z, Cummings SR, Lippuner K, Torring O, et al. Treatment with denosumab reduces the incidence of new vertebral and hip fractures in postmenopausal women at high risk. J Clin Endocrinol Metab. 2011 Jun;96(6):1727–36.
93. Bone HG, Bolognese MA, Yuen CK, Kendler DL, Wang H, Liu Y, et al. Effects of denosumab on bone mineral density and bone turnover in

Appendix 10, as supplied by the authors. Appendix to: Thériault G, Limburg H, Klarenbach S, et al. Recommendations on screening for primary prevention of fragility fractures. *CMAJ* 2023. doi: 10.1503/cmaj.221219. Copyright © 2023 The Author(s) or their employer(s). To receive this resource in an accessible format, please contact us at cmajgroup@cmaj.ca.

postmenopausal women. J Clin Endocrinol Metab. 2008 Jun;93(6):2149–57.
94. McCloskey E V, Johansson H, Oden A, Austin M, Siris E, Wang A, et al. Denosumab reduces the risk of osteoporotic fractures in postmenopausal women, particularly in those with moderate to high fracture risk as assessed with FRAX. J Bone Miner Res. 2012 Jul;27(7):1480–6.
95. McClung MR, Boonen S, Torring O, Roux C, Rizzoli R, Bone HG, et al. Effect of denosumab treatment on the risk of fractures in subgroups of women with postmenopausal osteoporosis. J Bone Miner Res. 2012 Jan;27(1):211–8.
96. Silverman S, Viswanathan HN, Yang Y-C, Wang A, Boonen S, Ragi-Eis S, et al. Impact of clinical fractures on health-related quality of life is dependent on time of assessment since fracture: results from the FREEDOM trial. Osteoporos Int. 2012 Apr 19;23(4):1361–9.
97. Orwoll E, Teglbjaerg CS, Langdahl BL, Chapurlat R, Czerwinski E, Kendler DL, et al. A randomized, placebo-controlled study of the effects of denosumab for the treatment of men with low bone mineral density. J Clin Endocrinol Metab. 2012 Sep;97(9):3161–9.
98. Merlijn T, Swart KMA, van der Horst HE, Netelenbos JC, Elders PJM. Fracture prevention by screening for high fracture risk: a systematic review and meta-analysis. Osteoporos Int. 2020 Feb 14;31(2):251–7.
99. Fink HA, MacDonald R, Forte ML, Rosebush CE, Ensrud KE, Schousboe JT, et al. Long-Term Drug Therapy and Drug Discontinuations and Holidays for Osteoporosis Fracture Prevention: A Systematic Review. Ann Intern Med. 2019 Nov;171(1):37–50.
100. Viswanathan M, Reddy S, Berkman N, Cullen K, Middleton JC, Nicholson WK, et al. Screening to Prevent Osteoporotic Fractures. JAMA. 2018 Jun 26;319(24):2532.
101. Crandall CJ, Newberry SJ, Diamant A, Lim Y-W, Gellad WF, Booth MJ, et al. Comparative effectiveness of pharmacologic treatments to prevent fractures: an updated systematic review. Ann Intern Med. 2014 Nov;161(10):711–23.
102. Crandall CJ, Newberry SJ, Gellad WG, Diamant A, Lim YW, Suttorp M, et al. Treatment to Prevent Fractures in Men and Women with Low Bone Density or Osteoporosis: Update of a 2007 Report. Comparative Effectiveness Review No. 53. (Prepared by Southern California Evidence-based Practice Center under Contract No. HHSA-290-2007-10062-I.). Rockville, MD: Agency for Healthcare Research and Quality; 2012.
103. Chen L-X, Ning G-Z, Zhou Z-R, Li Y-L, Zhang D, Wu Q-L, et al. The Carcinogenicity of Alendronate in Patients with Osteoporosis: Evidence from Cohort Studies. PLoS One. 2015 Apr 16;10(4):e0123080.
104. Davis S, Martyn-St James M, Sanderson J, Stevens J, Goka E, Rawdin A, et al. A systematic review and economic evaluation of bisphosphonates for the prevention of fragility fractures. Health Technol Assess (Rockv). 2016 Oct;20(78):1–406.
105. Kranenburg G, Bartstra JW, Weijmans M, de Jong PA, Mali WP, Verhaar HJ, et al. Bisphosphonates for cardiovascular risk reduction: A systematic review and meta-analysis. Atherosclerosis. 2016 Sep;252:106–15.
106. Lv F, Cai X, Yang W, Gao L, Chen L, Wu J, et al. Denosumab or romosozumab therapy and risk of cardiovascular events in patients with primary osteoporosis: Systematic review and meta- analysis. Bone. 2020 Nov;130:115121.
107. Diédhiou D, Cuny T, Sarr A, Norou Diop S, Klein M, Weryha G. Efficacy and safety of denosumab for the treatment of osteoporosis: A systematic review. Ann Endocrinol (Paris). 2015 Dec;76(6):650–7.
108. Davis S, Simpson E, Hamilton J, James MM-S, Rawdin A, Wong R, et al. Denosumab, raloxifene, romosozumab and teriparatide to prevent osteoporotic fragility fractures: a systematic review and economic evaluation. Health Technol Assess. 2020 Nov;24(29):1–314.

Appendix 10, as supplied by the authors. Appendix to: Thériault G, Limburg H, Klarenbach S, et al. Recommendations on screening for primary prevention of fragility fractures. *CMAJ* 2023. doi: 10.1503/cmaj.221219. Copyright © 2023 The Author(s) or their employer(s). To receive this resource in an accessible format, please contact us at cmajgroup@cmaj.ca.

109. Schünemann H, Brożek J, Guyatt G, Oxman A. GRADE Handbook. Vol. 2021. 2013.
110. Sheridan SL, Sutkowi-Hemstreet A, Barclay C, Brewer NT, Dolor RJ, Gizlice Z, et al. A Comparative Effectiveness Trial of Alternate Formats for Presenting Benefits and Harms Information for Low-Value Screening Services. JAMA Intern Med. 2016 Jan 1;176(1):31.
111. de Bekker-Grob EW, Essink-Bot ML, Meerding WJ, Pols HAP, Koes BW, Steyerberg EW. Patients' preferences for osteoporosis drug treatment: a discrete choice experiment. Osteoporos Int. 2008 Jul 8;19(7):1029–37.
112. Si L, Tu L, Xie Y, Palmer AJ, Gu Y, Zheng X, et al. Chinese patients' preference for pharmaceutical treatments of osteoporosis: a discrete choice experiment. Arch Osteoporos. 2019 Dec 31;14(1):85.
113. Hudson B, Toop L, Mangin D, Pearson J. Risk communication methods in hip fracture prevention: a randomised trial in primary care. Br J Gen Pract. 2011 Aug 1;61(589):e469–76.
114. Kalluru R, Petrie KJ, Grey A, Nisa Z, Horne AM, Gamble GD, et al. Randomised trial assessing the impact of framing of fracture risk and osteoporosis treatment benefits in patients undergoing bone densitometry. BMJ Open. 2017 Feb 10;7(2):e013703.
115. Billington EO, Feasel AL, Kline GA. At Odds About the Odds: Women's Choices to Accept Osteoporosis Medications Do Not Closely Agree with Physician-Set Treatment Thresholds. J Gen Intern Med. 2020 Jan 17;35(1):276–82.
116. Smallwood AJ, Schapira MM, Fedders M, Neuner JM. A pilot randomized controlled trial of a decision aid with tailored fracture risk tool delivered via a patient portal. Osteoporos Int. 2017 Feb 19;28(2):567–76.
117. Liu CS, Feasel AL, Kline GA, Billington EO. Pharmacotherapy decisions among postmenopausal women attending a group medical consultation or a one-on-one specialist consultation at an osteoporosis center: an observational cohort study. Osteoporos Int. 2021 Jul 18;32(7):1421–7.
118. LeBlanc A, Wang AT, Wyatt K, Branda ME, Shah ND, Van Houten H, et al. Encounter Decision Aid vs. Clinical Decision Support or Usual Care to Support Patient-Centered Treatment Decisions in Osteoporosis: The Osteoporosis Choice Randomized Trial II. PLoS One. 2015 May 26;10(5):e0128063.
119. Montori VM, Shah ND, Pencille LJ, Branda ME, Van Houten HK, Swiglo BA, et al. Use of a Decision Aid to Improve Treatment Decisions in Osteoporosis: The Osteoporosis Choice Randomized Trial. Am J Med. 2011 Jun;124(6):549–56.
120. Hudson B, Zarifeh A, Young L, Wells JE. Patients' Expectations of Screening and Preventive Treatments. Ann Fam Med. 2012 Nov 1;10(6):495–502.
121. Neuner JM, Schapira MM. Patient Perceptions of Osteoporosis Treatment Thresholds. J Rheumatol. 2014 Mar;41(3):516–22.
122. Andrews JC, Schunemann HJ, Oxman AD, Pottie K, Meerpohl JJ, Coello PA, et al. GRADE guidelines: 15. Going from evidence to recommendation-determinants of a recommendation's direction and strength. J Clin Epidemiol. 2013 Jul;66(7):726–35.
123. Klarenbach S, Sims-Jones N, Lewin G, Singh H, Theriault G, Tonelli M, et al. Recommendations on screening for breast cancer in women aged 40-74 years who are not at increased risk for breast cancer. C Can Med Assoc J. 2018;190(49):E1441-51.
124. Turner DA, Khioe RFS, Shepstone L, Lenaghan E, Cooper C, Gittoes N, et al. The Cost-Effectiveness of Screening in the Community to Reduce Osteoporotic Fractures in Older Women in the UK: Economic Evaluation of the SCOOP Study. J Bone Miner Res. 2018;33(5).
125. Söreskog E, Borgström F, Shepstone L, Clarke S, Cooper C, Harvey I, et al. Long-term cost-effectiveness of screening for fracture risk in a UK primary care setting: the SCOOP study. Osteoporos Int. 2020;31(8).

Appendix 10, as supplied by the authors. Appendix to: Thériault G, Limburg H, Klarenbach S, et al. Recommendations on screening for primary prevention of fragility fractures. *CMAJ* 2023. doi: 10.1503/cmaj.221219. Copyright © 2023 The Author(s) or their employer(s). To receive this resource in an accessible format, please contact us at cmajgroup@cmaj.ca.

126. The Ministry of Health and Long-Term Care (Ontario). Schedule of Facility Fees for Independent Health Facilities Under the Independent Health Facilities Act. Vol. 2017. 2015.
127. Osteoporosis Canada. Osteoporosis Canada. Provincial Drug Coverage. 2017.
128. CADTH. Common Drug Review: Denosumab (Prolia - Amgen Canada) Indication: Osteoporosis in Men [Internet]. 2015 [cited 2021 Mar 26]. Available from: https://www.cadth.ca/sites/default/files/cdr/complete/SR0414_cdr_complete_Prolia-Men_Sept-21-15-e.pdf
129. Jaglal S, Cameron C, Croxford R, MacKay C, Yasmin F. Ontario Osteoporosis Strategy - Provincial Performance Data for Osteoporosis Management Technical Report [Internet]. 2020 [cited 2021 Dec 6]. Available from: https://osteostrategy.on.ca/wp-content/uploads/Final-OOS-Provincial-Performance-Data-Technical-Report-Nov-18-20.pdf
130. CADTH. Guidelines for the Economic Evaluation of Health Technologies: Canada 4th Edition. Vol. 2021. 2017.
131. Cadarette SM, Gignac MAM, Jaglal SB, Beaton DE, Hawker GA. Access to Osteoporosis Treatment is Critically Linked to Access to Dual-Energy X-ray Absorptiometry Testing. Med Care. 2007 Sep;45(9):896–901.
132. Demeter S, Leslie WD, Lix L, MacWilliam L, Finlayson GS, Reed M. The effect of socioeconomic status on bone density testing in a public health-care system. Osteoporos Int. 2007 Feb;18(2):153–8.
133. Leslie WD. Clinical review: Ethnic differences in bone mass–clinical implications. J Clin Endocrinol Metab. 2012 Dec;97(12):4329–40.
134. Leslie WD, Brennan SL, Prior HJ, Lix LM, Metge C, Elias B. The post-fracture care gap among Canadian First Nations peoples: a retrospective cohort study. Osteoporos Int. 2012 Mar;23(3):929–36.
135. Lewiecki EM, Wright NC, Singer AJ. Racial disparities, FRAX, and the care of patients with osteoporosis. Osteoporos Int. 2020 Nov;31(11):2069–71.
136. Government of Canada. Osteoporosis and related fractures in Canada: Report from the Canadian Chronic Disease Surveillance System 2020. 2021 [cited 2021 Mar 26]; Available from: https://www.canada.ca/en/public-health/services/publications/diseases-conditions/osteoporosis-related-fractures-2020.html.
137. Statistics Canada. Access to a regular family physician [Internet]. 2021 [cited 2021 Mar 26]. Available from: https://www150.statcan.gc.ca/t1/tbl1/en/tv.action?pid=1310048401
138. Kendler DL, Adachi JD, Brown JP, Juby AG, Kovacs CS, Duperrouzel C, et al. A scorecard for osteoporosis in Canada and seven Canadian provinces. Osteoporos Int. 2021 Jan;32(1):123–32.
139. Munce SEP, Allin S, Carlin L, Sale J, Hawker G, Kim S, et al. Understanding Referral Patterns for Bone Mineral Density Testing among Family Physicians: A Qualitative Descriptive Study. J Osteoporos. 2016;2016:1–6.
140. Statistics Canada. Canadian Community Health Survey 2009 [Internet]. 2012 [cited 2021 Jul 16]. Available from: https://www23.statcan.gc.ca/imdb/p3Instr.pl?Function=assembleInstr&a=1&&lang=en&Item_Id=76867
141. Ministry of Health and Long-Term Care. Bone Mineral Density (BMD) Testing [Internet]. 2013 [cited 2021 Mar 26]. Available from: https://www.health.gov.on.ca/en/public/publications/ohip/bone.aspx
142. Siminoski K, Leslie WD, Frame H, Hodsman A, Josse RG, Khan A, et al. Recommendations for bone mineral density reporting in Canada. Can Assoc Radiol J [Internet]. 2005 Jun;56(3):178–88. Available from: https://ovidsp.ovid.com/ovidweb.cgi?T=JS&CSC=Y&NEWS=N&PAGE=fulltext&D=med6&AN=16144280;

Appendix 10, as supplied by the authors. Appendix to: Thériault G, Limburg H, Klarenbach S, et al. Recommendations on screening for primary prevention of fragility fractures. *CMAJ* 2023. doi: 10.1503/cmaj.221219. Copyright © 2023 The Author(s) or their employer(s). To receive this resource in an accessible format, please contact us at cmajgroup@cmaj.ca.

Appendix 10, as supplied by the authors. Appendix to: Thériault G, Limburg H, Klarenbach S, et al. Recommendations on screening for primary prevention of fragility fractures. *CMAJ* 2023. doi: 10.1503/cmaj.221219. Copyright © 2023 The Author(s) or their employer(s). To receive this resource in an accessible format, please contact us at cmajgroup@cmaj.ca.
