# Supplementary material for: Recommendations on screening for primary prevention of fragility fractures
Source: CMAJ. 2023 May 8;195(18):E639–49. doi: 10.1503/cmaj.221219 (PMC10166624; doi:10.1503/cmaj.221219)
Supplement: Appendix 11: Task Force Public Advisors Network (TFPAN) Protocol [file 221219-guide-11-at.pdf]

Task Force Public Advisors Network (TF-PAN) Protocol

Prepared By:
Knowledge Translation Program
Li Ka Shing Knowledge Institute
St. Michael's Hospital

Contacts:

Vanja Grubac | Research Coordinator

E: Vanja.Grubac@unityhealth.to

Sarah Deshpande | Research Assistant

E: Sarah.Deshpande@unityhealth.to

Appendix 11, as supplied by the authors. Appendix to: Thériault G, Limburg H, Klarenbach S, et al. Recommendations on screening for primary prevention of fragility fractures.
CMAJ 2023. doi: 10.1503/cmaj.221219. Copyright © 2023 The Author(s) or their employer(s).
To receive this resource in an accessible format, please contact us at cmajgroup@cmaj.ca.

Table of Contents

Table of Contents 2
Background and Rationale 3
Methods 4
  Objectives 4
  Recruitment 4
    Recruitment Strategy 4
    Recruitment Materials 5
  Panel Selection: 5
  Orientation and Training 5
  TF-PAN Activities 7
    Community Jury Approach 7
  Compensation 10
Evaluation of the Network 10
  Purpose 11
  Objectives 11
  Process Evaluation Indicators 12
  Outcome Evaluation Indicators 13
  References: 14
Appendix 16

Appendix 11, as supplied by the authors. Appendix to: Thériault G, Limburg H, Klarenbach S, et al.
Recommendations on screening for primary prevention of fragility fractures.
CMAJ 2023. doi: 10.1503/cmaj.221219. Copyright © 2023 The Author(s) or their employer(s).
To receive this resource in an accessible format, please contact us at cmajgroup@cmaj.ca.

Background and Rationale

Involving patients in the development of clinical practice guidelines can yield recommendations that are more likely to be patient-centered, practical, and can provide opportunities for shared decision-making.[1,4-8] For guideline developers, patient involvement may enhance the credibility, transparency, and applicability of clinical practice guidelines (CPGs). Numerous international organizations that appraise the quality of CPGs, explicitly call for patient involvement in the guideline development process.[5-7] Similarly, findings from a literature review conducted by The National Institute for Health and Care Excellence,[8] suggested that involving patients earlier in the guideline development process and asking how they would like to be engaged can improve the effectiveness of participants' contributions, as well as influence guideline scope and improve inclusion of patient-relevant topics.[8,9,14-18] Despite the benefits of involving patients in the guideline development process, adequate training and support are commonly cited as barriers to patient involvement.[8,9,14-18]

The Canadian Task Force for Preventive Health Care (Task Force) continues to engage the support of the Knowledge Translation (KT) team at St. Michael's Hospital to explore ways to involve patients and members of the public in its guideline development process. Results from nine engagement surveys completed by patients and administered as part of the existing patient preferences process during guideline development showed patients were unclear about the value and impact of their contributions and identified gaps in their knowledge, required to contribute meaningfully in the process. During the in-person Task Force meeting in February 2019, the KT team presented opportunities to improve and expand on the Task Force's existing patient and public engagement process. Additionally, considering the Task Force's prioritization on shared decision-making regarding preventive health care interventions, it is crucial to explore opportunities to meaningfully engage members of the public and to elicit their values and preferences for such interventions in order to inform guideline development.

The **Task Force Public Advisors Network (TF-PAN)** is an initiative to encourage early and meaningful engagement of members of the public with the Task Force by seeking their input throughout the development and dissemination of Task Force guidelines. Unlike the traditional Task Force patient preferences model, TF-PAN members learn background information on what the Task Force does and the types of methods/processes used to develop preventive health care guidelines in order to ensure informed participation in guideline development. TF-PAN members will form a stakeholder consultation group and will provide input on various phases of guideline development, as determined by the guideline Working Group chairs based on need and guideline context. In this protocol, we provide an overview of how TF-PAN will be established, operationalized, and evaluated.

Appendix 11, as supplied by the authors. Appendix to: Thériault G, Limburg H, Klarenbach S, et al. Recommendations on screening for primary prevention of fragility fractures.
CMAJ 2023. doi: 10.1503/cmaj.221219. Copyright © 2023 The Author(s) or their employer(s).
To receive this resource in an accessible format, please contact us at cmajgroup@cmaj.ca.

Methods

Objectives

The TF-PAN encourages early, meaningful and effective engagement with members of the public by seeking their input throughout the development and dissemination of Task Force guidelines. Specifically, TF-PAN members will:

1. Be made aware of the Task Force mandate, organizational structure, and processes for guideline development
2. Be able to provide input on the development, dissemination, and implementation of Task Force guidelines

The KT team will evaluate the implementation quality of the TF-PAN. Findings will be used to iteratively refine the initiative and processes. (See Evaluation).

Recruitment

Recruitment Strategy

The KT team will recruit the TF-PAN network, which will be composed of a group (n = 15-20) patients, caregivers, and members of the public (defined in this context as individuals who are not in clinical practice or former primary care professionals). The membership term will be one year with the potential for renewal (membership will be reviewed annually and recruitment undertaken to refresh the membership pool every year or as needed). The following methods will be used to recruit TF-PAN members:

1. Emailing members of the public who are part of the KT team's Patient Preferences database. This is a database of >200 patient partners that have previously participated in Task Force patient preferences projects and indicated that they would be interested in participating in future Task Force patient engagement initiatives.
2. Posting social media recruitment ads
3. Posting recruitment posters on the Task Force website and Task Force newsletter
4. Emailing external organizations (e.g., SPOR Evidence Alliance) to request dissemination of recruitment posters
5. Emailing Task Force members to post social media recruitment ads on their social media accounts and to disseminate recruitment posters to their relevant networks

The KT team will employ several additional recruitment strategies to specifically recruit French-speaking participants including: posting on Facebook Marketplace, posting to Facebook Volunteer/Research Volunteer Groups, and outreach to French-speaking public and patient groups.

Initial recruitment occurred from September 11th to October 11th 2020 and recruitment strategies will be reviewed and adapted and/or adjusted to inform recruitment in future years.

Appendix 11, as supplied by the authors. Appendix to: Thériault G, Limburg H, Klarenbach S, et al. Recommendations on screening for primary prevention of fragility fractures.
CMAJ 2023. doi: 10.1503/cmaj.221219. Copyright © 2023 The Author(s) or their employer(s).
To receive this resource in an accessible format, please contact us at cmajgroup@cmaj.ca.

Recruitment Materials

The KT team will engage with the Task Force Communications team to develop recruitment materials in English and French, including recruitment posters, email templates, social media posts, and Task Force newsletter posts.

The recruitment posters, email templates, and social media posts will direct any interested participants to the TF-PAN website page. From the website page, participants will be required to complete an application survey.

Panel Selection:

Interested participants will complete a TF-PAN application and demographic survey. The survey will be available in English and in French and will assess demographic factors (gender, age, geographic location, region, race/ethnicity, education level) and why participants are interested in joining TF-PAN. Participants will be asked to disclose any potential conflicts of interest. When selecting TF-PAN members, the KT Team will aim to select a panel that is diverse as possible in as many ways as possible, specifically considering the intersectionality of applicant gender, language (English and French), race-ethnicity, education, geographic location, and age. We will also reserve at least 15% of seats for French-speaking participants. To ensure feasibility, panel selection will consider demonstrated interest and motivation to participate and capacity to commit to the time requirements for TF-PAN training and activities.

Potential participants will be contacted by email, and members of the KT team will schedule brief interviews (via phone/video conference) to explain the terms of participation and to confirm interest. During this conversation, the KT team will discuss the preliminary terms of reference and the anticipated time commitment for required activities. If a participant is unable to commit to these activities or if they no longer demonstrate interest in the TF-PAN, the KT team will reach out to additional qualified applicants and repeat the confirmation call process.

Applicants that were not selected will be notified by email and asked whether they would like to be part of the Extended TF-PAN, which is a pool of members who are available to participate in future ad-hoc Task Force patient preferences related activities (e.g., participate in outcome ratings).

Orientation and Training

TF-PAN members will be invited to attend a welcome orientation session. At this session, members will be introduced to Task Force leadership, KT team members, and each other. The KT team will lead the participants in teamwork activities to encourage rapport building amongst participants. A reference document will be provided to clearly outline roles and responsibilities, expectations, meeting frequency, a communication plan, and decision-making protocols. The communication plan will encourage regular and ongoing communication, tailored to the needs of the members. For instance, the communication plan may include engaging network members in monthly teleconferences with the project team, providing opportunities for additional capacity-building opportunities, providing Task Force and TF-PAN updates, exploring TF-PAN members' research or dissemination ideas, or providing feedback on how to optimize KT products and tools. The terms of reference, communication plan, and decision-making protocols will be revised iteratively based on evaluation of the TF-PAN pilot cohort.

Based on best practices for meaningful patient engagement and adult education theory,[19], we will provide members of the network with relevant training (via live and recorded webinars). The

Appendix 11, as supplied by the authors. Appendix to: Thériault G, Limburg H, Klarenbach S, et al.
Recommendations on screening for primary prevention of fragility fractures.
CMAJ 2023. doi: 10.1503/cmaj.221219. Copyright © 2023 The Author(s) or their employer(s).
To receive this resource in an accessible format, please contact us at cmajgroup@cmaj.ca.

goals of this training are to educate panel members about the Task Force (including a high-level overview of Task Force methodology), preventive health care concepts and terms, patient and public engagement, and TF-PAN activities. The overarching goal of the training provided to TF-PAN members is to ensure that they are equipped with the knowledge to provide relevant and informed feedback on upcoming Task Force guidelines (see Community Juries). Training will be divided into 4 required topics and one optional topic:

Required

1. **Introduction to the Task Force**
   Learning Objectives:
   a. Discuss the Task Force mandate, history, membership, and structure
   b. Identify the goal of clinical practice guidelines
   c. Review commonly used Task Force guidelines
   d. Describe patient/public involvement in Task Force guideline development
2. **Introduction to TF-PAN**
   Learning Objectives:
   a. Describe the roles and responsibilities of TF-PAN members
   b. Discuss the TF-PAN terms of reference
   c. Describe community juries and why they are important
   d. Describe how community juries will be run with TF-PAN
3. **What are Preventive Care Services?**
   Learning Objectives
   a. Define preventive health care and describe what the Task Force evaluates under preventive health care
   b. Describe principles of interventions to reduce risk (e.g., obesity, tobacco cessation)
   c. Describe principles of screening interventions (e.g., breast cancer)
4. **How does the Task Force Evaluate Evidence to Make a Recommendation?**
   Learning Objectives
   a. Identify factors that contribute to guideline trustworthiness
   b. Describe the rationale for using evidence to inform decision making
   c. Describe steps to guideline development
   d. Describe how the Task Force “weighs” harms and benefits
   e. Describe GRADE and why we use it

Optional

5. **Patient and Public Engagement [optional]**
   Learning Objectives
   a. Define patient and public engagement
   b. Distinguish between meaningful and tokenistic engagement
   c. Discuss the importance of meaningful engagement
   d. Identify common barriers and facilitators to engagement

To ensure participant engagement, further explain the required topics, and facilitate rapport among TF-PAN members, the KT team and Task Force members will facilitate live sessions based on the outlined topics and will follow best practices for adult education learning (e.g., interactive activities, opportunities to ask questions).

The live presentations will be recorded and uploaded to the TF-PAN website as modules so participants can easily access them at their own convenience. Additionally, training slide decks

Appendix 11, as supplied by the authors. Appendix to: Thériault G, Limburg H, Klarenbach S, et al. Recommendations on screening for primary prevention of fragility fractures.
CMAJ 2023. doi: 10.1503/cmaj.221219. Copyright © 2023 The Author(s) or their employer(s).
To receive this resource in an accessible format, please contact us at cmajgroup@cmaj.ca.

for each module will be made available on the Task Force website. All resources will be written and presented in plain language to foster participant understanding. All resources will be available in French and English.

TF-PAN Activities

We will aim to optimize value for TF-PAN members by providing concrete opportunities to contribute to project directions in the guideline development process. Network members may choose to take part in particular activities based on their interest and availability. This means we can have different perspectives at different points in the process, and participants can choose how they want to engage. At minimum, we will look to engage members in three ways:

1. Participate in welcome orientation session to meet Task Force leadership, KT team members, and each other.
2. Participate in the training sessions on the Task Force, preventive health care concepts, and patient engagement (live sessions that will be recorded and posted online for TF-PAN members to access if they are unable to attend).
3. Participate in at least two Community Jury sessions per year to provide guideline specific input on public or patient preferences (note: this is the new approach to the ‘Patient Preferences’ methodology)

Although participating in Community Jury sessions will be the main activity TF-PAN members may optionally participate in other activities, such as: guideline or KT tool dissemination activities (e.g., providing input on media materials, identifying channels and networks for dissemination, or sharing materials through their own channels and networks etc.) or KT tool development (e.g., participating in tool usability testing).

Each TF-PAN member will be connected with a KT team research assistant to reach out to with any questions before and after meetings. Continued training and education will be offered to participants as needed.

Extended TF-PAN members, who are individuals in the TF-PAN applicant pool who demonstrated interest in TF-PAN but were not selected to participate in Community Jury sessions, may have the opportunity to participate in various ad-hoc patient preferences tasks. These might include activities such as Task Force guideline outcome ratings or KT tool usability testing on an as-needed basis, when these services are not feasible for the TFPAN community jury to undertake.

In order to ensure that TF-PAN members are up-to-date on the progress of the various Task Force Guideline Working Groups, working groups will fill out quarterly updates which the KT team will ensure is in plain language and emailed out to TF-PAN members in the form of a quarterly email newsletter. Other forms of updates will be explored, including virtual meetings or pre-recorded videos.

Community Jury Approach

The TF-PAN will be a transition from the previous Task Force patient preferences engagement approach. In the latter approach, individuals who reflected the guideline target population (e.g., women for asymptomatic bacteriuria screening) were engaged to share their values and

Appendix 11, as supplied by the authors. Appendix to: Thériault G, Limburg H, Klarenbach S, et al. Recommendations on screening for primary prevention of fragility fractures.
CMAJ 2023. doi: 10.1503/cmaj.221219. Copyright © 2023 The Author(s) or their employer(s).
To receive this resource in an accessible format, please contact us at cmajgroup@cmaj.ca.

preferences on screening outcomes to inform the guideline recommendation. In contrast, the TF-PAN panel will share values and preferences on all guidelines, even ones for which they do not individually reflect the target population. In this way, TF-PAN provides a population-level perspective to elicit feedback on perceptions of harms and benefits.

A community jury (modified terminology regarding a citizen's jury) approach will be used to engage TF-PAN members to provide input on guidelines. A community jury is a form of deliberative democracy and aims to elicit an *informed* community perspective on important and potentially controversial topics. Community jury participants are provided with expert presentations and opportunities to question the experts, engage in both facilitated and private deliberation, and are asked to form a consensus or majority 'verdict' on the topic question[20]. While first developed in the 1970s by the Jefferson Centre (United States), community juries have been used successfully in research to elicit informed perspectives for several health policy issues, for example screening (mammography, prostate cancer), case finding for dementia, and overdiagnosis[21]. Recently, Thomas et al. demonstrated that evidence-informed deliberation of the harms and benefits of PSA screening through a community jury approach impacted men's individual choice to be screened for prostate cancer. The research team concluded that community juries may be a valid method for eliciting target group input to policy decisions[22].

Community juries will be used to elicit TF-PAN members' perceptions on the domains of equity, feasibility, and cost in addition to the balance of benefits and harms. These judgements can be used to inform the direction and strength of Task Force recommendations as well as inform nuanced considerations around patient preferences that can be written into the guideline and KT Tools. Community juries will allow TF-PAN to be flexible in its ability to meet Task Force Guideline Working Group patient preferences needs, across the guideline development spectrum. Timing of and type of input will vary depending on needs as identified by the guideline working group chairs. Examples of input community juries to provide public feedback include:

- Providing input on the importance of which outcomes to consider for the systematic review
- Reviewing guideline key message statements for patients and public
- Providing feedback on findings from studies on patient preferences
- Providing a population perspective regarding benefits *relative* to harms when considering undergoing a preventive health care service or not
- Qualitative context for prioritizing benefits relative to harms when considering undergoing a preventive health care service
- Rating/Ranking the importance of different outcomes (harms and benefits of screening) from a population perspective
- Providing considerations around barriers and facilitators to screening from a population perspective
- Identifying 'key questions' or important information required to support shared decision making during recommendation implementation
- Providing input on how patient preferences should be incorporated into a guideline
- Providing input on inclusive language

Appendix 11, as supplied by the authors. Appendix to: Thériault G, Limburg H, Klarenbach S, et al. Recommendations on screening for primary prevention of fragility fractures.
CMAJ 2023. doi: 10.1503/cmaj.221219. Copyright © 2023 The Author(s) or their employer(s).
To receive this resource in an accessible format, please contact us at cmajgroup@cmaj.ca.

Thus, community juries can solicit feedback from TF-PAN at different points along the guideline development pathway. At TF-PAN launch, each guideline-working group can engage the TF-PAN to participate in one community jury per guideline. If a Guideline Working Group is interested in outcome ratings and determines that a TF-PAN community jury is not appropriate or suitable for outcome ratings, they can request the traditional outcome ratings method (i.e., recruit population-specific participants to rank outcomes). Recruitment for this particular activity will be offered to eligible Extended TF-PAN members. Guideline Working Groups will be asked to assess their patient engagement data needs and communicate them to the KT team once the topic is identified and key questions are being considered, so that appropriate timelines can be developed. Setting up a community jury session will take approximately 4-5 weeks of planning by the KT team.

Running a Community Jury

Community juries elicit the voices, values, and preferences of informed citizens who are presented with as systematically synthesized evidence. The sessions are structured to be a two-way dialogue with a 'topic' on trial. 'Jurors' then get a chance to deliberate the evidence before formulating opinions and recommendations. The structure of a session has two phases:

1. Educating Participants
2. Deliberation

In Phase 1, the KT team and the Guideline Working Group will collaborate to develop questions or goals for the community jury to guide the session. Each Guideline Working Group will determine these questions or goals. An example of a question is as follows: *"Should women between 40 to 49 years of age be encouraged to discuss their interest in breast cancer screening with their primary care provider?"* The KT team and Task Force Guideline Working Group Chair will co-develop a presentation that describes the intervention being evaluated and how the condition would be addressed without the intervention and will outline relevant outcomes, harms, and benefits as identified in the evidence. Additionally, participants will be allowed to consider the societal impact of a recommendation through information about equity, cost, and public access to a service, such that a diverse but informed range of potentially conflicting perspectives are considered.

Guideline Working Group Chairs and Co-Chairs will be responsible for presenting relevant information to TF-PAN members. It is critical that TF-PAN members are able to ask questions and seek clarification during this phase and structured time for questioning will be built into these presentations. The factor that distinguishes deliberative methods from other forms of public engagement is the process of iterative, two-way exchange between members of the public and topic experts.

In Phase 2, a patient engagement facilitator from the KT team will work with TF-PAN members in a structured session to produce the desired output for the session. The KT facilitator will follow a semi-structured discussion guide (developed in consultation with a community jury expert and Working Group Chair), in order to facilitate fair participant interactions. Guideline Working Group Chairs will be invited to attend the Phase 2 session, but will be placed in a separate breakout room during the deliberation session. Guideline Working Group Chairs will be

Appendix 11, as supplied by the authors. Appendix to: Thériault G, Limburg H, Klarenbach S, et al. Recommendations on screening for primary prevention of fragility fractures.
CMAJ 2023. doi: 10.1503/cmaj.221219. Copyright © 2023 The Author(s) or their employer(s).
To receive this resource in an accessible format, please contact us at cmajgroup@cmaj.ca.

invited to the main room to answer any questions and provide clarification to TF-PAN members, when needed. This will help to ensure that TF-PAN members do not experience any undue influence during the deliberation session, but receive the necessary information and clarification from Guideline Working Group Chairs. All outputs during this discussion will be recorded and are intended to reflect a broad view of the topic that addresses all issues considered important by participants.

The two phases will be conducted over a two-week period. Phase 1, Educating Participants, will be 1-2 hours long depending on the content presented. Phase 2, Deliberation, will be approximately 3-hours long, and will be held one week after Phase 1. This will allow participants to reflect on information presented in Phase 1 and to formulate thoughtful, informed opinions or questions to bring to Phase 2. If participants require further time for deliberation, an extra Phase 2 session will be rescheduled no more than a 1 week after the first. The length of this session will be determined by the TF-PAN staff, based on an estimate of how much more deliberation the group needs to complete the activity.

Given the pan-Canadian nature of TF-PAN, community juries will be held via videoconference. We therefore purposefully limited the amount of time spent per session (maximum two hours) to reduce participant burden and 'Zoom fatigue'. The KT team will track and iteratively refine the structure and timing of the sessions based on participant and working group chairs' feedback.

An important aspect of the patient/public feedback activities of TF-PAN will be to ensure that members are aware of how their input is being used. Therefore, after the community jury feedback is delivered to and used by the working group, the KT team will work with the Guideline Working Group Chair, Co-chair, and Science Team Lead to report back to each community jury group as to how exactly their input was used in the guideline development. The format of this reporting will be determined by the community jury group and may be a report, an email update, or a video update.

Compensation

TF-PAN members will be compensated at a rate of $25 per hour. If any engagement requires less than one hour in duration, the full hour will be compensated. A minimum and maximum number of hours required for annual TF-PAN activities will be discussed and agreed upon in advance upon onboarding.

TF-PAN members are responsible for tracking and claiming their own hours of contribution using the standardized form found on the TF-PAN website. TF-PAN members will be informed on expected hours per activity. Members will be compensated bi-annually upon submission of their completed forms. All payments will be mailed out in the form of cheque or gift card.

Evaluation of the Network

Please note: This is a preliminary outline. A separate evaluation protocol to include a more detailed methodology for evaluation of the TF-PAN processes and outcomes.

Appendix 11, as supplied by the authors. Appendix to: Thériault G, Limburg H, Klarenbach S, et al.
Recommendations on screening for primary prevention of fragility fractures.
CMAJ 2023. doi: 10.1503/cmaj.221219. Copyright © 2023 The Author(s) or their employer(s).
To receive this resource in an accessible format, please contact us at cmajgroup@cmaj.ca.

Purpose

Process evaluations determine whether program activities have been implemented as intended. Conducting a process evaluation of the TF-PAN can help identify strengths to build upon and challenges to address, as well as inform potential future next steps for the Network. Outcome evaluations determine how well a program achieved its objectives by measuring results. Conducting an outcome evaluation can help identify if TF-PAN is meeting its intended objectives.

The purpose of this evaluation is to assess TF-PAN membership, TF-PAN members' perceptions of the training they received, how they were engaged, and suggestions for improvement. Additionally, we're looking to evaluate if TF-PAN members were made aware of the Task Force mandate, organizational structure, and processes for guideline development and if members were able to provide meaningful and effective input on the development, dissemination, and implementation of Task Force guidelines.

The KT team will complete a process and outcome evaluation during year one of the network, by conducting surveys and interviews with TF-PAN members, as well as Task Force and Public Health Agency of Canada members who have engaged with the TF-PAN, if applicable.

Objectives

The objectives of this process evaluation are:

1. Determine the reach of TF-PAN (who applied to TF-PAN and who was selected?)
2. Determine perceptions of the training experienced by TF-PAN members
3. Determine quality of implementation of the community juries (e.g. the skill with which facilitators deliver material and interact with participants, the performance of technology used, etc.)
4. Evaluate perceptions of TF-PAN members' regarding the degree of successful engagement
5. Evaluate perceptions of Task Force and Public Health Agency of Health members regarding using TF-PAN for public engagement
6. Collect suggestions for program improvement

The objectives of this outcome evaluation are:

1. Evaluate how TF-PAN input was utilized in Task Force guideline development, dissemination, and implementation (i.e. the types of decisions they were involved in, how their input was used, etc.)
2. Determine the impact of the TF-PAN training on members' knowledge of Task Force and preventive health care concepts

Appendix 11, as supplied by the authors. Appendix to: Thériault G, Limburg H, Klarenbach S, et al.
Recommendations on screening for primary prevention of fragility fractures.
CMAJ 2023. doi: 10.1503/cmaj.221219. Copyright © 2023 The Author(s) or their employer(s).
To receive this resource in an accessible format, please contact us at cmajgroup@cmaj.ca.

Process Evaluation Indicators

Research question	Potential Measures	Data Source
RQ1. What was the reach of the TF-PAN pilot?	# of TF PAN members interested, demographics, # included on panel, # in extended panel, reasons for exclusion of interested participants, participant retention over time, reasons for attrition	Administrative Records
RQ2. What were the perceptions of the TF-PAN members on the training that they received?	# of training sessions attended, mandatory/optional session attended, quality of delivery, participant engagement, tailoring to participants done, participant perceptions of content, suggestions for improvement	Survey (after training sessions)<br><br>Survey (after community jury)
RQ3. What was the implementation quality of the community juries?	# of community juries done, length, participant satisfaction with presenter/facilitator, participant perceptions of community jury outcome, TF-PAN members' satisfaction/perceptions, suggestions for improvement	Survey (after each community jury)<br><br>Interview (with members)
RQ4. What were the members' perceptions of patient engagement	Respect, trust, legitimacy, fairness, competence, accountability	Survey (after training sessions)<br><br>Survey (after each community jury)<br><br>Interview (with members)
RQ5. Evaluate perceptions of Task Force and Public Health Agency of Health members regarding using TF-PAN for public engagement	# of activities completed, satisfaction with activities, satisfaction with scheduling process, satisfaction with outputs	Survey, Interview (with staff)

Appendix 11, as supplied by the authors. Appendix to: Thériault G, Limburg H, Klarenbach S, et al. Recommendations on screening for primary prevention of fragility fractures.
CMAJ 2023. doi: 10.1503/cmaj.221219. Copyright © 2023 The Author(s) or their employer(s).
To receive this resource in an accessible format, please contact us at cmajgroup@cmaj.ca.

Outcome Evaluation Indicators

Research question	Potential Measures	Data Source
RQ1. How was TF-PAN input was used in Task Force guideline development, dissemination, and implementation?	types of activities they were involved in, how their input was used in guidelines	Administrative Records<br><br>Interview (with staff)
RQ2. What was the impact of the TF-PAN training on members' knowledge about Task Force and preventive health care concepts?	knowledge of Task Force history, guidelines, methods, KT tools, critical appraisal,	Survey (after training sessions)

Appendix 11, as supplied by the authors. Appendix to: Thériault G, Limburg H, Klarenbach S, et al. Recommendations on screening for primary prevention of fragility fractures.
CMAJ 2023. doi: 10.1503/cmaj.221219. Copyright © 2023 The Author(s) or their employer(s).
To receive this resource in an accessible format, please contact us at cmajgroup@cmaj.ca.

References:

1) IKT Research Network. (n.d.) Retrieved from URL: https://iktrn.ohri.ca/

2) Graham, I.D., Korthari, A., & McCutcheon, C. Moving knowledge into action for more effective practice, programmes, and policy: protocol for a research programme on integrated knowledge translation. *Implementation Science.* 2018; 13:22 doi: 10.1186/s13012-017-0700-y

3) Kothari A, McCutcheon C, Graham I.D. Defining integrated knowledge translation and moving forward:a response to recent commentaries. *Kerman Univ Med Sci.* 2017;6:299–300.https://doi.org/10.15171/ijhpm.2017.15.

4) Canadian Institutes of Health Research. (2014, July). Strategy for Patient-Oriented Research - Patient Engagement Framework. (retrieved from: http://www.cihr-irsc.gc.ca/e/48413.html#a7)

5) Institute of Medicine . Clinical practice guidelines we can trust. Washington: The National Academies Press; 2011. [Google Scholar]

6) Brouwers MC, Kho ME, Browman GP, Burgers JS, Cluzeau F, Feder G, et al. AGREE II: Advancing guideline development, reporting and evaluation in healthcare. Can Med Assoc J. 2010;182:e839–42. doi: 10.1503/cmaj.090449.

7) Qaseem A, Forland F, Macbeth F, Ollenschläger G, Phillips S, van der Wees P, et al. Guidelines International Network: Toward International Standards for Clinical Practice Guidelines. Ann Intern Med. 2012;156:525–531. doi: 10.7326/0003-4819-156-7-201204030-00009

8) National Institute for Health and Care Excellence (NICE). Improving how patients and the public can help develop NICE guidance and standards. 2016. Retrieved from: https://www.nice.org.uk/Media/Default/About/NICE-Communities/Public-involvement/public-involvement-review-consultation-paper.pdf

9) Rashid, A., Thomas, V., Shaw, T. et al. Patient and public involvement in the development of healthcare guidance: An overview of current methods and future challenges. *The Patient.* 2017;10(3), 277-282. https://doi.org/10.1007/s40271-016-0206-8

10) Roman BR, Feingold J. Patient-centered guideline development best practices can improve the quality and impact of guidelines.*Otolaryngol Head Neck Surg*. 2014;151(4):530–2.

11) Facey KM, Hansen HP. Patient-focused HTAs. *Int J Technol Assess Health Care*. 2011;27(4):273–4.

12) Krahn M, Naglie G. The next step in guideline development: incorporating patient preferences. *JAMA*. 2008;300(4):436–8

13) Bridges JF, Jones C. Patient-based health technology assessment: a vision of the future. *Int J Technol Assess Health Care*.2007;23(1):30–5.

14) Black, A., Strain, K., Wallsworth, C., Chalrton, S-G., Chang, W., McNamee, K., & Hamilton, C. What constitutes meaningful engagement for patients and families as partners on research teams? *Journal of Health Services Research & Policy.* 2018;23(3), 158-167. doi: 10.1177/1355819618762960

Appendix 11, as supplied by the authors. Appendix to: Thériault G, Limburg H, Klarenbach S, et al. Recommendations on screening for primary prevention of fragility fractures.
CMAJ 2023. doi: 10.1503/cmaj.221219. Copyright © 2023 The Author(s) or their employer(s).
To receive this resource in an accessible format, please contact us at cmajgroup@cmaj.ca.

15) Armstrong, M.J., Mullins, C.D., Gronseth, G.S., Gagliardi, A.R. Impact of patient involvement on clinical practice guideline development: a parallel group study. *Implementation Science*. 2018;13:55 DOI: https://doi.org/10.1186/s13012-018-0745-6

16) Armstrong, M.J., Rueda, J.D., Gronseth, G.S., & Mullins, C.D. Framework for enhancing clinical practice guidelines through continuous patient engagement. *Health Expectations.* 2016;20, 3-10. doi: 10.1111/hex.12467

17) Holmes, B.J., Bryan, S., Ho, K., and McGavin, C. Engaging patients as partners in health research: Lessons from BC, Canada. *Healthcare Management Forum*. 2018;31(2) 41-44.doi: 10.1177/0840470417741712

18) Brouwers, M.C., Vukmirovic, M., Spithoff, K., and Makarski, J. Understanding optimal approaches to patient and caregiver engagement in the development of cancer practice guidelines; a mixed methods study. *BMC Health Services Research*. 2017; 17:186

19) Brouwers, M.C., et al. Engaging cancer patients in clinical practice guideline development: a pilot study. *Current Oncology*. 2018; 25(4): 250

20) Thomas R, Scott AM, Sims R, et al. Exploring women's priorities for the potential consequences of a gestational diabetes diagnosis: A pilot community jury. Health Expectations : an International Journal of Public Participation in Health Care and Health Policy. 2020 Jun;23(3):593-602. DOI: 10.1111/hex.13036.

21) **Street, J., Duszynski, K., Krawczyk, S., & Braunack-Mayer, A. (2014). The use of citizens' juries in health policy decision-making: a systematic review. Social science & medicine, 109, 1-9.**

22) Thomas, R., Glasziou, P., Rychetnik, L., Mackenzie, G., Gardiner, R., & Doust, J. (2014). Deliberative democracy and cancer screening consent: a randomised control trial of the effect of a community jury on men's knowledge about and intentions to participate in PSA screening. BMJ open, 4(12), e005691. https://doi.org/10.1136/bmjopen-2014-005691

Appendix 11, as supplied by the authors. Appendix to: Thériault G, Limburg H, Klarenbach S, et al. Recommendations on screening for primary prevention of fragility fractures.
CMAJ 2023. doi: 10.1503/cmaj.221219. Copyright © 2023 The Author(s) or their employer(s).
To receive this resource in an accessible format, please contact us at cmajgroup@cmaj.ca.

Appendix

<u>TF-PAN Educational Outline</u>

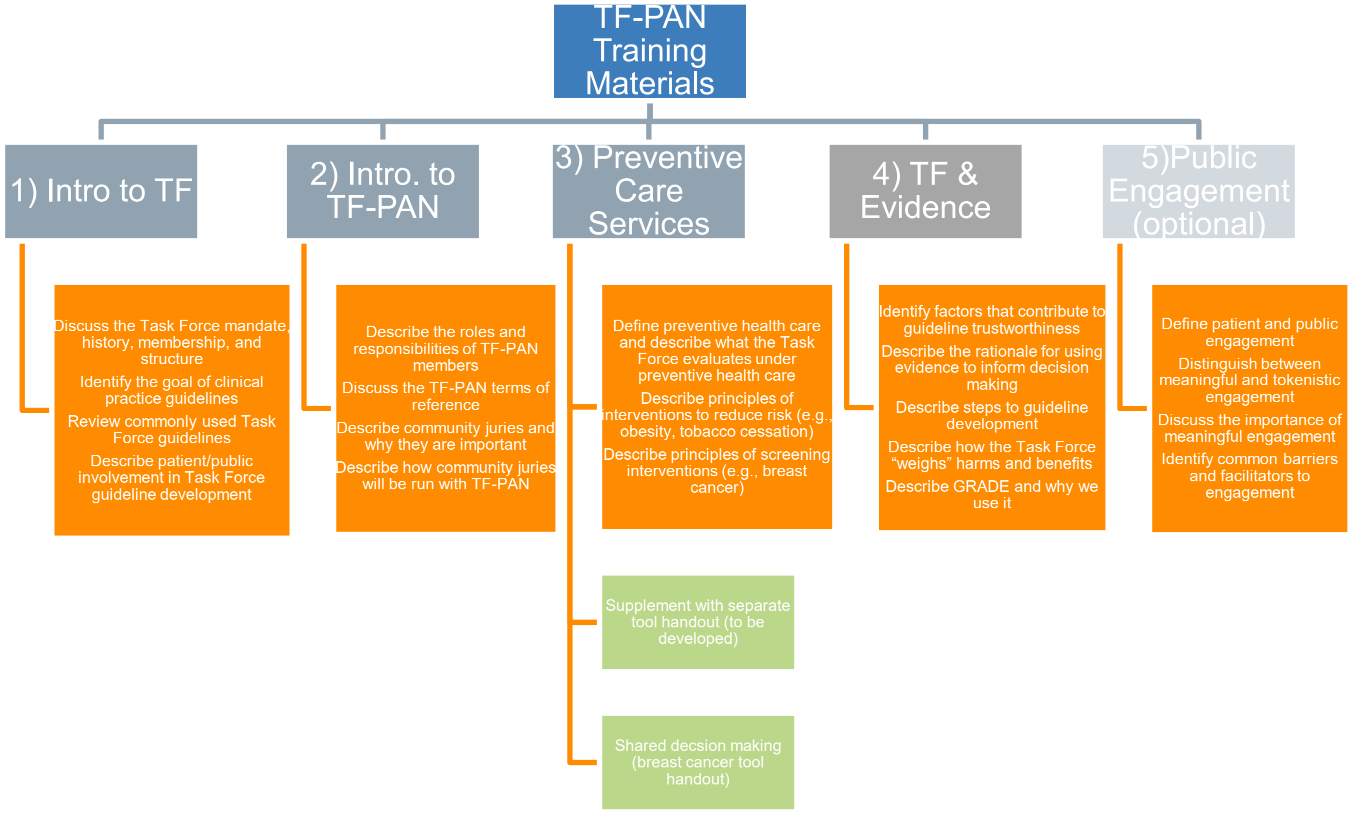

Appendix 11, as supplied by the authors. Appendix to: Thériault G, Limburg H, Klarenbach S, et al. Recommendations on screening for primary prevention of fragility fractures.
CMAJ 2023. doi: 10.1503/cmaj.221219. Copyright © 2023 The Author(s) or their employer(s).
To receive this resource in an accessible format, please contact us at cmajgroup@cmaj.ca.
