# Supplementary material for: Recommendations on screening for primary prevention of fragility fractures
Source: CMAJ. 2023 May 8;195(18):E639–49. doi: 10.1503/cmaj.221219 (PMC10166624; doi:10.1503/cmaj.221219)
Supplement: Appendix 2: Benefits of pharmacologic treatments [file 221219-guide-2-at.pdf]

Appendix 2: Benefits of pharmacologic treatments[1]

Outcome & Study approach	Studies; sample size; follow-up	Anticipated absolute effects (95% CI): Assumed population risk*	Anticipated absolute effects (95% CI): Risk with treatment	Anticipated absolute effects (95% CI): Absolute difference	Relative OR (95% CI)	Certainty	Judgement
**Bisphosphonates (alendronate, risedronate or zoledronic acid) vs placebo (postmenopausal females)**							
Hip fractures<br>Intention to treat	14 RCT; 21,038<br>Follow-up: 12-72 months<br>(1–17)	Control event rate (study data)			0.73 (0.58 to 0.92)	Low[a,c]	May reduce
		11 in 1000	8.1 per 1,000 (6.4 to 10.1)	2.9 fewer in 1000<br>(4.6 fewer to 0.9 fewer)			
		General population risk* (females)					
		20 in 1000	14.7 per 1,000 (11.7 to 18.4)	5.3 fewer in 1000<br>(8.3 fewer to 1.6 fewer)			
Clinical fragility fractures<br>Intention to treat or ≥1 dose	19 RCT; 22,482<br>Follow-up: 12-72 months<br>(1,3,12,13,15–22,4,23–25,5–11)	Control event rate (study data)			0.80 (0.73 to 0.88)	Moderate[a,c]	Probably reduces
		58 in 1000	46.9 per 1,000 (43.0 to 51.4)	11.1 fewer in 1000<br>(15.0 fewer to 6.6 fewer)			
		General population risk* (females)					
		202 in 1000	168.4 per 1,000 (156.0 to 182.2)	33.6 fewer in 1000<br>(46.0 fewer to 19.8 fewer)			
Clinical vertebral fractures; Intention to treat or ≥1 dose	11 RCT; 8,921<br>Follow-up: 12-72 months<br>(1,2,20,22,25,5–9,11,14,16)	Control event rate (study data)			0.52 (0.33, 0.81)	Low[a,b,d]	May reduce
		21 in 1000	11.0 per 1000 (7.0 to 17.1)	10.0 fewer in 1000<br>(14.0 fewer to 3.9 fewer)			
		General population risk* (females)					
		27 in 1000	14.2 per 1000 (9.1 to 22.0)	12.8 fewer in 1000<br>(17.9 fewer to 5.0 fewer)			
All-cause mortality<br>Intention to treat or exposed to ≥1 dose	8 RCT; 8,542<br>Follow-up: 12-72 months<br>(2,5,6,8,14,16,21,24,26,27)	Control event rate (study data)			0.81 (0.59 to 1.12)	Low[a-c]	May not reduce
		30 in 1000	24.0 per 1,000 (17.6 to 32.9)	5.5 fewer in 1000<br>(11.9 fewer to 3.4 more)			
		General population risk* (females)					
		57 in 1000	46.7per 1,000 (34.4 to 63.4)	10.3 fewer in 1000<br>(22.6 fewer to 6.4 more)			
**Alendronate vs placebo (post-menopausal females)**							
Hip fractures<br>Intention to treat	7 RCT; 9,226<br>Follow-up: 12-48 months<br>(1,2,11–16)	Control event rate (study data)			0.73 (0.43, 1.24)	Low[b,c,d]	May not reduce
		8 in 1000	5.9 per 1000 (3.5 to 9.9)	2.1 fewer in 1000<br>(4.5 fewer to 1.9 more)			
		General population risk* (females)					
		20 in 1000	5.9 per 1000 (3.5 to 9.9)	5.3 fewer in 1000<br>(11.3 fewer to 4.7 more)			
	8 RCT; 8,854	Control event rate (study data)				Moderate[b,c]	Probably reduces

[1] Vitamin D and calcium were usually given as part of treatment to both cases and controls

Appendix 2, as supplied by the authors. Appendix to: Thériault G, Limburg H, Klarenbach S, et al. Recommendations on screening for primary prevention of fragility fractures. *CMAJ* 2023. doi: 10.1503/cmaj.221219. Copyright © 2023 The Author(s) or their employer(s). To receive this resource in an accessible format, please contact us at cmajgroup@cmaj.ca.

Outcome & Study approach	Studies; sample size; follow-up	Anticipated absolute effects (95% CI)			Relative OR (95% CI)	Certainty	Judgement
		Assumed population risk*	Risk with treatment	Absolute difference			
Clinical fragility fractures<br>Intention to treat	Follow-up: 12-48 months<br>(1,10,21,11–13,15,16,18–20)	96 in 1000	79.3 per 1000 (69.5 to 91.4)	14.7 fewer in 1000 (24.5 fewer to 2.6 fewer)	0.83 (0.72, 0.97)		
		General population risk* (females)					
		202 in 1000	173.6 per 1000 (154.2 to 197.1)	28.4 fewer in 1000 (47.8 fewer to 4.9 fewer)			
Clinical vertebral fractures	5 RCTs; 6,324<br>Follow-up: 12-48 months<br>(1,2,11,14,16,20)	The evidence from 5 RCTs was very uncertain				Very low[a,b,c,d]	Very uncertain
All-cause mortality	4 RCTs; 5,272<br>Follow-up: 12-48 months<br>(2,14,16,21,26)	The evidence from 4 RCTs was very uncertain				Very low[a,b,c,d]	Very uncertain
**Risedronate vs placebo (post-menopausal females)**							
Hip fractures<br>Intention to treat	4 RCT; 9,672<br>Follow-up: 12-36 months<br>(3–5,17)	Control event rate (study data)			0.73 (0.56 to 0.95)	Low[b,c]	May reduce
		30 in 1000	22.1 per 1000 (17.0 to 28.5)	7.9 fewer in 1000 (13.0 fewer to 1.5 fewer)			
		General population risk* (females)					
		20 in 1000	14.7 per 1000 (11.3 to 19.0)	5.3 fewer in 1000 (8.7 fewer to 1.0 fewer)			
Clinical fragility fractures<br>Intention to treat or ≥1 dose	7 RCT; 10,572<br>Follow-up: 12-36 months<br>(3–5,17,19,22,23)	Control event rate (study data)			0.83 (0.73, 0.95)	Low[a-c]	May reduce
		48 in 1000	40.2 per 1000 (35.5 to 45.7)	7.8 fewer in 1000 (12.5 fewer to 2.3 fewer)			
		General population risk* (females)					
		202 in 1000	173.6 per 1000 (156.0 to 193.9)	28.4 fewer in 1000 (46.0 fewer to 8.1 fewer)			
Clinical vertebral fractures	2 RCT; 230<br>Follow-up: 12-36 months<br>(5,22)	The evidence from 2 RCTs is very uncertain				Very low	Very uncertain
All-cause mortality	1 RCT; 170<br>Follow-up: 12 months (5)	The evidence from 1 RCT is very uncertain				Very low	Very uncertain
**Zoledronic acid vs placebo (post-menopausal females)**							
Hip fractures<br>Intention to treat	3 RCT; 2,200<br>Follow-up: 12-72 months	Control event rate (study data)			0.69 (0.29 to 1.63)	Low[b,c]	May not reduce
		12 in 1000	8.3 per 1,000 (3.5 to 19.4)	3.7 fewer in 1000 (8.5 fewer to 7.4 more)			
		General population risk* (females)					

Appendix 2, as supplied by the authors. Appendix to: Thériault G, Limburg H, Klarenbach S, et al. Recommendations on screening for primary prevention of fragility fractures. *CMAJ* 2023. doi: 10.1503/cmaj.221219. Copyright © 2023 The Author(s) or their employer(s). To receive this resource in an accessible format, please contact us at cmajgroup@cmaj.ca.

Outcome & Study approach	Studies; sample size; follow-up	Anticipated absolute effects (95% CI)			Relative OR (95% CI)	Certainty	Judgement
		Assumed population risk*	Risk with treatment	Absolute difference			
	(6–9)	20 in 1000	13.9 per 1,000 (5.9 to 32.2)	6.1 fewer in 1000 (14.1 fewer to 12.2 more)			
Clinical fragility fractures Intention to treat	5 RCT; 3,218 Follow-up: 12-72 months (6–9,24,25)	Control event rate (study data)			0.64 (0.51 to 0.82)	Moderate[b]	Probably reduces
		50 in 1000	37.9 per 1,000 (30.4 to 48.1)	20.1 fewer in 1000 (27.6 fewer to 9.9 fewer)			
		General population risk* (females)					
		202 in 1000	139.4 per 1,000 (114.3 to 171.9)	62.6 fewer in 1000 (87.7 fewer to 30.1 fewer)			
Clinical vertebral fractures Intention to treat	4 RCT; 2,367 Follow-up: 12-72 months (6–9,25)	Control event rate (study data)			0.44 (0.24 to 0.8)	Low[a,b,d]	May reduce
		34 in 1000	15.3 per 1,000 (8.4 to 27.4)	18.7 fewer in 1000 (25.6 fewer to 6.6 fewer)			
		General population risk* (females)					
		27 in 1000	12.1 per 1,000 (6.6 to 21.7)	14.9 fewer in 1000 (20.4 fewer to 5.3 fewer)			
All-cause mortality	3 RCT; 2,656: Follow-up: 12-72 months (6,8,24,27)	The evidence from 3 RCTs is very uncertain				Very low[a,b,c,d]	Very uncertain
**Zoledronic acid vs placebo (males)**							
Hip fractures Intention to treat	1 RCT; 1,199 Follow-up: 24 months (28)	Control event rate (study data)			2.08 (0.19 to 22.98)	Low[b,c]	May not reduce
		2 in 1000	4.2 per 1,000 (0.4 to 44.0)	2.2 more in 1000 (1.6 fewer to 42.0 more)			
		General population risk* (males)					
		16 in 1000	32.7 per 1,000 (3.1 to 272.0)	16.7 more in 1000 (12.9 fewer to 256.0 more)			
Clinical fragility fractures Intention to treat	1 RCT; 1,199 Follow-up: 24 months (28)	Control event rate (study data)			0.57 (0.21 to 1.54)	Low[b,d]	May not reduce
		18 in 1000	10.3 per 1,000 (3.8 to 27.5)	7.7 fewer in 1000 (14.2 fewer to 9.5 more)			
		General population risk* (males)					
		105 in 1000	62.7 per 1,000 (24.0 to 153.0)	42.3 fewer in 1000 (81.0 fewer to 48.0 more)			
All-cause mortality	1 RCT; 1,199: Follow-up 24 months (28)	The evidence from 1 RCT is very uncertain				Very low[a,b,d]	Very uncertain
**Denosumab vs placebo (post-menopausal females)**							
Hip fractures	3 RCT; 8,542	Control event rate (study data)				Low[b-d]	May not reduce

Appendix 2, as supplied by the authors. Appendix to: Thériault G, Limburg H, Klarenbach S, et al. Recommendations on screening for primary prevention of fragility fractures. *CMAJ* 2023. doi: 10.1503/cmaj.221219. Copyright © 2023 The Author(s) or their employer(s). To receive this resource in an accessible format, please contact us at cmajgroup@cmaj.ca.

Outcome & Study approach	Studies; sample size; follow-up	Anticipated absolute effects (95% CI)			Relative OR (95% CI)	Certainty	Judgement
		Assumed population risk*	Risk with treatment	Absolute difference			
Intention to treat	Follow-up: 6-36 months (29–33)	11 in 1000	7.1 per 1,000 (4.3 to 11.2)	3.9 fewer in 1000 (6.7 fewer to 0.2 more)	0.64 (0.39 to 1.02)		
		General population risk* (females)					
		20 in 1000	12.9 per 1,000 (7.9 to 20.4)	7.1 fewer in 1000 (12.1 fewer to 0.4 more)			
Clinical fragility fractures Intention to treat or ≥1 dose	5 RCT; 9,231 Follow-up: 12-36 months (21,26,29–32,34)	Control event rate (study data)			0.70 (0.59 to 0.82)	Moderate[b]	Probably reduces
		42 in 1000	29.8 per 1,000 (25.2 to 34.7)	12.2 fewer in 1000 (16.8 fewer to 7.3 fewer)			
		General population risk* (females)					
		202 in 1000	150.5 per 1,000 (129.9 to 171.9)	51.5 fewer in 1000 (72.1 fewer to 30.1 fewer)			
Clinical vertebral fractures Intention to treat or ≥1 dose	3 RCT; 8,397 Follow-up: 6-36 months (29,32,34–36)	Control event rate (study data)			0.32 (0.21 to 0.49)	Moderate[b]	Probably reduces
		24 in 1000	7.8 per 1,000 (5.1 to 11.9)	16.2 fewer in 1000 (18.9 fewer to 12.1 fewer)			
		General population risk* (females)					
		27 in 1000	8.8 per 1,000 (5.8 to 13.4)	18.2 fewer in 1000 (21.2 fewer to 13.6 fewer)			
All-cause mortality Intention to treat or ≥1 dose	5 RCT; 9,185 Follow-up: 6-36 months (21,26,29–32,34,36)	Control event rate (study data)			0.79 (0.58 to 1.08)	Moderate[b,d]	Probably does not reduce
		23 in 1000	18.3 per 1,000 (13.5 to 24.8)	4.7 fewer in 1000 (9.5 fewer to 1.8 more)			
		General population risk* (females)					
		57 in 1000	45.6 per 1,000 (33.9 to 61.3)	11.4 fewer in 1000 (23.1 fewer to 4.3 more)			
Health-related quality of life (OPAQ-SV; 0-100; higher = better) after 3-y of treatment	5 RCT; 9,185 (21,26,29–32,34,36)	Change from baseline: physical function (-1.3 vs. -1.2), emotional status (-1.4 vs. -1.6), and back pain (4.1 vs. 4.3) for denosumab vs. placebo.				Moderate[b,c]	Probably does not change
**Denosumab vs placebo (men)**							
Hip fractures Intention to treat	1 RCT; 242 Follow-up: 12 months (37)	Control event rate (study data)			1.00 (0.02 to 50.80)	Very low[a,b,d]	Very uncertain
		0 in 1000	0.0 per 1,000 (0 to 0)	No difference in 1000			
		General population risk* (males)					
		16 in 1000	16.0 per 1,000 (0.3 to 452.4)	No difference in 1000 (15.7 fewer to 436.4 more)			
Clinical fragility fractures Intention to treat	1 RCT; 242 Follow-up: 12 months (37)	Control event rate (study data)			0.5 (0.04 to 5.59)	Very low[a,b,d]	Very uncertain
		16 in 1000	8.6 per 1,000 (0.7 to 88.2)	8.4 fewer in 1000 (16.3 fewer to 71.2 more)			
		General population risk* (males)					

Appendix 2, as supplied by the authors. Appendix to: Thériault G, Limburg H, Klarenbach S, et al. Recommendations on screening for primary prevention of fragility fractures. *CMAJ* 2023. doi: 10.1503/cmaj.221219. Copyright © 2023 The Author(s) or their employer(s). To receive this resource in an accessible format, please contact us at cmajgroup@cmaj.ca.

Outcome & Study approach	Studies; sample size; follow-up	Anticipated absolute effects (95% CI)			Relative OR (95% CI)	Certainty	Judgement
		Assumed population risk*	Risk with treatment	Absolute difference			
		105 in 1000	55.4 per 1,000 (4.7 to 396.1)	49.6 fewer in 1000 (100.3 fewer to 291.1 more)			
Clinical vertebral fractures Intention to treat	1 RCT; 242 Follow-up: 12 months (37)	Control event rate (study data)			1.00 (0.02 to 50.80)	Very low[a,b,d]	Very uncertain
		0 in 1000	0.0 per 1,000 (0 to 0)	No difference in 1000			
		General population risk* (males)					
		10 in 1000	9.8 fewer to 329.1 more	No difference in 1000			
All-cause mortality Exposed to ≥1 dose	1 RCT; 240 Follow-up: 12 months (37)	Control event rate (study data)			1.00 (0.06 to 16.17)	Very low[a,b,d]	Very uncertain
		8 in 1000	7.5 fewer to 107.4 more	No difference in 1000			
		General population risk* (males)					
		76 in 1000	(71.1 fewer to 494.8 more)	No difference in 1000			

CI=confidence interval; RCT=randomized controlled trial; NA=not applicable; OPAQ-SV=Osteoporosis Assessment Questionnaire-Short Version; OR = Odds ratio; y=years
a=risk of bias; b=inconsistency; c=indirectness; d=imprecision

* The effects without treatment (i.e., assumed population risk) for the general risk (Canadian) population (≥65 years) are estimated from Prior et al. based on 10 year follow-up (29)

Data for the general population <65 years is not included in the summary table.

References

1. Ascott-Evans BH, Guañabens N, Kivinen S, Stuckey BGA, Magaril CH, Vandormael K, et al. Alendronate Prevents Loss of Bone Density Associated With Discontinuation of Hormone Replacement Therapy. Arch Intern Med. 2003 Apr 14;163(7):789.
2. Chesnut CH, McClung MR, Ensrud KE, Bell NH, Genant HK, Harris ST, et al. Alendronate treatment of the postmenopausal osteoporotic woman: Effect of multiple dosages on bone mass and bone remodeling. Am J Med. 1995 Aug;99(2):144–52.
3. McClung MR, Geusens P, Miller PD, Zippel H, Bensen WG, Roux C, et al. Effect of Risedronate on the Risk of Hip Fracture in Elderly Women. N Engl J Med. 2001 Feb;344(5):333–40.
4. Mortensen L, Charles P, Bekker PJ, Digennaro J, Johnston CC. Risedronate Increases Bone Mass in an Early Postmenopausal Population: Two Years of Treatment Plus One Year of Follow-Up1. J Clin Endocrinol Metab. 1998 Feb 1;83(2):396–402.
5. Valimaki M, Farreronsminguella J, Halse J, Kroger H, Maroni M, Mulder H, et al. Effects of risedronate 5 mg/d on bone mineral density and bone turnover markers in late-postmenopausal women with osteopenia: A multinational, 24-month, randomized, double-blind, placebo-controlled, parallel-group, phase III

 Copyright © 2023 The Author(s) or their employer(s). To receive this resource in an accessible format, please contact us at cmajgroup@cmaj.ca.

trial. Clin Ther. 2007 Sep;29(9):1937–49.
6. Grey A, Bolland M, Mihov B, Wong S, Horne A, Gamble G, et al. Duration of antiresorptive effects of low-dose zoledronate in osteopenic postmenopausal women: a randomized, placebo-controlled trial. J Bone Miner Res. 2014 Jan;29(1):166–72.
7. Grey A, Bolland MJ, Wattie D, Horne A, Gamble G, Reid IR. The Antiresorptive Effects of a Single Dose of Zoledronate Persist for Two Years: A Randomized, Placebo-Controlled Trial in Osteopenic Postmenopausal Women. J Clin Endocrinol Metab. 2009 Feb 1;94(2):538–44.
8. Reid IR, Horne AM, Mihov B, Stewart A, Garratt E, Wong S, et al. Fracture Prevention with Zoledronate in Older Women with Osteopenia. N Engl J Med. 2018 Nov;379(25):2407–16.
9. Grey A, Bolland M, Wong S, Horne A, Gamble G, Reid IR. Low-dose zoledronate in osteopenic postmenopausal women: a randomized controlled trial. J Clin Endocrinol Metab. 2012 Jan;97(1):286–92.
10. Donaldson MG, Palermo L, Ensrud KE, Hochberg MC, Schousboe JT, Cummings SR. Effect of alendronate for reducing fracture by FRAX score and femoral neck bone mineral density: The fracture intervention trial. J Bone Miner Res. 2012 Aug;27(8):1804–10.
11. Hosking D, Chilvers CE, Christiansen C, Ravn P, Wasnich R, Ross P, et al. Prevention of bone loss with alendronate in postmenopausal women under 60 years of age. Early Postmenopausal Intervention Cohort Study Group. N Engl J Med. 1998 Feb;338(8):485–92.
12. Liberman UA, Weiss SR, Broll J, Minne HW, Quan H, Bell NH, et al. Effect of oral alendronate on bone mineral density and the incidence of fractures in postmenopausal osteoporosis. The Alendronate Phase III Osteoporosis Treatment Study Group. N Engl J Med. 1995 Nov;333(22):1437–43.
13. Pols HAP, Felsenberg D, Hanley DA, Štepán J, Muñoz-Torres M, Wilkin TJ, et al. Multinational, Placebo-Controlled, Randomized Trial of the Effects of Alendronate on Bone Density and Fracture Risk in Postmenopausal Women with Low Bone Mass: Results of the FOSIT Study. Osteoporos Int. 1999 Apr;9(5):461–8.
14. Yan Y, Wang W, Zhu H, Li M, Liu J, Luo B, et al. The efficacy and tolerability of once-weekly alendronate 70 mg on bone mineral density and bone turnover markers in postmenopausal Chinese women with osteoporosis. J Bone Miner Metab. 2009 Jul 3;27(4):471–8.
15. Tucci JR, Tonino RP, Emkey RD, Peverly CA, Kher U, Santora AC 2nd. Effect of three years of oral alendronate treatment in postmenopausal women with osteoporosis. Am J Med. 1996 Nov;101(5):488–501.
16. Cummings SR, Black DM, Thompson DE, Applegate WB, Barrett-Connor E, Musliner TA, et al. Effect of alendronate on risk of fracture in women with low bone density but without vertebral fractures: results from the Fracture Intervention Trial. JAMA. 1998 Dec;280(24):2077–82.
17. Yuming L, Zhongzhi Z, Xiuling D, Lulu C. Efficacy and safety of risedronate sodium in treatment of postmenopausal osteoporosis. J Huazhong Univ Sci Technol [Medical Sci. 2005 Sep;25(5):527–9.
18. Bell NH, Bilezikian JP, Bone III HG, Kaur A, Maragoto A, Santora AC. Alendronate Increases Bone Mass and Reduces Bone Markers in Postmenopausal African-American Women. J Clin Endocrinol Metab. 2002 Jun 1;87(6):2792–7.
19. Hosking D, Adami S, Felsenberg D, Andia JC, Välimäki M, Benhamou L, et al. Comparison of change in bone resorption and bone mineral density with once-weekly alendronate and daily risedronate: a randomised, placebo-controlled study. Curr Med Res Opin. 2003 Jan 22;19(5):383–94.
20. Hochberg MC, Thompson DE, Black DM, Quandt SA, Cauley J, Geusens P, et al. Effect of alendronate on the age-specific incidence of symptomatic osteoporotic fractures. J Bone Miner Res. 2005 Jun;20(6):971–6.
21. McClung MR, Lewiecki EM, Cohen SB, Bolognese MA, Woodson GC, Moffett AH, et al. Denosumab in Postmenopausal Women with Low Bone Mineral Density. N Engl J Med. 2006 Feb 23;354(8):821–31.
22. Fogelman I, Ribot C, Smith R, Ethgen D, Sod E, Reginster for the bmd-mn Study Grou J-Y. Risedronate Reverses Bone Loss in Postmenopausal Women with Low Bone Mass: Results From a Multinational, Double-Blind, Placebo-Controlled Trial [1]. J Clin Endocrinol Metab. 2000 May;85(5):1895–900.
23. Hooper MJ, Ebeling PR, Roberts AP, Graham JJ, Nicholson GC, D'Emden M, et al. Risedronate prevents bone loss in early postmenopausal women: a prospective randomized, placebo-controlled trial. Climacteric. 2005 Sep;8(3):251–62.
24. McClung M, Miller P, Recknor C, Mesenbrink P, Bucci-Rechtweg C, Benhamou C-L. Zoledronic Acid for the Prevention of Bone Loss in Postmenopausal Women With Low Bone Mass. Obstet Gynecol. 2009 Nov;114(5):999–1007.
25. Reid IR, Brown JP, Burckhardt P, Horowitz Z, Richardson P, Trechsel U, et al. Intravenous Zoledronic Acid in Postmenopausal Women with Low Bone Mineral Density. N Engl J Med. 2002 Feb 28;346(9):653–61.
26. Lewiecki EM, Miller PD, McClung MR, Cohen SB, Bolognese MA, Liu Y, et al. Two-Year Treatment With Denosumab (AMG 162) in a Randomized Phase 2 Study of Postmenopausal Women With Low BMD. J Bone Miner Res. 2007 Aug 16;22(12):1832–41.
27. Grey A, Bolland MJ, Horne A, Mihov B, Gamble G, Reid IR. Duration of antiresorptive activity of zoledronate in postmenopausal women with osteopenia: a

 Copyright © 2023 The Author(s) or their employer(s). To receive this resource in an accessible format, please contact us at cmajgroup@cmaj.ca.

randomized, controlled multidose trial. Can Med Assoc J. 2017 Sep 11;189(36):E1130–6.
28. Boonen S, Reginster J-Y, Kaufman J-M, Lippuner K, Zanchetta J, Langdahl B, et al. Fracture Risk and Zoledronic Acid Therapy in Men with Osteoporosis. N Engl J Med. 2012 Nov;367(18):1714–23.
29. Cummings SR, Martin JS, McClung MR, Siris ES, Eastell R, Reid IR, et al. Denosumab for prevention of fractures in postmenopausal women with osteoporosis. N Engl J Med. 2009 Aug;361(8):756–65.
30. Nct. Denosumab China Phase III Study [Internet]. Vol. 2021. 2016 [cited 2021 Mar 1]. Available from: https://www.cochranelibrary.com/central/doi/10.1002/central/CN-01479832/full and https://clinicaltrialsgov/show/NCT02014467
31. Zhu HM, Tang T, Cheng Q, He L, Li PQ, Xue QY, et al. Efficacy and Safety of Denosumab in Chinese Postmenopausal Women with Osteoporosis at Increased Risk of Fracture: Results From a 12-Month, Randomized, Double-blind, Placebo-controlled Phase III Study. Vol. 31, J Bone Miner Res. 2016. p. S160.
32. Pitale S, Thomas M, Rathi G, Deshmukh V, Kumar P, Reddy S, et al. A randomized placebo-controlled trial of the efficacy of denosumab in Indian postmenopausal women with osteoporosis. Indian J Endocrinol Metab. 2015 Jan;19(1):148–54.
33. Boonen S, Adachi JD, Man Z, Cummings SR, Lippuner K, Torring O, et al. Treatment with denosumab reduces the incidence of new vertebral and hip fractures in postmenopausal women at high risk. J Clin Endocrinol Metab. 2011 Jun;96(6):1727–36.
34. Bone HG, Bolognese MA, Yuen CK, Kendler DL, Wang H, Liu Y, et al. Effects of denosumab on bone mineral density and bone turnover in postmenopausal women. J Clin Endocrinol Metab. 2008 Jun;93(6):2149–57.
35. McCloskey E V, Johansson H, Oden A, Austin M, Siris E, Wang A, et al. Denosumab reduces the risk of osteoporotic fractures in postmenopausal women, particularly in those with moderate to high fracture risk as assessed with FRAX. J Bone Miner Res. 2012 Jul;27(7):1480–6.
36. McClung MR, Boonen S, Torring O, Roux C, Rizzoli R, Bone HG, et al. Effect of denosumab treatment on the risk of fractures in subgroups of women with postmenopausal osteoporosis. J Bone Miner Res. 2012 Jan;27(1):211–8.
37. Orwoll E, Teglbjaerg CS, Langdahl BL, Chapurlat R, Czerwinski E, Kendler DL, et al. A randomized, placebo-controlled study of the effects of denosumab for the treatment of men with low bone mineral density. J Clin Endocrinol Metab. 2012 Sep;97(9):3161–9.

Appendix 2, as supplied by the authors. Appendix to: Thériault G, Limburg H, Klarenbach S, et al. Recommendations on screening for primary prevention of fragility fractures. *CMAJ* 2023. doi: 10.1503/cmaj.221219. Copyright © 2023 The Author(s) or their employer(s). To receive this resource in an accessible format, please contact us at cmajgroup@cmaj.ca.
