# Supplementary material for: Recommendations on screening for primary prevention of fragility fractures
Source: CMAJ. 2023 May 8;195(18):E639–49. doi: 10.1503/cmaj.221219 (PMC10166624; doi:10.1503/cmaj.221219)
Supplement: Appendix 3: Calculations for overdiagnosis [file 221219-guide-3-at.pdf]

# Appendix 3: Calculations for overdiagnosis

**Definition:**
In the setting of screening to identify risk, we defined overdiagnosis as the identification of high risk in individuals who, if not screened, would never have known that they were at risk and would never have experienced a fracture. This calculation describes the extent of overdiagnosis in the screened population (compared to no screening).

**Calculation:**
**W =** proportion (%) of individuals deemed at high risk (based on threshold) or shared decision making
**y =** mean % risk in this high risk population
**100 – y =** % who would theoretically not fracture

**Extent of overdiagnosis = W x (100-y) / 100**

**Overdiagnosis using trial data:**

| Trials | SCOOP (Shepstone, 2018)<br>Females 70-80 years<br>10-year risk of hip fracture | | SALT (Merlijn, 2019)[a]<br>Females 65-90 years<br>10-year risk of MOF |
|---|---|---|---|
| | Offer-to-screen in "select population" | Screened as high-risk with clinical FRAX and referred for BMD | Offer-to screen in "select population" |
| Number offered screening | 6233 | 3064 | 5575 |
| Number above the treatment threshold | 898 | 898 | 1417 |
| % above the treatment threshold (W) | 14.4% | 29.3% | 25.4% |
| Mean risk in high risk group (y)[b] | 17.9% | 17.9% | 23.9% |
| Calculation of overdiagnosis | 14.4 x (100-17.9) / 100 | 29.3 x (100-17.9) / 100 | 25.4 x (100-23.9) / 100 |
| % overdiagnosed | 11.8% | 24.1% | 19.3% |

**MOF = major osteoporotic fracture**
**[a] This study included only women with at least one risk factor, so the proportion above the treatment threshold would be expected to be higher than the general population**
**[b] Calculated using clinical FRAX (without BMD); note that the trials did not use clinical FRAX for treatment thresholds**

Appendix 3, as supplied by the authors. Appendix to: Thériault G, Limburg H, Klarenbach S, et al. Recommendations on screening for primary prevention of fragility fractures. *CMAJ* 2023. doi: 10.1503/cmaj.221219. Copyright © 2023 The Author(s) or their employer(s). To receive this resource in an accessible format, please contact us at cmajgroup@cmaj.ca.
