# Supplementary material for: Recommendations on screening for primary prevention of fragility fractures
Source: CMAJ. 2023 May 8;195(18):E639–49. doi: 10.1503/cmaj.221219 (PMC10166624; doi:10.1503/cmaj.221219)
Supplement: Appendix 4: Harms (pharmacological) of screening to prevent fragility fractures [file 221219-guide-4-at.pdf]

Appendix 4: Harms (pharmacological)[1] of screening to prevent fragility fractures

Outcome; (Systematic review)	Intervention;	Studies; sample size	Assumed pop. risk[*]	Absolute effects[†] (95% CI)	RR unless otherwise stated (95% CI)	Certainty
**Serious Adverse Events**						
**Atypical femoral fracture;** (1–4)	Alendronate	1 cohort; 220,360	0.06 per 1000	0.08 more per 1000 (0.05 more to 0.14 more)	HR 2.41 (1.78 to 3.27)	Low[a,b,e]
		1 cohort; 220,360	0.03 per 1000	0.06 more per 1000 (0.03 more to 0.10 more)	HR 2.90 (1.97 to 4.26)	Low[a,b,e]
	Bisphosphonates (as a class)	1 cohort; ~2.8 million	0.3 in 1000	11 (7 to 14) more in 10,000	OR 126 (55 to 288)	Low[a,e]
		1 case-control; 1,368		NA	OR 93 (66 to 132) for >5 years of use	
		1 case-control; 290		NA	OR 25.65 (10.74 to 61.28)	
		3 RCT; NR		1.0 more in 1000 (2.6 fewer to 41.1 more)	1.33 (0.14 to 14.7)	Low[b]
		1 SR of 11 observational; NR		0.2 more in 1000 (0.1 more to 0.4 more)	1.70 (1.22 to 2.37)	
		Pooled: safety databases; NR		1.1 more in 1000 (0.7 more to 1.5 more)	4.51 (3.44 to 5.92)	
**Osteonecrosis of the jaw;** (1–4)	Alendronate	1 cohort; 220,360	0.1 per 1000	0.22 more per 1000 (0.04 more to 0.59 more)	HR 3.15 (1.44 to 6.87)	Low[a,b,e]
	Bisphosphonates (as a class)	Case series, SRs; NR	NR	Inconsistent, 0.3 to 43.0 more in 1000	NA	Low[b]
**Non-serious Adverse Events**						
**Any non-serious adverse event;** (5)	Zoledronic acid	6 RCT; 9,575	915 in 1000	51.8 more in 1000 (no difference to 112.2 more)	1.06 (1.00 to 1.13)	Moderate[a]
**Arthritis and arthralgia** (2–4)	Zoledronic acid	6 RCT; 11,171	145 in 1000	178.5 more in 1000 (137.4 more to 224.1 more)	OR 2.82 (2.32 to 3.45)[‡]	Moderate[a]
**Arthralgia, myalgia, pyrexia, chills, & influenza-like symptoms** (2–4)		6 RCT; 11,676	219 in 1000	422.8 more in 1000 (398.6 more to 446.3 more)	OR 6.39 (5.76 to 7.09)[‡]	Low[a,c]
**Chills** (5)		2 RCT; 799	12 in 1000	33.7 more in 1000 (3.0 more to 127.2 more)	3.81 (1.25 to 11.6)	Low[a,b,d]
**Eczema** (6,7)	Denosumab	1 RCT; 7,762	17 in 1000	13.8 more in 1000 (5.8 more to 24.5 more)	1.81 (1.34 to 2.44)	Low[a,b]

[1] Vitamin D and calcium were usually given as part of treatment to both cases and controls

Appendix 4, as supplied by the authors. Appendix to: Thériault G, Limburg H, Klarenbach S, et al. Recommendations on screening for primary prevention of fragility fractures. *CMAJ* 2023. doi: 10.1503/cmaj.221219. Copyright © 2023 The Author(s) or their employer(s). To receive this resource in an accessible format, please contact us at cmajgroup@cmaj.ca.

**Headache** (5)	Zoledronic acid	4 RCT; 9,712	53 in 1000	60.4 more in 1000 (19.1 more to 126.7 more)	2.14 (1.36 to 3.39)	Moderate[a]
**Infections** (2–4)	Denosumab	4 RCT; 8,691	7 in 1000	1.8 more in 1000 (0.1 more to 4.0 more)	1.26 (1.01 to 1.57)	Moderate[a]
**Influenza-like symptoms** (2–4)	Zoledronic acid	5 RCT; 10,695	44 in 1000	142.5 more in 1000 (105.5 more to 188.4 more)	OR 4.98 (3.82 to 6.58)‡	Moderate[a]
**Myalgia** (2–4)		5 RCT; 11,065	17 in 1000	70.7 more in 1000 (54.6 more to 90.8 more)	OR 5.56 (4.46 to 6.99)‡	Moderate[a]
**Non-serious gastrointestinal adverse events** (2–4)	Alendronate	50 RCT; 22,549	589 per 1000	16.3 more per 1000 (2.4 more to 31.3 more)	OR 1.07 (1.01 to 1.14)‡	Moderate[a]
	Denosumab	3 RCT; 8,454	105 in 1000	64.5 more in 1000 (26.4 more to 113.3 more)	OR 1.74 (1.29 to 2.38)‡	Moderate[a]
**Pyrexia** (5)	Zoledronic acid	5 RCT; 11,823	38 in 1000	127.7 more in 1000 (34.6 more to 337.4 more)	4.36 (1.91 to 9.88)	Moderate[a]
**Rash or eczema** (6,7)	Denosumab	3 RCT; 8,454	17 in 1000	15.8 more in 1000 (7.6 more to 27.0 more)	OR 1.96 (1.46 to 2.66)‡	Moderate[a]

CI=confidence interval;; HR=Hazard ratio; NA=Not applicable; NR=Not reported; OR=Odds ratio; RCT=Randomized controlled trial; SR=systematic review;

*The assumed population risk (without treatment) is the median rate in the control group for studies in the analysis. These were extracted directly from the systematic reviews when possible. Data were extracted from the included primary studies when there were ≤5 in the analysis or from the 5 largest studies from larger analyses to calculate the control event rate.

a=risk of bias; b=inconsistency; c=indirectness; d=imprecision; e=Large magnitude of effect (+1)

†The absolute effect (and its 95% CI) is based on applying the relative effect of the intervention (and its 95% CI) to the assumed risk. When an OR is presented, the following formula was used: intervention risk per 1000 = 1000 x (OR x ACR / 1 – ACR + (OR x ACR)) (8) ACR=Assumed control risk.

‡Odds ratio derived from exact logistic regression meta-analysis (109).

References

1. Fink HA, MacDonald R, Forte ML, Rosebush CE, Ensrud KE, Schousboe JT, et al. Long-Term Drug Therapy and Drug Discontinuations and Holidays for Osteoporosis Fracture Prevention: A Systematic Review. Ann Intern Med. 2019 Nov;171(1):37–50.
2. Qaseem A, Forciea MA, McLean RM, Denberg TD. Treatment of low bone density or osteoporosis to prevent fractures in men and women: A clinical practice guideline update from the American college of physicians. Ann Intern Med. 2017 Jun;166(11):818–39.
3. Crandall CJ, Newberry SJ, Diamant A, Lim Y-W, Gellad WF, Booth MJ, et al. Comparative effectiveness of pharmacologic treatments to prevent fractures: an updated systematic review. Ann Intern Med. 2014 Nov;161(10):711–23.
4. Crandall CJ, Newberry SJ, Gellad WG, Diamant A, Lim YW, Suttorp M, et al. Treatment to Prevent Fractures in Men and Women with Low Bone Density or Osteoporosis: Update of a 2007 Report. Comparative Effectiveness Review No. 53. (Prepared by Southern California Evidence-based Practice Center under Contract No. HHSA-290-2007-10062-I.). Rockville, MD: Agency for Healthcare Research and Quality; 2012.
5. Davis S, Martyn-St James M, Sanderson J, Stevens J, Goka E, Rawdin A, et al. A systematic review and economic evaluation of bisphosphonates for the prevention of fragility fractures. Health Technol Assess (Rockv). 2016 Oct;20(78):1–406.
6. Viswanathan M, Reddy S, Berkman N, Cullen K, Middleton JC, Nicholson WK, et al. Screening to Prevent Osteoporotic Fractures: An Evidence Review for

Copyright © 2023 The Author(s) or their employer(s). To receive this resource in an accessible format, please contact us at cmajgroup@cmaj.ca.

the U.S. Preventive Services Task Force. Evidence Synthesis No.162. Rockville, MD: Agency for Healthcare Research and Quality; 2018.

7. Viswanathan M, Reddy S, Berkman N, Cullen K, Middleton JC, Nicholson WK, et al. Screening to Prevent Osteoporotic Fractures. JAMA. 2018 Jun 26;319(24):2532.

8. Higgins J, Thomas J, Chandler J, Cumpston M, Li T, Page M, et al. Cochrane Handbook for Systematic Reviews of Interventions version 6.2 [Internet]. 2021 [cited 2021 Nov 19]. Available from: www.training.cochrane.org/handbook
