# Supplementary material for: Recommendations on screening for primary prevention of fragility fractures
Source: CMAJ. 2023 May 8;195(18):E639–49. doi: 10.1503/cmaj.221219 (PMC10166624; doi:10.1503/cmaj.221219)
Supplement: Appendix 5: Accuracy of risk asesment tools (calibrated for Canada) [file 221219-guide-5-at.pdf]

Appendix 5: Accuracy of risk assessment tools (calibrated for Canada)

Outcome[*]<br>Studies; sample size	Findings	Certainty of calibration[†]	Discrimination[‡] (pooled AUC, 95% CI)
**Clinical FRAX (lower risk of bias studies)**			
**10-year hip fractures**<br>3 cohort; 67,611<br>(1–3)	All studies used the FRAX tool calibrated for Canada. The pooled O:E showed acceptable calibration with some underestimation of the observed fracture risk, and a wide confidence interval (pooled O:E 1.13, 95% CI 0.74-1.72, I^2=89.2%).	Low[b,d]	All studies, regardless of risk of bias:<br>Females: 0.76 (0.72-0.81)<br>Males: 0.73 (0.68-0.77)
**10-year clinical fragility fractures**<br>3 cohort; 67,611<br>(1–3)	All studies used the FRAX tool calibrated for Canada. The pooled O:E showed acceptable calibration with some underestimation of the observed fracture risk (O:E 1.10, 95% CI 1.01-1.20, I^2=50.4%).	Moderate[b,d]	All studies, regardless of risk of bias:<br>F: 0.67 (0.65-0.68)<br>M: 0.62 (0.61-0.64)
**5-y hip fractures**<br>1 cohort; 68,730 (62,275 females, 6,445 males)<br>(3)	A single study, which used the FRAX tool calibrated for Canada, showed large overestimation of the observed 5-year risk of hip fracture in females (O:E 0.68, 95% CI 0.62-0.73) and imprecise overestimation in males (O:E 0.82, 95% CI 0.60- 1.03).	Low[a,b,d]	Not reported
**5-year clinical fragility fractures[8]**<br>1 cohort; 68,730 (62,275 females, 6,445 males)<br>(3)	A single study, which used the FRAX tool calibrated for Canada, found acceptable calibration in females (O:E 0.93, 95% CI 0.89-0.96). The tool imprecisely underestimated the observed fracture risk in males (O:E 1.23, 95% CI 1.08-1.38).	Low[a,b,d]	Not reported
**FRAX + BMD (lower risk of bias studies)**			
**10-year hip fractures**<br>3 cohort; 61,156 (1–3)	All studies used the FRAX tool calibrated for Canada. The pooled O:E showed underestimation of the observed risk with a high level of inconsistency (O:E 1.31, 95% CI 0.91-2.13, I^2 = 92.7%); two comparisons showed acceptable calibration while two others showed substantial underestimation of the observed fracture risk.	Low[b,d]	All studies, regardless of risk of bias:<br>Females: 0.79 (0.76-0.81)<br>Males: 0.76 (0.72-0.80)
**10-year clinical fragility fractures**<br>3 cohort; 61,156 (1–3)	All studies used the FRAX tool calibrated for Canada. The pooled O:E showed acceptable calibration with some underestimation of the observed risk (O:E 1.16, 95% CI 1.12-1.20, I^2 = 0%).	Moderate[b,d]	All studies, regardless of risk of bias:<br>Females: 0.70 (0.68-0.71)<br>Males: 0.67 (0.66-0.68)
**5-year hip fractures[8]**<br>1 cohort; 68,730 (62,275 females, 6,445 males)<br>(3)	A single study, which used the FRAX tool calibrated for Canada, showed acceptable calibration with some overestimation in females (O:E 0.88, 95% CI 0.81-0.95) and males (O:E 0.88, 95% CI 0.65-1.10).	Low[,b,d]	Not reported
**5-year clinical fragility fractures[8]**<br>1 cohort; 68,730 (62,275 females, 6,445 males)<br>(3)	A single, which used the FRAX tool calibrated for Canada, study provided inconsistent findings, showing acceptable calibration in females (O:E 1.00, 95% CI 0.97-1.04). The tool imprecisely underestimated the observed fracture risk in males (O:E 1.22, 95% CI 1.07, 1.37).	Low[a,b,d]	Not reported
**CAROC (includes BMD)**			
**10-year clinical fragility fractures**<br>1 cohort; 34,060 (4)	Study did not report an O:E ratio. Observed fracture risk (95% CI) was 6.4 (6.0-6.8)% in the low risk (<10%) group, 13.8 (13.1-14.5)% in the moderate risk group (10-20%), and 23.8 (22.5-25.0)% in the high risk group (>20%).	Low[a-c]	Not reported

AUC=Area under the curve; BMD=bone mineral density; CI=confidence interval; F=female; M=male; O:E ratio=ratio of observed to expected (predicted) events

Appendix 5, as supplied by the authors. Appendix to: Thériault G, Limburg H, Klarenbach S, et al. Recommendations on screening for primary prevention of fragility fractures. *CMAJ* 2023. doi: 10.1503/cmaj.221219. Copyright © 2023 The Author(s) or their employer(s). To receive this resource in an accessible format, please contact us at cmajgroup@cmaj.ca.

a=risk of bias; b=inconsistency; c=indirectness; d=imprecision
[*]Rows for 5-year fractures have been omitted from the table when no studies were located that reported on this outcome.
[†] When our assessment of the certainty of evidence fell between levels, we assigned the level that best represented our actual certainty.
[‡]Extracted directly from Viswanathan et al., 2018 (5).

References

1. Crandall CJ, Schousboe JT, Morin SN, Lix LM, Leslie W. Performance of FRAX and FRAX-Based Treatment Thresholds in Women Aged 40 Years and Older: The Manitoba BMD Registry. J Bone Miner Res. 2019 Aug 17;34(8):1419–27.
2. Fraser L-A, Langsetmo L, Berger C, Ioannidis G, Goltzman D, Adachi JD, et al. Fracture prediction and calibration of a Canadian FRAX® tool: a population-based report from CaMos. Osteoporos Int. 2011 Mar 16;22(3):829–37.
3. Leslie WD, Majumdar SR, Morin SN, Lix LM, Johansson H, Oden A, et al. FRAX for fracture prediction shorter and longer than 10 years: the Manitoba BMD registry. Osteoporos Int. 2017 Sep 7;28(9):2557–64.
4. Leslie WD, Majumdar SR, Lix LM, Josse RG, Johansson H, Oden A, et al. Direct comparison of FRAX(R) and a simplified fracture risk assessment tool in routine clinical practice: a registry-based cohort study. Osteoporos Int. 2016 Nov;27(9):2689–95.
5. Viswanathan M, Reddy S, Berkman N, Cullen K, Middleton JC, Nicholson WK, et al. Screening to Prevent Osteoporotic Fractures: An Evidence Review for the U.S. Preventive Services Task Force. Evidence Synthesis No.162. Rockville, MD: Agency for Healthcare Research and Quality; 2018.

 Copyright © 2023 The Author(s) or their employer(s). To receive this resource in an accessible format, please contact us at cmajgroup@cmaj.ca.
