# Supplementary material for: Recommendations on screening for primary prevention of fragility fractures
Source: CMAJ. 2023 May 8;195(18):E639–49. doi: 10.1503/cmaj.221219 (PMC10166624; doi:10.1503/cmaj.221219)
Supplement: Appendix 6: Patient preferences for screening to prevent fragility factures: Data summary [file 221219-guide-6-at.pdf]

Canadian Task Force on Preventive Health Care

Patient preferences for screening to prevent fragility factures: Data summary

Prepared for the Canadian Task Force on Preventive Health Care

Submitted: November 5th, 2018

Prepared By:
Lynsey Burnett, Kathleen Einarson, Alekhya Mascarenhas, Rossella Scoleri, Sherry Stein, Sharon Straus, and Ainsley Moore

Knowledge Translation Program
Li Ka Shing Knowledge Institute
St. Michael's Hospital

Contact:
Lynsey Burnett

E: burnettly@smh.ca
T: 416-864-6060 ext. 77566

St. Michael's
Inspired Care. Inspiring Science.

Appendix 6, as supplied by the authors. Appendix to: Thériault G, Limburg H, Klarenbach S, et al. Recommendations on screening for primary prevention of fragility fractures. *CMAJ* 2023. doi: 10.1503/cmaj.221219.
Copyright © 2023 The Author(s) or their employer(s). To receive this resource in an accessible format, please contact us at cmajgroup@cmaj.ca.

Table of Contents

Introduction 2
Methods 2
Participants 2
Recruitment 2
Characteristics of included participants 3
Outcome ratings 3
Outcomes scale ratings 3
Overall preferences for screening 7
Participant perceptions of outcomes for screening 8
Participant requests for information 8
Values and preferences for screening 10
Factors influencing access to screening 16
Participant engagement ratings scale 17
Participant experience ratings scale 19
Participants' overall experience 20
Limitations 24
Suggestions for applying findings 24
Conclusion 26
References 28
Appendix A: Screening questionnaire 29
Appendix B: Background sheet 43
Appendix C: Pre- and post-focus group survey 45
Appendix D: Sample personalized response sheet 58
Appendix E: Focus group guide 69
Appendix F: Patient engagement survey 78

Appendix 6, as supplied by the authors. Appendix to: Thériault G, Limburg H, Klarenbach S, et al. Recommendations on screening for primary prevention of fragility fractures. *CMAJ* 2023. doi: 10.1503/cmaj.221219.

Copyright © 2023 The Author(s) or their employer(s). To receive this resource in an accessible format, please contact us at cmajgroup@cmaj.ca.

Introduction

The Canadian Task Force on Preventive Health Care (CTFPHC) recruits members of the public to provide input during the guideline development and knowledge translation (KT) tool development process at up to three critical phases. This document presents summary data from Phase 1 of the CTFPHC patient preferences assessment about screening to prevent fragility fractures. We obtained Phase 1 data via focus groups and surveys. We examined patients' perceptions of the outcomes of screening to determine if someone is at high risk for fragility fractures. Specifically, we asked how important patients believe it is to consider various outcomes (harms and benefits) of screening to determine if they are at high risk for fragility fractures when making screening decisions. We also examined participants' experiences in the project. We collected data between August 29th and October 5th, 2018.

Methods

For a detailed description of the methods used in this project, please refer to Phase 1 of the CTFPHC's Patient Engagement Protocol (http://canadiantaskforce.ca/methods/patient-preferences-protocol/).

Participants

Recruitment

Participants were English-speaking men and women in Canada who are either members of the screening population, or those who have experienced a fragility fracture and/or been previously or currently diagnosed with osteoporosis or osteopenia. We recruited participants by posting recruitment advertisements on public advertisement websites (i.e., Craigslist and Kijiji). We also circulated recruitment advertisements in the newsletters and internal communications of relevant organizations: Osteoporosis Canada, Bone and Joint Health in Alberta, Strategy for Patient Oriented Research Evidence Alliance, and the Canadian Frailty Network.

We asked individuals who responded to the recruitment announcement to complete a brief online screening questionnaire to assess their eligibility to take part in the project (see Appendix A). Participants were eligible to take part if they were aged 50 years and older.

Participants were not eligible for the project if they identified with one or more of the following statements:

- I am less than 50 years of age;
- I am a health care practitioner;
- I am aware of conflicts of interest relevant to the guideline topic (e.g., Owning, or owning shares in, a company that provides products or services related to osteoporosis);
- I am in long-term care or hospitalized;
- I have a disorder likely to be related to metabolic bone disease, such as premature ovarian failure, hypogonadism, prior cancer/chemotherapy, chronic kidney disease (requiring dialysis), untreated hyperthyroidism, hyperparathyroidism, adrenal insufficiency, or Cushing's syndrome;

Appendix 6, as supplied by the authors. Appendix to: Thériault G, Limburg H, Klarenbach S, et al.
Recommendations on screening for primary prevention of fragility fractures. *CMAJ* 2023. doi: 10.1503/cmaj.221219.
Copyright © 2023 The Author(s) or their employer(s). To receive this resource in an accessible format, please contact us at cmajgroup@cmaj.ca.

- I chronically use steroid medications (>5 mg/day **oral** prednisone [or equivalent] for ≥3 months).

Participants were compensated $50 for participating in the project as per the SMH KT Program internal reimbursement policy.

Characteristics of included participants

The final sample consisted of 4 males and 21 females who were 52 to 73 years of age (mean age = 62.8 years, standard deviation = 6.2). Two participants self-identified as Indigenous (i.e., First Nations, Métis, or Inuit). Participants were from Ontario (*n* = 14), British Columbia (*n* = 3), Saskatchewan (*n* = 3), Alberta (*n* = 2), Quebec (*n* = 1), Prince Edward Island (*n* = 1), and Manitoba (*n* = 1). The majority of participants lived in urban and suburban areas (*n* = 16; *n* = 6); three participants lived in a rural area and one participant chose not to answer this question. The majority of participants had a college diploma or bachelor's degree (*n* = 15) or a graduate or professional degree (*n* = 8). Participants had household incomes of less than $30,000 (*n* = 5), $30,000-$39,999 (*n* = 2), $40,000-$49,999 (*n* = 1), $50,000-$59,999 (*n* = 2), $60,000-$69,999 (*n* = 2) $70,000-$79,999 (*n* = 4), $80,000 - $89,999 (*n* = 1), $90,000 - $99,999 (*n* = 1), and $100,000 or more (*n* = 7).

Based on eligibility survey responses, we separated participants into two groups; *'exposed'* and *'unexposed'*. We defined *'exposed'* participants (*n*= 13) as those who have experienced a fragility fracture and /or have previously or currently been diagnosed with osteoporosis or osteopenia. Participants were considered '*unexposed' (n*=12*)* if they did not meet these criteria (i.e. were part of the screening population).

Outcome ratings

Below is a summary of participants' perceptions of the outcomes of screening to determine if someone is at high risk for fragility fractures. As explained in the Patient Engagement Protocol, these data were collected using a modified RAND Appropriateness Method (RAM)[1] using surveys and focus groups.

Outcomes scale ratings

In the first part of the survey, participants rated the importance of outcomes of screening to determine if someone is at high risk for fragility fractures. We provided all participants with information on each of these potential outcomes, also referred to as harms and benefits. For each outcome, we asked participants to rate how much the outcome would influence their decision on whether or not to be screened to prevent fragility fractures.

Participants rated the importance of the outcome from 1-9, where scores indicated:

- 1-3 - not important for decision making
- 4-6 - important for decision making
- 7-9 - critical for decision making

Appendix 6, as supplied by the authors. Appendix to: Thériault G, Limburg H, Klarenbach S, et al. Recommendations on screening for primary prevention of fragility fractures. CMAJ 2023. doi: 10.1503/cmaj.221219.
Copyright © 2023 The Author(s) or their employer(s). To receive this resource in an accessible format, please contact us at cmajgroup@cmaj.ca.

Table 1 provides the full description of the outcomes that participants were asked to rate. The short descriptions of outcomes are used in Figure 1 and Table 2.

Table 1. Descriptions for outcomes

Short description	Full description
**Benefits (*n* = 6)**	
Decreased hip bone fractures	Screening may decrease the number of hip bone fractures that someone will experience
Decreased fragility fractures	Screening may decrease the number of broken bones caused by a minor fall or normal activity that usually should not cause a fracture in healthy adults.
Decreased chance of death **(direct)**	Screening may decrease the chance of death **directly** related to a bone fracture.
Decreased chance of death **(indirect)**	Screening may decrease the chance of death from causes or conditions that may be **indirectly** related to, or worsened by, a bone fracture.
Improved quality of life	Screening that leads to a decrease in fractures may improve overall well-being and quality of life.
Improved day-to-day functions	Screening that leads to a decrease in fractures may improve your ability to perform every-day activities. This can include decreased admissions to long-term care.
**Harms (*n* = 7)**	
Treatment – non-serious side effects	Screening may lead to treatment with medications used to lower the risk of fractures. These medications can have unwanted, but not serious, side effects. This could include nausea, heartburn, abdominal pain, loose bowels, rashes, muscle cramps, or non-serious infections.
Stopping treatment	Screening may result in treatment with medications used to lower the risk of fractures. In some cases treatment is stopped because of unwanted side effects of these medications
Treatment – serious side effects	Screening may lead to treatment with medications used to lower the risk of fractures. In very rare cases, medication can have serious side effects, including death or serious illness.
Over-diagnosis	Screening may correctly identify you as being at high risk of a fracture, even though you would never have actually experienced a fracture in your lifetime. This can lead to unnecessary tests, treatments, worry and concern (called over-diagnosis)
Overtreatment	Screening may result in treatment to prevent fragility fractures when there is little or no

Appendix 6, as supplied by the authors. Appendix to: Thériault G, Limburg H, Klarenbach S, et al. Recommendations on screening for primary prevention of fragility fractures. CMAJ 2023. doi: 10.1503/cmaj.221219.
Copyright © 2023 The Author(s) or their employer(s). To receive this resource in an accessible format, please contact us at cmajgroup@cmaj.ca.

	evidence that the benefits of treatment would outweigh the harms of treatment (called overtreatment).
False positive	Screening may result in incorrectly identifying someone as being high-risk for fractures, when they are actually not at high-risk for fractures (called a false positive result)
False negative	Screening may result in incorrectly identifying someone as being low-risk for fractures, when they are actually at a high-risk for fractures (called a false negative)

A summary of survey responses is presented below as well as in Figure 1 and Table 2. Figure 1 and the synopsis below are based on the post-focus group survey results. However, in Table 2 both pre- and post-focus group survey data are included for comparison purposes.

How to read the box plot

To show participant ratings, we used the box plot throughout this report. The box plot whiskers show the full range of responses, the box shows the interquartile range (IQR), and the line within the box indicates the median. For instance, looking at "ectopic pregnancy" in the sample figure below, the range is 3-9, the IQR is 5-9, and the median is 7. All possible responses are whole numbers; therefore, the median will sometimes be the same value as the first or third quartile. Similarly, a quartile may be the same value as the corresponding whisker. In those cases, a line next to the quartile indicates the median or whisker is the same number

Sample figure: Box plot

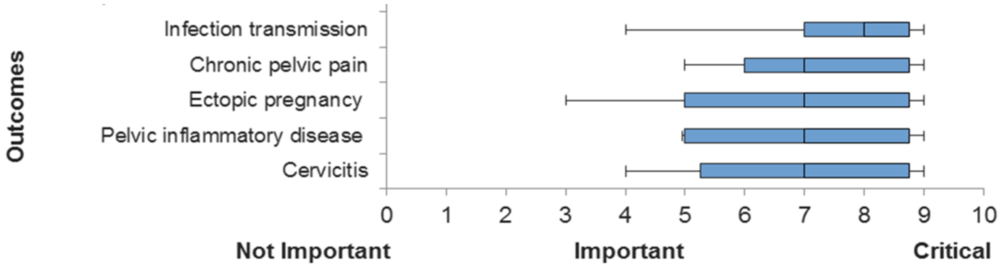

St. Michael's
Inspired Care.
Inspiring Science.

Appendix 6, as supplied by the authors. Appendix to: Thériault G, Limburg H, Klarenbach S, et al. Recommendations on screening for primary prevention of fragility fractures. CMAJ 2023. doi: 10.1503/cmaj.221219.

Copyright © 2023 The Author(s) or their employer(s). To receive this resource in an accessible format, please contact us at cmajgroup@cmaj.ca.

Figure 1: Post-survey harms and benefits scale ratings (n = 25)

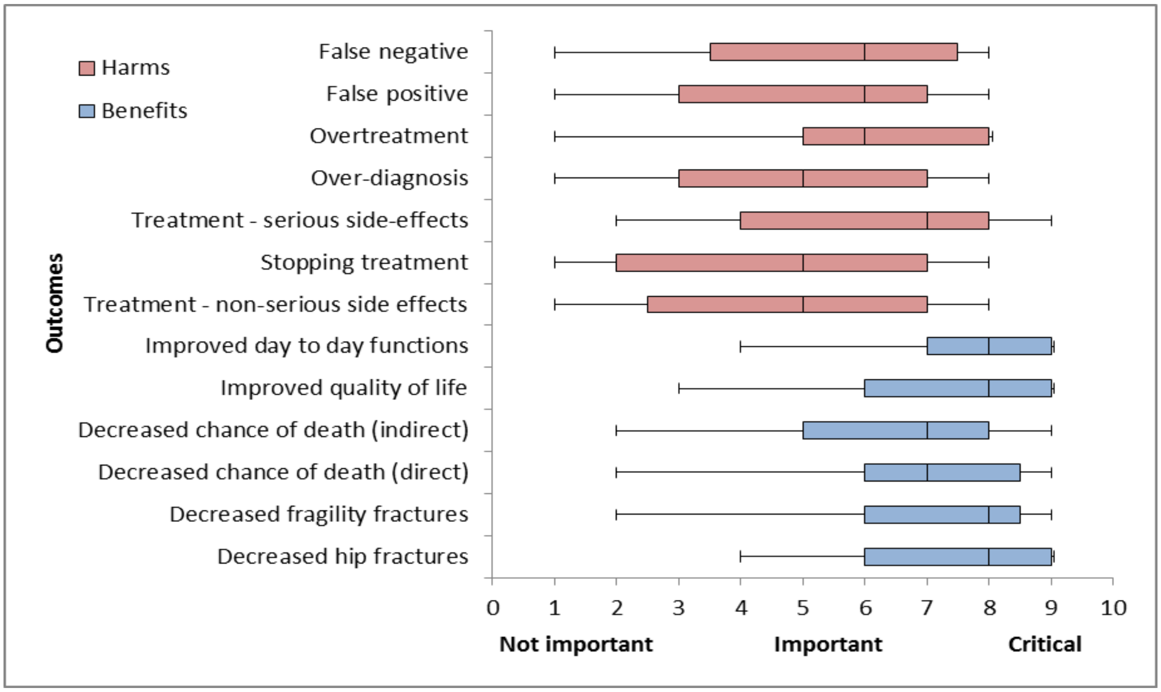

Table 2. Pre- and post-survey harms and benefits scale ratings (n= 29; n = 25)

Outcome	Pre-survey (n=29)			Post-survey (n=25)		
	Median	IQR*	Range	Median	IQR	Range
**Benefits**						
Decreased hip fractures	7	5.5-8	5-9	8	6-9	4-9
Decreased fragility fractures	7	6-8	3-9	8	6-8.5	2-9
Decreased chance of death (direct)	7	6-8.5	1-9	7	6-8.5	2-9
Decreased chance of death (indirect)	7	5-8	1-9	7	5-8	2-9
Improved quality of life	8	7-9	5-9	8	6-9	3-9
Improved day-to-day functions	8	7-9	5-9	8	7-9	4-9
**Harms**						
Treatment – non-serious side effects	5	3.5-7	1-8	5	2.5-7	1-8
Stopping treatment	5	3-7	1-9	5	2-7	1-8
Treatment – serious side effects**	7	4.5-8	2-9	-	-	-
Over-diagnosis	5	4-7	1-9	5	3-7	1-8
Overtreatment	6	4.5-8	1-9	6	5-8	1-8
False positive	5	3-7	1-9	6	3-7	1-8
False negative	6	5-7.5	1-8	6	3.5-7.5	1-8

**Note: IQR = interquartile range.*

*** Post-survey responses were not recorded for this outcome; Figure 1 shows pre-survey values for this outcome.*

Appendix 6, as supplied by the authors. Appendix to: Thériault G, Limburg H, Klarenbach S, et al. Recommendations on screening for primary prevention of fragility fractures. CMAJ 2023. doi: 10.1503/cmaj.221219.

Copyright © 2023 The Author(s) or their employer(s). To receive this resource in an accessible format, please contact us at cmajgroup@cmaj.ca.

Median post-survey outcome ratings for benefits ranged from 7 to 8. Median post-survey outcome ratings for harms ranged from 5 to 6. The rated importance of most outcomes presented remained generally the same between the pre and post surveys. Post-survey IQR of ratings for benefit outcomes indicated participants felt all benefits were *important* or *critical*. The post-survey IQR of ratings for harm outcomes were lower overall, ranging from *not important* to *critical.* This suggests participants generally placed less importance on harms compared with benefits when deciding whether to be screened for fragility fractures; however, the median rating was 5 or 6 for all harms, which indicates participants still felt all harms were *important* to consider.

We also asked participants to select up to five outcomes that they think were most critical to consider when making a decision about being screened to prevent fragility fractures. Participants generally included benefits more often than harms in their top five outcomes to consider when making a screening decision.

Overall preferences for screening

In the second part of the survey, participants rated their overall preference for screening to prevent fragility fractures. We asked participants to rate the statement “Considering the potential harms and benefits of screening to determine if someone is at high risk for fragility fractures, how much would you want to be screened?” on a scale from 1-9. A score of 1 indicated “Not at all”; a score of 5 indicated “Neutral”; and a score of 9 indicated “Very much”.

A summary of survey responses is presented below as well as in Table 3. Table 3 presents overall preferences for screening, and includes pre- and post- focus group survey data for comparison purposes.

Table 3. Pre- and post-survey overall screening preferences (n =29; n = 25)

Outcome	Pre-survey (*n*=29)			Post-survey (*n*=25)		
	Median	IQR*	Range	Median	IQR	Range
Overall screening preference	8	5.5-9	4-9	8	6.5-9	4-9

**Note: IQR = interquartile range.*

Participants showed a wide range of preferences for screening. However, the median post-survey preference for screening was 8, indicating most participants had a strong preference for screening to determine if they are at high risk for fragility fractures. The median preference rating in the *unexposed* group was 7, and median preference rating in the *exposed* group was 9. This suggests that those who have previously been diagnosed with a fragility fracture and/or been diagnosed with osteoporosis or osteopenia may have a stronger preference for screening than those who have not; however, a larger sample size would be needed to determine if the difference in preference rating between the two groups is statistically significant.

Appendix 6, as supplied by the authors. Appendix to: Thériault G, Limburg H, Klarenbach S, et al.
Recommendations on screening for primary prevention of fragility fractures. CMAJ 2023. doi: 10.1503/cmaj.221219.
Copyright © 2023 The Author(s) or their employer(s). To receive this resource in an accessible format, please contact us at cmajgroup@cmaj.ca.

We further explored participant preferences for screening to determine if someone is at high risk for fragility fractures in the focus groups. The results of the focus group discussions are presented below.

Participant perceptions of outcomes for screening

We used four focus groups (n = 25) to gather qualitative data from participants about the importance of the outcomes of screening to determine if someone is at high risk for fragility fractures when deciding whether or not be screened, and their overall preferences for screening. We conducted two focus groups with exposed participants (n = 13) and two focus groups with unexposed participants (n= 12). We coded focus group transcripts using a directed content analysis approach[2].

A summary of focus group discussions and survey responses are presented in Tables 5 and 6.

Participant requests for information

Table 5. Information requested by participants (n=25)

Needs	Description	Illustrative quotes
**Background information sheet**	**EXPOSED:**<br>It was noted that the term 'fragility fracture' is not gender neutral.<br><br>Participants found the background sheet to be well organized. In particular:<br><br>i) sections were an appropriate length<br><br>ii) paragraphs were brief and to the point<br><br>iii) language level was appropriate	*"Fragile has some connotations that would be off-putting to men who have to be screened for the problem."* FG4<br><br>*"I like how you explained terms, not left to assumption. It was clear and concise."* FG3
	**UNEXPOSED:**<br>In general, participants found the background sheet clear and easy to understand. In particular:<br><br>i) it was well organized<br><br>ii) Q and A format was helpful<br><br>It was also noted it was not a 'light' read.	*"I really had to pay attention. Not something you can browse through."* FG1
**Additional information to be added to background sheet**	**EXPOSED:**<br>Participants made a number of suggestions regarding information they felt should be included in the background sheet:<br><br>i) an explanation of the progression from osteopenia to osteoporosis	

Appendix 6, as supplied by the authors. Appendix to: Thériault G, Limburg H, Klarenbach S, et al. Recommendations on screening for primary prevention of fragility fractures. CMAJ 2023. doi: 10.1503/cmaj.221219.
Copyright © 2023 The Author(s) or their employer(s). To receive this resource in an accessible format, please contact us at cmajgroup@cmaj.ca.

	ii) factors that determine the need for screening (e.g. age, heritable factors, ethnicity)<br><br>iii) available screening methods<br><br>iv) screening vs. diagnostic testing<br><br>v) additional information on medications<br><br>vi) treatment options other than medications (e.g. lifestyle)<br><br>vii) age at which screening would be most beneficial in order to access prevention options	*"Screening should provide information on prevention, so more things to do to prevent getting fractures."* FG3
	**UNEXPOSED:**<br><br>Suggestions for additional information include:<br><br>i) various methods of screening available, including screening modalities offered outside of Canada<br><br>ii) osteoporosis risk factors<br><br>iii) ability to self-diagnose (e.g. does bone density of one's teeth indicate risk of osteoporosis?)<br><br>iv) additional information on medication, including types of medication, risks, frequency and route of administration<br><br>v) identification of target populations and frequency of screening	*"The one question I would like to see added is if one can self-diagnose the risk. Does frame, overweight, etc. factors increase risk? So, listing factors that can increase one's risk for fragility fractures."* FG1<br><br>*"It does not give an indication of the percentage of population who would be affected or severity of patients. Should this be targeted or widespread screening?"* FG1

This table summarizes the additional background information participants requested, as well as information and topics participants considered important to discuss with their primary care providers in order to make an informed screening decision.

Participants requested more information on treatment options (i.e. different types of medications or lifestyle changes) and their associated risks and effectiveness, the types of screening tests used, the proposed frequency of screening, as well as what the specific risk factors are for osteoporosis. Participants also wondered what the screening test or tool would look like (e.g. what types of questions would be asked in a fragility risk questionnaire). In particular, participants requested further clarification on the difference between screening and diagnostic testing (i.e. who would be eligible for screening). Participants felt a conversation with their health care provider about any preventative measures that could be taken to improve outcomes of those who are considered high-risk would also be helpful as part of a shared decision-making process.

Appendix 6, as supplied by the authors. Appendix to: Thériault G, Limburg H, Klarenbach S, et al.
Recommendations on screening for primary prevention of fragility fractures. CMAJ 2023. doi: 10.1503/cmaj.221219.
Copyright © 2023 The Author(s) or their employer(s). To receive this resource in an accessible format, please contact us at cmajgroup@cmaj.ca.

Values and preferences for screening

The qualitative data collected through focus groups (*n* = 25) revealed the outcomes of screening that may influence a patient's overall preference for being screened to prevent fragility fractures. Table 6 summarizes all unique values and preferences present in the qualitative data.

Table 6. Participants' values and preferences for screening (n = 19)

Factors	Description		Illustrative quotes
**Perceived benefits of screening**	1. Screening may decrease the chance of death directly related to a bone fracture.	**EXPOSED:**<br><br>Participants felt they were faced with a decision on the value of screening in a worst case scenario only.<br><br>In addition, participants felt it was an unrealistic leap from screening to decreasing death.	*"Does it have to be the worst possible outcome before you value the screening?"* FG3
		**UNEXPOSED:**<br><br>This was generally deemed a long-term benefit and not considered important.<br><br>Participants felt that statistics for this potential benefit would be important to consider.	*"I rated it low because I thought the chances would be low."* FG1
	2. Screening may decrease the chance of death from causes or conditions that may be indirectly related to, or worsened by, a bone fracture.	**EXPOSED:**<br><br>Participants noted that this benefit became increasingly important with age.	*"This is important if it would decrease death or other conditions because of bone fracture. This would push me to get screened."* FG1
		**UNEXPOSED:**<br><br>Participants felt this to be an important factor in deciding whether to undergo screening.<br><br>Hip fracture was frequently cited as a concern by participants. They appeared	*"[Hip fracture] stats show that the number of such deaths are very high. If screening could decrease this, it would benefit the senior age group*

Appendix 6, as supplied by the authors. Appendix to: Thériault G, Limburg H, Klarenbach S, et al. Recommendations on screening for primary prevention of fragility fractures. CMAJ 2023. doi: 10.1503/cmaj.221219.
Copyright © 2023 The Author(s) or their employer(s). To receive this resource in an accessible format, please contact us at cmajgroup@cmaj.ca.

		particularly aware of the seriousness of this complication.	*and high risk groups."* FG2
	3. Screening that leads to a decrease in fractures may improve overall wellbeing and quality of life.	**EXPOSED:**<br><br>Participants considered this to be a relevant factor in deciding whether or not to be screened.	*"You care more about this because you know what's at risk and you know how you want to live your life when you've only got 20, 30, 40 years left in a normal cycle."* FG4
		**UNEXPOSED:**<br><br>Participants expressed mixed attitudes regarding this potential benefit.<br><br>While some rated it as an important factor, others felt screening based on this possible benefit was subjective.	*"I rated it high because I was unfortunately a bone fracture patient this year. I saw how decreased my quality of life was. I would do everything to avoid this from happening again."* FG1<br><br>*"It should be an optional choice in my opinion. Some would say as a woman I should have bone density scans but my active lifestyle, etc. is a good indicator that I am not at high risk."* FG2

Appendix 6, as supplied by the authors. Appendix to: Thériault G, Limburg H, Klarenbach S, et al. Recommendations on screening for primary prevention of fragility fractures. CMAJ 2023. doi: 10.1503/cmaj.221219.
Copyright © 2023 The Author(s) or their employer(s). To receive this resource in an accessible format, please contact us at cmajgroup@cmaj.ca.

**Perceived harms of screening**	1. Potential medication side effects and possible cessation of treatment, serious illness or death	**EXPOSED:**<br><br>Potential side effects and the possibility of ceasing treatment was not a deterrent to most participants. The potential benefits of treatment were considered more important than this possible harm.<br><br>The possibility of death, however, elicited a far more concerned response that would deter individuals from seeking treatment.<br><br>Screening and treatment were considered separate components with respect to determining potential harms.<br><br>A number of participants did express concern about medication side effects. In particular, they cited the possibility that both starting and stopping medication could have harmful effects.	*"Knowing side effects is important. They have to disclose all worst case scenarios for side effects and it's helpful for me."* FG3<br><br>*"Results or risks of taking meds is not that important. I want to know my risk level."* FG3
		**UNEXPOSED:**<br><br>This was generally considered by participants to be a significant harm and would negatively factor into the decision to screen.<br><br>Information regarding incidence rates of harms would also impact the decision.<br><br>Despite this general consensus, there were participants who expressed awareness that side effects vary by individual and may be short term. In addition, some were reassured by the knowledge that severe side effects were rare.	*"Reference to treatment being stopped influenced my answer because the side effects must have been bad so had to stop treatment."* FG1<br><br>*"I don't know if I would participate in screening if medication would be as risky as if I had the fragility facture in the first place."* FG2<br><br>*"It would not stop me from getting screened but when it comes to treatment I would choose medication very carefully."* FG2

St. Michael's
Inspired Care.
Inspiring Science.

Appendix 6, as supplied by the authors. Appendix to: Thériault G, Limburg H, Klarenbach S, et al. Recommendations on screening for primary prevention of fragility fractures. CMAJ 2023. doi: 10.1503/cmaj.221219.
Copyright © 2023 The Author(s) or their employer(s). To receive this resource in an accessible format, please contact us at cmajgroup@cmaj.ca.

	2. Over diagnosis (i.e. identification as high risk with no lifetime fracture occurring)	**EXPOSED:**<br><br>The possibility of over diagnosis was considered significant and could deter some individuals from seeking screening or treatment.<br><br>However, the importance of weighing potential harms of over diagnosis against the benefit of treatment was noted to be important to the process.<br><br>Over diagnosis is considered an important factor in the decision as to whether or not to accept treatment. Having a relationship with a physician who understands your concerns was considered important.	*"It [over diagnosis] puts a lot of onus on any one patient in the system to share their doubts."* FG4
		**UNEXPOSED:** n/a	n/a
	3. False positive	**EXPOSED:**<br><br>Participants would take into account the incidence rate of false positives when determining their willingness to screen and take next steps.<br><br>Generally, a false positive was considered less concerning than a false negative	*"I would rather have a false positive and change lifestyle. Doing that would be better than having a false negative. I would rather worry on the side of what can I do to help the situation."* FG3

Appendix 6, as supplied by the authors. Appendix to: Thériault G, Limburg H, Klarenbach S, et al. Recommendations on screening for primary prevention of fragility fractures. CMAJ 2023. doi: 10.1503/cmaj.221219.
Copyright © 2023 The Author(s) or their employer(s). To receive this resource in an accessible format, please contact us at cmajgroup@cmaj.ca.

		**UNEXPOSED:**<br><br>Participants wanted additional information before they could identify how this possible harm would affect their decision to screen:<br><br>i) percentage of tests that are false positive<br><br>ii) whether secondary screening methods are used to confirm the result<br><br>In general, a false positive was considered less significant than a false negative. However, concern regarding unnecessary psychological effects (e.g. anxiety) was expressed.	*"In the big picture, having bone fragility is not the worst news you can get in the medical system."* FG2
	4. False negative	**EXPOSED:**<br><br>Participants would generally not be deterred by the possibility of a false negative but would take this into account when determining next steps (i.e. subsequent screening).	*"Being able to verify the accuracy of screening would be important."* FG1
		**UNEXPOSED:**<br><br>Participants felt a false negative was a more significant outcome than a false positive.	
**Preferences for screening:**	**EXPOSED:**<br><br>Participants offered mixed preferences around screening.<br><br>For those who held the opinion that there were more benefits than harms of screening, the following responses were noted:<br><br>i) bone scans are relatively low risk<br><br>ii) harms and benefits of screening and treatment should be differentiated from each other<br><br>iii) screening has a positive impact on seniors' health		*"Making a decision on screening is one thing and then making a decision on treatment is another. I see that there are different risks for each situation. I would want to be screened to see if high risk but then the decision on treatment would be different."* FG3

Appendix 6, as supplied by the authors. Appendix to: Thériault G, Limburg H, Klarenbach S, et al.
Recommendations on screening for primary prevention of fragility fractures. CMAJ 2023. doi: 10.1503/cmaj.221219.
Copyright © 2023 The Author(s) or their employer(s). To receive this resource in an accessible format, please contact us at cmajgroup@cmaj.ca.

	iv) screening provides informed options<br><br>v) awareness of risk factors would motivate screening<br><br>For those who would not screen or were indecisive, the following responses were noted:<br><br>i) if screening were less invasive it would be acceptable<br><br>ii) the decision would depend on the method of screening and its accuracy<br><br>iii) more evidence and education would be required before a decision to screen could be made<br><br>iv) clarification is needed as to why screening would be required if an individual is considered to be at risk<br><br>v) the possible negative impact of medication on daily activities of life would decrease the likelihood of screening	*"If I had been screened early, I might have been able to prevent the fractures I have now."* FG4<br><br>*"I would be screened. I believe you get a range so do it multiple times to see how badly you are progressing."* FG3
	**UNEXPOSED:**<br><br>As with the exposed group, participants expressed mixed attitudes toward screening.<br><br>While some participants would only be screened if they were at high risk, others would choose to be screened regardless of risk factors.<br><br>In addition, personal knowledge of someone who has experienced a hip fracture encouraged positive attitudes towards screening.	*"I wouldn't be screened. If I can't change the risk factors, I don't want to worry about something I can't control."* FG1

This table summarizes participants' overall preferences for taking part in screening to prevent fragility fractures, as well as participant discussions about specific screening outcomes.

Participants in both the exposed and unexposed focus groups held mixed feelings and attitudes towards screening. Some felt that the type of screening tests offered, including their effectiveness and invasiveness, would influence their screening decision. More invasive testing generally reduced the likelihood participants would participate in screening. Some participants' willingness to screen increased if measures were possible to reduce their risk. The likelihood of harms from medication would greatly impact some participants' willingness to be screened (i.e. if harms from treatment were likely, they would be less inclined to participate in screening). However, some participants emphasized that they felt treatment outcomes and screening outcomes should be listed and considered separately, because they viewed treatment and screening as separate decisions.

It should be noted that throughout focus group discussions, it was evident that further clarification on the difference between screening and diagnostic testing is required (i.e. some participants indicated a preference for screening *if* they were at high risk, however this particular screening is to *determine if* someone is at high risk for fragility fractures). This confusion, coupled with some participants' confusion surrounding how to interpret questions that combined screening and treatment outcomes, may have led some participants to interpret survey questions differently.

Appendix 6, as supplied by the authors. Appendix to: Thériault G, Limburg H, Klarenbach S, et al. Recommendations on screening for primary prevention of fragility fractures. CMAJ 2023. doi: 10.1503/cmaj.221219.
Copyright © 2023 The Author(s) or their employer(s). To receive this resource in an accessible format, please contact us at cmajgroup@cmaj.ca.

Factors influencing access to screening

Focus group (*n* = 25) responses revealed several barriers and facilitators to accessing screening to determine if someone is at high risk for fragility fractures. A summary is provided in Table 7.

Table 7. Factors that influence participants' access to screening (n = 25)

Factors	Description	Illustrative quotes
**Potential <u>barriers</u> to screening**	**EXPOSED:**<br><br>Barriers to screening were identified as:<br><br>i) cost<br><br>ii) fear of screening<br><br>iii) failure of family physician to provide a guideline for screening	*"I don't want paying for this screening to be an issue especially if I am fairly confident that I would get a fragility fracture."* FG3
	**UNEXPOSED:**<br><br>Barriers to screening were identified as:<br><br>i) travel distance and time required to access screening<br><br>ii) expense involved in travelling to screening<br><br>iii) language/literacy issues related to screening	
**Potential <u>facilitators</u> to screening**	**EXPOSED:**<br><br>Accessibility to a healthcare provider was identified as a facilitator to screening.	
	**UNEXPOSED:**<br><br>Facilitators to screening were identified as:<br><br>i) access to screening as part of annual medical tests<br><br>ii) access to questionnaire at home (with phone assistance available)<br><br>iii) relationship with family physician	*"It's important that the doctor knows the patient well and their lifestyle. This would help the doctor know if they have to recommend screening or not."* FG2

In summary, participants described several perceived barriers and facilitators to accessing screening to determine if someone is at high risk for fragility fractures.

Appendix 6, as supplied by the authors. Appendix to: Thériault G, Limburg H, Klarenbach S, et al. Recommendations on screening for primary prevention of fragility fractures. CMAJ 2023. doi: 10.1503/cmaj.221219.
Copyright © 2023 The Author(s) or their employer(s). To receive this resource in an accessible format, please contact us at cmajgroup@cmaj.ca.

Some participants identified language and literacy issues as barriers to screening, specifically if a questionnaire is used as a screening tool. Participants also felt costs associated with travelling to medical services and screening, including time required and potential missed work, could be a potential barrier. Some participants noted the importance of having an established, trusted relationship between a patient and health care provider when discussing screening, so that the patient's unique values and preferences were understood and considered. Participants felt improving accessibility, such as being able to complete a screening questionnaire at home or including screening as part of an annual check-up, could facilitate screening.

In the post-focus group survey, we asked participants to provide feedback on their experience in the project. The focus group and survey questions are available in Appendix E: Focus group guide and Appendix F: Participant engagement and experience items. For the full data collection method, see the Patient Engagement Protocol. Responses are summarized below.

Participant engagement ratings scale

In the post-focus group survey, we asked participants a series of questions about their experience in the project.[3] Participants responded using a 7-item scale, with the following response options: No extent (1), Very small extent (2), Small extent (3), Fair extent (4), Moderate extent (5), Large extent (6), or Very large extent (7). We also asked participants to explain their ratings for each engagement item. The quantitative responses to these questions are summarized in Figure 3 and Table 8. The quantitative ratings and relevant qualitative explanations are also summarized below.

The majority of experience questions had a median response of 6, indicating an overall positive engagement experience. Similar to trends seen in several previous data summary reports, the participants gave lower ratings for questions asking if participants felt their input would influence final guideline decisions and if they believed their values and preferences would be included in the final advice of the CTFPHC. Participants also rated the question asking if participants were able to clearly express viewpoints slightly lower, with a median of 5. Based on the open-ended response questions, lower ratings for this question were generally associated with participants from larger focus groups (*n* >10).

Appendix 6, as supplied by the authors. Appendix to: Thériault G, Limburg H, Klarenbach S, et al.
Recommendations on screening for primary prevention of fragility fractures. CMAJ 2023. doi: 10.1503/cmaj.221219.
Copyright © 2023 The Author(s) or their employer(s). To receive this resource in an accessible format, please contact us at cmajgroup@cmaj.ca.

Figure 3. Survey responses for participant engagement items (n = 25)

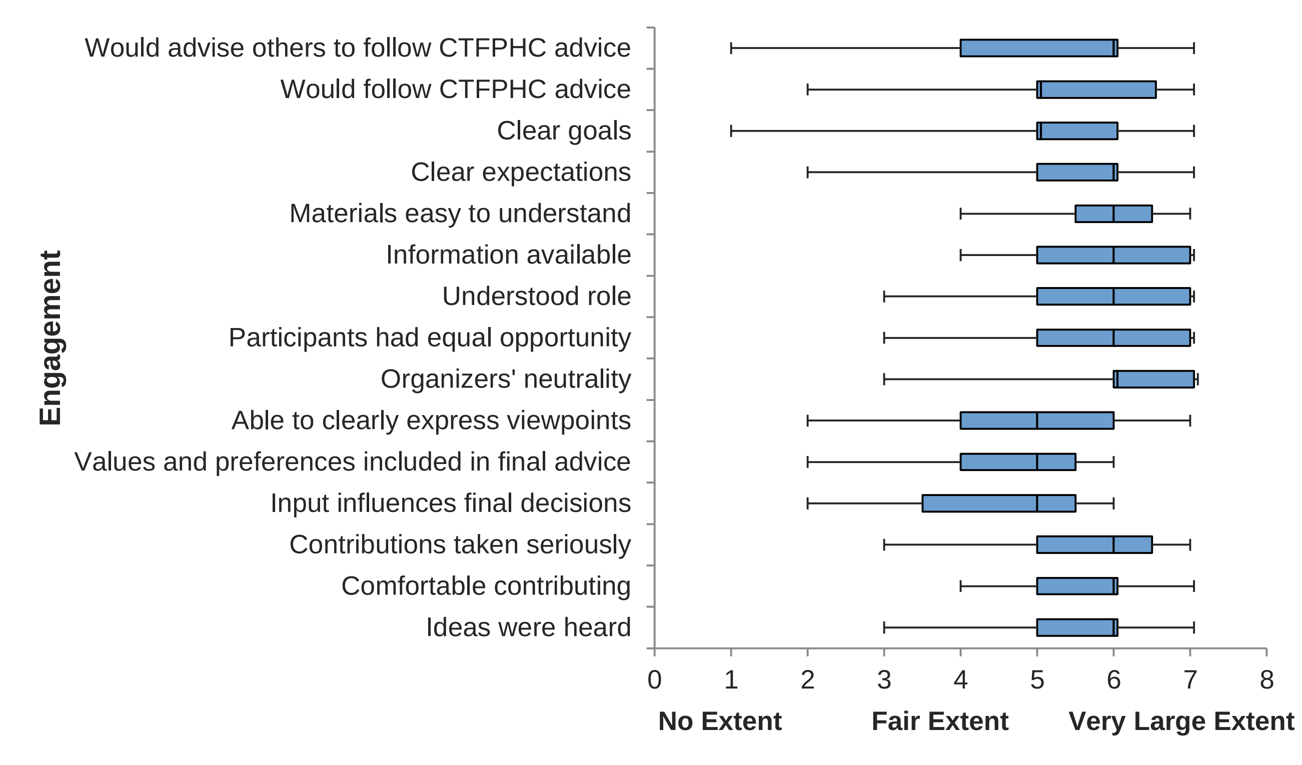

Appendix 6, as supplied by the authors. Appendix to: Thériault G, Limburg H, Klarenbach S, et al. Recommendations on screening for primary prevention of fragility fractures. CMAJ 2023. doi: 10.1503/cmaj.221219.
Copyright © 2023 The Author(s) or their employer(s). To receive this resource in an accessible format, please contact us at cmajgroup@cmaj.ca.

Table 8. Survey responses for participant engagement items (n = 25)

Question	Median	IQR*	Range
To what extent do you believe that your ideas were heard during the engagement process?	6	5-6	3-7
To what extent did you feel comfortable contributing your ideas to the engagement process?	6	5-6	4-7
Did organizers take your contributions to the engagement process seriously?	6	5-6.5	3-7
To what extent do you believe that your input will influence final decisions that underlie the engagement process?	5	3.5-5.5	2-6
To what extent do you believe that your values and preferences will be included in the final health advice from this process?	5	4-5.5	2-6
To what extent were you able to clearly express your viewpoints?	5	4-6	2-7
How neutral in their opinions (regarding topics) were organizers during the engagement process?	6	6-7	3-7
Did all participants have equal opportunity to participate in discussions?	6	5-7	3-7
How clearly did you understand your role in the process?	6	5-7	3-7
To what extent was information made available to you either prior or during the engagement process so as to participate knowledgeably in the process?	6	5-7	4-7
To what extent were the ideas contained in the information material easy to understand?	6	5.5-6.5	4-7
How clearly did you understand what was expected of you during the engagement process?	6	5-6	2-7
How clearly did you understand what the goals of the engagement process were?	5	5-6	1-7
To what extent would you follow health advice from the Canadian Task Force on Preventive Health Care (if it related to your health condition)?	5	5-6.5	2-7
To what extent would you advise others to follow health advice from the Canadian Task Force on Preventive Health Care (if it related to their health condition)?	6	4-6	1-7

**Note: IQR = interquartile range*

Participant experience ratings scale

After participants responded to questions about their engagement, they responded to questions about the clarity and ease of the tasks that they were requested to complete. We asked participants to rate questions using a 9-point scale: a score of 1 indicated “Not at all”; a score of 5 indicated “Neutral”; and a score of 9 indicated “Very much”. A summary of the responses is presented in Figure 4 and Table 9.

Overall, participants responded positively to all five questions, with a median rating of 7 or 8 for all engagement questions. This indicates most participants completed the survey tasks with clarity and ease. However it should be noted that the wide range of responses for these questions, coupled with some participants’ comments in focus group discussions and open-ended survey questions, indicate some participants found survey questions and tasks unclear. We also asked participants to summarize what they had been asked to do in the survey. The majority of participants accurately described the survey tasks they completed; seven participants chose not to answer the open-ended question. Thus,

Appendix 6, as supplied by the authors. Appendix to: Thériault G, Limburg H, Klarenbach S, et al.
Recommendations on screening for primary prevention of fragility fractures. CMAJ 2023. doi: 10.1503/cmaj.221219.
Copyright © 2023 The Author(s) or their employer(s). To receive this resource in an accessible format, please contact us at cmajgroup@cmaj.ca.

while there is converging evidence that most participants understood the survey tasks, some participants were unclear about the wording of some survey questions and the end goal of the study.

Figure 4. Survey responses for experience items (n = 25)

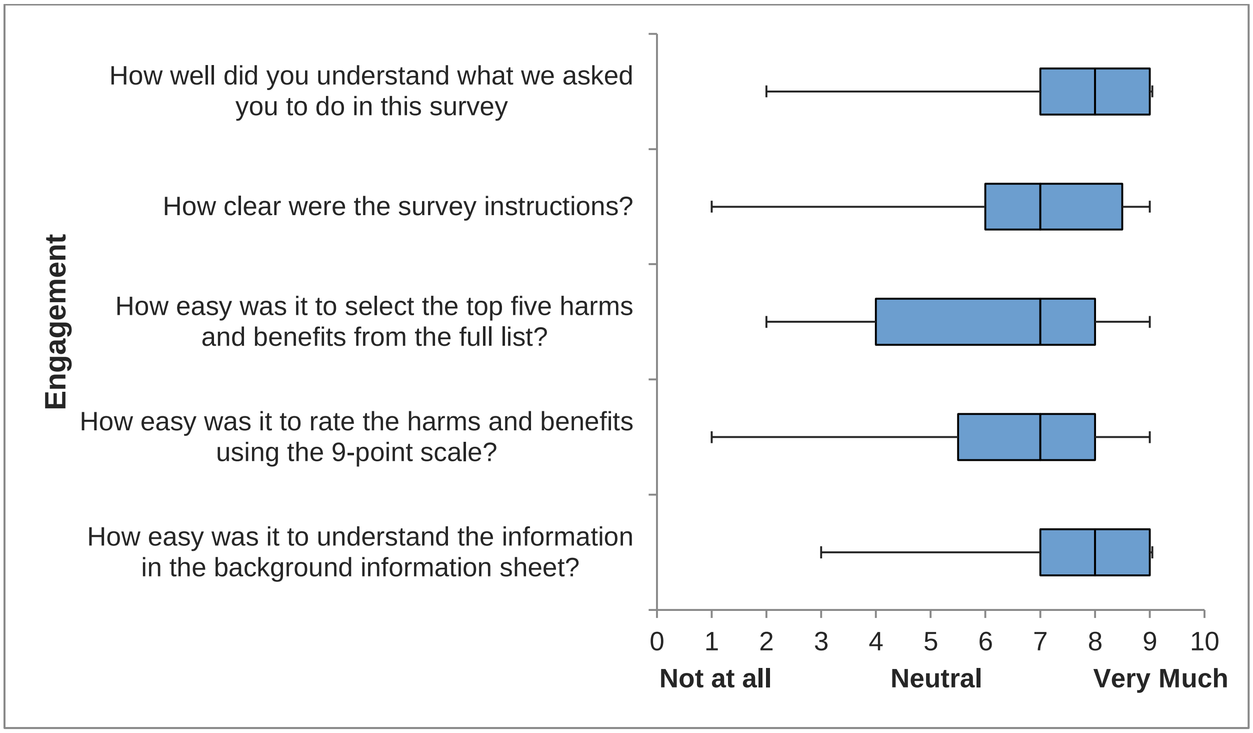

Table 9. Survey responses for experience items (n = 19)

Question	Median	IQR*	Range
How easy was it to understand the information in the background information sheet?	8	7-9	3-9
How easy was it to rate the harms and benefits using the 9-point scale?	7	5.5-8	1-9
How easy was it to select the top five harms and benefits from the full list?	7	4-8	2-9
How clear were the survey instructions?	7	6-8.5	1-9
How well did you understand what we asked you to do in this survey?	8	7-9	2-9

**Note: IQR = interquartile range*

Participants’ overall experience

We conducted four focus groups (*n* = 25), and asked 19 open-ended survey questions (*n* =19) to gather qualitative data from participants about their experience in the project. Table 10 below summarizes participants’ main impressions of the background information sheet, focus group, and survey.

Appendix 6, as supplied by the authors. Appendix to: Thériault G, Limburg H, Klarenbach S, et al.
Recommendations on screening for primary prevention of fragility fractures. CMAJ 2023. doi: 10.1503/cmaj.221219.
Copyright © 2023 The Author(s) or their employer(s). To receive this resource in an accessible format, please contact us at cmajgroup@cmaj.ca.

Table 10. Qualitative data for project experience (n = 25)

Project component	Participants' impressions	Illustrative quotes
**Background information sheet**	Participants found the information presented in the background document clear and understandable.<br><br>One participant noted language barriers, since English is not their first language<br><br>Some participants noted that it would have been helpful to have access to the background information document throughout the survey and focus group, rather than only prior to beginning the survey	*"Perhaps the survey design does not permit this, but it would have been more useful to have the "background information" material on benefits and harms summarized on the survey itself, rather than on a separate sheet. As I was working through the questions, it was easy to forget some of the specific information and definitions in the background sheet." (AdultOsteo_PH1_47)*
	Participants requested statistics on the likelihoods of harms and benefits, including effectiveness of screening tests and treatments.	*"The only issue I had in the harms section is that I would have liked to know the likelihood of false positive or negatives to accurately assess the statements about these issues" (AdultOsteo_PH1_40)*<br><br>*"Provide participants more information on the prevalence of fragility fractures, the success rates of the screening process, the success rate of treatments and the rate of negative effects of the treatments. I feel that my input would have been more rational with this type of info. As is, my input was not well-informed and rather subjective." (AdultOsteo_PH1_45)*
**Focus groups**	**Positive feedback**<br><br>Participants felt their comments were equally considered and valued.<br><br>Several participants expressed that they felt heard, and appreciated the moderator's respectfulness, openness to feedback and impartiality.	*"I was impressed with the discussion format you had developed -- all of the discussion participants felt very comfortable speaking up." (AdultOsteo_PH1_47)*
	Several participants considered the process to be professional and well-organized, with clear communication.<br><br>Several participants appreciated the opportunity to share their viewpoints, as well as found it interesting and helpful to hear different opinions.	*"I liked to be able to contribute - liked listening to the comments and ideas of others and liked having the opportunity to read and learn more about this topic" (AdultOsteo_PH1_40)*

Appendix 6, as supplied by the authors. Appendix to: Thériault G, Limburg H, Klarenbach S, et al.
Recommendations on screening for primary prevention of fragility fractures. CMAJ 2023. doi: 10.1503/cmaj.221219.
Copyright © 2023 The Author(s) or their employer(s). To receive this resource in an accessible format, please contact us at cmajgroup@cmaj.ca.

	**Suggestions for improvement**<br><br>Some participants that were part of the larger focus groups (n >10) felt that not everyone was able to contribute equally<br><br>On the other hand, participants in smaller focus groups (n = 2) felt discussion could have benefited from more participants<br><br>Some participants expressed that a telephone conference may hinder participants' ability to voice their opinions<br><br>A suggestion for improvement was to provide more details on the objective of the focus group beforehand; this could include providing an agenda, or outline of the questions that will be asked prior to the focus group call to allow for more thoughtful responses.	*"The telephone focus group was a bit too large for everyone to have their say on everything" (AdultOsteo_PH1_28)*<br><br>*"I feel the organizers did a very good job, but the medium of a telephone conference with people you don't know presents a bit of a challenge to sharing opinions" (AdultOsteo_PH1_13)*<br><br>*"It would have been helpful for us to have an agenda prior to the meeting, so that we would have known that you wanted to focus discussion on the questions where there was no uniformity in the answers." (AdultOsteo_PH1_47)*
**Overall project experience**	**Positive feedback**<br><br>Participants appreciated that patient preferences are considered in the guideline development process.<br><br>Participants found it interesting to hear the thoughts and opinions of others, as well as the opportunity to contribute their own comments and ideas.<br><br>Participants appreciated the organized coordination and communication of focus group and survey activities.<br><br>Some participants appreciated seeing their results in bar graph form on the participant data summary sheet.	*"I am encouraged that researchers want to engage in the 'sometimes messy' process of engagement with patients but very much appreciate the inclusion of patient views. More patient involvement is sorely needed." (AdultOsteo_PH1_48)*<br><br>*"Just having a voice to help shape policy"(AdultOsteo_PH1_22)*<br><br>*"I enjoy having input into a process which will eventually help others." (AdultOsteo_PH1_28)*
	**Suggestions for improvement**<br><br>Some participants suggested an initial education session for patients prior to completing the survey and focus groups, to promote a common understanding of terms and objectives<br><br>Some participants suggested emphasizing that screening would be done on those showing no	*"Have the focus group (educational session for patients) prior to taking the survey the first time to broaden participant ability to understand the concepts involved." (AdultOsteo_PH1_48)*<br><br>*"Would have liked to have a con call first to go over the objectives, background and q&a." (AdultOsteo_PH1_25)*<br><br>*"I think the word "potential" should be emphasized and that it should be made clearer*

Appendix 6, as supplied by the authors. Appendix to: Thériault G, Limburg H, Klarenbach S, et al.
Recommendations on screening for primary prevention of fragility fractures. CMAJ 2023. doi: 10.1503/cmaj.221219.
Copyright © 2023 The Author(s) or their employer(s). To receive this resource in an accessible format, please contact us at cmajgroup@cmaj.ca.

	symptoms– some in the 'exposed' group expressed confusion as to why they were being asked their opinion on screening, when they've already been diagnosed.<br><br>Some participants found the wording of survey questions unclear (e.g. what do you mean by 'the engagement process'?)<br><br>Some participants suggested separating treatment outcomes from direct screening outcomes; some felt they should be asked as separate questions.<br><br>Some participants suggested way to improve the survey format, such as including open-ended comment boxes for <u>each</u> outcome rating in the survey, so that participants can clearly explain their rating of each particular outcome, as well as a 'back' button to that participants can view their previous answers.<br><br>One participant expressed concern around the usefulness of the data from this patient engagement process, due to confusion surrounding some survey wording and terminology (i.e. screening vs. treatment outcomes).<br><br>Participants felt a more detailed explanation of the goals of the project should be included, including a more in depth explanation of how these results will be used in the process.<br><br>Some participants doubted that their input would influence the final decision making process, and requested more details on how their feedback would be incorporated	*that the screening would be done on persons who had not yet been diagnosed." (AdultOsteo_PH1_28)*<br><br>*"The questions seemed to be too wordy and some sounded the same to me. A bit hard to take it all in." (AdultOsteo_PH1_36)*<br><br>*"Ask one set of questions about whether people would be screened or not, and another set of questions about whether people would be treated or not." (AdultOsteo_PH1_49)*<br><br>*"Also, the questions merged "screening" and "treatment" and as a result some of the questions were harder to understand. While the list of "benefits of screening" was clear, the list of "harms of screening" were mostly theoretical ("overdiagnosis") while the "harms of treatment" were real (side effects of medications)." (AdultOsteo_PH1_47)*<br><br>*"The questionnaire should permit people to go back to see their previous answers." (AdultOsteo_PH1_47)*<br><br>*"I would need someone to convince me that they were going to make changes" (AdultOsteo_PH1_49)*<br><br>*"I would like to have been told a bit more about what any documents would look like after the process was completed." (AdultOsteo_PH1_49)*

Appendix 6, as supplied by the authors. Appendix to: Thériault G, Limburg H, Klarenbach S, et al.
Recommendations on screening for primary prevention of fragility fractures. CMAJ 2023. doi: 10.1503/cmaj.221219.
Copyright © 2023 The Author(s) or their employer(s). To receive this resource in an accessible format, please contact us at cmajgroup@cmaj.ca.

Limitations

In addition to the limitations of the methods discussed in the Patient Engagement Protocol, there were further limitations specific to this project. Our sample is not representative of the target screening population in Canada. The majority (*n* = 23) had a college diploma, bachelor's, graduate, or professional degree. Due to the high education level of participants, these participants may have higher health literacy, different risk factors or protective factors, and/or preferences that differ from the target screening population. Furthermore, the majority of participants lived in urban or suburban areas (*n* = 22), and only two participants identified as Indigenous. As such, the preferences, barriers, and facilitators facing typically underserved groups such as rural Canadians and Indigenous populations are unlikely to be adequately represented in these results.

Based on the focus group discussion and feedback from participants, the results for this project may have additional limitations:

- Participants expressed confusion surrounding the differences between screening and diagnostic testing. For example, several participants spoke of their desire to be screened *if* they were at high risk for fragility fractures. However, the purpose of this particular screening test is to *determine if* someone is at high-risk for fragility fractures; therefore participants would not know if they are at high risk or not prior to screening.
- Participants had difficulty differentiating *treatment* and *screening* outcomes when answering some questions. Many felt that screening and treatment would be separate decisions, and that questions about *treatment* outcomes and preferences should be separate from questions pertaining to *screening* outcomes and preferences.
- The *exposed* group expressed doubts about the usefulness of their contributions, and were confused about whether to consider their screening preferences from the perspective of having no symptoms, or as they are now (with symptoms). Participants wondered about the goal of recruiting *exposed* patients.

These points of potential misinterpretation make it harder to distinguish if differences observed are due to actual differences in preferences and values, or instead due to differences in understanding. Some participants indicated that they felt their interpretations conflicted with others in the group, and this impacted their responses. The CTFPHC may consider these limitations when interpreting the results for their guideline development process.

Suggestions for applying findings

Below are our suggestions for applying the findings from this project to the CTFPHC's guideline regarding screening to prevent fragility fractures:

1. **Include outcomes participants identified as important or critical in the evidence review protocol.** Participants rated all outcomes of screening to determine if someone is at high risk for fragility fractures as either *important* or *critical*. Participants may therefore be more responsive to a guideline that is based on evidence of all outcomes included in this project. Some participants felt that screening and treatment decisions were separate decisions, and that *screening* outcomes and preferences should be addressed separately from *treatment* outcomes and preferences. The CTFPHC may consider addressing screening outcomes and treatment outcomes separately in future patient preferences topics, as well as in the development of KT

Appendix 6, as supplied by the authors. Appendix to: Thériault G, Limburg H, Klarenbach S, et al.
Recommendations on screening for primary prevention of fragility fractures. CMAJ 2023. doi: 10.1503/cmaj.221219.
Copyright © 2023 The Author(s) or their employer(s). To receive this resource in an accessible format, please contact us at cmajgroup@cmaj.ca.

tools. The CTFPHC may also consider including patients at the stage of refining the question for evidence review, as this could lead participant views and feedback being incorporated into Phase 1 of the review and guideline. Differentiating between screening and treatment decisions and outcomes, as well as how the two relate, could be useful to consider as part of a shared-decision making discussion between clinicians and patients.

2. **Provide resources to support a discussion of patients' preferences and shared decision making.** Because the CTFPHC develops evidence-based guidelines, the CTFPHC may not always be able to produce guideline recommendations that are consistent with all patients' preferences. In this case, the CTFPHC may consider developing and disseminating resources that encourage a discussion about patients' preferences and support shared decision-making between clinicians and patients. Specifically, the CTFPHC may produce KT tools that assist clinicians in discussing screening in the context of a patient's preferences. In addition, the CTFPHC may develop KT tools for patients that explain the difference between screening and diagnostic testing, as well as the balance between the harms and benefits of screening.

   Participants noted that preference for screening may be influenced by the availability and type of preventative measures or treatment options, in the event of a screening test that identifies them as high-risk (e.g. exercise, lifestyle changes, medication). The CTFPHC may consider providing information about potential treatment options in the event of a high risk diagnosis, as part of KT tools to facilitate shared decision-making around screening to prevent fragility fractures in the context of each patient's particular circumstances.

3. **Develop KT tools that address information needs of participants.** Participants had additional questions about the specific likelihoods of each outcome, particularly those related to side effects of medications and increased mortality as a direct or indirect result of bone fractures. Participants requested more information on preventative lifestyle changes that could help reduce fragility fractures following a high-risk diagnosis, as well as screening test efficacy. Thus, the guideline and KT tools should integrate relevant information to help patients make an informed choice about screening to determine if they are at high risk for fragility fractures.

   Participants also had different understandings of screening and diagnostic testing, as well as how potential *treatment* outcomes may influence their *screening* decision. The focus group guide may be revised to include a question on whether participants understand the difference between screening and diagnosis, as well as place more emphasis on explaining these key terms in plain language prior to beginning the focus groups. Providing a plain language visual display of the background information, for example a PowerPoint through webconference, may be useful at the start of the focus groups to help participants understand and refer to key background information and terms. An additional suggestion may be to consult patients on the best ways to engage them during the guideline development process (e.g. How do we best explain these concepts? What are the best ways to frame questions and obtain their input?). This could potentially be achieved through a focus group session dedicated to seeking patient input on improving the patient preferences process. The CTFPHC may also consider providing an explanation of the goal of involving *exposed* participants, including how *exposed* participants

Appendix 6, as supplied by the authors. Appendix to: Thériault G, Limburg H, Klarenbach S, et al.
Recommendations on screening for primary prevention of fragility fractures. CMAJ 2023. doi: 10.1503/cmaj.221219.
Copyright © 2023 The Author(s) or their employer(s). To receive this resource in an accessible format, please contact us at cmajgroup@cmaj.ca.

should approach the survey questions (e.g. should they consider their screening preferences from the perspective of having no symptoms, or as they are now, with symptoms).

4. **Send participants a summary of how their feedback in the final guideline and KT tools was used.** Participants answered two engagement questions measuring the extent that they believed that their input, values, and preferences would influence and/or be included in final CTFPHC advice. These ratings were lower than most other engagement questions. Similarly, open-ended responses indicated some participants were not confident that their input would be incorporated or valued. This has been a trend across several guideline data summary reports. It may be useful to provide a more detailed explanation of how the CTFPHC plans to use participant's input prior to the focus groups (e.g. provide specific examples from previous patient preferences that have influenced the final advice and guideline development). Upon public release of the guideline and KT tools, the CTFPHC may send an email to participants to explain how their feedback was integrated into the final guideline and KT tools, also providing specific examples. The CTFPHC may also request that participants complete the participant engagement measure again to explore whether participants' beliefs shifted when presented this information. Involving participants earlier in the guideline development process may also help participants feel that their input is valued, and understand how their values and preferences are incorporated into guideline development. It may also be important to consult participants on how they would like to be engaged, in order to align the engagement process with the needs of participants and shift participants' beliefs on the influence their input and preferences have in final CTFPHC advice and guidelines.

Conclusion

Through this project we explored screening to prevent fragility fractures preferences for a sample of the intervention population to whom the guideline will be relevant (unexposed), as well as those who have previously experienced a fragility fracture and/or have been diagnosed with osteoporosis or osteopenia (exposed). This was the first patient preferences project that separated exposed and unexposed participants in focus groups. In the surveys, participants generally rated the importance of considering benefits of screening higher than harms. Participants also included benefits more often than harms in their top five outcomes to consider when making a decision about being screened to prevent fragility fractures. However, participants rated all outcomes included in the surveys as *important* or *critical.* The majority of participants expressed a preference for screening. Participants who have previously been diagnosed with a fragility fracture and/or have been diagnosed with osteoporosis or osteopenia indicated a slightly stronger preference for screening compared with participants who did not fit those criteria, although a larger sample size would be needed to establish the statistical significance of this observation. Many participants enjoyed the opportunity to participate and found the project interesting. Several participants provided useful, critical feedback that can be used to improve future patient preferences projects. There were some limitations that should be considered in the interpretation of these results, including participant confusion around the definition of screening vs. diagnostic testing, difficulty differentiating between *screening* and *treatment* outcomes and preferences, and confusion about how *exposed* participants were to interpret survey questions (i.e. Should they consider their screening preferences from the perspective of having no symptoms, or as they are now, with

Appendix 6, as supplied by the authors. Appendix to: Thériault G, Limburg H, Klarenbach S, et al.
Recommendations on screening for primary prevention of fragility fractures. CMAJ 2023. doi: 10.1503/cmaj.221219.
Copyright © 2023 The Author(s) or their employer(s). To receive this resource in an accessible format, please contact us at cmajgroup@cmaj.ca.

symptoms). The findings should be integrated into screening to prevent fragility fractures guideline and KT tools, as well as into future CTFPHC patient engagement projects.

Appendix 6, as supplied by the authors. Appendix to: Thériault G, Limburg H, Klarenbach S, et al.
Recommendations on screening for primary prevention of fragility fractures. CMAJ 2023. doi: 10.1503/cmaj.221219.
Copyright © 2023 The Author(s) or their employer(s). To receive this resource in an accessible format, please contact us at cmajgroup@cmaj.ca.

References

1. Fitch K, Bernstein SJ, Aguilar AD, Burnand, B, LaCalle JR, et al. The RAND/UCLA Appropriateness Method user's manual. RAND. 2001.
2. Hseih H, Shannon SE (2005). Three Approaches to Qualitative Content Analysis. Qualtitative Health Research. 15 (9): 1277-1288.
3. Moore, A. Development and Preliminary Evaluation of a Patient and Public Engagement Evaluation Tool. Prepared for the Canadian Task Force for Preventive Health Care, Knowledge Translation Working Group, 2016.

Appendix 6, as supplied by the authors. Appendix to: Thériault G, Limburg H, Klarenbach S, et al.
Recommendations on screening for primary prevention of fragility fractures. CMAJ 2023. doi: 10.1503/cmaj.221219.

Copyright © 2023 The Author(s) or their employer(s). To receive this resource in an accessible format, please contact us at cmajgroup@cmaj.ca.

Appendix A: Screening questionnaire

Introduction
This survey is designed to assess your eligibility for the Canadian Task Force on Preventive Health Care patient preferences project on screening to prevent fragility fractures among adults. Please answer the following questions accurately and honestly.

If you have any questions, concerns, or technical difficulties, please contact Rossella Scoleri, at scolerir@smh.ca.

Please note that the information provided to us through this survey will be kept confidential and will not be shared with anyone outside of the Task Force.

Please enter your first and last name:

____

Please enter your email address:

____

Are you a practicing health care professional?

- ❍ Yes
- ❍ No

Display This Question:

If Are you a practicing health care professional? = Yes

Thank you for taking the time to fill out this survey.
Unfortunately, it appears that you are not eligible to take part in this initiative.
The Task Force is exclusively soliciting the opinions of members of the general public who are not practicing health care professionals.

Appendix 6, as supplied by the authors. Appendix to: Thériault G, Limburg H, Klarenbach S, et al.
Recommendations on screening for primary prevention of fragility fractures. CMAJ 2023. doi: 10.1503/cmaj.221219.
Copyright © 2023 The Author(s) or their employer(s). To receive this resource in an accessible format, please contact us at cmajgroup@cmaj.ca.

Display This Question:

If Are you a practicing health care professional? = Yes

Take Part in Future Projects
The Knowledge Translation Program at St. Michael's Hospital conducts other projects where we involve practicing health care professionals. Even if you are not eligible to take part in this project, you may be able to participate in other current or future projects conducted by the Knowledge Translation Program.
Would you be interested in joining our mailing list for project and research study recruitment? If you indicate yes, we will take this as your consent for your name and email address to be added to our mailing list.

- Yes
- No

Skip To: End of Survey If Take Part in Future Projects The Knowledge Translation Program at St. Michael's Hospital conducts... = Yes

End of Block: Yes, HCP

Start of Block: Eligibility: Age

How old are you?

- 17 years old or younger
- 18-29
- 30-39
- 40-49
- 50-59
- 60-69
- 70-79
- 80 years or older

End of Block: Eligibility: Age

Start of Block: Under 18

Appendix 6, as supplied by the authors. Appendix to: Thériault G, Limburg H, Klarenbach S, et al.
Recommendations on screening for primary prevention of fragility fractures. CMAJ 2023. doi: 10.1503/cmaj.221219.
Copyright © 2023 The Author(s) or their employer(s). To receive this resource in an accessible format, please contact us at cmajgroup@cmaj.ca.

Display This Question:

If How old are you? = 17 years old or younger

Or How old are you? = 18-29

Or How old are you? = 30-39

Or How old are you? = 40-49

Thank you for taking the time to fill out this survey.
Unfortunately, it appears that you are not eligible to take part in this initiative.

At this time, the Task Force is exclusively soliciting the opinions of people aged 50 years of age or older.

Take Part in Future Projects
The Knowledge Translation Program at St. Michael's Hospital conducts other projects where we involve practicing health care professionals. Even if you are not eligible to take part in this project, you may be able to participate in other current or future projects conducted by the Knowledge Translation Program. Would you be interested in joining our mailing list for project and research study recruitment? If you indicate yes, we will take this as your consent for your name and email address to be added to our mailing list.

- Yes
- No

Skip To: End of Survey If Take Part in Future Projects The Knowledge Translation Program at St. Michael's Hospital conducts... = Yes

Skip To: End of Survey If Take Part in Future Projects The Knowledge Translation Program at St. Michael's Hospital conducts... = No

End of Block: Under 18

Start of Block: Eligibility: Conflict of interest

Appendix 6, as supplied by the authors. Appendix to: Thériault G, Limburg H, Klarenbach S, et al.
Recommendations on screening for primary prevention of fragility fractures. CMAJ 2023. doi: 10.1503/cmaj.221219.
Copyright © 2023 The Author(s) or their employer(s). To receive this resource in an accessible format, please contact us at cmajgroup@cmaj.ca.

Do you have any conflict of interest related to osteoporosis? Examples include but are not limited to the following:

- Being a member of an organization related to osteoporosis
- Owning a company that provides products or services related to osteoporosis
- Owning shares in a company that provides products or services related to osteoporosis
- Conducting research on osteoporosis

❍ Yes (please describe) ____

❍ No

End of Block: Eligibility: Conflict of interest

Start of Block: Osteoporosis

Have you ever been diagnosed by a health care provider with one or more of the hormonal problems listed below?

❑ Early menopause (before age 40)

❑ Prolonged unexplained amenorrhea (lack of period not due to menopause, pregnancy or breastfeeding)

❑ Hyperthyroidism (overactive thyroid, high thyroid hormone levels, Graves' disease)

❑ Hyperparathyroidism (high parathyroid hormone levels)

❑ Hypogonadism (low estrogen or low testosterone)

❑ Hypopituitarism (low pituitary hormone levels)

❑ Cushing's syndrome (high cortisol levels)

❑ Adrenal insufficiency (low cortisol, Addison's disease, congenital adrenal hyperplasia)

❑ Hyperprolactinemia (high prolactin levels)

❑ Other hormonal problem (please specify):
____

❑ No, I have not been diagnosed with a hormonal problem

Skip To: Q34 If Have you ever been diagnosed by a health care provider with one or more of the hormonal problems... = Early menopause (before age 40)

Skip To: Q34 If Have you ever been diagnosed by a health care provider with one or more of the hormonal problems... = Prolonged unexplained amenorrhea (lack of period not due to menopause, pregnancy or breastfeeding)

Skip To: Q34 If Have you ever been diagnosed by a health care provider with one or more of the hormonal problems... = Hyperthyroidism (overactive thyroid, high thyroid hormone levels, Graves' disease)

Appendix 6, as supplied by the authors. Appendix to: Thériault G, Limburg H, Klarenbach S, et al.
Recommendations on screening for primary prevention of fragility fractures. CMAJ 2023. doi: 10.1503/cmaj.221219.
Copyright © 2023 The Author(s) or their employer(s). To receive this resource in an accessible format, please contact us at cmajgroup@cmaj.ca.

Skip To: Q34 If Have you ever been diagnosed by a health care provider with one or more of the hormonal problems... = Hyperparathyroidism (high parathyroid hormone levels)

Skip To: Q34 If Have you ever been diagnosed by a health care provider with one or more of the hormonal problems... = Hypogonadism (low estrogen or low testosterone)

Skip To: Q34 If Have you ever been diagnosed by a health care provider with one or more of the hormonal problems... = Hypopituitarism (low pituitary hormone levels)

Skip To: Q34 If Have you ever been diagnosed by a health care provider with one or more of the hormonal problems... = Cushing's syndrome (high cortisol levels)

Skip To: Q34 If Have you ever been diagnosed by a health care provider with one or more of the hormonal problems... = Adrenal insufficiency (low cortisol, Addison's disease, congenital adrenal hyperplasia)

Skip To: Q34 If Have you ever been diagnosed by a health care provider with one or more of the hormonal problems... = Hyperprolactinemia (high prolactin levels)

Skip To: Q59 If Have you ever been diagnosed by a health care provider with one or more of the hormonal problems...(Other hormonal problem (please specify):) Is Not Empty

Skip To: Q59 If Have you ever been diagnosed by a health care provider with one or more of the hormonal problems... = No, I have not been diagnosed with a hormonal problem

Thank you for taking the time to fill out this survey.
Unfortunately, it appears that you are not eligible to take part in this initiative.

Take Part in Future Projects
The Knowledge Translation Program at St. Michael's Hospital conducts other projects where we involve practicing health care professionals. Even if you are not eligible to take part in this project, you may be able to participate in other current or future projects conducted by the Knowledge Translation Program. Would you be interested in joining our mailing list for project and research study recruitment? If you indicate yes, we will take this as your consent for your name and email address to be added to our mailing list.

- Yes
- No

Skip To: End of Survey If Take Part in Future Projects The Knowledge Translation Program at St. Michael's Hospital conducts... = Yes

Skip To: End of Survey If Take Part in Future Projects The Knowledge Translation Program at St. Michael's Hospital conducts... = No

Appendix 6, as supplied by the authors. Appendix to: Thériault G, Limburg H, Klarenbach S, et al.
Recommendations on screening for primary prevention of fragility fractures. CMAJ 2023. doi: 10.1503/cmaj.221219.
Copyright © 2023 The Author(s) or their employer(s). To receive this resource in an accessible format, please contact us at cmajgroup@cmaj.ca.

Have you ever been diagnosed by a health care provider with one or more of the specified bone conditions?

- ❑ Paget’s disease of bone (bone deformities)
- ❑ Osteogenesis imperfecta (brittle bone disease)
- ❑ Osteomalacia (softening of the bones, low vitamin D levels, rickets)
- ❑ Osteitis fibrosa cystica (osteodystrophia fibrosa, weakened bones)
- ❑ Other bone condition (please specify):

____

- ❑ No, I have not been diagnosed with a bone condition

Skip To: Q35 If Have you ever been diagnosed by a health care provider with one or more of the specified bone con... = Paget’s disease of bone (bone deformities)

Skip To: Q35 If Have you ever been diagnosed by a health care provider with one or more of the specified bone con... = Osteogenesis imperfecta (brittle bone disease)

Skip To: Q35 If Have you ever been diagnosed by a health care provider with one or more of the specified bone con... = Osteomalacia (softening of the bones, low vitamin D levels, rickets)

Skip To: Q35 If Have you ever been diagnosed by a health care provider with one or more of the specified bone con... = Osteitis fibrosa cystica (osteodystrophia fibrosa, weakened bones)

Skip To: Q60 If Have you ever been diagnosed by a health care provider with one or more of the specified bone con... = Other bone condition (please specify):

Skip To: Q60 If Have you ever been diagnosed by a health care provider with one or more of the specified bone con... = No, I have not been diagnosed with a bone condition

Thank you for taking the time to fill out this survey.
Unfortunately, it appears that you are not eligible to take part in this initiative.

Take Part in Future Projects
The Knowledge Translation Program at St. Michael’s Hospital conducts other projects where we involve practicing health care professionals. Even if you are not eligible to take part in this project, you may be able to participate in other current or future projects conducted by the Knowledge Translation Program. Would you be interested in joining our mailing list for project and research study recruitment? If you

Appendix 6, as supplied by the authors. Appendix to: Thériault G, Limburg H, Klarenbach S, et al. Recommendations on screening for primary prevention of fragility fractures. CMAJ 2023. doi: 10.1503/cmaj.221219.
Copyright © 2023 The Author(s) or their employer(s). To receive this resource in an accessible format, please contact us at cmajgroup@cmaj.ca.

indicate yes, we will take this as your consent for your name and email address to be added to our mailing list.

- ❍ Yes
- ❍ No

Skip To: End of Survey If Take Part in Future Projects The Knowledge Translation Program at St. Michael's Hospital conducts... = Yes

Skip To: End of Survey If Take Part in Future Projects The Knowledge Translation Program at St. Michael's Hospital conducts... = No

---

Have you used oral* glucocorticoid medications (for example, corticosteroids, cortisone, prednisone, prednisolone) for longer than 3 months"?

*Note: Oral glucocorticoids are taken by mouth and do not include creams or gels applied to the skin (e.g. steroidal creams for rash, psoriasis or other skin conditions) or inhaled medication (e.g. inhaler for asthma or chronic lung disease).

- ❍ Yes
- ❍ No

Skip To: Q36 If Have you used oral glucocorticoid medications (for example, corticosteroids, cortisone, predniso... = Yes*

Skip To: Q23 If Have you used oral glucocorticoid medications (for example, corticosteroids, cortisone, predniso... = No*

Thank you for taking the time to fill out this survey.
Unfortunately, it appears that you are not eligible to take part in this initiative.

Appendix 6, as supplied by the authors. Appendix to: Thériault G, Limburg H, Klarenbach S, et al.
Recommendations on screening for primary prevention of fragility fractures. CMAJ 2023. doi: 10.1503/cmaj.221219.
Copyright © 2023 The Author(s) or their employer(s). To receive this resource in an accessible format, please contact us at cmajgroup@cmaj.ca.

Take Part in Future Projects
The Knowledge Translation Program at St. Michael's Hospital conducts other projects where we involve practicing health care professionals. Even if you are not eligible to take part in this project, you may be able to participate in other current or future projects conducted by the Knowledge Translation Program. Would you be interested in joining our mailing list for project and research study recruitment? If you indicate yes, we will take this as your consent for your name and email address to be added to our mailing list.

- Yes
- No

Skip To: End of Survey If Take Part in Future Projects The Knowledge Translation Program at St. Michael's Hospital conducts... = Yes

Skip To: End of Survey If Take Part in Future Projects The Knowledge Translation Program at St. Michael's Hospital conducts... = No

Do you require dialysis?

- Yes
- No

Skip To: Q37 If Do you require dialysis? = Yes

Skip To: Q31 If Do you require dialysis? = No

Thank you for taking the time to fill out this survey.
Unfortunately, it appears that you are not eligible to take part in this initiative.

Appendix 6, as supplied by the authors. Appendix to: Thériault G, Limburg H, Klarenbach S, et al.
Recommendations on screening for primary prevention of fragility fractures. CMAJ 2023. doi: 10.1503/cmaj.221219.
Copyright © 2023 The Author(s) or their employer(s). To receive this resource in an accessible format, please contact us at cmajgroup@cmaj.ca.

Take Part in Future Projects
The Knowledge Translation Program at St. Michael's Hospital conducts other projects where we involve practicing health care professionals. Even if you are not eligible to take part in this project, you may be able to participate in other current or future projects conducted by the Knowledge Translation Program. Would you be interested in joining our mailing list for project and research study recruitment? If you indicate yes, we will take this as your consent for your name and email address to be added to our mailing list.

- Yes
- No

Skip To: End of Survey If Take Part in Future Projects The Knowledge Translation Program at St. Michael's Hospital conducts... = Yes

Skip To: End of Survey If Take Part in Future Projects The Knowledge Translation Program at St. Michael's Hospital conducts... = No

Have you ever been diagnosed with cancer or received chemotherapy and/or radiation therapy?

- Yes
- No

Skip To: Q38 If Have you ever been diagnosed with cancer or received chemotherapy and/or radiation therapy? = Yes

Skip To: Q24 If Have you ever been diagnosed with cancer or received chemotherapy and/or radiation therapy? = No

Thank you for taking the time to fill out this survey.
Unfortunately, it appears that you are not eligible to take part in this initiative.

Take Part in Future Projects
The Knowledge Translation Program at St. Michael's Hospital conducts other projects where we involve practicing health care professionals. Even if you are not eligible to take part in this project, you may be able to participate in other current or future projects conducted by the Knowledge Translation Program.

Appendix 6, as supplied by the authors. Appendix to: Thériault G, Limburg H, Klarenbach S, et al.
Recommendations on screening for primary prevention of fragility fractures. CMAJ 2023. doi: 10.1503/cmaj.221219.
Copyright © 2023 The Author(s) or their employer(s). To receive this resource in an accessible format, please contact us at cmajgroup@cmaj.ca.

Would you be interested in joining our mailing list for project and research study recruitment? If you indicate yes, we will take this as your consent for your name and email address to be added to our mailing list.

- ❍ Yes
- ❍ No

Skip To: End of Survey If Take Part in Future Projects The Knowledge Translation Program at St. Michael's Hospital conducts... = Yes

Skip To: End of Survey If Take Part in Future Projects The Knowledge Translation Program at St. Michael's Hospital conducts... = No

Have you previously been diagnosed with a fragility fracture?
Fragility fracture is any bone fracture occurring spontaneously or following minor trauma such as a fall from standing height or less.

- ❍ Yes
- ❍ No

Have you been previously and/or currently received a diagnosis of osteoporosis or osteopenia?

- ❍ Yes
- ❍ No

Have you been diagnosed with any chronic health problems (i.e. heart disease, asthma, etc.)

- ❍ Yes (please specify): ____
- ❍ No

End of Block: Osteoporosis

Start of Block: Demographic Questions

Appendix 6, as supplied by the authors. Appendix to: Thériault G, Limburg H, Klarenbach S, et al.
Recommendations on screening for primary prevention of fragility fractures. CMAJ 2023. doi: 10.1503/cmaj.221219.
Copyright © 2023 The Author(s) or their employer(s). To receive this resource in an accessible format, please contact us at cmajgroup@cmaj.ca.

How did you hear about this opportunity?

- ❍ Charity Village
- ❍ Craiglist
- ❍ Kijiji
- ❍ St. Michael's Hospital reached out to me
- ❍ Other, please specify... ____

Which province or territory do you live in?

- ❍ British Columbia
- ❍ Alberta
- ❍ Saskatchewan
- ❍ Manitoba
- ❍ Ontario
- ❍ Quebec
- ❍ New Brunswick
- ❍ Nova Scotia
- ❍ Prince Edward Island
- ❍ Newfoundland and Labrador
- ❍ Yukon Territory
- ❍ Northwest Territories
- ❍ Nunavut

Appendix 6, as supplied by the authors. Appendix to: Thériault G, Limburg H, Klarenbach S, et al.
Recommendations on screening for primary prevention of fragility fractures. CMAJ 2023. doi: 10.1503/cmaj.221219.
Copyright © 2023 The Author(s) or their employer(s). To receive this resource in an accessible format, please contact us at cmajgroup@cmaj.ca.

Which time zone do you live in?

- ❍ Pacific
- ❍ Mountain
- ❍ Central
- ❍ Eastern
- ❍ Atlantic
- ❍ Newfoundland
- ❍ I don't know

Which type of region do you live in?

- ❍ Urban
- ❍ Suburban
- ❍ Rural

What is your gender?

- ❍ Male
- ❍ Female
- ❍ Non-binary
- ❍ Prefer to self-describe ____
- ❍ Prefer not to say

Do you identify as part of one of the following Indigenous groups?

- ❍ First Nations
- ❍ Métis
- ❍ Inuit
- ❍ No, I am not Indigenous

Appendix 6, as supplied by the authors. Appendix to: Thériault G, Limburg H, Klarenbach S, et al.
Recommendations on screening for primary prevention of fragility fractures. CMAJ 2023. doi: 10.1503/cmaj.221219.
Copyright © 2023 The Author(s) or their employer(s). To receive this resource in an accessible format, please contact us at cmajgroup@cmaj.ca.

What is the highest level of education that you have completed?

- Less than high school
- High school
- College diploma or bachelor's degree
- Graduate or professional degree

What is your annual household income?

- less than $30,000
- $30,000-$39,999
- $40,000-$49,999
- $50,000-$59,999
- $60,000-$69,999
- $70,000-$79,999
- $80,000-$89,999
- $90,000-$99,999
- $100,000 or more

End of Block: Demographic Questions

Start of Block: Conclusion

Thank you for taking the time to fill out this survey.
The project team will only contact you by email if you are eligible and space permits to take part in this project.

Take Part in Future Projects
The Knowledge Translation Program at St. Michael's Hospital conducts other projects similar to this. Even if you are not eligible to take part in this project, you may be able to participate in other current or future projects conducted by the Knowledge Translation Program.
Would you be interested in joining our mailing list for project and research study recruitment? If you

Appendix 6, as supplied by the authors. Appendix to: Thériault G, Limburg H, Klarenbach S, et al. Recommendations on screening for primary prevention of fragility fractures. CMAJ 2023. doi: 10.1503/cmaj.221219.
Copyright © 2023 The Author(s) or their employer(s). To receive this resource in an accessible format, please contact us at cmajgroup@cmaj.ca.

indicate yes, we will take this as your consent for your name and email address to be added to our mailing list.

- ❍ Yes
- ❍ No

Appendix 6, as supplied by the authors. Appendix to: Thériault G, Limburg H, Klarenbach S, et al.
Recommendations on screening for primary prevention of fragility fractures. CMAJ 2023. doi: 10.1503/cmaj.221219.
Copyright © 2023 The Author(s) or their employer(s). To receive this resource in an accessible format, please contact us at cmajgroup@cmaj.ca.

Appendix B: Background sheet

Canadian Task Force on Preventive Health Care
Background Information Sheet on Screening to Prevent Fragility Fractures in Adults over age 50

What are fragility fractures?

Fragility fractures are broken bones caused by a minor fall or normal activity that usually should not cause a fracture in healthy adults. These fractures may occur due to weakened bone structure, a condition known as *osteoporosis.* The most common sites of fragility fractures are the hip, spine and wrist.

Fragility fractures are serious injuries that may lead to pain, disability, loss of function, emergency room visits, hospital admission, surgery, complications, or admission to long-term care. Fractures may affect quality of life if fracture patients have a difficult time moving around or if they are unable to care for themselves in the short- or long-term. Fragility fractures are associated with increased risk of death, especially among elderly patients with other chronic diseases like diabetes or heart disease. Hip fractures are particularly associated with increased disability and premature death.

Why do fragility fractures mainly affect adults over age 50?

As people age, old bone may not be replaced by new bone as quickly or effectively, resulting in weak or fragile bones. This reduces bone strength and increases the risk of a broken bone (fracture). The risk of fracture rises with age. Women are more likely to be affected than men because they have lower overall bone density at any age, and because bone loss speeds up after menopause.

How can we check to see if an adult is at risk for fragility fractures?

Different screening tests can be used to help determine if someone is at high risk for fragility fractures. Some screening uses only a questionnaire, while some involve an x-ray test (with or without a questionnaire) to check the level of risk. The x-ray test measures bone mineral density or bone thickness. Measurements are usually taken at the hip and spine, and sometimes at the forearm. A finding of low bone mineral density may help predict a higher risk of fragility fractures but this test might not always be necessary.

What is the benefit of screening to prevent fragility fractures?

Screening may allow health care professionals to identify adults who are at increased risk of fragility fracture. Adults at high risk of fragility fracture may need further testing and may also receive drug treatment. Drug treatment to reduce the risk of fragility fractures involves taking medications that either promote bone formation or decrease bone loss.

What are the potential benefits of treating adults when screening results show they are at risk of fragility fractures?

Appendix 6, as supplied by the authors. Appendix to: Thériault G, Limburg H, Klarenbach S, et al.
Recommendations on screening for primary prevention of fragility fractures. CMAJ 2023. doi: 10.1503/cmaj.221219.
Copyright © 2023 The Author(s) or their employer(s). To receive this resource in an accessible format, please contact us at cmajgroup@cmaj.ca.

Treatment of adults at high risk may lower the number of fragility fractures that these patients experience, or prevent fractures from happening. In doing so, treatment may improve quality of life by reducing fracture-related disability, pain, surgery, complications, hospitalization, and long-term care admissions. Treatment may also lower the chance that a fracture and the disability it causes will contribute to a decline in health for someone who eventually dies of another cause.

What are the potential harms of screening and treating adults to prevent fragility fractures?

There are several potential harms involved with screening and treatment. These could include:

- *False positive test* – being incorrectly identified as high-risk when further testing will show that you are not
- *False negative test* – being incorrectly identified as low-risk when you are actually at high risk
- *Overdiagnosis* – being correctly identified as high risk of a fracture even though you would never have actually experienced a fracture
    - Being overdiagnosed can also lead to *overtreatment*, when you receive treatment for an outcome that you would not have experienced even if you were untreated.
- Side effects from the medications used to lower the risk of fractures are not very common but can sometimes be a problem.
    - The most common non-serious symptoms include nausea, heartburn, abdominal pain, loose bowels, muscle cramps, hot flashes, rashes, or infection.
    - Very rarely other more serious effects may occur, including abnormal leg bone fractures, osteonecrosis (death of bone tissue in the jaw), blood clots, heart problems, and an increased risk of stomach, esophageal, breast, or endometrial cancer.
    - Some patients may choose to stop taking their medication (discontinue treatment) due to the side effects.

Appendix 6, as supplied by the authors. Appendix to: Thériault G, Limburg H, Klarenbach S, et al.
Recommendations on screening for primary prevention of fragility fractures. CMAJ 2023. doi: 10.1503/cmaj.221219.
Copyright © 2023 The Author(s) or their employer(s). To receive this resource in an accessible format, please contact us at cmajgroup@cmaj.ca.

Appendix C: Pre- and post-focus group survey

CTFPHC Survey on Public Perceptions of Screening to Prevent Fragility Fractures among Adults: Survey 2 (post-focus group survey)

Introduction:
The Canadian Task Force on Preventive Health Care (CTFPHC) receives funding from the Public Health Agency of Canada (PHAC) to develop evidence-based clinical practice guidelines for preventive health care in Canada. The CTFPHC has created the following survey to assess how members of the public view screening for osteoporosis to prevent fragility fractures in adults over 50. Getting screened to determine if you are at high risk for fragility fractures has both harms and benefits. In this survey, the CTFPHC would like to know how important you think it is to consider each of these harms and benefits when people make decisions about screening to determine if they are at high risk for fragility fractures. The survey will take approximately 10–15 minutes to complete.

If you have any questions, concerns, or technical difficulties, please contact the research assistant, Rossella Scoleri, at scolerir@smh.ca

Confidentiality Agreement:

The individual acknowledges that information that is considered confidential and/or commercially sensitive (“Confidential Information”) that may be disclosed to them, must remain confidential under all circumstances.

1.The aforementioned individual acknowledges that they will ensure that all persons associated with them, including but not limited to directors, employees or contracted workers, will:(a) keep all documents and information that the above individual may receive from the Public Health Agency of Canada (PHAC) on behalf of the Canadian Task Force on Preventive Health Care (CTFPHC) in the course of carrying out their responsibilities as an above individual, or that the CTFPHC may develop while performing its mandate, strictly confidential;(b) not use any Confidential Information for any purpose other than those indicated by the CTFPHC; (c) Not disclose any Confidential Information to any third party without the prior written consent of the Chair of the CTFPHC, and in the event that such disclosure is permitted, the above individual shall procure that said third party is fully aware of and agrees to be bound by these undertakings.

2. No Waiver of Privilege The above individual acknowledges that the Confidential Information is the property of the CTFPHC (and as some cases may allow, a third party), and that none of the latter intend to and do not waive, any rights, title or privilege they may have in respect of any of the Confidential Information.

3. Specific Exclusions The above individual’s obligation to protect Confidential Information hereunder does not apply to Confidential Information which, even if it may be marked “confidential”, in the following circumstances:(a) IN PUBLIC DOMAIN – the information was legally and legitimately published, or otherwise part of the public domain (unless due to the disclosure or other violation of this

Appendix 6, as supplied by the authors. Appendix to: Thériault G, Limburg H, Klarenbach S, et al. Recommendations on screening for primary prevention of fragility fractures. CMAJ 2023. doi: 10.1503/cmaj.221219.
Copyright © 2023 The Author(s) or their employer(s). To receive this resource in an accessible format, please contact us at cmajgroup@cmaj.ca.

Confidentiality Agreement by the above individual);(b) ALREADY KNOWN TO THE above individual – the information was already in the possession of the above individual at the time of its disclosure to the above individual and was not acquired by the above individual, directly or indirectly, from the CTFPHC, the ERSC nor PHAC;(c) THIRD PARTY DISCLOSES – the information becomes available from an outside source who has a lawful and legitimate right to disclose the information to others;(d) INDEPENDENTLY DEVELOPED – the information was independently developed by the above individual without any of the Confidential Information being reviewed or accessed by the above individual.

4. The above individual acknowledges that there are no conflicts of interest or if there are, that they are indicated on the attached CONFLICT DISCLOSURE form

I acknowledge that I have read and agree to the above Confidentiality Agreement

- Yes
- No

Thank you for taking the time to fill out this survey.

Unfortunately, it appears that you are not eligible to take part in this research project.

The CTFPHC is exclusively soliciting the opinions of members of the general public who have read and agree to the CTFPHC Confidentiality Agreement.

Participant ID:

Please enter your participant ID in the box below. You can find your participant ID in the email that you received from Rossella Scoleri with the link to the survey.

____

Date:

____

Before you begin the survey, please take the time to read the Background Information Sheet:

I have read the Background Information Sheet and am ready to proceed with the survey.

Appendix 6, as supplied by the authors. Appendix to: Thériault G, Limburg H, Klarenbach S, et al. Recommendations on screening for primary prevention of fragility fractures. CMAJ 2023. doi: 10.1503/cmaj.221219.
Copyright © 2023 The Author(s) or their employer(s). To receive this resource in an accessible format, please contact us at cmajgroup@cmaj.ca.

❍ I agree

Screening to Prevent Fragility Fractures among Adults over 50:

Below is a series of statements about the potential **benefits** that adults over 50 may experience after being screened to prevent fragility fractures.

For each statement, please rate how much it would influence your decision on whether or not to be screened to prevent fragility fractures.

RECALL: Screening is using one or more tests for all patients, even if they are not seeking help with any particular symptoms, to help identify a condition or illness in some. An example of a screening test to determine if someone is at high risk for fragility fractures would be a standard questionnaire, or an x-ray to measure bone density or bone thickness, or both.

If you were making a decision on whether or not to be screened to determine if you are at high risk for fragility fractures, how important would these outcomes be for you?

1-3 not important for decision-making
4-6 important for decision-making
7-9 critical for decision-making

	1	Not important for decision-making 2	3	4	Important for decision-making 5	6	7	Critical for decision-making 8	9
Screening may decrease the number of hip bone fractures that someone will experience	❍	❍	❍	❍	❍	❍	❍	❍	❍
Screening may decrease the	❍	❍	❍	❍	❍	❍	❍	❍	❍

Appendix 6, as supplied by the authors. Appendix to: Thériault G, Limburg H, Klarenbach S, et al.
Recommendations on screening for primary prevention of fragility fractures. CMAJ 2023. doi: 10.1503/cmaj.221219.
Copyright © 2023 The Author(s) or their employer(s). To receive this resource in an accessible format, please contact us at cmajgroup@cmaj.ca.

number of broken bones caused by a minor fall or normal activity that usually should not cause a fracture in healthy adults									
Screening may decrease the chance of death **<u>directly</u>** related to a bone fracture	❍	❍	❍	❍	❍	❍	❍	❍	❍
Screening may decrease the chance of death from causes or conditions that may be **<u>indirectly</u>** related to, or worsened by, a bone fracture	❍	❍	❍	❍	❍	❍	❍	❍	❍
Screening that leads to a decrease in fractures may improve overall well-being and quality of life	❍	❍	❍	❍	❍	❍	❍	❍	❍
Screening that leads to a	❍	❍	❍	❍	❍	❍	❍	❍	❍

St. Michael's
Inspired Care.
Inspiring Science.

Appendix 6, as supplied by the authors. Appendix to: Thériault G, Limburg H, Klarenbach S, et al. Recommendations on screening for primary prevention of fragility fractures. CMAJ 2023. doi: 10.1503/cmaj.221219.
Copyright © 2023 The Author(s) or their employer(s). To receive this resource in an accessible format, please contact us at cmajgroup@cmaj.ca.

decrease in fractures may improve your ability to perform every-day activities. This can include decreased admissions to long-term care

If you would like to provide any comments about your rating, please enter them in the space below.

____

Screening to Prevent Fragility Fractures among Adults over 50

Below is a series of statements about the potential **harms** that adults over 50 may experience after being screened to prevent fragility fractures.

For each statement, please rate how much it would influence your decision on whether or not to be screened to prevent fragility fractures.

RECALL: Screening is using one or more tests for all patients, even if they are not seeking help with any particular symptoms, to help identify a condition or illness in some. An example of a screening test to determine if someone is at high risk for fragility fractures would be a standard questionnaire, or an x-ray to measure bone density or bone thickness, or both.

If you were making a decision on whether or not to be screened to determine if you are at high risk for fragility fractures, how important would these outcomes be for you?

1-3 not important for decision-making
4-6 important for decision-making
7-9 critical for decision-making

Appendix 6, as supplied by the authors. Appendix to: Thériault G, Limburg H, Klarenbach S, et al.
Recommendations on screening for primary prevention of fragility fractures. CMAJ 2023. doi: 10.1503/cmaj.221219.
Copyright © 2023 The Author(s) or their employer(s). To receive this resource in an accessible format, please contact us at cmajgroup@cmaj.ca.

	1	Not important for decision-making 2	3	4	Important for decision-making 5	6	7	Critical for decision-making 8	9
Screening may lead to treatment with medications used to lower the risk of fractures. These medications can have unwanted, but not serious, side effects. This could include nausea, heartburn, abdominal pain, loose bowels, rashes, muscle cramps or non-serious infections	❍	❍	❍	❍	❍	❍	❍	❍	❍
Screening may result in treatment with medications used to lower the risk of fractures. In some cases treatment is **<u>stopped</u>** because of unwanted side effects of these medications	❍	❍	❍	❍	❍	❍	❍	❍	❍
Screening may lead to treatment with medications used to lower the risk of fractures. In very rare	❍	❍	❍	❍	❍	❍	❍	❍	❍

Appendix 6, as supplied by the authors. Appendix to: Thériault G, Limburg H, Klarenbach S, et al.
Recommendations on screening for primary prevention of fragility fractures. CMAJ 2023. doi: 10.1503/cmaj.221219.
Copyright © 2023 The Author(s) or their employer(s). To receive this resource in an accessible format, please contact us at cmajgroup@cmaj.ca.

cases, medication can have serious side effects, including death or serious illness.									
Screening may correctly identify you as being at high risk of a fracture, even though you would never have actually experienced a fracture in your lifetime. This can lead to unnecessary tests, treatments, worry and concern (called over-diagnosis)	❍	❍	❍	❍	❍	❍	❍	❍	❍
Screening may result in treatment to prevent fragility fractures when there is little or no evidence that the benefits of treatment would outweigh the harms of treatment (called overtreatment).	❍	❍	❍	❍	❍	❍	❍	❍	❍
Screening may result in incorrectly identifying someone as being high-risk for fractures, when they are actually not at high-risk for	❍	❍	❍	❍	❍	❍	❍	❍	❍

Appendix 6, as supplied by the authors. Appendix to: Thériault G, Limburg H, Klarenbach S, et al.
Recommendations on screening for primary prevention of fragility fractures. CMAJ 2023. doi: 10.1503/cmaj.221219.
Copyright © 2023 The Author(s) or their employer(s). To receive this resource in an accessible format, please contact us at cmajgroup@cmaj.ca.

fractures (called a false positive result)									
Screening may result in incorrectly identifying someone as being low-risk for fractures, when they are actually at a high-risk for fractures (called a false negative)	❍	❍	❍	❍	❍	❍	❍	❍	❍

If you would like to provide any comments about your rating, please enter them in the space below.

____

Screening to Prevent Fragility Fractures amoung adults over 50:

Below is the same list of statements about potential harms and benefits of screening to prevent fragility fractures you just rated. Please select **five** items on this list that you think are most critical to consider when adults make decisions about screening to prevent fragility fractures.

Indicate your response by clicking on the statement that you wish to select.

Please do not select more than five items.

Appendix 6, as supplied by the authors. Appendix to: Thériault G, Limburg H, Klarenbach S, et al.
Recommendations on screening for primary prevention of fragility fractures. CMAJ 2023. doi: 10.1503/cmaj.221219.
Copyright © 2023 The Author(s) or their employer(s). To receive this resource in an accessible format, please contact us at cmajgroup@cmaj.ca.

Below is the same list of statements about the potential harms and benefits of screening to prevent fragility fractures among adults that you just rated. Please select **five** items on this list that you think are most critical to consider when people make decisions about screening to prevent fragility fractures.

Indicate your response by clicking on the statement that you wish to select.

Please do not select more than five items.

- ❑ Screening may decrease the number of hip bone fractures that someone will experience
- ❑ Screening may decrease the number of broken bones caused by a minor fall or normal activity that usually should not cause a fracture in healthy adults
- ❑ Screening may decrease the chance of death **directly** related to a bone fracture.
- ❑ Screening may decrease the chance of death from causes or conditions that may be **indirectly** related to, or worsened by, a bone fracture.
- ❑ Screening that leads to a decrease in fractures may improve overall well-being and quality of life.
- ❑ Screening that leads to a decrease in fractures may improve your ability to perform every-day activities. This can include decreased admissions to long-term care
- ❑ Screening may lead to treatment with medications used to lower the risk of fractures. These medications can have unwanted, but not serious, side effects. This could include nausea, heartburn, abdominal pain, loose bowels, rashes, muscle cramps or non-serious infections.
- ❑ Screening may result in starting treatment with medications used to lower the risk of fractures. In some cases treatment is **stopped** because of unwanted side effects of these medications.
- ❑ Screening may lead to treatment with medications used to lower the risk of fractures. In very rare cases, medication can have serious side effects, including death or serious illness.
- ❑ Screening may correctly identify you as being at high risk of a fracture, even though you would never have actually experienced a fracture in your lifetime. This can lead to unnecessary tests, treatments, worry and concern (called over-diagnosis).
- ❑ Screening may result in treatment to prevent fragility fractures when there is little or no evidence that the benefits of treatment would outweigh the harms of treatment (called overtreatment).
- ❑ Screening may result in incorrectly identifying someone as being high-risk for fractures, when they are actually not at high-risk for fractures (called a false positive result).
- ❑ Screening may result in incorrectly identifying someone as being low-risk for fractures, when they are actually at a high-risk for fractures (called a false negative).

Appendix 6, as supplied by the authors. Appendix to: Thériault G, Limburg H, Klarenbach S, et al.
Recommendations on screening for primary prevention of fragility fractures. CMAJ 2023. doi: 10.1503/cmaj.221219.
Copyright © 2023 The Author(s) or their employer(s). To receive this resource in an accessible format, please contact us at cmajgroup@cmaj.ca.

If you would like to provide any comments about your rating, please enter them in the space below.

In the space below, please list any additional harms and benefits of screening that did not appear on the rating list but that you think are critical for adults to consider when making a decision about whether or not to be screened to determine if they are at high risk for fragility fractures.

Recall: Screening is using one or more tests for all patients, even if they are not seeking help with any particular symptoms, to help identify a condition or illness in some. An example of a screening test to determine if someone is at high risk for fragility fractures would be a standard questionnaire, or an x-ray to measure bone density or bone thickness, or both.

Considering the potential harms and benefits of screening to determine if someone is at high risk for fragility fractures, how much would you want to be **screened**?

	Not at all 1	2	3	4	Neutral 5	10	6	7	8	Very much 9
I would want to be screened to determine if I am at high risk for fragility fractures	❍	❍	❍	❍	❍	❍	❍	❍	❍	❍

If you would like to provide any comments about your rating, please enter them in the space below:

We will now ask you some questions about your experience participating in this project.

Appendix 6, as supplied by the authors. Appendix to: Thériault G, Limburg H, Klarenbach S, et al.
Recommendations on screening for primary prevention of fragility fractures. CMAJ 2023. doi: 10.1503/cmaj.221219.
Copyright © 2023 The Author(s) or their employer(s). To receive this resource in an accessible format, please contact us at cmajgroup@cmaj.ca.

In the space below, please briefly summarize the tasks that we asked you to perform in this survey.

____

	Not at all 1	2	3	4	Neutral 5	6	7	8	Very much 9
How easy was it to understand the information in the background information sheet?	❍	❍	❍	❍	❍	❍	❍	❍	❍
How easy was it to rate the harms and benefits using the 9-point scale?	❍	❍	❍	❍	❍	❍	❍	❍	❍
How easy was it to select the top five harms and benefits from the full list?	❍	❍	❍	❍	❍	❍	❍	❍	❍
How clear were the survey responses?	❍	❍	❍	❍	❍	❍	❍	❍	❍
How well did you understand what we asked you to do in this survey	❍	❍	❍	❍	❍	❍	❍	❍	❍

In the space provided, please describe anything we could do to make the survey tasks easier to complete:

____

Appendix 6, as supplied by the authors. Appendix to: Thériault G, Limburg H, Klarenbach S, et al. Recommendations on screening for primary prevention of fragility fractures. CMAJ 2023. doi: 10.1503/cmaj.221219.
Copyright © 2023 The Author(s) or their employer(s). To receive this resource in an accessible format, please contact us at cmajgroup@cmaj.ca.

Please describe anything that we could change to improve this project:

____

Please describe what you liked about taking part in this project:

____

Please describe what you did not like about taking part in this project:

____

Demographic Information

What is your age?

____

What is your gender?

____

What is your ethnicity?

____

Appendix 6, as supplied by the authors. Appendix to: Thériault G, Limburg H, Klarenbach S, et al.
Recommendations on screening for primary prevention of fragility fractures. CMAJ 2023. doi: 10.1503/cmaj.221219.
Copyright © 2023 The Author(s) or their employer(s). To receive this resource in an accessible format, please contact us at cmajgroup@cmaj.ca.

Which province or territory do you live in?

- ❍ British Columbia
- ❍ Alberta
- ❍ Saskatchewan
- ❍ Manitoba
- ❍ Ontario
- ❍ Quebec
- ❍ New Brunswick
- ❍ Nova Scotia
- ❍ Prince Edward Island
- ❍ Newfoundland and Labrador
- ❍ Yukon Territory
- ❍ Northwest Territories
- ❍ Nunavut

Next Steps:

Thank you for completing this second survey. If you have questions about any part of the project, please contact Rossella Scoleri at scolerir@smh.ca.

We will now process your honorarium payment. Please note that it may take up to 45 days for you to receive your payment by mail after we submit it for processing.

Once the data have been analyzed, you will be sent a summary report that details the findings from this project. You will then be invited to participate in an optional debrief teleconference to discuss the project findings. Once the CTFPHC publishes its guideline, you will also be sent a copy of the guideline and the accompanying knowledge translation tools.

Thank you for your participation in this project!

Appendix 6, as supplied by the authors. Appendix to: Thériault G, Limburg H, Klarenbach S, et al.
Recommendations on screening for primary prevention of fragility fractures. CMAJ 2023. doi: 10.1503/cmaj.221219.
Copyright © 2023 The Author(s) or their employer(s). To receive this resource in an accessible format, please contact us at cmajgroup@cmaj.ca.

Appendix D: Sample personalized response sheet

CTFPHC Survey on Public Perceptions of Screening for Fragility Fractures

Personalized Rating Sheet

Survey 1

Prepared for Participant Number MASTER (exposed group)

Introduction

A total of 16 people from across Canada completed the CTFPHC Survey on Public Perceptions of Screening for Fragility Fractures in Adults. This sheet provides a summary of the survey responses.

For each survey question you answered, you will see a separate bar graph. We have shown your individual answer along with a summary of the answers from all of the participants. This way you can have a record of your responses and can also see what your peers answered for each question.

Outcomes Scale Ratings

This section provides information about how to read the ratings that participants provided in the survey.

For each of these potential outcomes, also referred to as harms and benefits, all participants were provided with information about the outcome and asked "If you were making a decision on whether or not to be screened for fragility fractures, how important would these outcomes be for you?"

Participants could rate the importance of the information from 1-9:

- 1-3 - not important to my decision to be screened or not
- 4-6 - important to my decision to be screened or not
- 7-9 - critical to my decision to be screened or not

Appendix 6, as supplied by the authors. Appendix to: Thériault G, Limburg H, Klarenbach S, et al.
Recommendations on screening for primary prevention of fragility fractures. CMAJ 2023. doi: 10.1503/cmaj.221219.
Copyright © 2023 The Author(s) or their employer(s). To receive this resource in an accessible format, please contact us at cmajgroup@cmaj.ca.

EXAMPLE: How to read the graph

Sample Outcomes Scale Rating

Here is a sample of a graph and what the different parts mean:

Sample Survey Outcome: *Description of the potential harm or benefit*

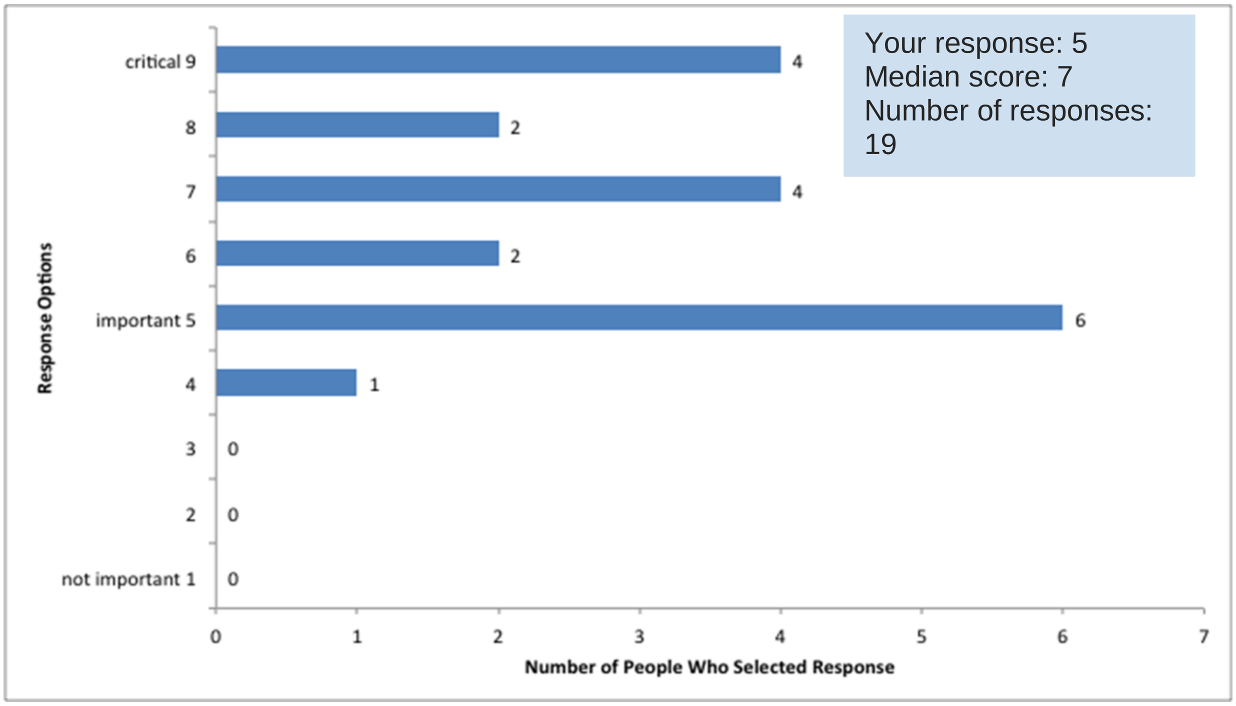

At the top of the graph you will see which potential harm or benefit this graph is about.

Along the *y*-axis of the graph (the vertical axis, running top to bottom), you will see all possible numbers on the rating scale that participants could use to rate the outcome.

Along the *x*-axis of the graph (the horizontal axis, running left to right), you will see numbers which show how many participants chose each number on the rating scale.

The box in the upper-right corner contains three pieces of information:

- The number on the rating scale that you selected for this outcome
- The median rating for this outcome across all participants (you can think of this like an "average" of the ratings selected by all participants)
- The total number of participants who rated this outcome

In this example, four participants rated the question with a "9", two participants rated it an "8", four participants rated it a "7", two participants rated it a "6", six participants rated it a "5", one participant [illegible]

Appendix 6, as supplied by the authors. Appendix to: Thériault G, Limburg H, Klarenbach S, et al.
Recommendations on screening for primary prevention of fragility fractures. CMAJ 2023. doi: 10.1503/cmaj.221219.
Copyright © 2023 The Author(s) or their employer(s). To receive this resource in an accessible format, please contact us at cmajgroup@cmaj.ca.

Your personalized answers are broken down by potential harms and benefits for fragility fracture screening below.

Summary of Outcomes Ratings

1. **Survey Benefit:** Screening may decrease the number of hip bone fractures that someone will experience.

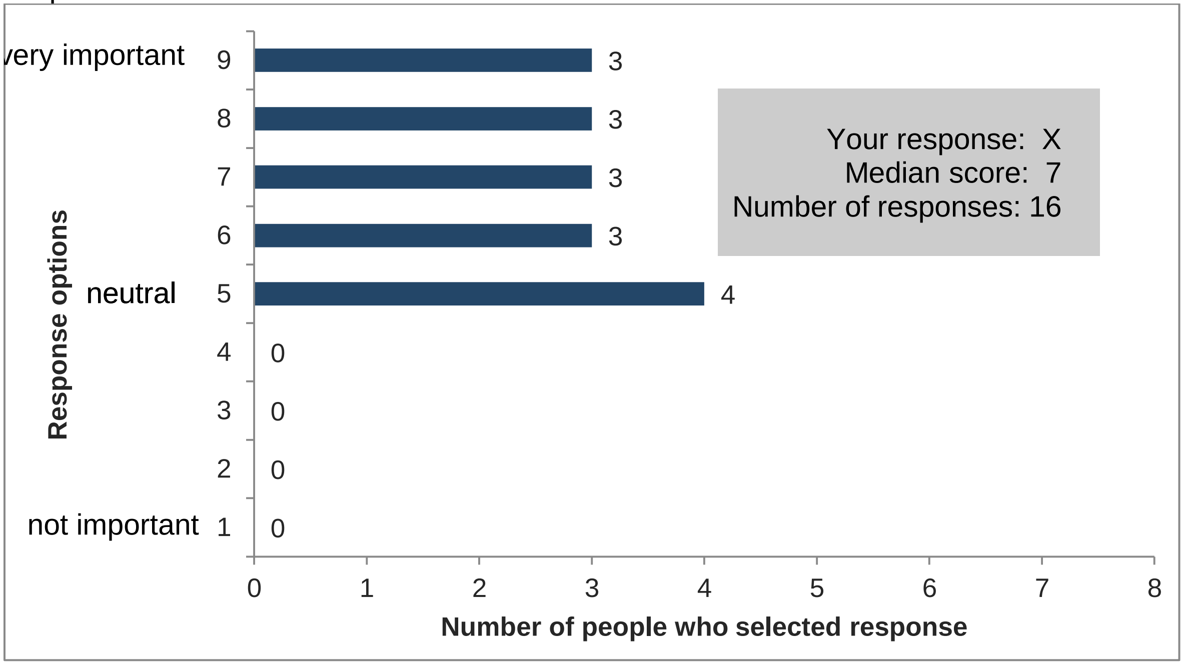

2. **Survey Benefit:** Screening may decrease the number of broken bones caused by a minor fall or normal activity that usually should not cause a fracture in healthy adults.

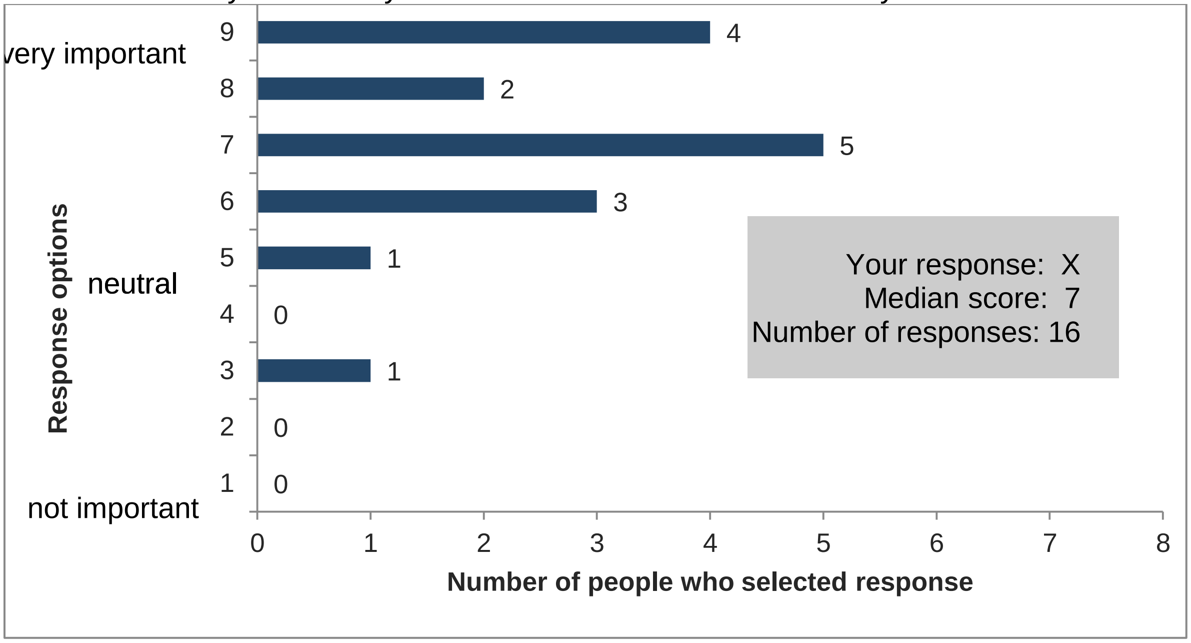

Appendix 6, as supplied by the authors. Appendix to: Thériault G, Limburg H, Klarenbach S, et al. Recommendations on screening for primary prevention of fragility fractures. CMAJ 2023. doi: 10.1503/cmaj.221219.
Copyright © 2023 The Author(s) or their employer(s). To receive this resource in an accessible format, please contact us at cmajgroup@cmaj.ca.

3. **Survey Benefit:** Screening may decrease the chance of death directly related to a bone fracture.

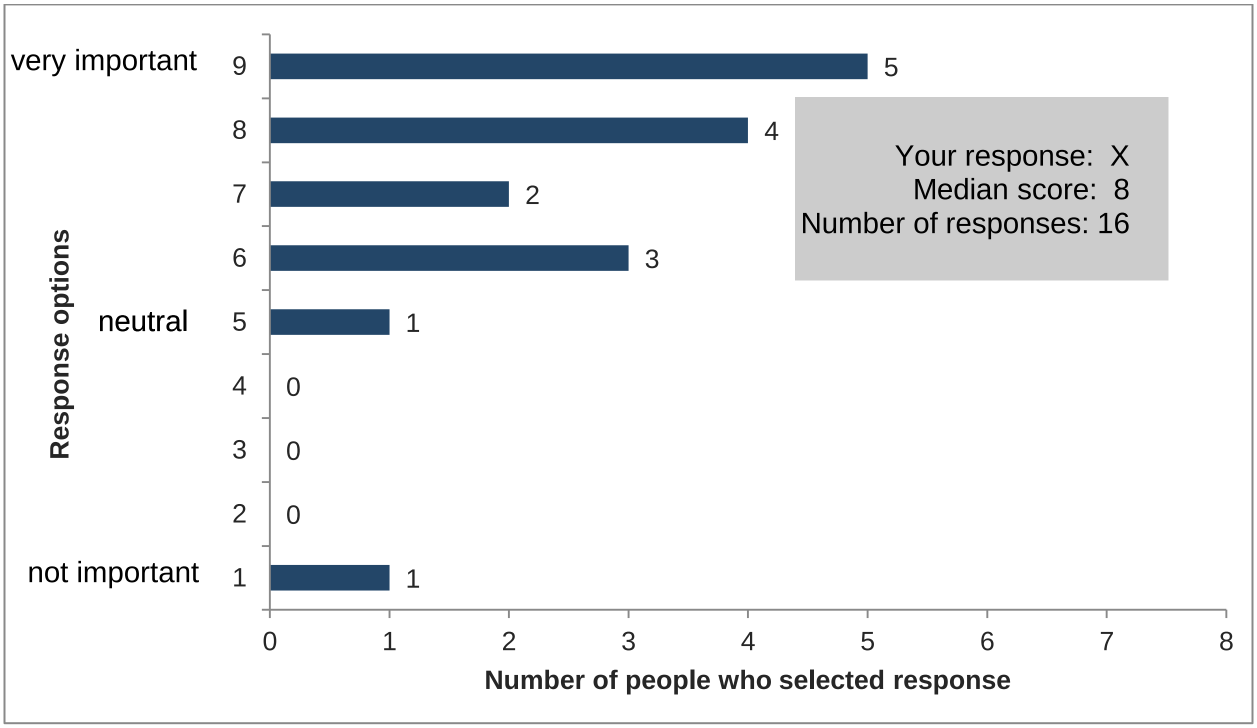

4. **Survey Benefit:** Screening may decrease the chance of death from causes or conditions that may be indirectly related to, or worsened by, a bone fracture.

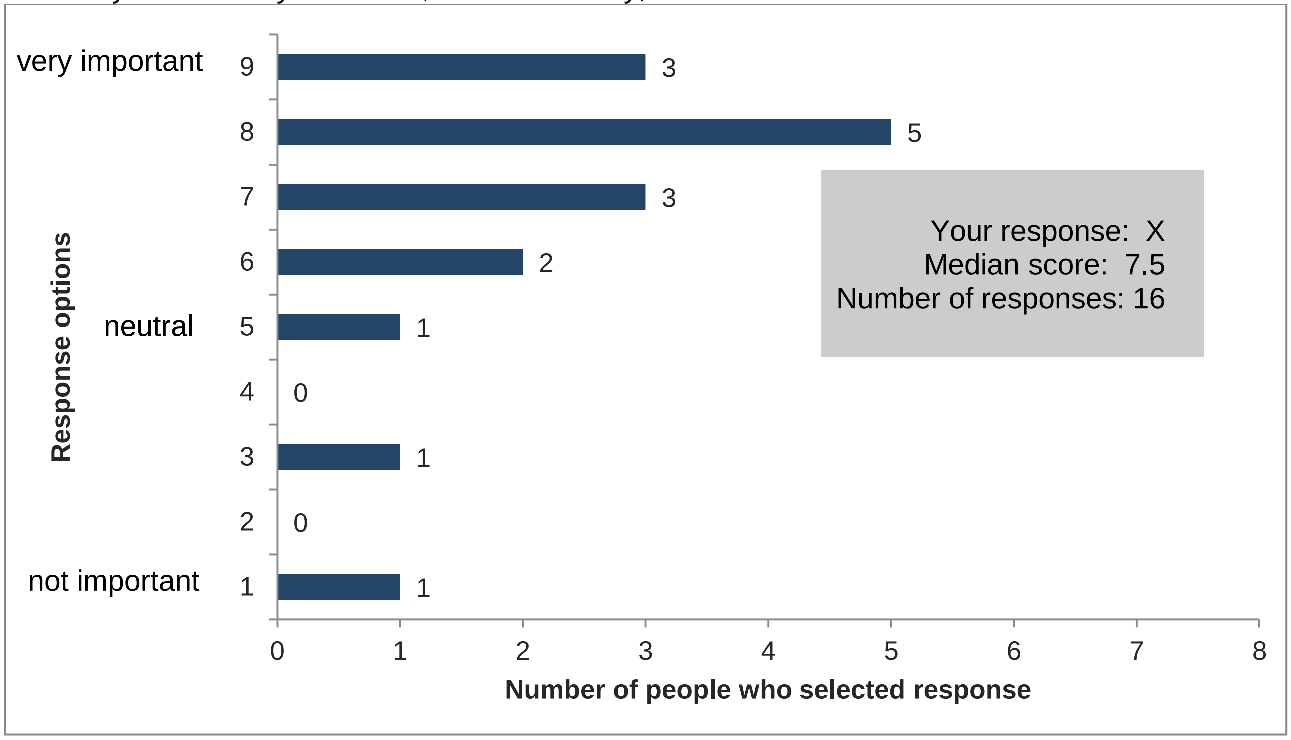

Appendix 6, as supplied by the authors. Appendix to: Thériault G, Limburg H, Klarenbach S, et al.
Recommendations on screening for primary prevention of fragility fractures. CMAJ 2023. doi: 10.1503/cmaj.221219.

Copyright © 2023 The Author(s) or their employer(s). To receive this resource in an accessible format, please contact us at cmajgroup@cmaj.ca.

5. **Survey Benefit:** Screening that leads to a decrease in fractures may improve overall well-being and quality of life.

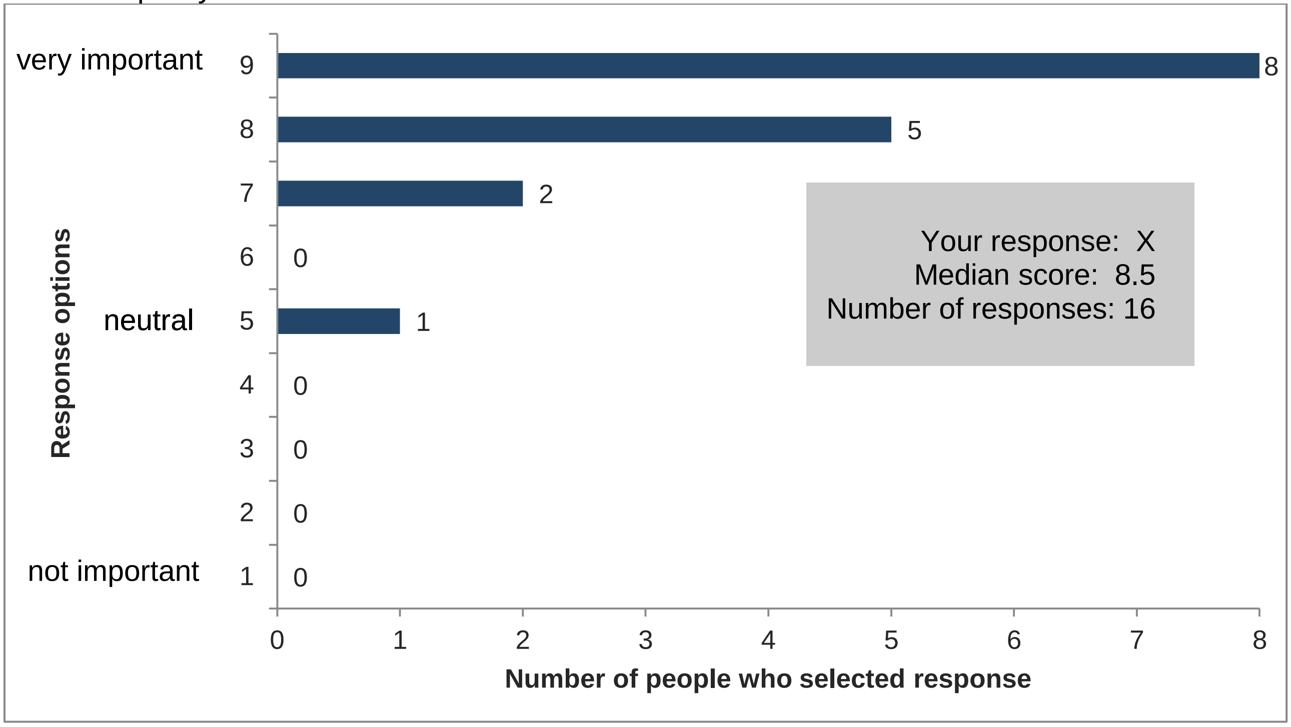

6. **Survey Benefit:** Screening that leads to a decrease in fractures may improve your ability to perform every-day activities. This can include decreased admissions to long-term care.

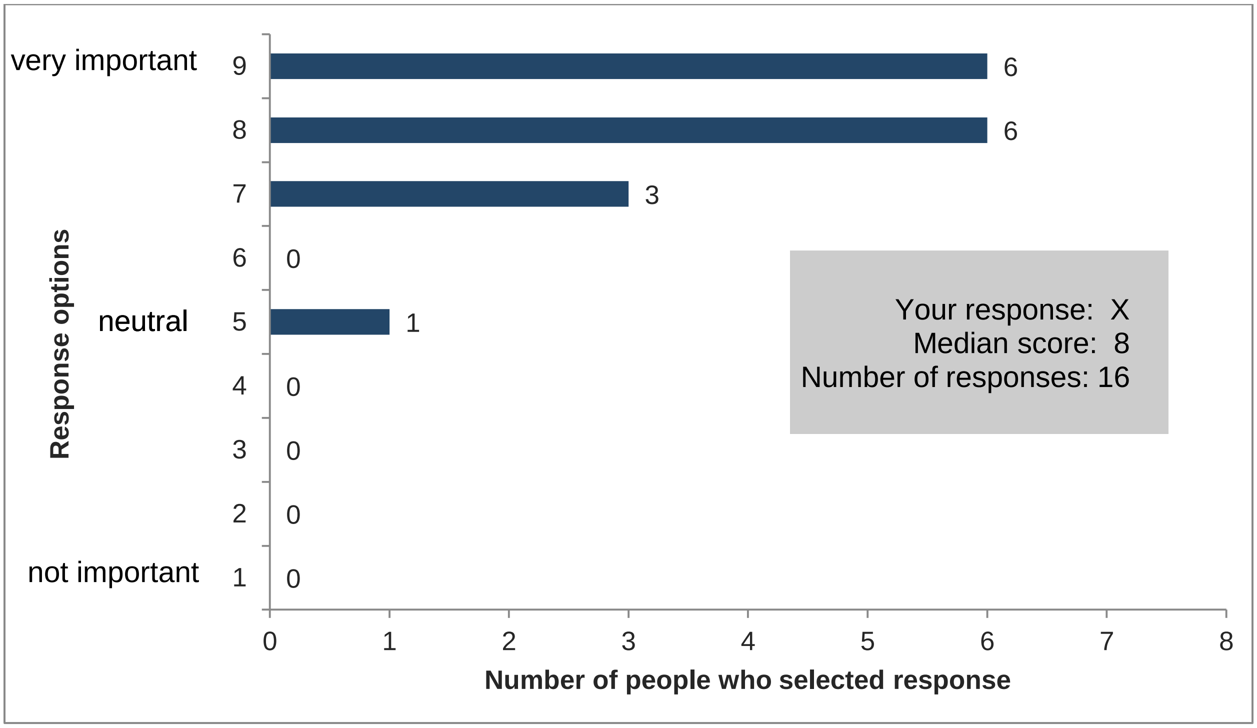

Appendix 6, as supplied by the authors. Appendix to: Thériault G, Limburg H, Klarenbach S, et al.
Recommendations on screening for primary prevention of fragility fractures. CMAJ 2023. doi: 10.1503/cmaj.221219.
Copyright © 2023 The Author(s) or their employer(s). To receive this resource in an accessible format, please contact us at cmajgroup@cmaj.ca.

7. **Survey Harm:** Screening may lead to treatment with medications used to lower the risk of fractures. These medications can have unwanted, but not serious, side effects. This could include nausea, heartburn, abdominal pain, loose bowels, rashes, muscle cramps or non-serious infections.

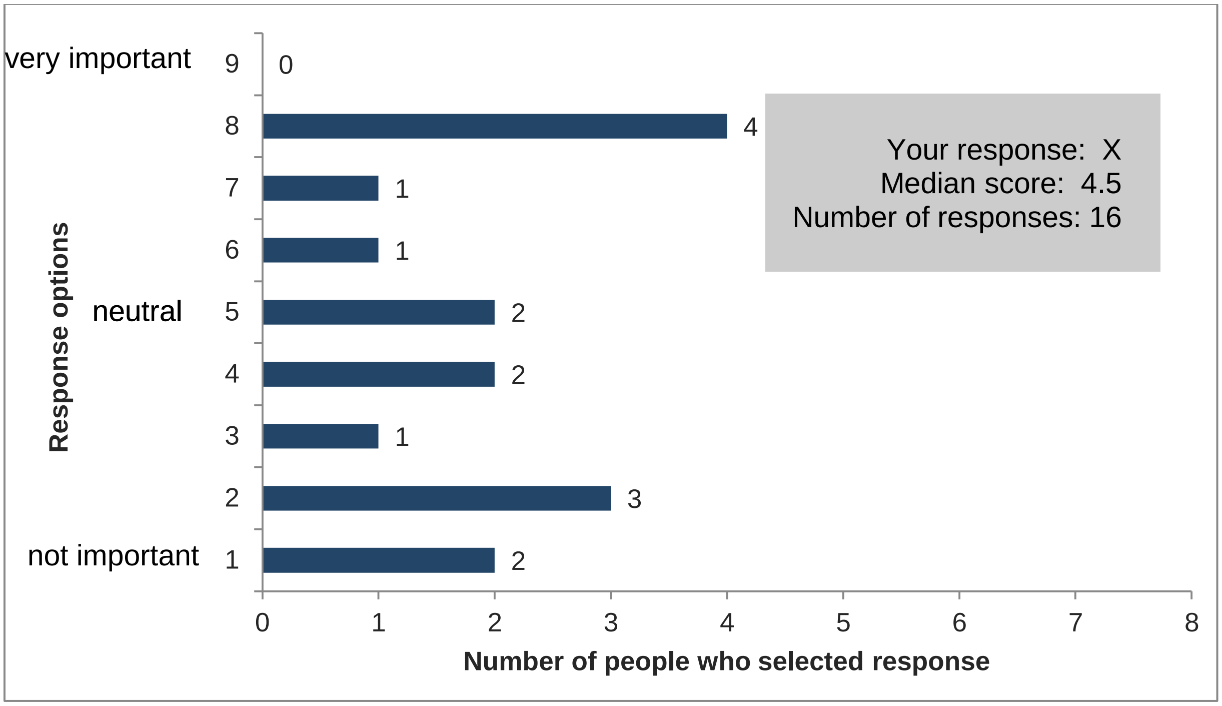

8. **Survey Harm:** Screening may result in treatment with medications used to lower the risk of fractures. In some cases treatment is stopped because of unwanted side effects of these medications.

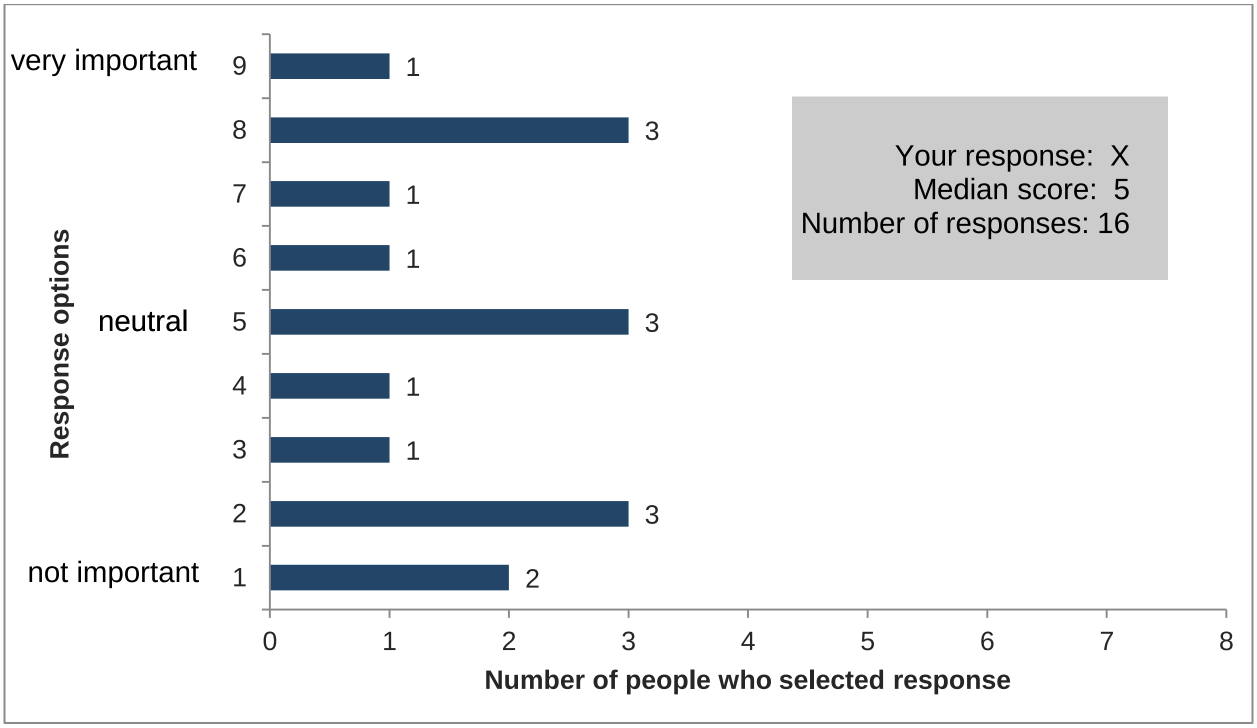

Appendix 6, as supplied by the authors. Appendix to: Thériault G, Limburg H, Klarenbach S, et al.
Recommendations on screening for primary prevention of fragility fractures. CMAJ 2023. doi: 10.1503/cmaj.221219.

Copyright © 2023 The Author(s) or their employer(s). To receive this resource in an accessible format, please contact us at cmajgroup@cmaj.ca.

9. **Survey Harm:** Screening may lead to treatment with medications used to lower the risk of fractures. In very rare cases, medication can have serious side effects, including death or serious illness.

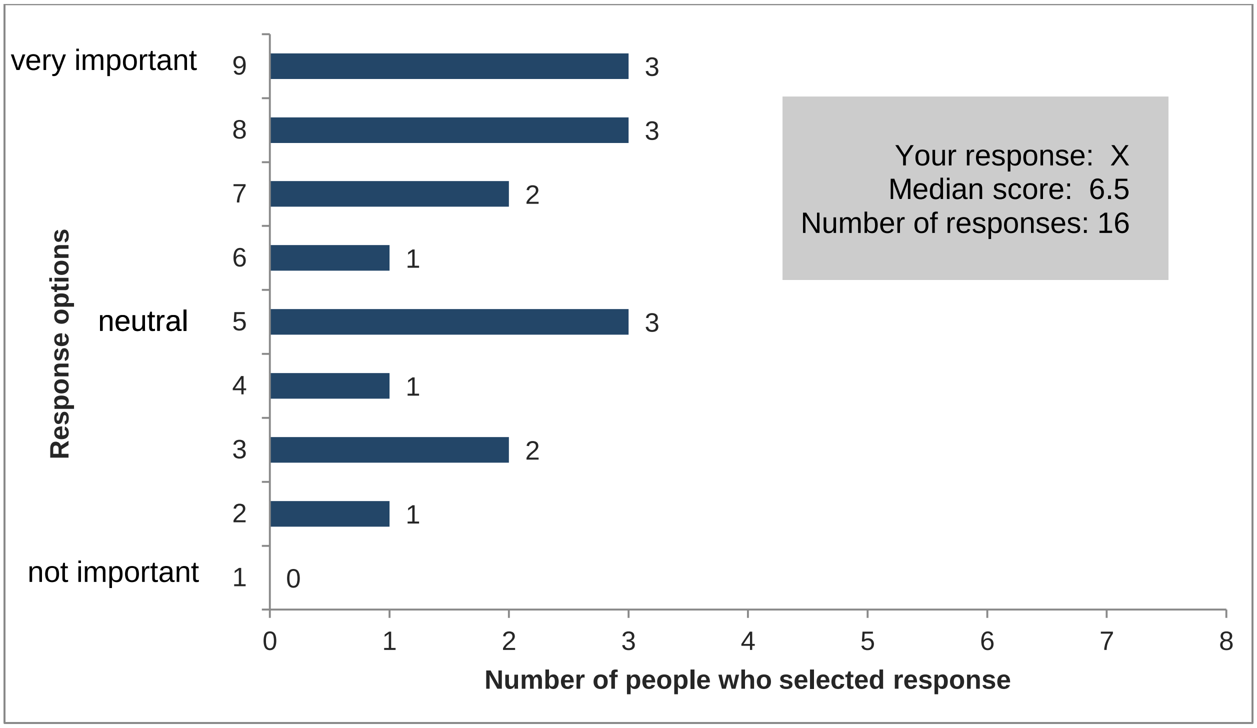

10. **Survey Harm:** Screening may correctly identify you as being at high risk of a fracture, even though you would never have actually experienced a fracture in your lifetime. This can lead to unnecessary tests, treatments, worry and concern (called over-diagnosis).

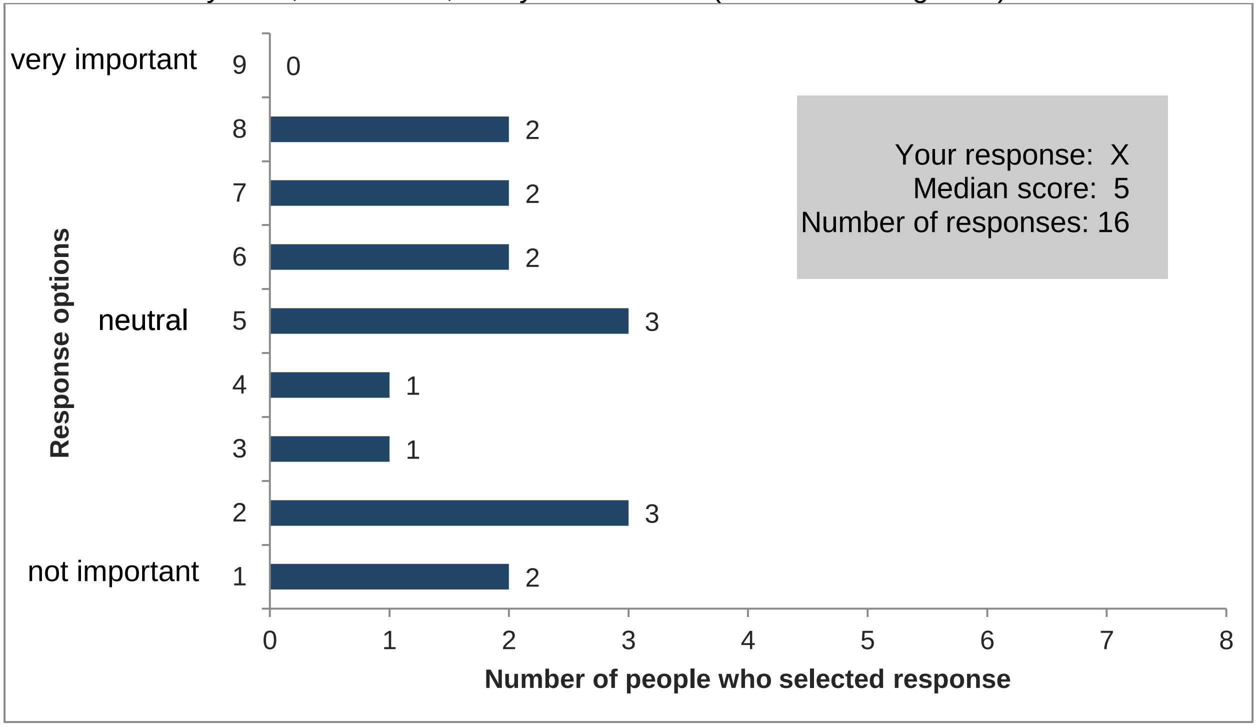

Appendix 6, as supplied by the authors. Appendix to: Thériault G, Limburg H, Klarenbach S, et al.
Recommendations on screening for primary prevention of fragility fractures. CMAJ 2023. doi: 10.1503/cmaj.221219.

Copyright © 2023 The Author(s) or their employer(s). To receive this resource in an accessible format, please contact us at cmajgroup@cmaj.ca.

11. Survey Harm: Screening may result in treatment to prevent fragility fractures when there is little or no evidence that the benefits of treatment would outweigh the harms of treatment (called overtreatment).

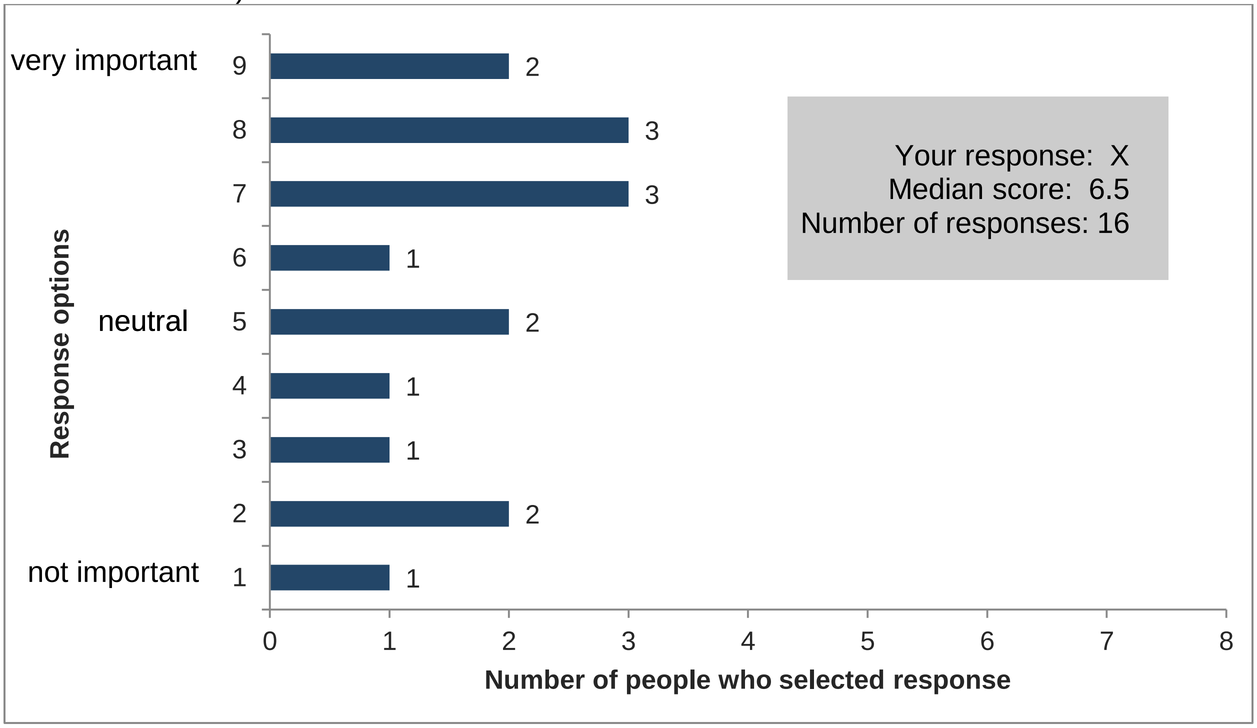

12. Survey Harm: Screening may result in incorrectly identifying someone as being high-risk for fractures, when they are actually not at high-risk for fractures (called a false positive result).

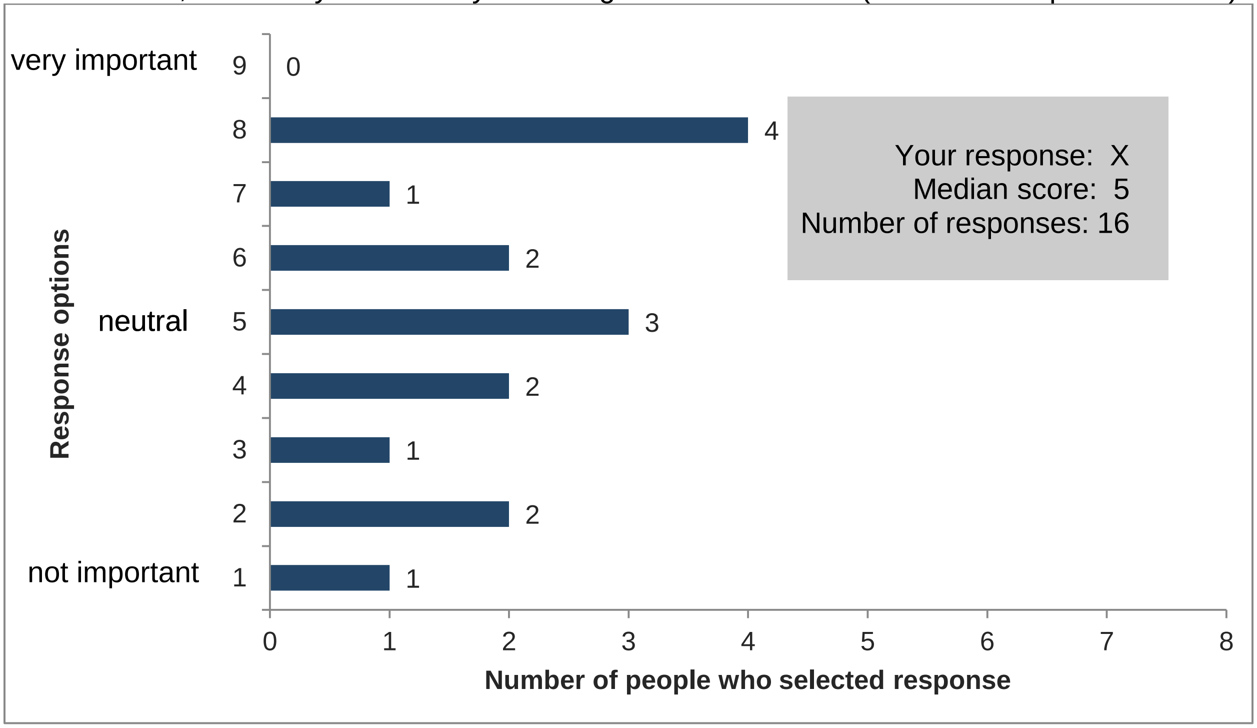

Appendix 6, as supplied by the authors. Appendix to: Thériault G, Limburg H, Klarenbach S, et al. Recommendations on screening for primary prevention of fragility fractures. CMAJ 2023. doi: 10.1503/cmaj.221219.

Copyright © 2023 The Author(s) or their employer(s). To receive this resource in an accessible format, please contact us at cmajgroup@cmaj.ca.

13. Survey Harm: Screening may result in incorrectly identifying someone as being low-risk for fractures, when they are actually at a high-risk for fractures (called a false negative)

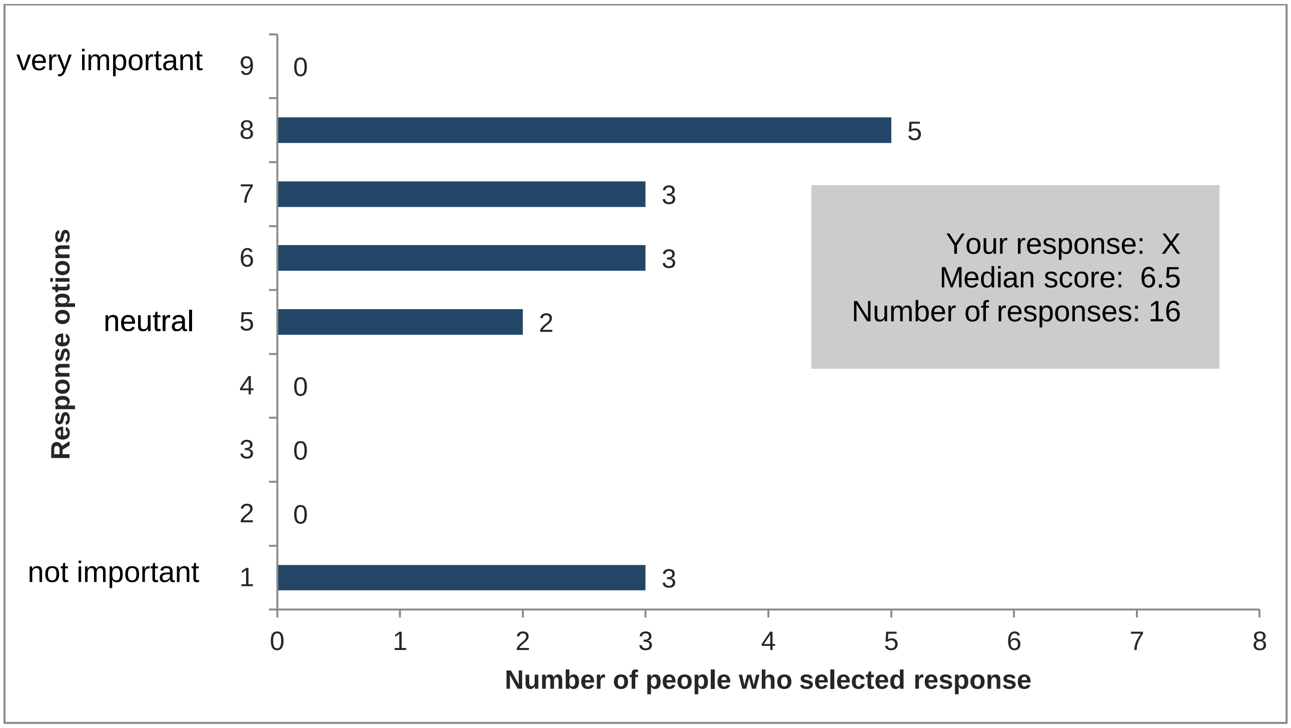

Selection of the Top Five Potential Outcomes for Fragility Fracture Screening in Adults

In the survey, we listed 13 potential outcomes (harms and benefits) of screening for fragility fractures in adults and asked you to select the five items on the list that you think are most critical to consider when people make decisions about whether or not to be screened to determine if they are at high risk for fragility fractures. Here are the outcomes that **you** selected as the top five items that are most important to consider (in no particular order):

- Selected Outcome 1
- Selected Outcome 2
- Selected Outcome 3
- Selected Outcome 4
- Selected Outcome 5

Below is a table that lists of **all** of the statements about outcomes of fragility fracture screening, and the number of participants who selected each option as one of their "top five" items that were most critical to consider. 'Harm' outcomes are highlighted in red, while 'benefit' outcomes are highlighted in green.

Appendix 6, as supplied by the authors. Appendix to: Thériault G, Limburg H, Klarenbach S, et al. Recommendations on screening for primary prevention of fragility fractures. CMAJ 2023. doi: 10.1503/cmaj.221219.
Copyright © 2023 The Author(s) or their employer(s). To receive this resource in an accessible format, please contact us at cmajgroup@cmaj.ca.

Potential Outcome:	# of participants who selected this as a "top five" item to consider
Screening that leads to a decrease in fractures may improve overall well-being and quality of life	**11**
Screening may decrease the number of broken bones caused by a minor fall or normal activity that usually should not cause a fracture in healthy adults	**10**
Screening may decrease the number of hip bone fractures that someone will experience	**9**
Screening that leads to a decrease in fractures may improve your ability to perform every-day activities. This can include decreased admissions to long-term care	**9**
Screening may lead to treatment with medications used to lower the risk of fractures. These medications can have unwanted, but not serious, side effects. This could include nausea, heartburn, abdominal pain, loose bowels, rashes, muscle cramps or non-serious infections	**8**
Screening may decrease the chance of death directly related to a bone fracture	**6**
Screening may lead to treatment with medications used to lower the risk of fractures. In very rare cases, medication can have serious side effects, including death or serious illness	**6**
Screening may decrease the chance of death from causes or conditions that may be indirectly related to, or worsened by, a bone fracture	**4**
Screening may result in treatment to prevent fragility fractures when there is little or no evidence that the benefits of treatment would outweigh the harms of treatment (called overtreatment)	**4**
Screening may result in incorrectly identifying someone as being low-risk for fractures, when they are actually at a high-risk for fractures (called a false negative)	**4**
Screening may result in incorrectly identifying someone as being high-risk for fractures, when they are actually not at high-risk for fractures (called a false positive result)	**3**

Appendix 6, as supplied by the authors. Appendix to: Thériault G, Limburg H, Klarenbach S, et al.
Recommendations on screening for primary prevention of fragility fractures. CMAJ 2023. doi: 10.1503/cmaj.221219.
Copyright © 2023 The Author(s) or their employer(s). To receive this resource in an accessible format, please contact us at cmajgroup@cmaj.ca.

Screening may correctly identify you as being at high risk of a fracture, even though you would never have actually experienced a fracture in your lifetime. This can lead to unnecessary tests, treatments, worry and concern (called over-diagnosis)	**2**
Screening may result in treatment with medications used to lower the risk of fractures. In some cases treatment is stopped because of unwanted side effects of these medications	**2**

Considerations for Screening Scale Ratings

For this question, participants were asked to rate how much they would want to be screened to determine if they are at high risk of fragility fractures.

Participants could rate the phrase "I would want to be screened to determine if I am at high risk for fragility fractures" from 1 - 9: 1 being "Not at all"; 5 being "Neutral"; and 9 being "Very much".

Your answer and the answers given by all participants are presented in the same graph format as the earlier questions.

Summary of Considerations for Screening Scale Ratings

1. **Survey Question:** Considering the potential harms and benefits of screening to determine if someone is at high risk for fragility fractures, how much would you want to be screened?

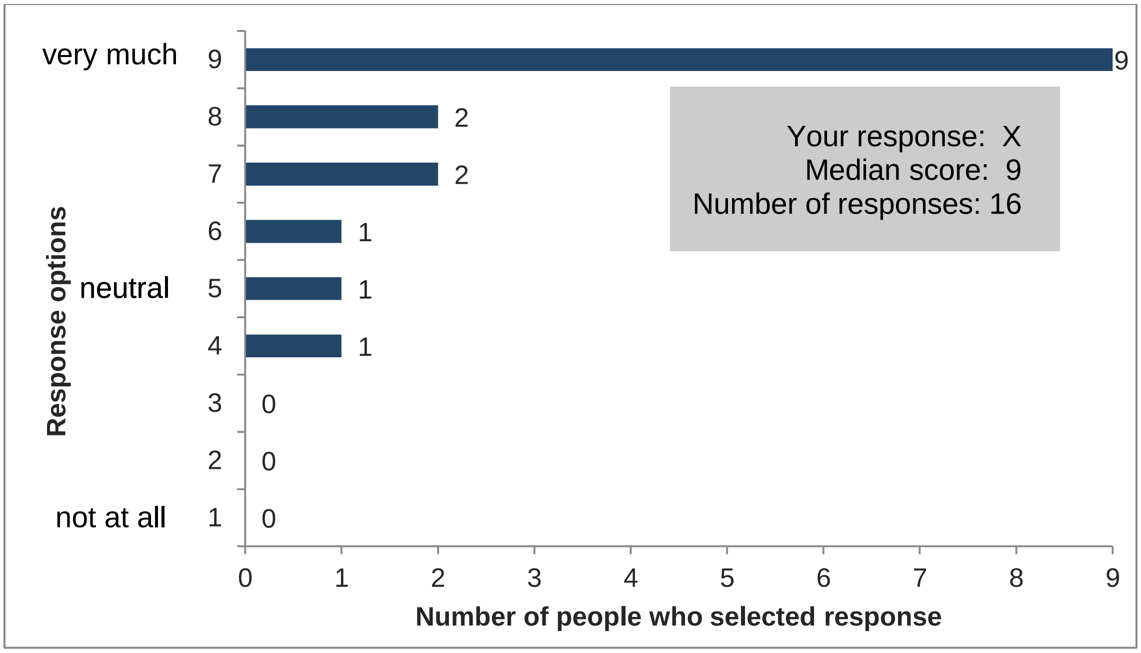

St. Michael's
Inspired Care.
Inspiring Science.

Appendix 6, as supplied by the authors. Appendix to: Thériault G, Limburg H, Klarenbach S, et al.
Recommendations on screening for primary prevention of fragility fractures. CMAJ 2023. doi: 10.1503/cmaj.221219.
Copyright © 2023 The Author(s) or their employer(s). To receive this resource in an accessible format, please contact us at cmajgroup@cmaj.ca.

Appendix E: Focus group guide

Welcome, introductions, and ground rules

Welcome (greet people as they join the teleconference)

Hello everyone and thank you for joining us today for the Canadian Task Force on Preventive Health Care focus group about screening to determine if someone is at high risk for fragility fractures.

My name is _______________ and I am from the Knowledge Translation Program at St. Michael's Hospital in Toronto. I am going to be the focus group moderator today.

We are going to go through some background information, and instructions for the next 5 to 7 minutes. I will mute everyone's line while I'm providing this information, and I will unmute everyone once we get into the discussion.

I have two colleagues joining me today. The first is ___(Rossella)____ who will be our note taker. The second is Dr. Guylene Theriault who is the chair of the Task Force's guideline development working group for screening to prevent fragility fractures. She will be on the line to answer any content related questions you may have.

Background information:

I will now give some background information on the project.

- The Canadian Task Force on Preventive Health Care creates evidence-based guidelines about different types of screening and health interventions. These guidelines are for primary care providers, such as family physicians, and they recommend who to screen and when to screen, as well as who *not* to screen and when *not* to screen.
- Now, the Task Force is developing a guideline on screening to determine if someone is at high risk for fragility fractures.
- The purpose of this conversation that we are having today is to get feedback from members of the public on your opinions about the outcomes of screening. Today, when we say 'outcome', we mean the effects screening to determine if fragility fractures could have on someone's health, either directly or indirectly.
- We are using what is called a Modified Delphi technique, which is a method that repeats the same questions in a survey, a focus group, and a second survey to understand your preferences.
- First, in the survey you've already completed, we provided you with some background information on fragility fractures and then asked you to rate how important the screening outcomes are to you in a survey.
- Today we will discuss the outcomes you rated in the survey. We ask that you please have your participant data summary sheet and background information sheet in front of you for the call, since those are the materials we will be discussing as a group today.
- After the focus group, we will send you another survey and ask you to re-rate the same outcomes to see if you change any of your ratings based on any new information we discuss during today's session.

Appendix 6, as supplied by the authors. Appendix to: Thériault G, Limburg H, Klarenbach S, et al.
Recommendations on screening for primary prevention of fragility fractures. CMAJ 2023. doi: 10.1503/cmaj.221219.
Copyright © 2023 The Author(s) or their employer(s). To receive this resource in an accessible format, please contact us at cmajgroup@cmaj.ca.

- We really encourage you to ask Dr. Theriault any content questions you may have about screening to prevent fragility fractures after reviewing the materials that were sent to you.

Reminders

- Some reminders for the call:
- Please mute yourself when you are not speaking. You can mute yourself using the mute button on your phone.
- If people do not mute themselves and we can hear a lot of background noise, we may mute you from our end. If we do this, a voice will come over your line to tell you that you have been muted. To unmute yourself, you can press **.
- To allow us to capture all the information being discussed today as a group, please try to say your name before you speak and take turns speaking. It is helpful for the transcriptionist when they are converting the audio to text.
- I also want to be clear that there is no need to wait for me to call on you to speak. Feel free to jump in once the other person is done talking. That said, I may call on people if the group is very quiet or if the discussion is going very fast just to make sure everyone has a chance to contribute.
- I want to emphasize that there are no right or wrong answers on today's call. Please feel free to ask any questions at any point during the focus group, and if you want me to repeat any information please let me know.
- Finally, just to re-emphasize, today when we say screening, we mean testing people who do **not** have any signs or symptoms, or, in this case, have **not** already confirmed that they are at high risk for fragility fractures. Screening in this case involves using screening tests to help **determine if** someone is at high risk for fragility fractures. So you would not know if you are at a high risk for fragility fractures or not before screening. Some screening uses only a questionnaire, while some involve an x-ray test (with or without a questionnaire) to help determine the level of risk. Remember that screening programs can do both harm and good. In the survey you completed, we identified 13 potential outcomes: 6 potential benefits and 7 potential harms, of screening to determine if you are at risk for fragility fractures. The purpose of today's call is to discuss which outcomes (which includes both harms and benefits), that you think are **important** for someone to consider when deciding whether or not to be screened to determine if they are at high risk for fragility fractures. Again, this would apply to those who are not showing symptoms, those who are being screened would **not** already know their risk level.

Confidentiality and consent to audio record

- Now I will talk about confidentiality: We take the issue of confidentiality seriously. No personal information about you will be shared with anyone outside of the study team. Your real name will not appear anywhere in the reports from today's session.

Appendix 6, as supplied by the authors. Appendix to: Thériault G, Limburg H, Klarenbach S, et al.
Recommendations on screening for primary prevention of fragility fractures. CMAJ 2023. doi: 10.1503/cmaj.221219.
Copyright © 2023 The Author(s) or their employer(s). To receive this resource in an accessible format, please contact us at cmajgroup@cmaj.ca.

- Any other information from today that could identify who you are will also be changed. So for example, if you say “in Toronto, where I live” we will replace that with something like “in the place where the participant lives”.

- We strongly urge you to respect each other’s privacy and not discuss what is said in the focus group with others. Also, please do not share the study materials with anyone outside of the study. The documents shared with you are not publicly available yet. Once the guideline recommendations are finalized they will be emailed to you and posted to the Task Force website.
- To respect everyone’s privacy; we want to give you the option of using either your first name OR your participant ID number for the recording. I will call on each of you to state whether you would prefer to be called by your participant ID number or first name.
- Please also state that you consent to participate in today’s recorded discussion. For example, “This is [name/participant number], I consent to participate”. Let’s begin with:

Participant Name	Email	Phone Number	Notes

- Have I missed anyone? Thank you.

- We are now ready to begin. I have unmuted everyone and we will begin audio recording. If anyone is opposed to audio recording today’s session please let me know now.

  [Turn recorder on]

- The audio recorder is now on. Today’s date is ______________, and I am conducting the Task Force focus group for screening to prevent fragility fractures. There are ____ participants present on the call today.

Appendix 6, as supplied by the authors. Appendix to: Thériault G, Limburg H, Klarenbach S, et al.
Recommendations on screening for primary prevention of fragility fractures. CMAJ 2023. doi: 10.1503/cmaj.221219.
Copyright © 2023 The Author(s) or their employer(s). To receive this resource in an accessible format, please contact us at cmajgroup@cmaj.ca.

We will begin with some questions related to the fragility fractures background information sheet:

1) Fragility Fractures background sheet:

1) While reviewing this document, did you have any questions or general thoughts about the document?

2) How easy was the information to understand?
    a. What in particular made the document easy to understand?

3) Do you believe additional information should be included in this background information sheet?

4) When having a discussion with your family physician about screening to determine if you are at high risk for fragility fractures, what types of information would you like him/her to bring up?

    a. How much information do you feel you need before you can make a decision about screening to prevent fragility fractures?

2) Overall preference before discussion:

Just as a reminder, from the background information sheet: Screening for fragility fractures involves using screening tests to help determine if someone is at high risk for fragility fractures. So those being screened would not already know their risk level. Some screening uses only a questionnaire, while some involve an x-ray test (with or without a questionnaire) to check the level of risk.

Please turn to *page 11.* The question reads ‘*Considering the potential harms and benefits of screening to determine if someone is at high risk for fragility fractures, how much would you want to be screened?’* Responses ranged from *4-9* with a median, or middle, of 9.

a. Are there any questions about **screening** for our content expert?
b. Take a look at how you rated this question. What was your rationale for rating the question the way you did?

1. After reviewing the background document and completing the pre-focus group survey, what is your overall preference for screening to determine if you are at high risk for fragility fractures? That is, if given the opportunity, would you choose to be screened or not?

3) Pre-focus group survey results – fragility fracture screening harms and benefits:

Appendix 6, as supplied by the authors. Appendix to: Thériault G, Limburg H, Klarenbach S, et al.
Recommendations on screening for primary prevention of fragility fractures. CMAJ 2023. doi: 10.1503/cmaj.221219.
Copyright © 2023 The Author(s) or their employer(s). To receive this resource in an accessible format, please contact us at cmajgroup@cmaj.ca.

We are now going to review the pre-focus group survey results. Our discussion will focus on the potential harms and benefits that were rated differently (largest range in responses) across the group. Just a reminder again that as part of the survey and focus group process, we are looking for feedback on how important each harm and benefit is for you when making a decision on whether or not to be screened to determine if you are at a high-risk for fragility fractures. Remember that there are no right or wrong answers. Please have your personalized data summary sheet in front of you so that you can review during the conversation.

Note: facilitator will discreetly call upon participants who responded differently from the group and probe why.

14. **Potential Screening Benefit:** Screening may decrease the chance of death directly related to a bone fracture

    2. Please turn to page 4 and refer to question 3 located at *top* of the page. The outcome reads '**Survey Benefit***: Screening may decrease the chance of death directly related to a bone fracture.* Responses ranged from *1-9* with a median of 8.
        - **a.** Are there any questions about this *benefit* for our content expert?
        - **b.** Take a look at how you rated this question. What was your rationale for rating the question the way you did, or why did you feel rate this outcome as being important or unimportant in your decision on whether or not to be screened?

15. **Potential Screening Benefit:** Screening may decrease the chance of death from causes or conditions that may be indirectly related to, or worsened by, a bone fracture

    3. Please turn to page 4 and refer to question 4 located at *the bottom* of the page. The outcome reads '**Survey Benefit: '***Screening may decrease the chance of death from causes or conditions that may be indirectly related to, or worsened by, a bone fracture.'* Responses ranged from 1-9 with a median of 8.5.
        - **a.** Are there any questions about this *benefit* for our content expert?
        - **b.** Take a look at how you rated this question. What was your rationale for rating the question the way you did, or why did you feel rate this outcome as being important or unimportant in your decision on whether or not to be screened?

16. **Potential Screening Harm:** Screening may lead to treatment with medications used to lower the risk of fractures. These medications can have unwanted, but not serious, side effects. This could include nausea, heartburn, abdominal pain, loose bowels, rashes, muscle cramps or non-serious infections

Please turn to page 6 and refer to question 7 located at *top* of the page. The outcome reads '**Survey Harm***: Screening may lead to treatment with medications used to lower the risk of fractures. These medications can have unwanted, but not serious, side effects. This could include nausea, heartburn, abdominal pain, loose bowels, rashes, muscle cramps or non-serious infections*

4. Responses ranged from *1-8* with a median of 4.5.
    - **a.** Are there any questions about this *benefit* for our content expert?

St. Michael's
Inspired Care.
Inspiring Science.

Appendix 6, as supplied by the authors. Appendix to: Thériault G, Limburg H, Klarenbach S, et al.
Recommendations on screening for primary prevention of fragility fractures. CMAJ 2023. doi: 10.1503/cmaj.221219.
Copyright © 2023 The Author(s) or their employer(s). To receive this resource in an accessible format, please contact us at cmajgroup@cmaj.ca.

b. Take a look at how you rated this question. What was your rationale for rating the question the way you did?

17. **Potential Screening Harm:** Screening may result in treatment with medications used to lower the risk of fractures. In some cases treatment is stopped because of unwanted side effects of these medications

5. Please turn to page 6 and refer to question 8 located at the *bottom* of the page. The outcome reads "**Survey Harm*:*** *Screening may result in treatment with medications used to lower the risk of fractures. In some cases treatment is stopped because of unwanted side effects of these medications'* Responses ranged from 1-9 with a median of 5.
   - **a.** Are there any questions about this *harm* for our content expert?
   - **b.** Take a look at how you rated the outcome of stopping treatment as a result of side effects. What was your rationale for rating the outcome of discontinuing or stopping treatment as unimportant or important for your decision-making for whether or not to be screened?

18. **Potential Screening Harm:** Screening may lead to treatment with medications used to lower the risk of fractures. In very rare cases, medication can have serious side effects, including death or serious illness.

6. Please turn to page 7 and refer to question 9 located at *top* of the page. The outcome reads "**Survey Harm:** *Screening may lead to treatment with medications used to lower the risk of fractures. In very rare cases, medication can have serious side effects, including death or serious illness.* Responses ranged from 2 to 9 with a median of 6.5.
   - **a.** Are there any questions about this *harm* for our content expert?
   - **b.** Take a look at how you rated this question. What was your rationale for rating the importance of this outcome in your screening decision?
     - **i.** Did anyone rate differently than group (for example, about half of people rated it as important but you rated it as critical or not important)?

19. Potential Screening Harm: Screening may correctly identify you as being at high risk of a fracture, even though you would never have actually experienced a fracture in your lifetime. This can lead to unnecessary tests, treatments, worry and concern (called over-diagnosis)

7. Please turn to page 7 and refer to question 10 located at *bottom* of the page. The outcome reads "**Survey Harm:** *Screening may lead to treatment with medications used to lower the risk of fractures. In very rare cases, medication can have serious side effects, including death or serious illness.* Responses ranged from 1 to 8 with a median of 5.
   - **c.** Are there any questions about this *harm* for our content expert?
   - **d.** Take a look at how you rated this question. What was your rationale for rating the importance of this outcome in your screening decision?
     - **i.** Did anyone rate differently than group (for example, about half of people rated it as important but you rated it as critical or not important)?

Appendix 6, as supplied by the authors. Appendix to: Thériault G, Limburg H, Klarenbach S, et al.
Recommendations on screening for primary prevention of fragility fractures. CMAJ 2023. doi: 10.1503/cmaj.221219.
Copyright © 2023 The Author(s) or their employer(s). To receive this resource in an accessible format, please contact us at cmajgroup@cmaj.ca.

20. **Potential Screening Harm:** Screening may result in incorrectly identifying someone as being high-risk for fractures, when they are actually not at high-risk for fractures (called a false positive result)

8. Please turn to page 8 and refer to question 12 located at *bottom* of the page. The outcome reads '**Survey Harm***: Screening may result in incorrectly identifying someone as being high-risk for fractures, when they are actually not at high-risk for fractures (called a false positive result)."* Responses ranged from 1 to 8 with a median of 5.
   - **a.** Are there any questions about this *harm* for our content expert?
   - **b.** Take a look at how you rated this question. What was your rationale for rating the question the way you did? What was your rationale for rating the importance of this outcome in your screening decision?
      - i. Did anyone rate differently than group (for example, about half of people rated it as important but you rated it as critical or not important)?

21. **Potential Screening Harm:** Screening may result in incorrectly identifying someone as being low-risk for fractures, when they are actually at a high-risk for fractures (called a false negative)

9. Please turn to page 9 and refer to question 13 located at *the top* of the page. The outcome reads '**Survey Harm:** *Screening may result in incorrectly identifying someone as being low-risk for fractures, when they are actually at a high-risk for fractures (called a false negative)."* Responses ranged from 1 to 8 with a median of 6.5.
   - **a.** Are there any questions about this *harm* for our content expert?
   - **b.** Take a look at how you rated this question. What was your rationale for rating the question the way you did? What was your rationale for rating the importance of this outcome in your screening decision?

Selection of the Top 5 Potential Harms and Benefits for Screening to prevent fragility fractures

10. Please turn to page 10 and refer to the list of 13 potential outcomes (or harms and benefits) of screening to prevent fragility fractures. We asked you to select five items on the list that you think were most critical to consider when people are making decisions about screening. Benefit outcomes are highlighted in green, and harm outcomes are highlighted in red in the table. The second column indicates the number of participants who selected each option as one of their 'Top Five' items that were most critical to consider when making a screening decision.
   - a. Take a look at <u>your</u> selected top five outcomes, which are listed at the bottom of page 9. What was your rationale for selecting these outcomes? (How did you decide which outcomes were most important for you to consider, how did you decide which outcomes to include in your top five?)
   - b. Survey harms were also selected less frequently by participants as among their top 5 outcomes to consider. Do you have any thoughts about this?

<u>4) Overall preference after discussion:</u>

Appendix 6, as supplied by the authors. Appendix to: Thériault G, Limburg H, Klarenbach S, et al.
Recommendations on screening for primary prevention of fragility fractures. CMAJ 2023. doi: 10.1503/cmaj.221219.
Copyright © 2023 The Author(s) or their employer(s). To receive this resource in an accessible format, please contact us at cmajgroup@cmaj.ca.

2. **Survey Question:** Considering the potential harms and benefits of screening to determine if someone is at high risk for fragility fractures, how much would you want to be screened?

11. Please turn to *page 10.* The question reads '*Considering the potential harms and benefits of screening to determine if someone is at high risk for fragility fractures, how much would you want to be screened?'* Responses ranged from *4-9* with a median of 9.
    - **c.** Are there any questions about screening for our content expert?
    - **d.** Take a look at how you rated this question. What was your rationale for rating the question the way you did?
        - **a.** What harm or benefit is the most important for you when making this decision?
        - **b.** What harm or benefit is the least important for you when making this decision
    - **e.** Have your preferences changed from those you expressed in the first survey and earlier in today's discussion?

4) Additional Information:

12. Reflecting on today's discussion is there any other information you would like to know that would help you to make a decision if you had the opportunity to decide to be screened or not to determine if you are at high risk for fragility fractures?

5) Potential barriers or facilitators to screening:

13. Screening for fragility fractures involves using screening tests to help determine if someone is at high risk for fragility fractures. Some screening uses only a questionnaire, while some involve an x-ray test (with or without a questionnaire) to check the level of risk. The x-ray test measures bone mineral density or bone thickness. Measurements are usually taken at the hip and spine, and sometimes at the forearm. A finding of low bone mineral density may help predict a higher risk of fragility fractures.

    - **a.** If you choose to get screened, what are potential barriers to accessing the screening test, if any? – What would make accessing screening hard? What would make it easy?
        - **i.** Probe: out-of-pocket expenses (e.g., transportation or taking time off)
        - **ii.** Probe: lack of time (e.g., having to miss work to be screened etc.)
        - **iii.** Probe: fear (e.g., potential lifestyle changes)

    - **b.** If you choose to get screened, what would make getting the screening test easy, if anything?

Appendix 6, as supplied by the authors. Appendix to: Thériault G, Limburg H, Klarenbach S, et al.
Recommendations on screening for primary prevention of fragility fractures. CMAJ 2023. doi: 10.1503/cmaj.221219.
Copyright © 2023 The Author(s) or their employer(s). To receive this resource in an accessible format, please contact us at cmajgroup@cmaj.ca.

6) Closing remarks:

Does anyone have any final comments or questions before we end today's discussion?

Conclusion

- Thank you for taking the time to be a part of our focus group today.
- This week you will each receive a link to another online survey via email. This is the same survey you completed prior to today's discussion but with some extra questions about your experience participating in the project. The reason that the survey asks the same questions is so that you have an opportunity to change or confirm your responses from the first time you completed the survey. For example, a person may have developed new understanding or a new perspective after discussing the outcomes in greater detail during today's discussion and wants to change their rating of that outcome. Another person may feel surer about their responses and keep the ratings the same. We like to see the differences and the similarities in people's ratings before and after the teleconference discussion.
- You have approximately one week to complete the online survey.
- We will process your reimbursement payment once we close the survey. Please note that the reimbursement payment can take up to 45 days to process, but it usually doesn't take that long.
- Once we develop a report of our findings we will create a summary to send to you. You will also be invited to attend an optional debrief session to review the results of the study and add additional comments.
- We understand that questions or additional comments may come up after today's call. This is very normal. If you have any additional questions or something that you would like to add to today's discussion, please feel free to email Rossella. We will do our best to answer your question. If we are not able to answer your question we will forward it to the working group content expert for their opinion.
- Thank you and have a great day.

Appendix 6, as supplied by the authors. Appendix to: Thériault G, Limburg H, Klarenbach S, et al.
Recommendations on screening for primary prevention of fragility fractures. CMAJ 2023. doi: 10.1503/cmaj.221219.
Copyright © 2023 The Author(s) or their employer(s). To receive this resource in an accessible format, please contact us at cmajgroup@cmaj.ca.

Appendix F: Patient engagement survey

Please respond to each of the following statements using the scales provided.

Respond to each question 1-7: 1: No extent, 2: Very small extent, 3: Small extent, 4: Fair extent, 5: Moderate extent, 6: Large extent, 7: Very large extent. If you select 1-4 for any question, please explain your rating in the space below the question.

- To what extent do you believe that your ideas were heard during the engagement process?
- To what extent did you feel comfortable contributing your ideas to the engagement process?
- Did organizers take your contributions to the engagement process seriously?
- To what extent do you believe that your input will influence final decisions that underlie the engagement process?
- To what extent do you believe that your values and preferences will be included in the final health advice from this process?
- To what extent were you able to clearly express your viewpoints?
- How neutral in their opinions (regarding topics) were organizers during the engagement process?
- Did all participants have equal opportunity to participate in discussions?
- How clearly did you understand your role in the process?
- To what extent was information made available to you either prior or during the engagement process so as to participate knowledgeably in the process?
- To what extent were the ideas contained in the information material easy to understand?
- How clearly did you understand what was expected of you during the engagement process?
- How clearly did you understand what the goals of the engagement process were?
- To what extent would you follow health advice from the Canadian Task Force on Preventive Health Care (if it related to your health condition)?
- To what extent would you advise others to follow health advice from the Canadian Task Force on Preventive Health Care (if it related to their health condition)?

Appendix 6, as supplied by the authors. Appendix to: Thériault G, Limburg H, Klarenbach S, et al.
Recommendations on screening for primary prevention of fragility fractures. CMAJ 2023. doi: 10.1503/cmaj.221219.
Copyright © 2023 The Author(s) or their employer(s). To receive this resource in an accessible format, please contact us at cmajgroup@cmaj.ca.
