# Supplementary material for: Recommendations on screening for primary prevention of fragility fractures
Source: CMAJ. 2023 May 8;195(18):E639–49. doi: 10.1503/cmaj.221219 (PMC10166624; doi:10.1503/cmaj.221219)
Supplement: Appendix 7: Fragility Fractures Community Jury: Data Summary Report [file 221219-guide-7-at.pdf]

Canadian Task Force on Preventive Health Care

Fragility Fractures Community Jury: Data Summary Report

Prepared for the Canadian Task Force on Preventive Health Care

Submitted: March 16, 2022

Prepared By:
Harleen Buttar

Knowledge Translation Program
Li Ka Shing Knowledge Institute
St. Michael's Hospital, Unity Health Toronto

Contact:
Harleen Buttar
E: Harleen.Buttar@unityhealth.to
T: 416-864-6060 ext. 77566

St. Michael's
Inspired Care.
Inspiring Science.

UNITY HEALTH TORONTO

Table of Contents

Executive Summary 2

Background and Rationale 2

Introduction 3

The Community Jury Approach 3

Methods 4

Participants 4

- Recruitment 4
- Characteristics of Included Participants 4

Results 5

- Phase 2 – Key Message Statement Activity 5
- Phase 2 – Decision Aid Perceptions and Feedback 9

Participant Engagement and Experience 12

Suggestions for Applying Findings 12

Conclusion 12

References 13

Appendix A: Post-Community Jury Evaluation Survey 15

Appendix B: Deliberation Guide 48

Appendix 7, as supplied by the authors. Appendix to: Thériault G, Limburg H, Klarenbach S, et al. Recommendations on screening for primary prevention of fragility fractures. *CMAJ* 2023. doi: 10.1503/cmaj.221219.

Copyright © 2023 The Author(s) or their employer(s). To receive this resource in an accessible format, please contact us at cmajgroup@cmaj.ca.

Executive Summary

Incorporating patient priorities and perspectives into clinical practice guideline (CPG) development is an important part of patient-centered care. The Canadian Task Force for Preventive Health Care (Task Force) continues to engage the support of the Knowledge Translation (KT) team at St. Michael's Hospital to explore ways to meaningfully involve patients and members of the public in its guideline development process. Considering the Task Force's prioritization on shared decision making regarding preventive health care interventions, it is crucial to explore opportunities to meaningfully engage members of the public and to elicit their values and preferences for such interventions to inform guideline development.

The KT team recruited the TF-PAN network, which was composed of a group (n = 15-20) patients, caregivers, and members of the public (defined in this context as individuals who are not in clinical practice or former primary care professionals). Initial recruitment occurred from September 11th to October 11th, 2020.

A community jury was used to elicit TF-PAN members' perceptions on the domains of equity, feasibility, and cost in addition to the balance of benefits and harms. These judgements will be used to inform the direction and strength of Task Force recommendations as well as inform nuanced considerations around patient preferences that can be written into the guideline and KT Tools. The session had two phases: Educating Participants (Phase 1) and Deliberation (Phase 2).

Overall, participants felt that the key messages should be written so that they are shorter, simpler, and free of jargon. Participants recommended using key message statements that are easy for the public to read and comprehend. Furthermore, participants felt that the Bone Health Choice Decision Aid was a useful tool to assess fragility fracture risk. However, concerns around accessibility and useability were reoccurring themes. Participants felt only fairly convinced that their values and preferences would be included in the final advice.

Background and Rationale

Involving patients in the development of clinical practice guidelines (CPGs) can yield recommendations that are more likely to be patient-centered, practical, and can provide opportunities for shared decision-making.[1-6] For guideline developers, patient involvement may enhance the credibility, transparency, and applicability of CPGs. Numerous international organizations that appraise the quality of CPGs explicitly call for patient involvement in the guideline development process.[3-5] Similarly, findings from a literature review conducted by The National Institute for Health and Care Excellence[6] suggested that involving patients early in the guideline development process and asking how they would like to be engaged can improve the effectiveness of participants' contributions, as well as influence guideline scope and improve inclusion of patient-relevant topics.[6-12] Despite the benefits of involving patients in the guideline development process, adequate training and support are commonly cited as barriers to patient involvement.[6-12]

Appendix 7, as supplied by the authors. Appendix to: Thériault G, Limburg H, Klarenbach S, et al. Recommendations on screening for primary prevention of fragility fractures. *CMAJ* 2023. doi: 10.1503/cmaj.221219. Copyright © 2023 The Author(s) or their employer(s). To receive this resource in an accessible format, please contact us at cmajgroup@cmaj.ca.

The Canadian Task Force on Preventive Health Care's (Task Force) 'Task Force Public Advisors Network' (TF-PAN) is an initiative to encourage early and meaningful engagement of members of the public with the Task Force by seeking their input throughout the development and dissemination of Task Force guidelines. In contrast to the traditional Task Force patient preferences model, TF-PAN members were provided more in-depth background information on what the Task Force does, and the types of methods/processes used to develop preventive health care guidelines to ensure informed participation in guideline development. TF-PAN activities are facilitated by the St. Michael's Hospital Knowledge Translation (KT) Program in partnership with the TF. TF-PAN members formed a stakeholder consultation group and provided input in the early phase of fragility fracture guideline development, as determined by the guideline Working Group chair.

Introduction

Incorporating patient priorities and perspectives into clinical practice guideline (CPG) development is an important part of patient-centered care.

TF-PAN members are provided specific opportunities to contribute to project directions in the guideline development process. Network members may choose to take part in particular activities based on their interest and availability. This means TF-PAN can have different perspectives at different points in the process, and participants can choose how they want to engage. At minimum, TF-PAN looks to engage members in three ways:

1. Participate in welcome orientation session to meet Task Force leadership, KT team members, and each other.
2. Participate in the training sessions on the Task Force, preventive health care concepts, and patient engagement (live sessions that are recorded and posted online for TF-PAN members to access if they are unable to attend).
3. Participate in at least two Community Jury sessions per year to provide guideline specific input on public or patient preferences (note: this is the new approach to the 'Patient Preferences' methodology)

The Community Jury Approach

A community jury (modified terminology regarding a citizen's jury) approach was used to engage TF-PAN members to provide input on the upcoming fragility fractures guideline. A community jury is a form of deliberative democracy and aims to elicit an *informed* community perspective on important and potentially controversial topics. Community jury participants are provided with expert presentations and opportunities to question the experts, engage in both facilitated and private deliberation, and are asked to form a consensus or majority 'verdict' on the topic question[13].

Community juries elicit the voices, values, and preferences of informed citizens who are presented with as systematically synthesized evidence. The sessions are structured to be a two-way dialogue with a 'topic' on trial. 'Jurors' then get a chance to deliberate the evidence before formulating opinions and recommendations. The structure of a session has two phases:

St. Michael's Inspired Care. Inspiring Science. UNITY HEALTH TORONTO

Appendix 7, as supplied by the authors. Appendix to: Thériault G, Limburg H, Klarenbach S, et al. Recommendations on screening for primary prevention of fragility fractures. *CMAJ* 2023. doi: 10.1503/cmaj.221219. Copyright © 2023 The Author(s) or their employer(s). To receive this resource in an accessible format, please contact us at cmajgroup@cmaj.ca.

1. Educating Participants
2. Deliberation

The two phases were conducted over a two-week period. Phase 1, Educating Participants, was 2 hours long. Phase 2, Deliberation, was approximately 2 hours long, and was held one week after Phase 1. This allowed participants to reflect on information presented in Phase 1 and to formulate thoughtful, informed opinions or questions to bring to Phase 2.

This document presents summary data from Phase 2 of the Task Force's community jury about screening for fragility fractures. Data was collected between November 1st and December 31st, 2021.

This is the first community jury facilitated after transitioning from the previous Task Force patient preferences engagement approach.

Methods

Participants

Recruitment

The participants were recruited from the TF-PAN network, a group composed of (n = 15-20) patients, caregivers, and members of the public (defined in this context as individuals who are not in clinical practice or former primary care professionals). Participants have a membership term of one year with the potential for renewal. Initial recruitment into the network occurred from September 11th to October 11th, 2020.

Interested network participants completed a TF-PAN application and demographic survey. The survey was available in English and in French and assessed demographic factors (gender, age, geographic location, region, race/ethnicity, education level) and why participants were interested in joining TF-PAN. Participants were asked to disclose any potential conflicts of interest. When selecting TF-PAN members, the KT Team aimed to select a diverse panel, specifically considering the intersection of applicant gender, language (English and French), race-ethnicity, education, geographic location, and age. To ensure feasibility, panel selection considered a demonstrated interest and motivation to participate and capacity to commit to the time requirements for TF-PAN training and activities.

Participants were compensated $50 for participating in the project as per the SMH KT Program internal reimbursement policy.

From the network of 20 people, we identified 10 people who expressed interest in participating in a community jury on fragility fractures.

Characteristics of Included Participants

Ten participants were invited to participate in the community jury. The final sample consisted of 6 participants who were 18 to 45 years of age. 3 participants identified as male, and 3 identified as female. One participant self-identified as Indigenous (i.e., First Nations, Métis, or Inuit).

Knowledge Translation Program
St. Michael's Inspired Care. Inspiring Science.
UNITY HEALTH TORONTO
Appendix 7, as supplied by the authors. Appendix to: Thériault G, Limburg H, Klarenbach S, et al. Recommendations on screening for primary prevention of fragility fractures. *CMAJ* 2023. doi: 10.1503/cmaj.221219. Copyright © 2023 The Author(s) or their employer(s). To receive this resource in an accessible format, please contact us at cmajgroup@cmaj.ca.

Participants were from Ontario (*n* =3), Manitoba (*n* = 1), British Columbia (*n* = 1), and New Brunswick (*n* = 1). All participants lived in urban or suburban areas (*n* = 2; *n* = 4). All participants had either a high school diploma (n=1), college diploma or bachelor's degree (*n* = 4) or a graduate or professional degree (*n* = 1).

Community Jury

In this project, a community jury approach was used to engage TF-PAN members to:

1. Co-develop key message statements for the fragility fractures guideline
2. Provide feedback on the Bone Health Choice Decision Aid tool developed by the Mayo Clinic (https://osteoporosisdecisionaid.mayoclinic.org/) in the United States that helps primary care providers and the public understand who is at risk of developing a bone fracture and who needs to undergo subsequent screening procedures for fragility fractures. The Task Force is developing a similar tool assess fragility fracture risk in a Canadian context.

The community jury judgements will be used to inform the direction and strength of Task Force recommendations as well as inform nuanced considerations around patient preferences that can be written into the guideline and KT Tools.

In Phase 1, the KT team and the Guideline Working Group collaborated to develop questions or goals for the community jury to guide the session. These questions or goals were determined by the Guideline Working Group. The KT team and Task Force Guideline Working Group Chair co-developed a presentation that described the intervention being evaluated and how the condition would be addressed without the intervention along with relevant outcomes, harms, and benefits as identified in the evidence.

In Phase 2, participants co-developed key message statements and provided feedback on the Bone Health Decision Aid Tool. For a detailed description of the methods used in this project, please refer to Phase 2 of the Task Force's Method's document.

Participants' perceptions of their experience with the community jury were examined using an engagement evaluation tool in Phase 2.

Results

Below is a summary of participants' perceptions and feedback of the key message statements provided by the Working Group.

Phase 2 – Key Message Statement Activity

In the first part of the activity, participants were presented with the key message statements that they were introduced in Phase 1 of the community jury. Participants were asked to provide feedback, insight and create suggestions on key messages that would help the public understand guideline recommendations and their rationale.

Table 1. Summary of key messages, feedback, and illustrative quotes from activity 1 of the community jury.

Appendix 7, as supplied by the authors. Appendix to: Thériault G, Limburg H, Klarenbach S, et al. Recommendations on screening for primary prevention of fragility fractures. *CMAJ* 2023. doi: 10.1503/cmaj.221219. Copyright © 2023 The Author(s) or their employer(s). To receive this resource in an accessible format, please contact us at cmajgroup@cmaj.ca.

Key Message Statement	Summary of Feedback	Illustrative Quotes
**1.Females ≥ 65 years have the highest rate of fragility fractures and may reduce their risk through appropriate screening and preventive treatment**	• Consider adding a second line/bottom disclaimer for the male population.<br>• Consider adding a disclaimer for the population the key message statement doesn't apply to.<br>• The term "fragility fracture" is jargon. The general population may be unaware of this term.<br>• They key message focuses on the negative aspect first. For example, "Females have the highest rate of fragility fractures." The order of the message should be reversed. Call to action should be presented first, and evidence should be presented later.<br>• The message statement does not clearly identify next steps.<br>• Consider changing the wording to make the message more positive and uplifting.<br>• The wording can be simplified and accompanied by next steps.<br>• Using the term "highest rate" makes it sound as if there are other groups present with a high rate of fragility fractures. Who are these groups?	"The key thing here is, where is the message going? If the audience is the medical practitioners, then this would be suitable for them. But if this was on a poster, or something you would see on social media, I would definitely change this. Most people don't know what a fragility fracture is, so you are already using jargon. If you are trying to reach a large population of people, they are not going to know what a fragility fracture is."<br><br>"What is the main thing you want people to do? Do you want them to go talk to their doctor? Or do you just want them to read a message. I'm not quite sure because this just seems like a nice fact."<br><br>"When I first read *highest rate*, I didn't love it. The first thing I thought was, are there other groups who also have high rate but aren't the highest rate."

	• The phrase, "females have the highest rate of fragility fractures" can be overwhelming for some people. Instead, the key message can be reworded to state that screening and preventive treatment can lower the risk of fragility fractures.	
**2. The Task Force recommends females ≥ 65 years follow a two-step screening process:**<br><br>**<u>Step 1:</u> Answer simple questions so that your provider can calculate your risk of fragility fracture in order to inform you about the possible benefits and harms of treatments. This process is called shared decision making.**<br><br>**<u>Step 2:</u> If preventative treatment is considered undergo an Xray called a bone mineral density test. The result will be used to recalculate your risk to help you take the final decision about preventive treatment.**	• The key message is very overwhelming at first glance.<br><br>• There is too much information and too many steps.<br><br>• "Fragility Fracture" and "shared-decision making" are jargon terms and the public may not be aware of these terms<br><br>• The text should be much shorter and simpler.<br><br>• The first sentence in step 2, sounds awkward and does not fit with the rest of the sentence.<br><br>• The wording excludes a lot of the population. The message should be reworded so it includes are larger and more diverse population.<br><br>• People may not know who and what the Task Force is.<br><br>• The phrase, "The Task Force recommends" can be excluded, and perhaps a little blurb can be written at the bottom to convey that it is the	"When I see this, I am really overwhelmed looking at it. It's just so much information and I don't think your intention is to overwhelm people to the point where they think it's too complicated. … Again, you have the jargon of fragility fracture and now you are introducing the term shared decision making. I am aware of the term shared decision making but I am not sure if the general population is…I find this too much information and I would simplify this. What is the main thing you want people to do?"

Appendix 7, as supplied by the authors. Appendix to: Thériault G, Limburg H, Klarenbach S, et al. Recommendations on screening for primary prevention of fragility fractures. *CMAJ* 2023. doi: 10.1503/cmaj.221219. Copyright © 2023 The Author(s) or their employer(s). To receive this resource in an accessible format, please contact us at cmajgroup@cmaj.ca.

	Task Force who recommends this	
**3. There was no conclusive evidence showing a benefit of screening for males ≥40 and females 40-64 years. Given the absence of proven benefits, the potential side effects of treatment and the societal cost, screening is not recommended for these groups.**	• This message is not appropriate for the general population.<br><br>• The message is very complex and is similar to something published in an academic journal article.<br><br>• Due to the age ranges in the statement, perception of this message can vary.<br><br>• You are telling people, “You don’t need to do this.” This is not very appropriate.<br><br>• The focus should be on people who need to be screened	“I don’t know how helpful it is to tell people, you don’t need to do this. I am wondering if the focus should be on the people that do need to do this.”
**4. In Females ≥65 years we recommend screening with the Canadian FRAX risk assessment tool (without BMD)**<br><br>**Using the 10-year absolute risk of Major Osteoporotic Fracture to facilitate shared decision-making about the possible benefits and harms of treatment**<br><br>**If the patient is considering preventive treatment, we recommend refining the estimated fracture risk by adding the**	• The public would not know what a FRAX or risk assessment is.<br><br>• The message should be made simpler and to the point.<br><br>• The message should also be inclusive of men.	“If this is going to go the general population, then it needs to be really simplified. The clinicians would understand all of this. But in the general population, no one would know what BMD, osteoporotic fractures, or FRAX risk assessment tool is.”

Appendix 7, as supplied by the authors. Appendix to: Thériault G, Limburg H, Klarenbach S, et al. Recommendations on screening for primary prevention of fragility fractures. *CMAJ* 2023. doi: 10.1503/cmaj.221219. Copyright © 2023 The Author(s) or their employer(s). To receive this resource in an accessible format, please contact us at cmajgroup@cmaj.ca.

**BMD value in FRAX.**		

Overall Themes
In general, participants felt that the key messages should be written so that they are shorter, simpler, and free of jargon. Participants recommended using key message statements that are easy for the public to read and comprehend. Densely-worded text and complex sentences may be appropriate for healthcare practitioners but were perceived as too overwhelming for the public. The complexity may inhibit the public's understanding of guideline recommendations and their rationale. The target population and dissemination platform are important to consider when developing the messages. Participants feel that the key message statements excluded important populations such as men. Lastly, participants expressed their appreciation for Task's Force's focus on geriatric care.

Development of Key Messages
Participants were given an opportunity to develop their own key message statement. They were asked to consider the following:

- *What do you think are the essential words or phrases to be included in the key message statement?*
- *Who do you think are the target audience for this guideline? Who should be reading or hearing these messages?*
- *Do these statements sound conversational? Or are they too advanced and unclear?*

The following key message statements were developed by the participants during the community jury:

- "You can reduce your risk of fractures through simple screening. Talk to your doctor today."
- "Females ≥65 should get screened for fragility fractures. Talk to your doctor for more information"
- "If you are a female ≥65, you should get screened for fragility fractures."

Phase 2 – Decision Aid Perceptions and Feedback

In the second activity, participants were asked to provide their perceptions and feedback about the Bone Health Choice Decision Aid tool developed by the Mayo Clinic in the United States. Please refer to the following link for the online tool:
https://osteoporosisdecisionaid.mayoclinic.org/

This tool is designed for patients to use with their primary care provider to calculate their fragility fracture risk and to decide whether the patient requires further testing and preventive medication. The Task Force is developing a similar tool assess fragility fracture risk in a Canadian context.

The participants were asked the following deliberation questions:

- *Would it be helpful to have this tool in various languages?*

Appendix 7, as supplied by the authors. Appendix to: Thériault G, Limburg H, Klarenbach S, et al. Recommendations on screening for primary prevention of fragility fractures. *CMAJ* 2023. doi: 10.1503/cmaj.221219. Copyright © 2023 The Author(s) or their employer(s). To receive this resource in an accessible format, please contact us at cmajgroup@cmaj.ca.

- *What are your attitudes towards screening in general (example, feelings of anxiety, nervousness, confusion, apprehension?)*
- *Have you ever used a decision aid tool with your primary care provider before?*
- *Do you feel like a tool/decision aid like this would be helpful?*
- *What would help you decide about screening?*
- *Would adding visuals regarding the harms that can be associated with this decision aid (over screening leading to over diagnoses) be helpful for patients? What are other general elements that you would like to see in this decision aid?*

Table 2. Feedback from participants on the decision aid tool.

Deliberation Question	Summary of Feedback
Would it be helpful to have this tool in various languages?	Participants believe the tool should be available in French as Canada is a bilingual country. Furthermore, due to North America's diverse population, other languages to consider are, Spanish, German, Cantonese, Hindi, Urdu, Arabic, Cree etc. At least 10 languages should be represented.<br><br>Participants discussed that it would helpful if the tool is able to read the options aloud in an automated voice.
What are your attitudes towards screening in general (example, feelings of anxiety, nervousness, confusion, apprehension?)	One participant reported they believe there is a lot of anxiety around the COVID-19 screening. There is of pressure to conform to long screening procedures in facilities such as hospitals. For examples, people who walk into the hospital must remove their current mask and use the one provided by the hospital. In addition, invasive questions such as name, age, and date of birth are asked.<br><br>Another patient mentioned that Winnipeg has considered enforcing more security at emergency doors in hospitals for COVID-19. This adds more nervousness and anxiety prior to speaking to a clinician.<br><br>Another participant mentioned that screening makes them nervous. As a result, they avoid screening. They question if their doctor would have the time to go through the tool with them in detail. They believe it would be helpful if they could complete the screening on their own prior to speaking with their doctor.<br><br>Multiple participants believe if they were able to access the fragility fracture tool prior to seeing their doctor, it would help alleviate some stress and anxiety. A patient-centered tool should be considered.

Appendix 7, as supplied by the authors. Appendix to: Thériault G, Limburg H, Klarenbach S, et al. Recommendations on screening for primary prevention of fragility fractures. *CMAJ* 2023. doi: 10.1503/cmaj.221219. Copyright © 2023 The Author(s) or their employer(s). To receive this resource in an accessible format, please contact us at cmajgroup@cmaj.ca.

Would adding visuals regarding the harms that can be associated with this decision aid (over screening leading to over diagnoses) be helpful for patients?	Participants believe that the "100 person" diagram is too complicated. Instead, providing a percentage may be more useful.<br><br>Because this is an online tool, accessibility may be an issue. Many people do not have access to the internet. It is important to consider social and demographic factors.<br><br>Visuals should be colour-blind friendly.<br><br>A printout copy should be available for patients to help with information retention.<br><br>A "How to Use" prompt should be provided for patients to use on their own.<br><br>Another tab for additional resources may be helpful.

**Note: Not all questions were asked due to time constraints.*

Overall Themes

In general, participants felt that the Bone Health Choice Decision Aid was a useful tool to assess fragility fracture risk. However, concerns around accessibility and useability were reoccurring themes. Participants felt that the visuals on the tool were complex and overwhelming at first glance. Instead of a chart to represent 100 people, it would be more useful to provide patients with a percentage to indicate their risk of developing a fragility fracture. Furthermore, participants expressed their concern about using the tool with their clinician. Healthcare providers may not be able to explain the tool in depth due to time constraints. As a result, the Task Force may consider developing a patient-centered version that may be accessible to patients prior to their appointment with their healthcare provider. Participants reported this may alleviate feelings of nervousness and anxiety around screening.

 Copyright © 2023 The Author(s) or their employer(s). To receive this resource in an accessible format, please contact us at cmajgroup@cmaj.ca.

Participant Engagement and Experience

In the post-community jury survey, participants were asked a series of questions (see Appendix A for survey results) about their experience in the project. Participants responded using a 7-item scale, with the following response options: No extent (1), Very small extent (2), Small extent (3), Fair extent (4), Moderate extent (5), Large extent (6), or Very large extent (7).

Two participants completed the post-community jury evaluation survey. Overall, participant experience questions were moderately rated, indicating a positive engagement experience. Both participants indicated that they were able to clearly express their viewpoints, all participants had an equal opportunity to participate in discussions, their ideas were heard, and they felt comfortable contributing their ideas to a large extent.

The participants felt that their input will influence final decisions that underlie the deliberation process to a fair to moderate extent. When asked for more details, participants mentioned that they were unsure how their feedback was considered or incorporated in the final decision.

Overall, participants responded positively to all questions, indicating a sense of clarity and ease surrounding tasks and participation. The lowest score was a 4 and the highest was an 8. Future community juries should consider providing details about patient influence in final decisions.

Suggestions for Applying Findings

From the results, we provide several suggestions for the Task Force's guideline on screening for fragility fractures. Participants should be sent a summary of how their feedback in the final guideline and KT tools was used. Participants felt only fairly convinced that their values and preferences would be included in the final advice. Upon public release of the guideline and KT tools, the Task Force may send an email to participants to explain how their feedback was integrated into the final guideline and KT tools, providing specific examples. The Task Force may also request that participants complete the participant engagement measure again to explore whether participants' beliefs shifted when presented this information.

Conclusion

Through this project we gathered feedback and insight from the TF-PAN network about key message statements and a decision aid tool to assess the risk of fragility fractures. Participants believed there should be a greater effort to make tools and resources patient-centered. There was a concern around usability, accessibility, and ability to understand and apply concepts for the public. Participants were unaware how their input and insight would be incorporated in final decisions. Many participants enjoyed participating in the project and appreciated the opportunity to contribute to Canadian health care. These findings should be integrated into the screening for fragility guideline and KT tools, as well as into future Task Force patient engagement projects.

Appendix 7, as supplied by the authors. Appendix to: Thériault G, Limburg H, Klarenbach S, et al. Recommendations on screening for primary prevention of fragility fractures. *CMAJ* 2023. doi: 10.1503/cmaj.221219. Copyright © 2023 The Author(s) or their employer(s). To receive this resource in an accessible format, please contact us at cmajgroup@cmaj.ca.

References

1) IKT Research Network. (n.d.) Retrieved from URL: https://iktrn.ohri.ca/

2) Canadian Institutes of Health Research. (2014, July). Strategy for Patient-Oriented Research - Patient Engagement Framework. (retrieved from: http://www.cihr-irsc.gc.ca/e/48413.html#a7)

3) Institute of Medicine . Clinical practice guidelines we can trust. Washington: The National Academies Press; 2011. [Google Scholar]

4) Brouwers MC, Kho ME, Browman GP, Burgers JS, Cluzeau F, Feder G, et al. AGREE II: Advancing guideline development, reporting and evaluation in healthcare. Can Med Assoc J. 2010;182:e839–42. doi: 10.1503/cmaj.090449.

5) Qaseem A, Forland F, Macbeth F, Ollenschläger G, Phillips S, van der Wees P, et al. Guidelines International Network: Toward International Standards for Clinical Practice Guidelines. Ann Intern Med. 2012;156:525–531. doi: 10.7326/0003-4819-156-7-201204030-00009

6) National Institute for Health and Care Excellence (NICE). Improving how patients and the public can help develop NICE guidance and standards. 2016. Retrieved from: https://www.nice.org.uk/Media/Default/About/NICE-Communities/Public-involvement/public-involvement-review-consultation-paper.pdf

7) Rashid, A., Thomas, V., Shaw, T. et al. Patient and public involvement in the development of healthcare guidance: An overview of current methods and future challenges. *The Patient.* 2017;10(3), 277-282. https://doi.org/10.1007/s40271-016-0206-8

8) Black, A., Strain, K., Wallsworth, C., Chalrton, S-G., Chang, W., McNamee, K., & Hamilton, C. What constitutes meaningful engagement for patients and families as partners on research teams? *Journal of Health Services Research & Policy.* 2018;23(3), 158-167. doi: 10.1177/1355819618762960

9) Armstrong, M.J., Mullins, C.D., Gronseth, G.S., Gagliardi, A.R. Impact of patient involvement on clinical practice guideline development: a parallel group study. *Implementation Science*. 2018;13:55 DOI: https://doi.org/10.1186/s13012-018-0745-6

10) Armstrong, M.J., Rueda, J.D., Gronseth, G.S., & Mullins, C.D. Framework for enhancing clinical practice guidelines through continuous patient engagement. *Health Expectations.* 2016;20, 3-10. doi: 10.1111/hex.12467

Appendix 7, as supplied by the authors. Appendix to: Thériault G, Limburg H, Klarenbach S, et al. Recommendations on screening for primary prevention of fragility fractures. *CMAJ* 2023. doi: 10.1503/cmaj.221219. Copyright © 2023 The Author(s) or their employer(s). To receive this resource in an accessible format, please contact us at cmajgroup@cmaj.ca.

11) Holmes, B.J., Bryan, S., Ho, K., and McGavin, C. Engaging patients as partners in health research: Lessons from BC, Canada. *Healthcare Management Forum*. 2018;31(2) 41-44.doi: 10.1177/0840470417741712

12) Brouwers, M.C., Vukmirovic, M., Spithoff, K., and Makarski, J. Understanding optimal approaches to patient and caregiver engagement in the development of cancer practice guidelines; a mixed methods study. *BMC Health Services Research*. 2017; 17:186

13) Thomas R, Scott AM, Sims R, et al. Exploring women's priorities for the potential consequences of a gestational diabetes diagnosis: A pilot community jury. Health Expectations : an International Journal of Public Participation in Health Care and Health Policy. 2020 Jun;23(3):593-602. DOI: 10.1111/hex.13036.

Appendix 7, as supplied by the authors. Appendix to: Thériault G, Limburg H, Klarenbach S, et al. Recommendations on screening for primary prevention of fragility fractures. *CMAJ* 2023. doi: 10.1503/cmaj.221219. Copyright © 2023 The Author(s) or their employer(s). To receive this resource in an accessible format, please contact us at cmajgroup@cmaj.ca.

Appendix A: Post-Community Jury Evaluation Survey

Thank you for considering participating in our research study.

The purpose of this questionnaire is to evaluate your experience with engaging in community juries as a part of the TF-PAN. This survey will use your feedback to improve implementation processes for future cohorts. Full information on the study and the survey can be found in the information sheet that was attached to the invitation email that you received.

A few reminders:

• Participation in this survey is voluntary, your consent is demonstrated by completion and submission of this survey.

• The questionnaire should take about 10-20 minutes.

• If a question is not applicable to you or you feel uncomfortable answering it, you may skip the question.

• You should not include any identifiable information in the questionnaire. We will be able to link your responses back to you by using your study ID number, but to minimize the risk of others knowing who you are, we do not want you to put any identifiable information into the online survey.

• There are no major risks to completing this survey. The results of this survey will not benefit you directly. This survey is hosted on Qualtrics, which is a secure online data collection system. Results will be stored on Qualtrics' servers until downloaded onto our secure server for analysis. Qualtrics servers are protected by high-end firewall systems, and scans are performed regularly to ensure that any vulnerabilities are quickly found and patched. Data are stored in a specific Canadian location; data do not float around in the "cloud." In addition, all data are processed in Canada, and are not moved to another jurisdictional area (e.g., outside Canada).

• Access to the study data will be limited to the study investigators, study team members, and the Unity Health Toronto Research Ethics Board for the purposes of monitoring the study.

• If you have any questions regarding your rights as a research participant, you may contact the Chair of the Unity Health Toronto Research Ethics Board at **416-864-6060 ext. 42557** during business hours. • For more information, please refer to the letter of information for details. If you have any questions, or concerns, please do not hesitate to contact the Research Assistant, **Sarah Deshpande at Sarah.Deshpande@unityhealth.to.**

If you would like to continue, please click the **"Next"** button below. If you would **NOT** like to continue, please close the browser window and do not continue. We thank you for your time and due consideration.

Appendix 7, as supplied by the authors. Appendix to: Thériault G, Limburg H, Klarenbach S, et al. Recommendations on screening for primary prevention of fragility fractures. *CMAJ* 2023. doi: 10.1503/cmaj.221219. Copyright © 2023 The Author(s) or their employer(s). To receive this resource in an accessible format, please contact us at cmajgroup@cmaj.ca.

Q2 - Thank you for your interest in participating in this survey. Please indicate your level of agreement with each statement and check only one box for each statement. You may provide additional feedback in the comment boxes provided at the end the survey. Please enter your participant ID as provided in the email:

Thank you for your interest in participating in this survey. Please indicate your level of agreement with each statement and check only one box for each statement. You may provide additional feedback in the comment boxes provided at the end the survey. Please enter your participant ID as provided in the email:
2020
3003

St. Michael's Inspired Care. Inspiring Science.
UNITY HEALTH TORONTO

Appendix 7, as supplied by the authors. Appendix to: Thériault G, Limburg H, Klarenbach S, et al. Recommendations on screening for primary prevention of fragility fractures. *CMAJ* 2023. doi: 10.1503/cmaj.221219. Copyright © 2023 The Author(s) or their employer(s). To receive this resource in an accessible format, please contact us at cmajgroup@cmaj.ca.

Q3 - How many community juries have you been a part of? Please specify the number below.

How many community juries have you been a part of? Please specify the number below.
2
1

Appendix 7, as supplied by the authors. Appendix to: Thériault G, Limburg H, Klarenbach S, et al. Recommendations on screening for primary prevention of fragility fractures. *CMAJ* 2023. doi: 10.1503/cmaj.221219. Copyright © 2023 The Author(s) or their employer(s). To receive this resource in an accessible format, please contact us at cmajgroup@cmaj.ca.

St. Michael's
Inspired Care.
Inspiring Science.
UNITY HEALTH TORONTO

Q4 respect - Question 1

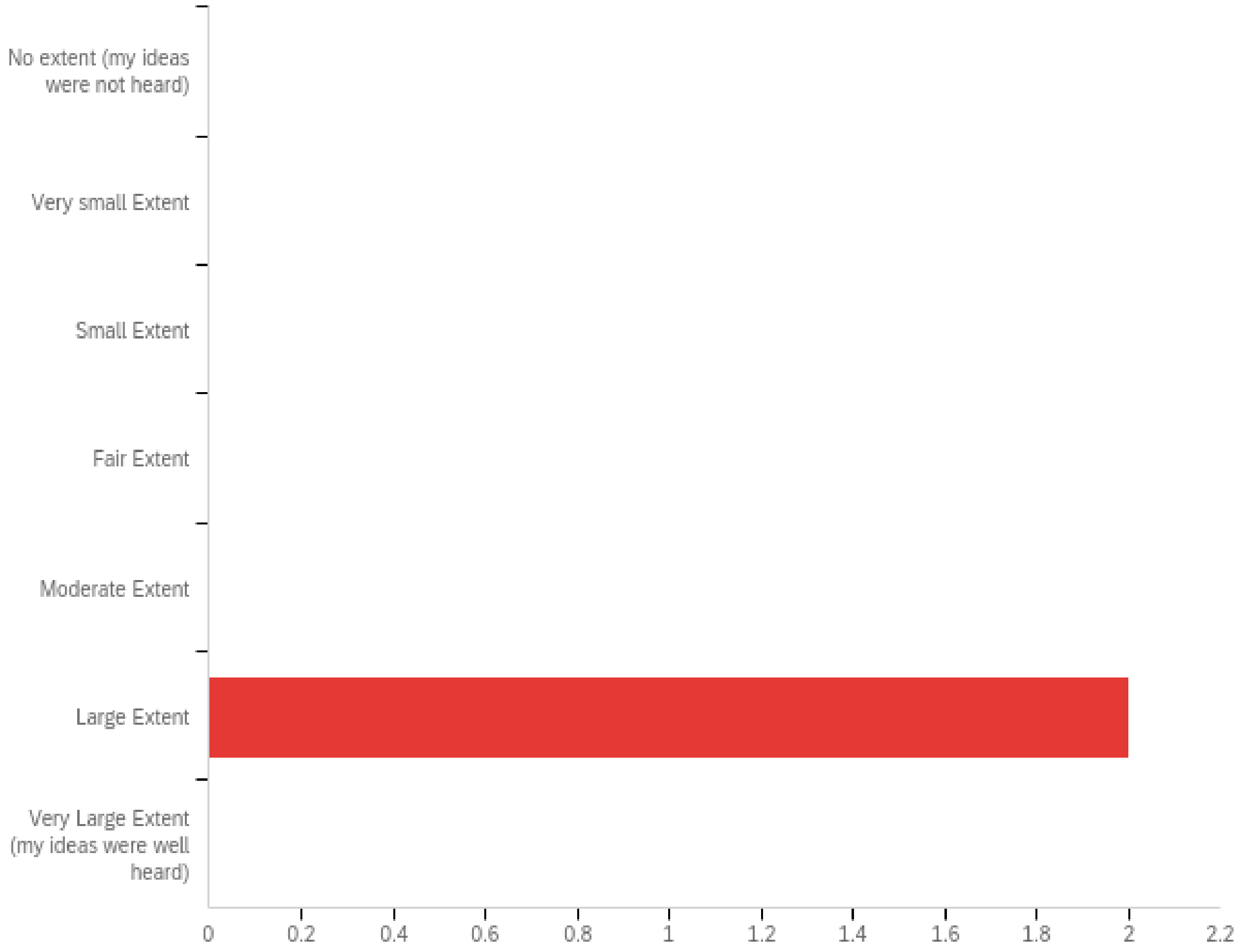

#	Field	Minimum	Maximum	Mean	Std Deviation	Variance	Count
1	To what extent do you believe that your ideas were heard during the deliberation process?	6.00	6.00	6.00	0.00	0.00	2

#	Answer	%	Count
1	No extent (my ideas were not heard)	0.00%	0
2	Very small Extent	0.00%	0
3	Small Extent	0.00%	0
4	Fair Extent	0.00%	0

Appendix 7, as supplied by the authors. Appendix to: Thériault G, Limburg H, Klarenbach S, et al. Recommendations on screening for primary prevention of fragility fractures. *CMAJ* 2023. doi: 10.1503/cmaj.221219. Copyright © 2023 The Author(s) or their employer(s). To receive this resource in an accessible format, please contact us at cmajgroup@cmaj.ca.

5	Moderate Extent	0.00%	0
6	Large Extent	100.00%	2
7	Very Large Extent (my ideas were well heard)	0.00%	0
	Total	100%	2

Appendix 7, as supplied by the authors. Appendix to: Thériault G, Limburg H, Klarenbach S, et al. Recommendations on screening for primary prevention of fragility fractures. *CMAJ* 2023. doi: 10.1503/cmaj.221219. Copyright © 2023 The Author(s) or their employer(s). To receive this resource in an accessible format, please contact us at cmajgroup@cmaj.ca.

St. Michael's
Inspired Care.
Inspiring Science.
UNITY HEALTH
TORONTO

Q6 - For Question 1, please explain your rating in the space below:

For Question 1, please explain your rating in the space below:
as all ideas that were discussed and questions asked were answered with respect

Appendix 7, as supplied by the authors. Appendix to: Thériault G, Limburg H, Klarenbach S, et al. Recommendations on screening for primary prevention of fragility fractures. *CMAJ* 2023. doi: 10.1503/cmaj.221219. Copyright © 2023 The Author(s) or their employer(s). To receive this resource in an accessible format, please contact us at cmajgroup@cmaj.ca.

Q7 respect - Question 2

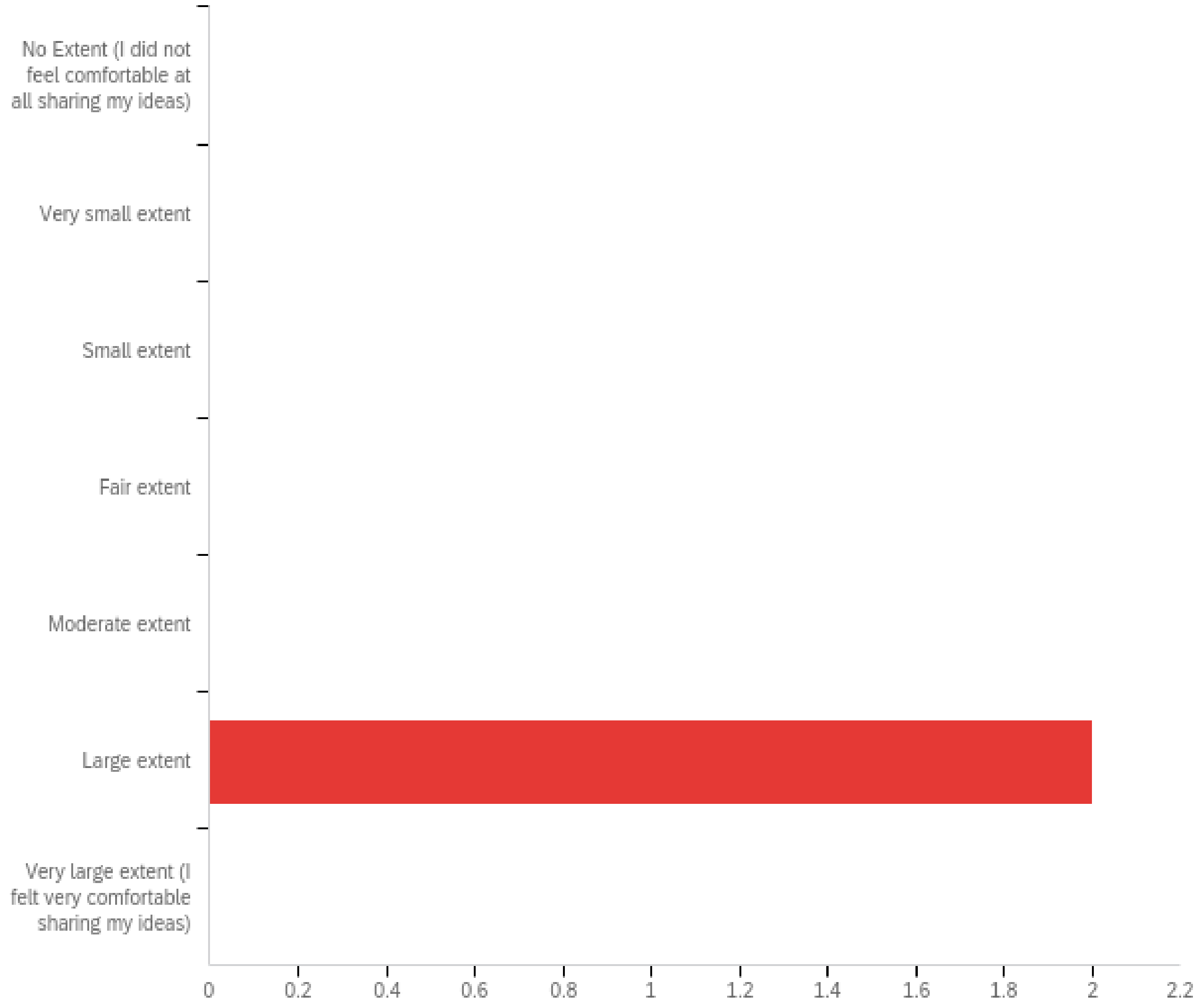

#	Field	Minimum	Maximum	Mean	Std Deviation	Variance	Count
1	To what extent did you feel comfortable contributing your ideas to the deliberation process?	6.00	6.00	6.00	0.00	0.00	2

#	Answer	%	Count
1	No Extent (I did not feel comfortable at all sharing my ideas)	0.00%	0
2	Very small extent	0.00%	0
3	Small extent	0.00%	0

St. Michael's Inspired Care. Inspiring Science.
UNITY HEALTH TORONTO

Appendix 7, as supplied by the authors. Appendix to: Thériault G, Limburg H, Klarenbach S, et al. Recommendations on screening for primary prevention of fragility fractures. *CMAJ* 2023. doi: 10.1503/cmaj.221219. Copyright © 2023 The Author(s) or their employer(s). To receive this resource in an accessible format, please contact us at cmajgroup@cmaj.ca.

4	Fair extent	0.00%	0
5	Moderate extent	0.00%	0
6	Large extent	100.00%	2
7	Very large extent (I felt very comfortable sharing my ideas)	0.00%	0
	Total	100%	2

Appendix 7, as supplied by the authors. Appendix to: Thériault G, Limburg H, Klarenbach S, et al. Recommendations on screening for primary prevention of fragility fractures. *CMAJ* 2023. doi: 10.1503/cmaj.221219. Copyright © 2023 The Author(s) or their employer(s). To receive this resource in an accessible format, please contact us at cmajgroup@cmaj.ca.

Q8 - For Question 2, please explain your rating in the space below.

For Question 2, please explain your rating in the space below.
i am comfortable in sharing my ideas

Appendix 7, as supplied by the authors. Appendix to: Thériault G, Limburg H, Klarenbach S, et al. Recommendations on screening for primary prevention of fragility fractures. *CMAJ* 2023. doi: 10.1503/cmaj.221219. Copyright © 2023 The Author(s) or their employer(s). To receive this resource in an accessible format, please contact us at cmajgroup@cmaj.ca.

St. Michael's
Inspired Care.
Inspiring Science.
UNITY HEALTH TORONTO

Q9 Trust - Question 3

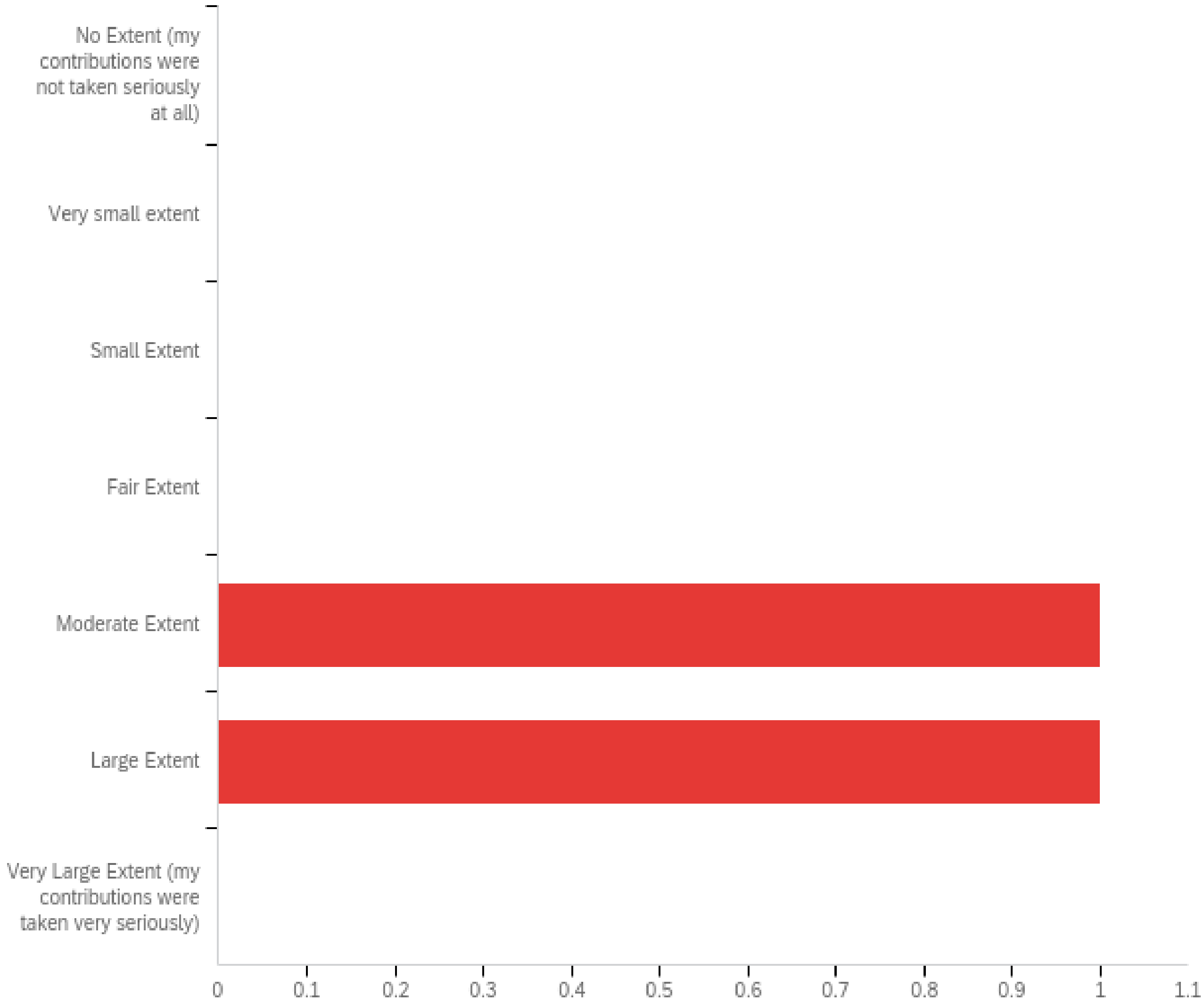

#	Field	Minimum	Maximum	Mean	Std Deviation	Variance	Count
1	To what extent do you believe community jury organizers took your contributions to the deliberation process seriously?	5.00	6.00	5.50	0.50	0.25	2

#	Answer	%	Count
1	No Extent (my contributions were not taken seriously at all)	0.00%	0
2	Very small extent	0.00%	0

Appendix 7, as supplied by the authors. Appendix to: Thériault G, Limburg H, Klarenbach S, et al. Recommendations on screening for primary prevention of fragility fractures. *CMAJ* 2023. doi: 10.1503/cmaj.221219. Copyright © 2023 The Author(s) or their employer(s). To receive this resource in an accessible format, please contact us at cmajgroup@cmaj.ca.

3	Small Extent	0.00%	0
4	Fair Extent	0.00%	0
5	Moderate Extent	50.00%	1
6	Large Extent	50.00%	1
7	Very Large Extent (my contributions were taken very seriously)	0.00%	0
	Total	100%	2

Appendix 7, as supplied by the authors. Appendix to: Thériault G, Limburg H, Klarenbach S, et al. Recommendations on screening for primary prevention of fragility fractures. *CMAJ* 2023. doi: 10.1503/cmaj.221219. Copyright © 2023 The Author(s) or their employer(s). To receive this resource in an accessible format, please contact us at cmajgroup@cmaj.ca.

Q10 - For Question 3, please explain your rating in the space below.

For Question 3, please explain your rating in the space below.
they provided feedback on how and what our contribution will be utilized

St. Michael's
Inspired Care.
Inspiring Science.

UNITY HEALTH TORONTO

Appendix 7, as supplied by the authors. Appendix to: Thériault G, Limburg H, Klarenbach S, et al. Recommendations on screening for primary prevention of fragility fractures. *CMAJ* 2023. doi: 10.1503/cmaj.221219. Copyright © 2023 The Author(s) or their employer(s). To receive this resource in an accessible format, please contact us at cmajgroup@cmaj.ca.

Q11: Trust - Question 4

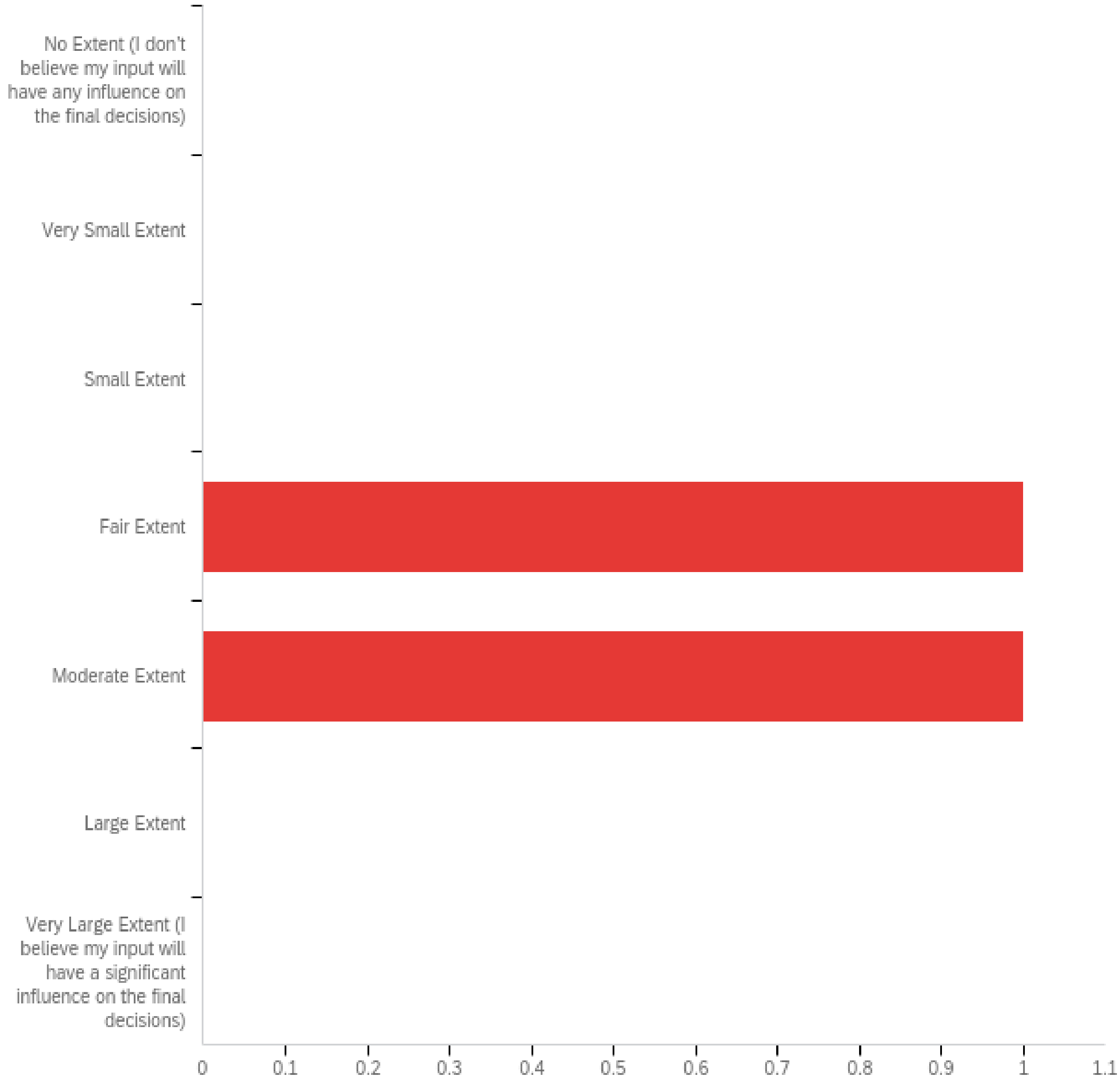

#	Field	Minimum	Maximum	Mean	Std Deviation	Variance	Count
1	To what extent do you believe that your input will influence final decisions that underlie the deliberation process?	4.00	5.00	4.50	0.50	0.25	2

Appendix 7, as supplied by the authors. Appendix to: Thériault G, Limburg H, Klarenbach S, et al. Recommendations on screening for primary prevention of fragility fractures. *CMAJ* 2023. doi: 10.1503/cmaj.221219. Copyright © 2023 The Author(s) or their employer(s). To receive this resource in an accessible format, please contact us at cmajgroup@cmaj.ca.

#	Answer	%	Count
1	No Extent (I don’t believe my input will have any influence on the final decisions)	0.00%	0
2	Very Small Extent	0.00%	0
3	Small Extent	0.00%	0
4	Fair Extent	50.00%	1
5	Moderate Extent	50.00%	1
6	Large Extent	0.00%	0
7	Very Large Extent (I believe my input will have a significant influence on the final decisions)	0.00%	0
	Total	100%	2

Appendix 7, as supplied by the authors. Appendix to: Thériault G, Limburg H, Klarenbach S, et al. Recommendations on screening for primary prevention of fragility fractures. *CMAJ* 2023. doi: 10.1503/cmaj.221219. Copyright © 2023 The Author(s) or their employer(s). To receive this resource in an accessible format, please contact us at cmajgroup@cmaj.ca.

Q12 - For Question 4, please explain your rating in the space below.

For Question 4, please explain your rating in the space below.
as my feedback was for consideration and feel that if they want to use they will
We haven't had feedback back to us as to how our comments were used or not. It's important to 'close the loop' with participants and show how their feedback was used

Appendix 7, as supplied by the authors. Appendix to: Thériault G, Limburg H, Klarenbach S, et al. Recommendations on screening for primary prevention of fragility fractures. *CMAJ* 2023. doi: 10.1503/cmaj.221219. Copyright © 2023 The Author(s) or their employer(s). To receive this resource in an accessible format, please contact us at cmajgroup@cmaj.ca.

Q13: Legitimacy - Question 5

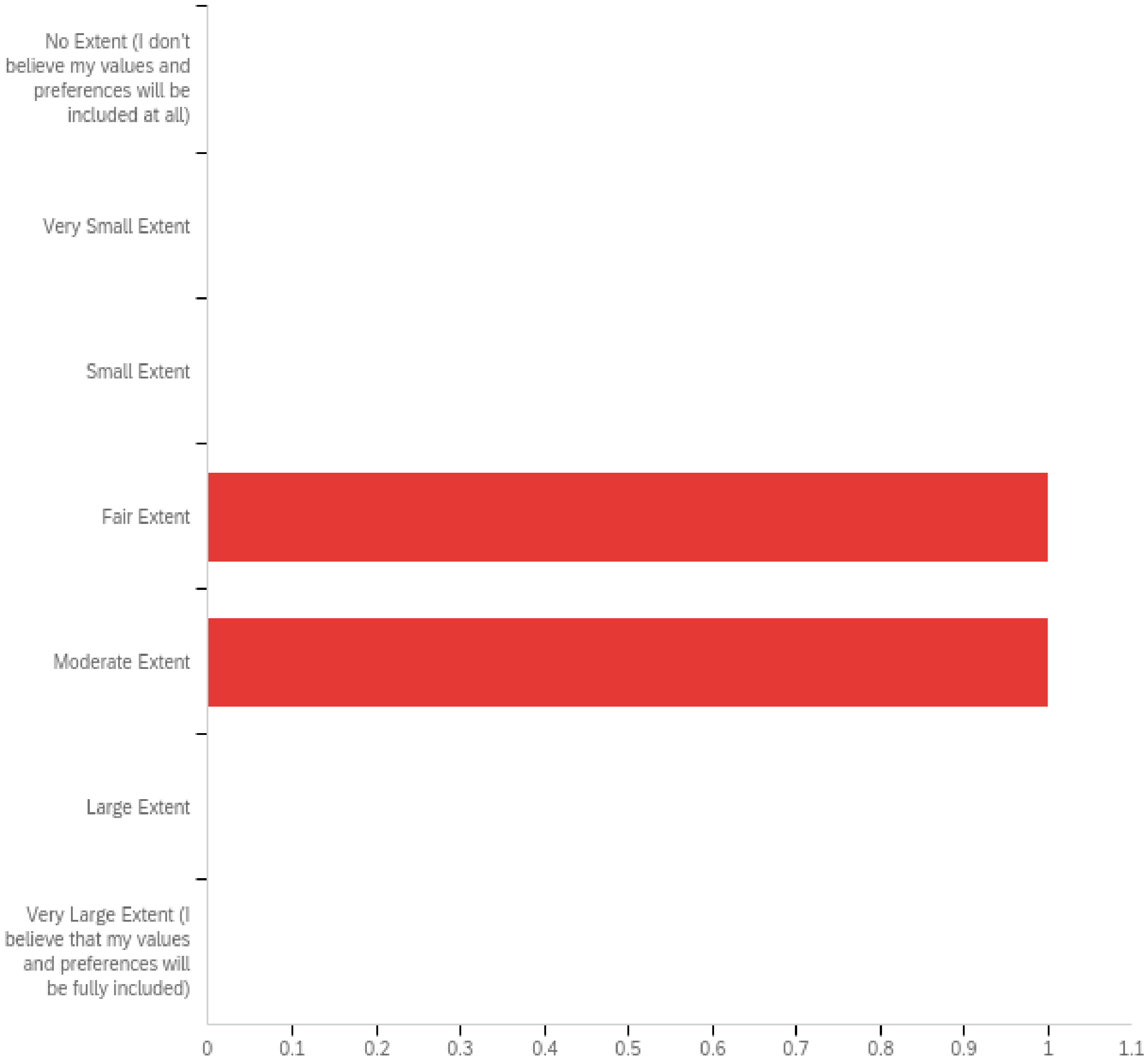

#	Field	Minimum	Maximum	Mean	Std Deviation	Variance	Count
1	To what extent do you believe that your values and preferences will be included in the final health advice/ guidelines from this process?	4.00	5.00	4.50	0.50	0.25	2

Appendix 7, as supplied by the authors. Appendix to: Thériault G, Limburg H, Klarenbach S, et al. Recommendations on screening for primary prevention of fragility fractures. *CMAJ* 2023. doi: 10.1503/cmaj.221219.

Copyright © 2023 The Author(s) or their employer(s). To receive this resource in an accessible format, please contact us at cmajgroup@cmaj.ca.

#	Answer	%	Count
1	No Extent (I don’t believe my values and preferences will be included at all)	0.00%	0
2	Very Small Extent	0.00%	0
3	Small Extent	0.00%	0
4	Fair Extent	50.00%	1
5	Moderate Extent	50.00%	1
6	Large Extent	0.00%	0
7	Very Large Extent (I believe that my values and preferences will be fully included)	0.00%	0
	Total	100%	2

Appendix 7, as supplied by the authors. Appendix to: Thériault G, Limburg H, Klarenbach S, et al. Recommendations on screening for primary prevention of fragility fractures. *CMAJ* 2023. doi: 10.1503/cmaj.221219. Copyright © 2023 The Author(s) or their employer(s). To receive this resource in an accessible format, please contact us at cmajgroup@cmaj.ca.

Q14 - For Question 5, please explain your rating in the space below.

For Question 5, please explain your rating in the space below.
havent heard back if my values or preference was used
I'm not clear how much influence our comments will have and who makes the final decision. Perhaps making clear what influence our thoughts have would be useful (eg. using the IAP2 Spectrum of Engagement: https://iap2.org.au/wp-content/uploads/2019/07/IAP2_Public_Participation_Spectrum.pdf

St. Michael's
Inspired Care.
Inspiring Science.
UNITY HEALTH TORONTO

Appendix 7, as supplied by the authors. Appendix to: Thériault G, Limburg H, Klarenbach S, et al. Recommendations on screening for primary prevention of fragility fractures. *CMAJ* 2023. doi: 10.1503/cmaj.221219. Copyright © 2023 The Author(s) or their employer(s). To receive this resource in an accessible format, please contact us at cmajgroup@cmaj.ca.

Q15: Legitimacy - Question 6: To what extent were you able to clearly express your viewpoints?

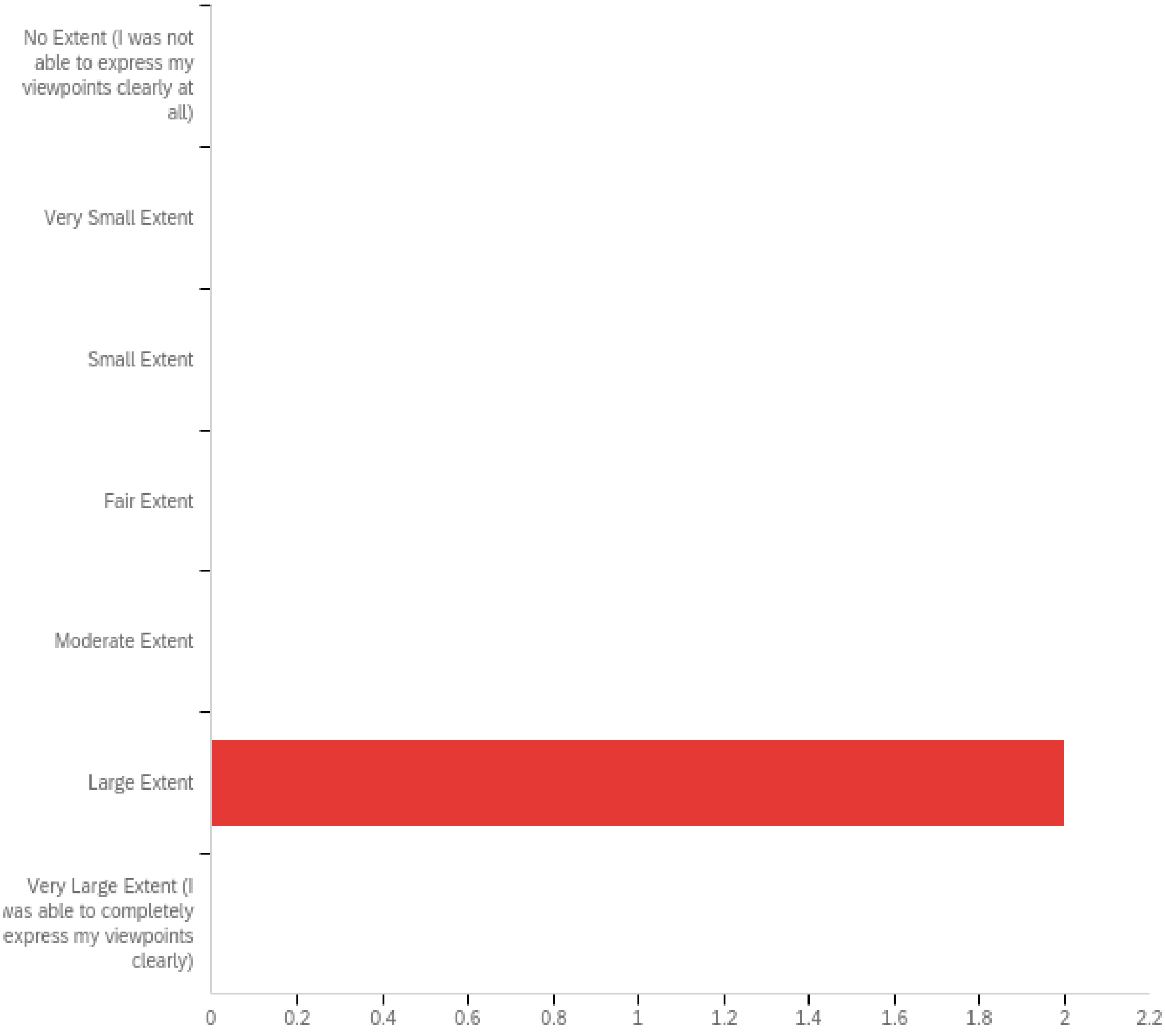

#	Field	Minimum	Maximum	Mean	Std Deviation	Variance	Count
1	To what extent were you able to clearly express your viewpoints?	6.00	6.00	6.00	0.00	0.00	2

#	Answer	%	Count
1	No Extent (I was not able to express my viewpoints clearly at all)	0.00%	0

St. Michael's Inspired Care. Inspiring Science.
UNITY HEALTH TORONTO

Appendix 7, as supplied by the authors. Appendix to: Thériault G, Limburg H, Klarenbach S, et al. Recommendations on screening for primary prevention of fragility fractures. *CMAJ* 2023. doi: 10.1503/cmaj.221219. Copyright © 2023 The Author(s) or their employer(s). To receive this resource in an accessible format, please contact us at cmajgroup@cmaj.ca.

2	Very Small Extent	0.00%	0
3	Small Extent	0.00%	0
4	Fair Extent	0.00%	0
5	Moderate Extent	0.00%	0
6	Large Extent	100.00%	2
7	Very Large Extent (I was able to completely express my viewpoints clearly)	0.00%	0
	Total	100%	2

Appendix 7, as supplied by the authors. Appendix to: Thériault G, Limburg H, Klarenbach S, et al. Recommendations on screening for primary prevention of fragility fractures. *CMAJ* 2023. doi: 10.1503/cmaj.221219.
Copyright © 2023 The Author(s) or their employer(s). To receive this resource in an accessible format, please contact us at cmajgroup@cmaj.ca.

Q16 - For Question 6, please explain your rating in the space below.

For Question 6, please explain your rating in the space below.
i am very comfortable in expressing myself

Appendix 7, as supplied by the authors. Appendix to: Thériault G, Limburg H, Klarenbach S, et al. Recommendations on screening for primary prevention of fragility fractures. *CMAJ* 2023. doi: 10.1503/cmaj.221219. Copyright © 2023 The Author(s) or their employer(s). To receive this resource in an accessible format, please contact us at cmajgroup@cmaj.ca.

Q17: Legitimacy - Question 7

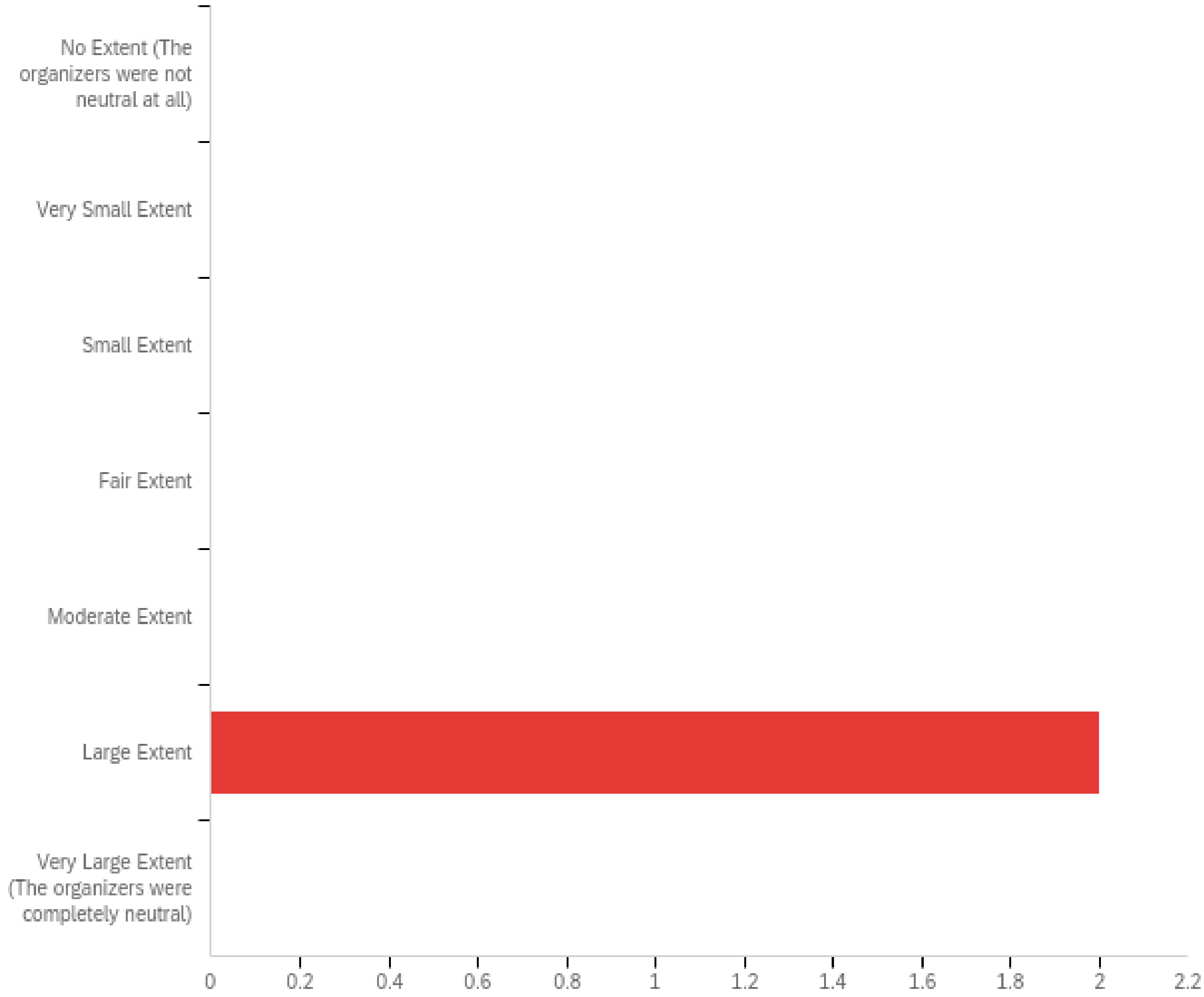

#	Field	Minimum	Maximum	Mean	Std Deviation	Variance	Count
1	To what extent were organizers neutral in their opinions (regarding topics) during the deliberation process?	6.00	6.00	6.00	0.00	0.00	2

#	Answer	%	Count
1	No Extent (The organizers were not neutral at all)	0.00%	0
2	Very Small Extent	0.00%	0
3	Small Extent	0.00%	0

Appendix 7, as supplied by the authors. Appendix to: Thériault G, Limburg H, Klarenbach S, et al. Recommendations on screening for primary prevention of fragility fractures. *CMAJ* 2023. doi: 10.1503/cmaj.221219. Copyright © 2023 The Author(s) or their employer(s). To receive this resource in an accessible format, please contact us at cmajgroup@cmaj.ca.

4	Fair Extent	0.00%	0
5	Moderate Extent	0.00%	0
6	Large Extent	100.00%	2
7	Very Large Extent (The organizers were completely neutral)	0.00%	0
	Total	100%	2

Appendix 7, as supplied by the authors. Appendix to: Thériault G, Limburg H, Klarenbach S, et al. Recommendations on screening for primary prevention of fragility fractures. *CMAJ* 2023. doi: 10.1503/cmaj.221219. Copyright © 2023 The Author(s) or their employer(s). To receive this resource in an accessible format, please contact us at cmajgroup@cmaj.ca.

Q18 - For Question 7, please explain your rating in the space below

For Question 7, please explain your rating in the space below
they asked open ended questions and for clarity in our responses and indicated they are noting our responses

Appendix 7, as supplied by the authors. Appendix to: Thériault G, Limburg H, Klarenbach S, et al. Recommendations on screening for primary prevention of fragility fractures. *CMAJ* 2023. doi: 10.1503/cmaj.221219. Copyright © 2023 The Author(s) or their employer(s). To receive this resource in an accessible format, please contact us at cmajgroup@cmaj.ca.

Q19: Fairness - Question 8

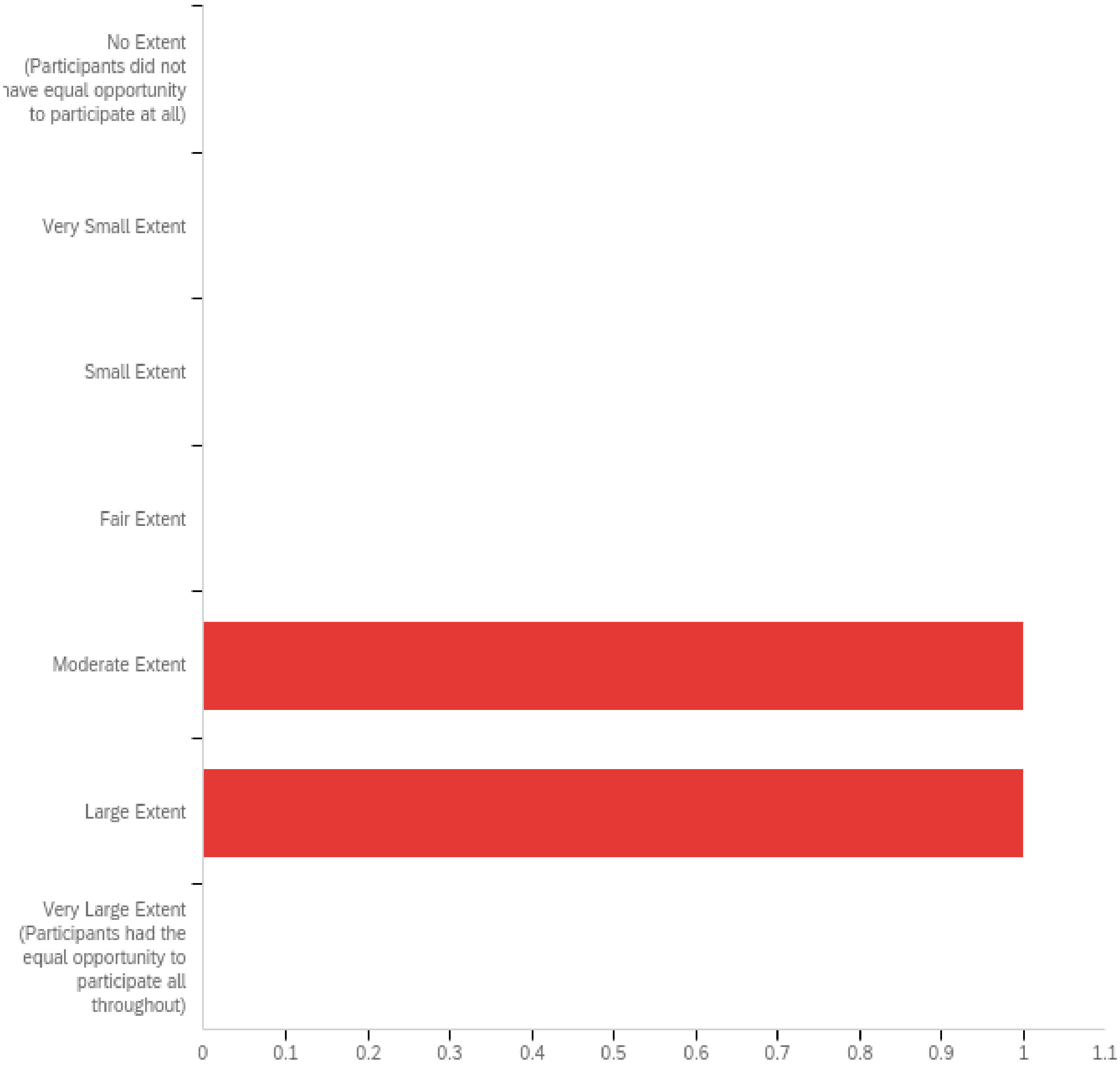

#	Field	Minimum	Maximum	Mean	Std Deviation	Variance	Count
1	To what extent did all participants have equal opportunity to participate in discussions?	5.00	6.00	5.50	0.50	0.25	2

#	Answer	%	Count
1	No Extent (Participants did not have equal opportunity to participate at all)	0.00%	0
2	Very Small Extent	0.00%	0
3	Small Extent	0.00%	0
4	Fair Extent	0.00%	0
5	Moderate Extent	50.00%	1
6	Large Extent	50.00%	1
7	Very Large Extent (Participants had the equal opportunity to participate all throughout)	0.00%	0
	Total	100%	2

Appendix 7, as supplied by the authors. Appendix to: Thériault G, Limburg H, Klarenbach S, et al. Recommendations on screening for primary prevention of fragility fractures. *CMAJ* 2023. doi: 10.1503/cmaj.221219. Copyright © 2023 The Author(s) or their employer(s). To receive this resource in an accessible format, please contact us at cmajgroup@cmaj.ca.

Q20 - For Question 8, please explain your rating in the space below.

For Question 8, please explain your rating in the space below.
everyone that was involved were able to express themselves clearly
There were some participants that didn't talk as much, so perhaps a way to engage quieter voices would be useful

St. Michael's
Inspired Care.
Inspiring Science.
UNITY HEALTH TORONTO

Appendix 7, as supplied by the authors. Appendix to: Thériault G, Limburg H, Klarenbach S, et al. Recommendations on screening for primary prevention of fragility fractures. *CMAJ* 2023. doi: 10.1503/cmaj.221219. Copyright © 2023 The Author(s) or their employer(s). To receive this resource in an accessible format, please contact us at cmajgroup@cmaj.ca.

Q21: Fairness - Question 9

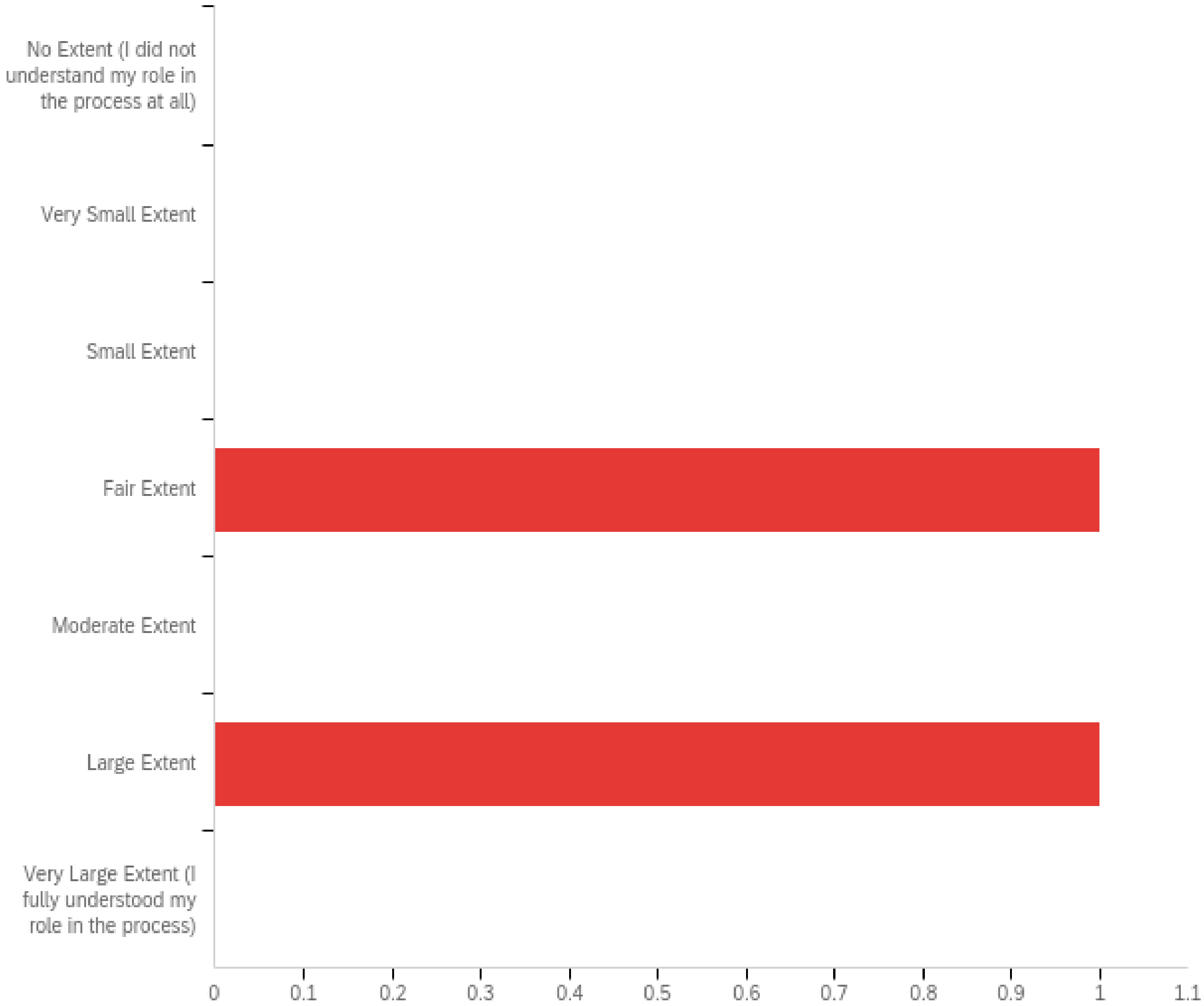

#	Field	Minimum	Maximum	Mean	Std Deviation	Variance	Count
1	To what extent did you clearly understand your role in the process?	4.00	6.00	5.00	1.00	1.00	2

#	Answer	%	Count
1	No Extent (I did not understand my role in the process at all)	0.00%	0
2	Very Small Extent	0.00%	0
3	Small Extent	0.00%	0

Appendix 7, as supplied by the authors. Appendix to: Thériault G, Limburg H, Klarenbach S, et al. Recommendations on screening for primary prevention of fragility fractures. *CMAJ* 2023. doi: 10.1503/cmaj.221219. Copyright © 2023 The Author(s) or their employer(s). To receive this resource in an accessible format, please contact us at cmajgroup@cmaj.ca.

4	Fair Extent	50.00%	1
5	Moderate Extent	0.00%	0
6	Large Extent	50.00%	1
7	Very Large Extent (I fully understood my role in the process)	0.00%	0
	Total	100%	2

Appendix 7, as supplied by the authors. Appendix to: Thériault G, Limburg H, Klarenbach S, et al. Recommendations on screening for primary prevention of fragility fractures. *CMAJ* 2023. doi: 10.1503/cmaj.221219. Copyright © 2023 The Author(s) or their employer(s). To receive this resource in an accessible format, please contact us at cmajgroup@cmaj.ca.

Q22 - For Question 9, please explain your rating in the space below.

For Question 9, please explain your rating in the space below.
the moderators clearly indicated how and why we were participating in the process
I've been a part of a deliberative dialogue research project as a participant and this process didn't seem like that. It seemed more like it was a feedback session to give thoughts on what the Task Force has come up with.

Appendix 7, as supplied by the authors. Appendix to: Thériault G, Limburg H, Klarenbach S, et al. Recommendations on screening for primary prevention of fragility fractures. *CMAJ* 2023. doi: 10.1503/cmaj.221219. Copyright © 2023 The Author(s) or their employer(s). To receive this resource in an accessible format, please contact us at cmajgroup@cmaj.ca.

Q23: Competence - Question 10

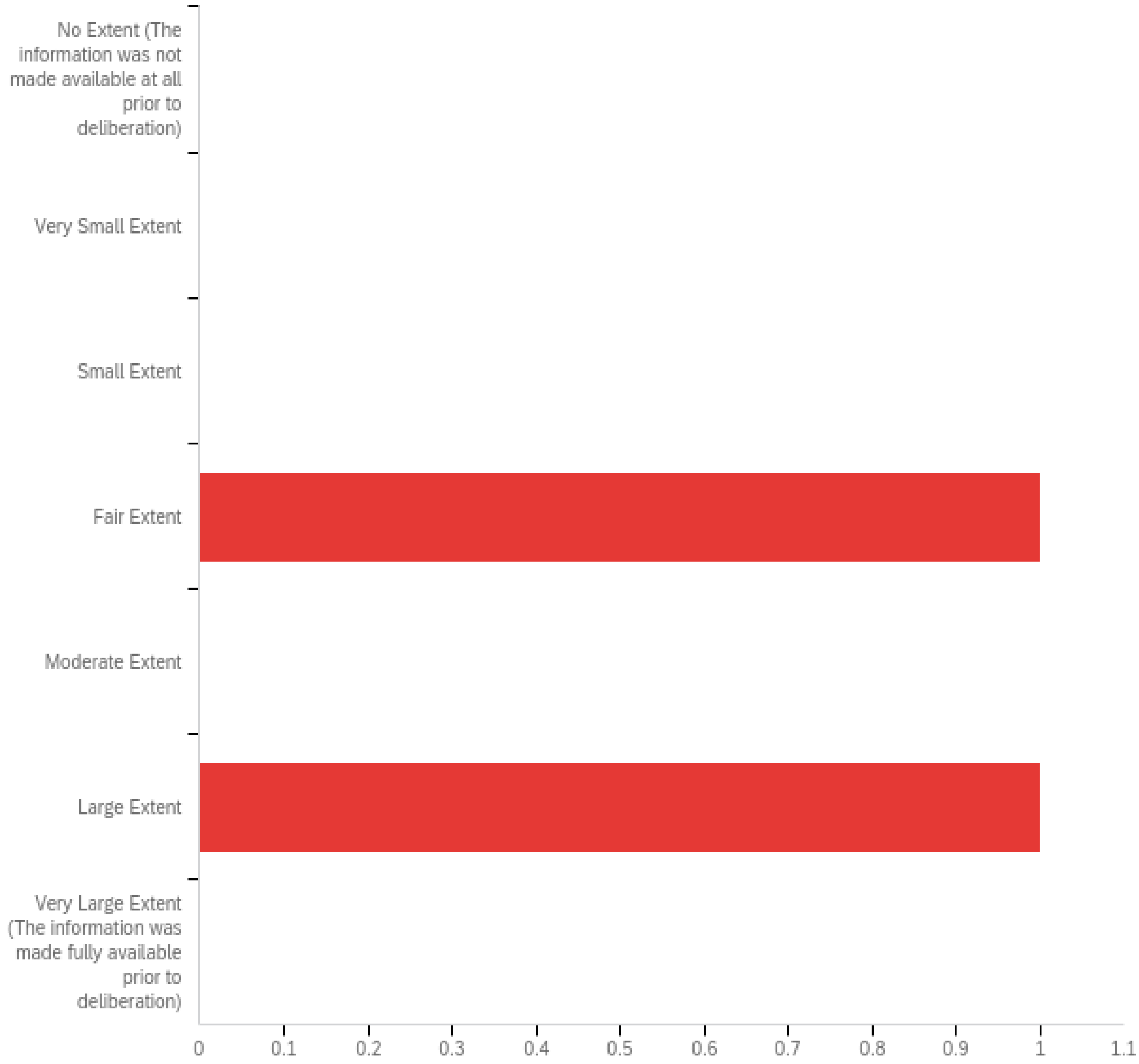

#	Field	Minimum	Maximum	Mean	Std Deviation	Variance	Count
1	To what extent was information made available to you either prior or during the deliberation process so as to participate knowledgeably in the process?	4.00	6.00	5.00	1.00	1.00	2

St. Michael's Inspired Care. Inspiring Science.
UNITY HEALTH TORONTO

Appendix 7, as supplied by the authors. Appendix to: Thériault G, Limburg H, Klarenbach S, et al. Recommendations on screening for primary prevention of fragility fractures. *CMAJ* 2023. doi: 10.1503/cmaj.221219. Copyright © 2023 The Author(s) or their employer(s). To receive this resource in an accessible format, please contact us at cmajgroup@cmaj.ca.

#	Answer	%	Count
1	No Extent (The information was not made available at all prior to deliberation)	0.00%	0
2	Very Small Extent	0.00%	0
3	Small Extent	0.00%	0
4	Fair Extent	50.00%	1
5	Moderate Extent	0.00%	0
6	Large Extent	50.00%	1
7	Very Large Extent (The information was made fully available prior to deliberation)	0.00%	0
	Total	100%	2

Appendix 7, as supplied by the authors. Appendix to: Thériault G, Limburg H, Klarenbach S, et al. Recommendations on screening for primary prevention of fragility fractures. *CMAJ* 2023. doi: 10.1503/cmaj.221219. Copyright © 2023 The Author(s) or their employer(s). To receive this resource in an accessible format, please contact us at cmajgroup@cmaj.ca.

Q24 - For Question 10, please explain your rating in the space below.

For Question 10, please explain your rating in the space below.
received and had time review the information before meeting
Send the slides ahead of time so we have them as a reference. There was a lot of info that was covered and it is challenging to remember all of the details when this is all new info to me

St. Michael's
Inspired Care.
Inspiring Science.
UNITY HEALTH TORONTO

Appendix 7, as supplied by the authors. Appendix to: Thériault G, Limburg H, Klarenbach S, et al. Recommendations on screening for primary prevention of fragility fractures. *CMAJ* 2023. doi: 10.1503/cmaj.221219. Copyright © 2023 The Author(s) or their employer(s). To receive this resource in an accessible format, please contact us at cmajgroup@cmaj.ca.

Appendix B: Deliberation Guide

Part 1: Welcome, Introductions and Ground Rules

Welcome __________ *Greet people as they join the zoom conference*
Before we get started, I just wanted to make sure that everyone can hear me and see me. *Wait for confirmation.* Okay great, I also wanted to ensure that everyone is aware of the mute and unmute function. To unmute yourself and begin talking, please click the unmute button (microphone icon) in the bottom-left corner of the meeting window. When you are finished speaking or when you want to mute yourself, similarly, click the same microphone icon in the bottom-left corner of the meeting window. Please feel free to unmute yourself when you have a question or if there is something you want to add. There is also a chat feature that allows you to send instant messages within our meeting. Feel free to use the chat to ask any questions or make comments, Sarah and I will both be monitoring the chat through the session. If no one has any immediate questions, we can begin!

Hello everyone and thank you for joining us today for the second TF-PAN community jury session with the Canadian Task Force on Preventive Health Care. In the last community jury, we were able to discuss the development process of the Task Force fragility fractures guideline. Today, at our second community jury session, we will delve more into coming up with key message statements for the guideline, as well as review the decision aid tool more in depth. We will be going over all the instructions and objectives before we begin.

My name is ___________ and I am from the Knowledge Translation Program at the Li Ka Shing Knowledge Institute of St. Michaels Hospital, and I am going to be the moderator today alongside ________ who is my colleague who will also be co-moderating with me today. Today, Guylene is back with us for the first 30 minutes, along with Heather Limburg of the Task Force's fragility fractures guideline working group who will be answering your questions, should you have any.

Purpose and Goals

Just as a quick reminder, the purpose of today's session is to co-develop key message statements and provide feedback to the developed decision aid tool from the fragility fractures group that will help primary care providers and the general public understand who is at risk of developing a bone fracture and who needs to undergo subsequent screening procedures for fragility fractures. Fragility fractures result from low energy trauma and occur spontaneously such as falling from a standing height or less. Additionally, after today's deliberation session, you will be emailed an evaluation survey that will allow you to share your feedback with us regarding the community jury session process. The gathering of this feedback is vital to ensure that TF-PAN and the Task Force can continue to innovate and refine future programming initiatives.

Confidentiality and Consent to Record Audio

- Now I will talk about confidentiality. We take the issue of confidentiality seriously. No personal information about you will be shared with anyone outside of the study team. Your real name will not appear anywhere in the reports from today's session.

St. Michael's Inspired Care. Inspiring Science. UNITY HEALTH TORONTO

Appendix 7, as supplied by the authors. Appendix to: Thériault G, Limburg H, Klarenbach S, et al. Recommendations on screening for primary prevention of fragility fractures. *CMAJ* 2023. doi: 10.1503/cmaj.221219. Copyright © 2023 The Author(s) or their employer(s). To receive this resource in an accessible format, please contact us at cmajgroup@cmaj.ca.

- Any other information from today that could identify who you are will also be changed. For example, if you say, "in Toronto, where I live," we will replace that with something like "in the place where the participant lives".
- We strongly urge you to respect each other's privacy and not discuss what is said in the deliberation session with others. Also, please do not share the study materials with anyone outside of the study. The documents shared with you are not publicly available yet. Once the guideline recommendations are finalized, they will be emailed to you and posted to the Task Force website.
- We also want to encourage everyone to be respectful about each other's ideas, outlooks, and personal experiences during this session. It is important that we create an environment that supports non-judgmental and open communication.
- We are now ready to begin. If anyone is opposed to video recording in today's session, please let me know now. *Turn recorder on*
- The audio recorder is now on. Today's date is __________ and we are conducting the Task Force deliberation session on fragility fractures. There are __________ participants present in the session today.

-----------------------QUESTION PERIOD-------------------------------

Key Message Statements – Activity 1

So, will we begin with the first activity. The purpose of this activity is to develop key message statements for this upcoming fragility fractures guideline. Just to further clarify, key messages are the main points of information you want your audience to hear, understand, and remember. They are bite-sized summaries that articulate what you do, why you do it, how you are different, and what value it brings to the public. Key messages are important because they serve as the foundation of all communications and should be reflected in written and spoken communications. We want to ask you all for suggestions on what key messages would help the public understand the message and rationale of the recommendations. To help with discussion, here are some examples of potential key messages. *Key Messages Appear on Screen*

______ and I will divide you all into two smaller breakout groups. A prompt will appear on your screen, asking you to join the breakout group. ______ will be in one breakout group and I will be in the other. You will be given 30-45 minutes to work together to produce key message statements from the presentation. On the screen, you will see key message provided by the working groups, and you will all work together to create key messages of your own. We will type up the key messages on the word document as you create them.
I will now put you into breakout groups, please wait for a prompt to appear on your screen.

Create breakout room

So now that you have been divided into two groups, we will get started. Please take a few minutes to read the key messages provided by the Working Group. Reading this, what do you think some of the key messages are and why do you think they are important?

I want to emphasize that there is no need to wait for me to call on you to speak, feel free to jump in once

Appendix 7, as supplied by the authors. Appendix to: Thériault G, Limburg H, Klarenbach S, et al. Recommendations on screening for primary prevention of fragility fractures. *CMAJ* 2023. doi: 10.1503/cmaj.221219. Copyright © 2023 The Author(s) or their employer(s). To receive this resource in an accessible format, please contact us at cmajgroup@cmaj.ca.

the other person is done talking. I may call on people if the group is quiet or if the discussion is going very fast just to make sure that everyone has a chance to speak if they wish. I also want to emphasize that there is no right or wrong answers. Please feel free to ask any questions at any point during the activity or if there is anything that you may need clarification with.

Question Prompts to Ask During Session

- What do you think are the essential words or phrases here?
- Who do you think is the target audience? Who should be reading or hearing these messages?
- Why would the target audience care to know this information?

Notes:

- Observe if anyone is too quiet and not contributing
- Note the time limit
- Type key messages on the word document

Let's wrap up the first activity. I hope that went well for everyone. *Provide a quick summary of what was discussed*

After the community jury sessions, the task force fragility fractures working group will review the input given by the TF-PAN members from the community jury and ensure that this patient perspective is incorporated into the existing key message statements.

If you have any other questions or concerns, please feel free to ask now.

Break

Now would be a great time to a quick break. Feel free move around, stretch, get a quick snack, or take a water break. Please remember to turn off and mute your microphone during the break if you are moving away from your computer. We will meet back here in 10 minutes, and we will pick up where we left off from.

Welcome back everybody. I hope you guys had a great break and are ready to take on activity number 2.

Decision Aid Development – Activity 2

Welcome back everybody, I'll just do a quick scan to make sure everyone is back from the break. We are now going to be going into our second activity of the deliberation session.
The purpose of the second activity is to get your feedback on the fragility fracture's working group's bone fracture risk clinical decision aid tool that they have presented to you during the first community jury education session. The Mayo Clinic website https://osteoporosisdecisionaid.mayoclinic.org/ (present example) is an example of what we hope to develop for the Canadian decision aid.

Appendix 7, as supplied by the authors. Appendix to: Thériault G, Limburg H, Klarenbach S, et al. Recommendations on screening for primary prevention of fragility fractures. *CMAJ* 2023. doi: 10.1503/cmaj.221219. Copyright © 2023 The Author(s) or their employer(s). To receive this resource in an accessible format, please contact us at cmajgroup@cmaj.ca.

Within your breakout groups again, we will be exploring a discussion surrounding what you all think would be most helpful when considering to be screened or not screened for bone fracture risk, from a patient perspective.
Other additional themes we will be exploring regarding the tool are:

- language of tool (French and English)
- treatment plans and options of preventive health measures (drug treatment plans) built into the tool
- adding visuals regarding the harms that can be associated with this decision aid (over screening leading to over diagnosis)

Present the Mayo clinic example and I have some specific questions for the TF-PAN but would also like an open discussion of what they think would most help when considering whether to be screened

It looks like the structure would be:

1) Present the Mayo clinic example
2) Go through specific questions for TF-PAN members about the example tool (heather will provide)
3) Develop some questions to guide members in a discussion about what would most help when considering to be screened for bone fracture risk.

Let's begin with the questions!
Suggestions to present to the TF-PAN:

- Change to Canadian FRAX (Mayo Clinic is American)
- Translate into a French version?
- Add additional harms (e.g., overdiagnosis)
- Add visual for harms
- Add denosumab as a drug type of preventive measure to prvent bone loss (It works to **prevent bone loss** by blocking a certain receptor in the body to decrease bone breakdown)

St. Michael's Inspired Care. Inspiring Science.
UNITY HEALTH TORONTO
Appendix 7, as supplied by the authors. Appendix to: Thériault G, Limburg H, Klarenbach S, et al. Recommendations on screening for primary prevention of fragility fractures. *CMAJ* 2023. doi: 10.1503/cmaj.221219. Copyright © 2023 The Author(s) or their employer(s). To receive this resource in an accessible format, please contact us at cmajgroup@cmaj.ca.

Appendix 7, as supplied by the authors. Appendix to: Thériault G, Limburg H, Klarenbach S, et al. Recommendations on screening for primary prevention of fragility fractures. *CMAJ* 2023. doi: 10.1503/cmaj.221219. Copyright © 2023 The Author(s) or their employer(s). To receive this resource in an accessible format, please contact us at cmajgroup@cmaj.ca.
