# Supplementary material for: Recommendations on screening for primary prevention of fragility fractures
Source: CMAJ. 2023 May 8;195(18):E639–49. doi: 10.1503/cmaj.221219 (PMC10166624; doi:10.1503/cmaj.221219)
Supplement: Appendix 8: Cost-effectivenes Analyses of 2-step Screening via SCOOP RCT [file 221219-guide-8-at.pdf]

Appendix 8: Cost-effectiveness Analyses of 2-step Screening via SCOOP RCT

Summary of studies

Items derived from CHEERS Checklist (1) for reporting of economic evaluations.

**Item**	**Turner 2018** (2)	**Soreskog 2020** (3)
**Type of analysis**	CUA & CEA	
**Target population and subgroups**	Eligible females 70-85 years by post via primary care	Eligible females 70-85 years by post via primary care
**Setting and location**	UK	
**Study perspective**	UK National Health Service (NHS)	
**Comparators**	2-step screening with clinical FRAX (10-yr risk for hip fracture) and FRAX-BMD (age-dependent thresholds for BMD [FRAX 5.2–8.5%; n=3,064 49%] and for treatment [FRAX-BMD 5.24% and 8.99%; n=898 14% offered; total 15% in yr 1 &13-14% other yrs]) versus usual care (4% treated at 1 yr & 10% in last yr)	2-step screening with clinical FRAX (10-yr risk for hip fracture) and FRAX-BMD (age-dependent thresholds for BMD [FRAX 5.2–8.5%; n=3,064 49%] and for treatment [FRAX-BMD 5.24% and 8.99%; n=898 14% offered; total 15% in yr 1 &13-14% other yrs]) versus usual care (4% treated at 1 yr & 10% in last yr)
**Time horizon**	5 years	Lifetime (14 yrs)
**Discount rate**	3.5% annually after yr 1 (outcomes and costs)	3.5% annually (outcomes and costs)
**Choice of health outcomes**	Primary and secondary from RCT	From RCT separating hip and vertebral from any osteoporotic fracture
**Measurement of effectiveness**	Osteoporosis-related fracture prevented (hip, wrist and spine; RCT: HR, 0.94, p=0.178; same HR for clinical fractures)<br><br>Hip fracture prevented (RCT: HR, 0.72, p=0.002)<br><br>NHS Digital admitted patient care (inpatient), outpatient, and accident and emergency (A&E) datasets; primary care records	Life years<br><br>QALYs<br><br>Other osteoporotic fracture (pelvis, rib, humerus, tibia, clavicle, scapula, sternum and other femoral fractures) & hip fracture reductions over 5-years as per SCOOP

Appendix 8, as supplied by the authors. Appendix to: Thériault G, Limburg H, Klarenbach S, et al. Recommendations on screening for primary prevention of fragility fractures. *CMAJ* 2023. doi: 10.1503/cmaj.221219.
Copyright © 2023 The Author(s) or their employer(s). To receive this resource in an accessible format, please contact us at cmajgroup@cmaj.ca.

	screened for fractures based on their GP Read codes' participants could also self-report fractures at each follow-up (all fractures verified)	
**Measurement and valuation of preference-based outcomes**	Via RCT data collection: Quality adjusted life years (QALY) via 3-level EQ-5D; valued by tariffs using time-trade offs in general population of England, Scotland and Wales (1994) for outcomes at 10-yr duration (NR if using tariffs for >60 females)<br><br>Death = 0<br><br>Complete case analysis (CCA) & multiple imputation (across all yrs)	Did not use RCT EQ5D data.<br><br>For "well" state EQ-5D-3L was assumed to be equal to the age and gender matched general UK population<br><br>Wrist, hip, vertebral and other fractures were assumed to have an impact on quality of life during the first year after fracture (weights 0.82, 0.55, 0.68 and 0.86, respectively); hip and vertebral fractures were assumed to also have an impact on quality of life in subsequent years (weights 0.82 and 0.84, respectively), derived from the International Costs and Utilities Related to Osteoporotic fractures Study (ICUROS)<br><br>Annual mortality in the general female population was obtained from the Office for National Statistics dataset. The relative risks of death in patients who had sustained a fracture compared with the general population were derived from a study; 30% of the excess mortality after a hip, vertebral, wrist and other osteoporotic fracture was related to the fracture event (remaining related to concomitant diseases)
**Estimating resources and costs**	Screening resources: NHS Digital for BMD measurement via DXA scans, calculation and clinical review of final fracture risk, written notification of initial and final fracture risk, and a GP consultation for identified high fracture risk individuals (not initial FRAX)<br><br>Screening costs: part of study or using NHS Reference costs 2013 to 2014 or Unit costs of health and social care. Personal Social Services Research Unit; 2014. (average cost £104)<br><br>Fracture related healthcare: inpatient (type of admission [elective or non-elective]; length of stay; short stays; and excess bed days), outpatient (by specialty & first or follow-up appointments & procedure costs), and accident and emergency (A&E) datasets via HRG 4+ grouper to derive the HRG codes; linked to National Health Service (NHS)	As per Turner for screening, drugs (average for 2 years and a maximum of 5 years) and administration<br><br>Clinical costs in the first and subsequent years after fracture were derived from two retrospective cohort studies that estimated fracture costs in postmenopausal females in the UK; includes hospitalizations, nursing homes, outpatient (details not specified)

Appendix 8, as supplied by the authors. Appendix to: Thériault G, Limburg H, Klarenbach S, et al. Recommendations on screening for primary prevention of fragility fractures. *CMAJ* 2023. doi: 10.1503/cmaj.221219.
Copyright © 2023 The Author(s) or their employer(s). To receive this resource in an accessible format, please contact us at cmajgroup@cmaj.ca.

	reference costs; medications as used and costed British National Formulary	
**Currency, price date and conversion**	2013/14 pounds sterling	2013/14 for non-clinical costs; refs to clinical costs from 2011/12 (NR if converted)
**Choice of model**	No model	Markov model
**Assumptions**	None	Risk reductions for fracture are only for 5 yrs as per RCT (risk same as control thereafter) despite that many females would stay on treatment for >5 yrs<br><br>Not clearly stated:<br><br>Transitions between states as per cited work<br><br>QALYs using age and gender matched general UK population despite outcome data in different population
**Analytic methods & study parameters**	All outcome and resource data from SCOOP<br><br>Seemingly unrelated regression, allows for correlation between costs and outcomes and is generally considered robust for skewed data<br><br>Both costs and effects used baseline EQ-5D, age, and study group, as explanatory variables.	Markov model w/ six-month cycle length and the cohort was followed from study participation until death or an age of 100<br><br>8 health states: wrist fracture, vertebral fracture, hip fracture, other osteoporotic fracture, post-vertebral fracture, post-hip fracture, dead and well (i.e. without fracture).<br><br>Modelled fracture risk corresponded to the risk observed in SCOOP.<br><br>Probabilities of transitions NR; model was adapted based on previously published models of osteoporosis interventions
**Incremental costs and outcomes**	<u>Base case</u>	Hip fractures: 9 fewer per 1000

Appendix 8, as supplied by the authors. Appendix to: Thériault G, Limburg H, Klarenbach S, et al. Recommendations on screening for primary prevention of fragility fractures. *CMAJ* 2023. doi: 10.1503/cmaj.221219.
Copyright © 2023 The Author(s) or their employer(s). To receive this resource in an accessible format, please contact us at cmajgroup@cmaj.ca.

	QALYs: 0.0237 per person (95% CI: -0.003 to 0.051) (adjusted for age and baseline EQ5D)<br><br>incremental cost-effectiveness ratio (ICER) £2,772/QALY<br><br>CEACs: 93% probability the intervention is cost-effective at the NICE threshold of £20,000 per QALY.<br><br>Osteoporotic-related fractures prevented: 0.0146 (95% CI: 0.0002 to 0.029); £4,478 per osteoporotic-related fracture<br><br>Hip fractures prevented: 0.0085 (95% CI: 0.0026 to 0.0144); £7,694 per hip fracture prevented; 87% probability of CE	Non-hip fractures: 20 fewer per 1000<br><br>QALYs/patient: 0.015 gained<br><br>Life years 0.002 gained<br><br>Costs saved £286/patient (fracture related costs £551 lower; drug and intervention cost £265 higher)
**Characterizing uncertainty**	CCA for QALYs (64% study population); missing cases had statistically significantly lower baseline EQ-5D, more incident fractures and higher fracture related healthcare costs<br><br>CCA (w/ fewer fractures)<br><br>QALYs: 0.0214 (95% CI: -0.011 to 0.054); £4,646/QALY; 83% probability<br><br>Osteoporotic-related fractures prevented 0.0094 (95% CI: -0.0073 to 0.026); £10,564	Probabilistic sensitivity analysis (PSA); 97% probability of cost-effective at WTP £20,000 and 98% at £30,000; cost-saving in 87% of simulations<br><br>Deterministic sensitivity analysis was conducted: assuming that screening had an effect only on hip fractures, changing the discount rate, modelling time horizon (10 year), age, and assuming that 100% of the excess mortality of fracture was related to the fracture event<br><br>Only hip fracture reduction: £241 saved & 0.011 QALYs gained<br><br>Other analyses all cost-saving, except for at age 71 where became cost-neutral.

Appendix 8, as supplied by the authors. Appendix to: Thériault G, Limburg H, Klarenbach S, et al. Recommendations on screening for primary prevention of fragility fractures. *CMAJ* 2023. doi: 10.1503/cmaj.221219.
Copyright © 2023 The Author(s) or their employer(s). To receive this resource in an accessible format, please contact us at cmajgroup@cmaj.ca.

	Hip fractures prevented 0.0045 (95% CI: -0.0018 to 0.0108); £22,067<br><br>None for uncertainty in RRR in fractures<br><br>Protocol stated SF-6D used for sensitivity but NR here	None for uncertainty in RRR in fractures or for QALYs from general populations
**Characterizing heterogeneity**	Adjusted for baseline EQ5D and age	As above
**Discussion/limitations**	• Not costed for routine primary care contacts (for monitoring meds etc.) and admissions to nursing homes (may underestimate savings from fewer fractures; # NR in publications)<br>• Missing EQ5D data possible for worse cases if fractured during collection; collected q 6-12 mos so <u>acute changes from fractures not captured</u><br>• Length of follow-up; Kanis et al investigated the effect on ICERs of a 10-yr compared to lifetime follow-up for 70 year old females. Increasing the length of follow-up led to a decrease in estimated ICERs (i.e. improved cost-effectiveness).<br>• Estimates may be conservative from healthier sample (50% fewer deaths, more educated, higher SES) and some costs related to RCT (£44/enrolled for identifying pts)	Model is a hierarchical structure that causes a slight underestimation of the number of less severe fractures, as patients suffering a hip or vertebral fracture cannot subsequently sustain wrist or other fractures in following cycles (i.e. remain in post hip/vertebral state)<br><br>Only used risk reductions for 5 yrs and effects may be seen after longer treatment or even after treatment discontinuation
**Source of funding**	Arthritis Research United Kingdom (ARUK), formerly the Arthritis Research Campaign (ARC), and the Medical Research Council (MRC) of the UK, jointly funded this trial.	As for RCT; NR funding for modeling study
**Conflicts of interest**	Several authors have funding by industry for other work	Several authors have funding by industry for other work

Appendix 8, as supplied by the authors. Appendix to: Thériault G, Limburg H, Klarenbach S, et al. Recommendations on screening for primary prevention of fragility fractures. *CMAJ* 2023. doi: 10.1503/cmaj.221219.
Copyright © 2023 The Author(s) or their employer(s). To receive this resource in an accessible format, please contact us at cmajgroup@cmaj.ca.

Reporting quality

Y=yes; P=partial (e.g., no rationale provided); N=No; NA=not applicable (e.g., not reported in published manuscript)

Section/Item	Item no.	Recommendation	Turner 2018 (2)	Söreskog 2020 (3)
**Title and abstract**				
Title		Identify the study as an economic evaluation or use more specific terms such as "cost-effectiveness analysis", and describe the interventions compared.	Y	Y
Abstract		Provide a structured summary of objectives, perspective, setting, methods (including study design and inputs), results (including base case and uncertainty analyses), and conclusions.	P; nothing about complete case analysis	P; does not specify perspective or uncertainty analysis
**Introduction**				
Background and objectives		Provide an explicit statement of the broader context for the study.<br><br>Present the study question and its relevance for health policy or practice decisions.	Y	Y
**Methods**				
Target population and subgroups		Describe characteristics of the base case population and subgroups analysed, including why they were chosen.	Y (especially since RCT published); describe subgroup with missing EQ5D data & rationale	Y

Appendix 8, as supplied by the authors. Appendix to: Thériault G, Limburg H, Klarenbach S, et al. Recommendations on screening for primary prevention of fragility fractures. *CMAJ* 2023. doi: 10.1503/cmaj.221219.
Copyright © 2023 The Author(s) or their employer(s). To receive this resource in an accessible format, please contact us at cmajgroup@cmaj.ca.

Setting and location		State relevant aspects of the system(s) in which the decision(s) need(s) to be made.		
Study perspective		Describe the perspective of the study and relate this to the costs being evaluated.	Y	N
Comparators		Describe the interventions or strategies being compared and state why they were chosen.	Y	Y
Time horizon		State the time horizon(s) over which costs and consequences are being evaluated and say why appropriate.	Y	Y
Discount rate		Report the choice of discount rate(s) used for costs and outcomes and say why appropriate.	Y (NICE guidance)	Y (NICE guidance)
Choice of health outcomes		Describe what outcomes were used as the measure(s) of benefit in the evaluation and their relevance for the type of analysis performed.	Y	Y
Measurement of effectiveness	a<br><br>b	*Single study-based estimates:* Describe fully the design features of the single effectiveness study and why the single study was a sufficient source of clinical effectiveness data.<br><br>*Synthesis-based estimates:* Describe fully the methods used for identification of included studies and synthesis of clinical effectiveness data.	*P: nothing about differences between SCOOP and other studies (but not published at that time)*	*P: nothing about chosen cohorts for utility data*
Measurement and valuation of preference based outcomes		If applicable, describe the population and methods used to elicit preferences for outcomes.	Y	P; cited but no details reported
Estimating resources and costs	a	*Single study-based economic evaluation:* Describe approaches used to estimate resource use associated with the alternative interventions. Describe primary or secondary research methods for valuing each resource item in terms of its unit cost.	*Y*	P; cited but no details reported e.g. type of hospitalization data

Appendix 8, as supplied by the authors. Appendix to: Thériault G, Limburg H, Klarenbach S, et al. Recommendations on screening for primary prevention of fragility fractures. *CMAJ* 2023. doi: 10.1503/cmaj.221219.
Copyright © 2023 The Author(s) or their employer(s). To receive this resource in an accessible format, please contact us at cmajgroup@cmaj.ca.

	b	Describe any adjustments made to approximate to opportunity costs.<br><br>*Model-based economic evaluation:* Describe approaches and data sources used to estimate resource use associated with model health states. Describe primary or secondary research methods for valuing each resource item in terms of its unit cost. Describe any adjustments made to approximate to opportunity costs		
Currency, price date, and conversion		Report the dates of the estimated resource quantities and unit costs. Describe methods for adjusting estimated unit costs to the year of reported costs if necessary. Describe methods for converting costs into a common currency base and the exchange rate.	Y; UK values reported but could have converted to 2018	P; UK sources reported with dates but NR if any conversion
Choice of model		Describe and give reasons for the specific type of decision-analytical model used. Providing a figure to show model structure is strongly recommended.	NA	P; no transition probabilities reported
Assumptions		Describe all structural or other assumptions underpinning the decision-analytical model.	NA	P
Analytical methods		Describe all analytical methods supporting the evaluation. This could include methods for dealing with skewed, missing, or censored data; extrapolation methods; methods for pooling data; approaches to validate or make adjustments (such as half cycle corrections) to a model; and methods for handling population heterogeneity and uncertainty.	Y	Y (used valid model)
**Results**				
Study parameters		Report the values, ranges, references, and, if used, probability distributions for all parameters. Report reasons or	Y	Y

Appendix 8, as supplied by the authors. Appendix to: Thériault G, Limburg H, Klarenbach S, et al. Recommendations on screening for primary prevention of fragility fractures. *CMAJ* 2023. doi: 10.1503/cmaj.221219.
Copyright © 2023 The Author(s) or their employer(s). To receive this resource in an accessible format, please contact us at cmajgroup@cmaj.ca.

		sources for distributions used to represent uncertainty where appropriate.<br><br>Providing a table to show the input values is strongly recommended.		
Incremental costs and outcomes		For each intervention, report mean values for the main categories of estimated costs and outcomes of interest, as well as mean differences between the comparator groups. If applicable, report incremental cost-effectiveness ratios.	Y	Y
Characterizing uncertainty		*Single study-based economic evaluation:* Describe the effects of sampling uncertainty for the estimated incremental cost and incremental effectiveness parameters, together with the impact of methodological assumptions (such as discount rate, study perspective).<br><br>*Model-based economic evaluation:* Describe the effects on the results of uncertainty for all input parameters, and uncertainty related to the structure of the model and assumptions.	*P for EQ5D only*	*P*
Characterizing heterogeneity		If applicable, report differences in costs, outcomes, or cost-effectiveness that can be explained by variations between subgroups of patients with different baseline characteristics or other observed variability in effects that are not reducible by more information.	N	P age only
**Discussion**				
Study findings, limitations, generalizability, and current knowledge		Summaries key study findings and describe how they support the conclusions reached. Discuss limitations and the generalizability of the findings and how the findings fit with current knowledge.	Y	Y
**Other**				

Appendix 8, as supplied by the authors. Appendix to: Thériault G, Limburg H, Klarenbach S, et al. Recommendations on screening for primary prevention of fragility fractures. *CMAJ* 2023. doi: 10.1503/cmaj.221219.
Copyright © 2023 The Author(s) or their employer(s). To receive this resource in an accessible format, please contact us at cmajgroup@cmaj.ca.

Source of funding		Describe how the study was funded and the role of the funder in the identification, design, conduct, and reporting of the analysis. Describe other non-monetary sources of support.	Y	P assuming RCT funders did not fund all modelling
Conflicts of interest		Describe any potential for conflict of interest of study contributors in accordance with journal policy. In the absence of a journal policy, we recommend authors comply with International Committee of Medical Journal Editors recommendations.	Y	Y

Appendix 8, as supplied by the authors. Appendix to: Thériault G, Limburg H, Klarenbach S, et al. Recommendations on screening for primary prevention of fragility fractures. *CMAJ* 2023. doi: 10.1503/cmaj.221219.
Copyright © 2023 The Author(s) or their employer(s). To receive this resource in an accessible format, please contact us at cmajgroup@cmaj.ca.

Cost analysis from the Evidence to Decision Framework

**How large are the resource requirements (costs)?**<br><br>○Large costs<br>○Moderate costs<br>○Negligible costs and savings<br>**X** Moderate savings<br>○Large savings<br><br>○Varies<br>○Don't know	A systematic cost-effectiveness analysis was not conducted as part of the systematic review.<br><br>**JUDGEMENT – RESOURCES REQUIRED**<br><br>There would be moderate cost savings with a strong recommendation against screening for men and younger females. Canadian data (2009) indicates that 35% of females 40-64 years and 10% of males ≥40 years (20% of males ≥65 years) self-reported receiving BMD scans (10). Additionally, there should be savings if the Task Force recommends for two-step screening among females ≥65 years (e.g. some BMD scans could be avoided based on FRAX score and shared decision making).<br><br>There may be additional costs if recommending for screening females ≥65 years as Canadian self-reported data (2009) found that 32% of females in this age group had never received a BMD test (10). However, as a conditional recommendation this would depend on the results of shared decision making.<br><br>A cost-effectiveness analysis of the SCOOP trial (5 year follow-up) showed that screening prevented fractures at a cost of £4,478 and £7,694 per fracture for MOF and hip fractures, respectively. It also improved QALY at an average incremental cost of £2,772 (2).<br><br>A Markov model of the SCOOP trial estimated long-term (mean=14 year) outcomes for screened vs unscreened individuals. Screening of 1,000 patients saved 9 hip fractures and 20 non-hip fractures. The screening arm also saved £286 in comparison with usual management arm (3).	Estimated cost for BMD (DEXA) in Ontario (4): (Billing schedule) $47.75 for one site<br>$61.55 for two sites (hip and spine)<br>Associated costs<br>- Radiation technologist<br>- Radiologist<br>- Family doctor<br>- Medication<br><br>There may be significant patient costs of medication as some provincial drug coverage only provides restricted access to certain medications (e.g. denosumab, zoledronic acid).<br>Canada >=65 years medication coverage (5,6)<br>Alendronate (CAD$122-$182/ year) or Risedronate (CAD$130-$600 /year): Coverage varies by province (open access or restricted access)

Appendix 8, as supplied by the authors. Appendix to: Thériault G, Limburg H, Klarenbach S, et al. Recommendations on screening for primary prevention of fragility fractures. *CMAJ* 2023. doi: 10.1503/cmaj.221219.
Copyright © 2023 The Author(s) or their employer(s). To receive this resource in an accessible format, please contact us at cmajgroup@cmaj.ca.

<table>
<tr>
<td></td>
<td>

<u>COST-EFFECTIVENESS ANALYSES OF THE SCOOP STUDY:</u>

1. Turner DA, Khioe RFS, Shepstone L, Lenaghan E, Cooper C, Gittoes N, et al. The Cost-Effectiveness of Screening in the Community to Reduce Osteoporotic Fractures in Older Women in the UK: Economic Evaluation of the SCOOP Study. J Bone Miner Res 2018 May;33(5):845-851. (2)
<u>Perspective:</u> A “within trial” economic analysis was undertaken on an “intention-to-treat” basis from the perspective of a national health payer, the UK National Health Service (NHS).
<u>Methods</u>: **Five-year time horizon** for cost (2013/14) per quality adjusted life year (QALY), osteoporosis-related fracture prevented (hip, vertebral, wrist), and hip fracture prevented. Point estimates from RCT for hip and osteoporotic fractures used. QALYs estimated (from AUC) from EQ-5D scores across all data points (at baseline, 6, 12 and annually; <u>major imputation requited for 36% participants</u>); tariffs from UK population from 10-year duration TTOs. Screening resources (BMD/DXA scans, calculation and clinical review of fracture risk, GP consultation; identifying women) were recorded as part of the SCOOP study and costed per study data or using NHS Reference costs 2013 to 2014 or unit costs of health and social care 2014. Resources and costs associated with fracture-related health care contacts (inpatient [elective or non-elective]; length of stay; short stays; and excess bed days), outpatient [by specialty & first or follow-up appointments & procedure costs], and accident and emergency (A&E) datasets using Health Resource Group codes were linked to NHS reference costs. Medication data were available for anti-osteoporosis medicines for the full period of follow-up for all study participants and were costed using prices from the British National Formulary. Sensitivity analysis using complete case analysis with patients completing all EQ5D data.
<u>Summary:</u> The screening arm had an average incremental **QALY gain of 0.0237** (95% CI -0.0034 to 0.00508) for the 5-year follow-up. The **cost per QALY gained was £2,772**. Cost-effectiveness acceptability curves indicated a 93% probability of the intervention being cost-effective at a threshold cost/QALY of ≤£20,000. The intervention arm prevented fractures at a cost of **£4,478 and £7,694 per fracture for osteoporosis-related hip fractures**, respectively. Complete case analysis had 2-3 times higher cost/QALYs (ICERs).

Table 8.1 Cost-effectiveness results for cases vs controls in SCOOP (130)

<table>
<tr><th></th><th>Usual management</th><th>Screening</th></tr>
<tr><td colspan="3">Mean costs, per patient (£)</td></tr>
<tr><td>Inpatient</td><td>531</td><td>482</td></tr>
<tr><td>A & E</td><td>162</td><td>160</td></tr>
</table>

</td>
<td>

<u>Zoledronic acid</u> (CAD$335/year): Coverage varies by province (restricted access or no coverage)
<u>Denosumab</u> (CAD$716/year) (restricted access in all provinces)
(5,6)

In Ontario, the total cost of treatment for all hip fractures occurring in 2015/16 (in adults aged 66+) was estimated to be $255,773,130 based on direct utilization costs for the episode of care. The median cost per single episode of care was $25,015 for direct utilization costs (7).

Utilization of BMD varies by sex with 8.15% of females vs 4.81% of males aged 40+ reporting a BMD scan in 2015 (8).

22.9% of eligible adults (aged 68-70) in

</td>
</tr>
</table>

Appendix 8, as supplied by the authors. Appendix to: Thériault G, Limburg H, Klarenbach S, et al. Recommendations on screening for primary prevention of fragility fractures. *CMAJ* 2023. doi: 10.1503/cmaj.221219.
Copyright © 2023 The Author(s) or their employer(s). To receive this resource in an accessible format, please contact us at cmajgroup@cmaj.ca.

<table>
<tr>
<td></td>
<td>
<table>
<tr><td>Outpatient</td><td>191</td><td>201</td></tr>
<tr><td>Medicines</td><td>8</td><td>13</td></tr>
<tr><td>Non-SCOOP DXA</td><td>9</td><td>9</td></tr>
<tr><td>Cost of SCOOP interventions</td><td>-</td><td>104</td></tr>
<tr><td>Total costs</td><td>900</td><td>968</td></tr>
</table>

2. Söreskog E, Borgström F, Shepstone L, Clarke S, Cooper C, Harvey I, et al. Long-term cost-effectiveness of screening for fracture risk in a UK primary care setting: the SCOOP study. Osteoporos Int 2020 Aug;31(8):1499-1506. (3)
<u>Perspective</u>: Cost-effectiveness based on the NICE’s willingness-to-pay (WTP) threshold for recommending new treatment of £20,000–30,000 per QALY gained
<u>Methods:</u> Health economic Markov model (6-month cycles and 8 health states: wrist fracture, vertebral fracture, hip fracture, other osteoporotic fracture, post-vertebral fracture, post-hip fracture, dead and well [i.e. without fracture]; transition probabilities NR but cited studies) following a cohort from study participation until death or an age of 100 years (mean 14 year time horizon). Outcomes were cost per quality adjusted life year (QALY) and life year. Point estimates from RCT for various discrete fractures used (risk reductions at 5 years were used without any assumptions of longer term effects). Resource use and costs for drugs, administration, screening intervention as per above analysis from SCOOP RCT. Clinical costs (hospitalizations, nursing homes, outpatient) in the first and subsequent years after fracture were derived from two retrospective cohort studies that estimated fracture costs in postmenopausal females in the UK. Quality of life weights for each health state, in the first year after fracture and subsequent years (for hip and vertebral), respectively, were derived from the International Costs and Utilities Related to Osteoporotic fractures Study (ICUROS). Annual mortality in the general female population was obtained from the Office for National Statistics dataset. The relative risks of death in patients who had sustained a fracture compared with the general population were derived from a study by Jönsson et al. In agreement with previous health economic studies of osteoporotic treatments it was assumed that 30% of the excess mortality after a hip, vertebral, wrist and other osteoporotic fracture was related to the fracture event. Probabilistic and deterministic sensitivity analyses including if no effect on non-hip fractures and age.
<u>Summary:</u> Screening of 1000 patients saved 9 hip fractures and 20 non-hip fractures over the remaining lifetime (mean 14 years) compared with usual management. Per patient, the screening arm saved £286 and gained 0.015 QALYs and 0.002 life years in comparison with the usual management arm. 97% probability of cost-effective at WTP
</td>
<td>Ontario reported ever being screened in 2017/18 (7).

13.4% of BMD scans in Ontario were performed on “low risk” adults aged ≥40 years in 2017/18 (7).</td>
</tr>
</table>

Appendix 8, as supplied by the authors. Appendix to: Thériault G, Limburg H, Klarenbach S, et al. Recommendations on screening for primary prevention of fragility fractures. *CMAJ* 2023. doi: 10.1503/cmaj.221219.
Copyright © 2023 The Author(s) or their employer(s). To receive this resource in an accessible format, please contact us at cmajgroup@cmaj.ca.

£20,000 and 98% at £30,000; cost-saving in 87% of simulations. Deterministic analyses all indicated cost-savings, except for at age 71 where screening became cost-neutral.

Table 8.2 Long-term cost-effectiveness results (Markov model) (3)

	Usual management	Screening	Screening vs. usual management
Mean costs, per patient (£)			
Hospitalisations	3059	2934	− 125
Nursing home	6056	5645	− 410
Outpatient	378	363	− 15
Total morbidity cost	9493	8942	− 551
Drugs	12	43	31
Treatment management	92	326	234
Total intervention cost	104	369	265
**Total cost**	**9596**	**9310**	**−286**

Appendix 8, as supplied by the authors. Appendix to: Thériault G, Limburg H, Klarenbach S, et al. Recommendations on screening for primary prevention of fragility fractures. *CMAJ* 2023. doi: 10.1503/cmaj.221219.
Copyright © 2023 The Author(s) or their employer(s). To receive this resource in an accessible format, please contact us at cmajgroup@cmaj.ca.

**What is the certainty of the evidence of resource requirements (costs)?**<br><br>○ Very low<br>**X** Low<br>○ Moderate<br>○High<br><br>○No included studies	A systematic cost-effectiveness analysis was not conducted as part of the systematic review.<br><br>**JUDGEMENT – CERTAINTY OF EVIDENCE OF RESOURCE REQUIREMENTS**<br><br>**There are serious limitations due to indirectness and risk of bias with the Turner et al., 2018 study.**<br><br>**There are serious concerns due to indirectness and some concerns with risk of bias with the Soreskog et al., 2020 study.**<br><br>**Overall for selected population, there is low certainty for being highly cost-effective (ICERs low) or cost-saving to the healthcare system (indirectness and risk of bias), but moderate certainty for likely meeting typical cost-effectiveness thresholds.**<br><br>1. The Cost-Effectiveness of Screening in the Community to Reduce Osteoporotic Fractures in Older Females in the UK: Economic Evaluation of the SCOOP Study (2)<br><br>Limitations:<br>• No sensitivity for uncertainty in hip fracture reductions (e.g. 95% CI) or lack of any effect on other fractures<br>• Large amount of missing EQ5D data. Missing cases had statistically significantly lower baseline EQ-5D, more incident fractures and higher fracture related healthcare costs. More missing EQ5D data possible for worse cases if fractured during data collection. Data collected q 6-12 months so acute changes from fractures not captured. Complete case analysis had 2-3 times higher cost/QALYs.<br>• Short time horizon and lack of effects from fractures on longer term and/or more reductions in fractures that may occur<br>• Estimates may be conservative from healthier sample (50% fewer deaths, more educated, higher SES) and some costs related to RCT (£44/enrolled for identifying pts)<br>• No patient or societal costs (e.g. family carers) accounted for (if desiring societal perspective).	Note: The cost-effectiveness threshold suggested by CADTH is $50,000 (9).

Appendix 8, as supplied by the authors. Appendix to: Thériault G, Limburg H, Klarenbach S, et al. Recommendations on screening for primary prevention of fragility fractures. *CMAJ* 2023. doi: 10.1503/cmaj.221219.
Copyright © 2023 The Author(s) or their employer(s). To receive this resource in an accessible format, please contact us at cmajgroup@cmaj.ca.

	• Not costed for routine primary care contacts (e.g. for monitoring patients on meds or treating AEs) or admissions to nursing homes (may underestimate savings from fewer fractures; # admissions NR in publications)<br>Serious indirectness: Use of all vertebral fractures, but little effect in this study. Recruitment methods and education of GPs not typical but effect on the outcomes is unclear. Short-term time horizon. Does not account for possible larger reduction in non-hip fractures seen in other RCTs and our meta-analysis (which may improve cost-effectiveness).<br>Serious ROB from large missing data for utilities (for ICER on QALYs), not full spectrum of costs accounted for, and contamination of control groups possibly impacting risk reductions.<br><br>Note: Indirect to use of clinical FRAX only (number treated may differ) or using FRAX+BMD treatment thresholds that are not age dependent.<br><br>2. Söreskog E, Borgström F, Shepstone L, Clarke S, Cooper C, Harvey I, et al. Long-term cost-effectiveness of screening for fracture risk in a UK primary care setting: the SCOOP study. Osteoporos Int 2020 Aug;31(8):1499-1506. (3)<br><br>Limitations:<br><br>• QALYs using age and gender matched general UK population despite outcome data in a different population. Assuming one consistent utility score for all non-fractured states and minimum 6-month period for fracture states (some utilities may change before this).<br>• Quality of data used for transition probabilities unknown ("valid model").<br>• No sensitivity for uncertainty in hip fracture reductions.<br>• Not costed for routine primary care contacts (e.g. for monitoring patients on meds or treating AEs)<br>• No patient or societal costs (e.g. family carers) accounted for.<br><br>Serious indirectness: Indirect sources for utilities and transitions. Indirect from use of all vertebral fractures, but effects minimal. Recruitment methods and education of GPs not typical but effect on the outcomes is unclear. Does not account for possible larger reduction in non-hip fractures seen in other RCTs and our meta-analysis (which may improve cost-effectiveness).	

Appendix 8, as supplied by the authors. Appendix to: Thériault G, Limburg H, Klarenbach S, et al. Recommendations on screening for primary prevention of fragility fractures. *CMAJ* 2023. doi: 10.1503/cmaj.221219.
Copyright © 2023 The Author(s) or their employer(s). To receive this resource in an accessible format, please contact us at cmajgroup@cmaj.ca.

	Some concern about ROB: from not full spectrum of costs accounted for, and contamination of control groups possibly impacting risk reductions.<br><br>Studies quite consistent, but overlap in data so not too surprising.<br><br>**Overall for selected population, would rate at low certainty for being highly cost-effective (ICERs low) or cost-saving to the healthcare system (indirectness and risk of bias), but moderate for likely meeting typical CE thresholds.**<br><br>Please see Cost-effectiveness analysis section for the full assessment	
**Does the cost-effectiveness of the intervention favor the intervention or the comparison?**<br><br>○Favors the comparison<br>○Probably favors the comparison<br>○Does not favor either the intervention or the comparison<br>**X** Probably favors the intervention<br>○Favors the intervention	**JUDGEMENT – COST-EFFECTIVENESS**<br><br>**Based on the two studies of SCOOP, it appears to favour screening to prevent fragility fractures (cost-saving to the health-care system). However, the certainty for this conclusion is low and is based only on one RCT.**<br><br>**When considering whether the cost to the healthcare system would meet typical cost-effectiveness thresholds the certainty was moderate.**	

Appendix 8, as supplied by the authors. Appendix to: Thériault G, Limburg H, Klarenbach S, et al. Recommendations on screening for primary prevention of fragility fractures. *CMAJ* 2023. doi: 10.1503/cmaj.221219.
Copyright © 2023 The Author(s) or their employer(s). To receive this resource in an accessible format, please contact us at cmajgroup@cmaj.ca.

○Varies<br>○No included studies		

References

1. Husereau D, Drummond M, Petrou S, Carswell C, Moher D, Greenberg D, et al. Consolidated Health Economic Evaluation Reporting Standards (CHEERS) statement. BMJ. 2013 Mar;346:f1049.

2. Turner DA, Khioe RFS, Shepstone L, Lenaghan E, Cooper C, Gittoes N, et al. The Cost-Effectiveness of Screening in the Community to Reduce Osteoporotic Fractures in Older Women in the UK: Economic Evaluation of the SCOOP Study. J Bone Miner Res. 2018;33(5).

3. Söreskog E, Borgström F, Shepstone L, Clarke S, Cooper C, Harvey I, et al. Long-term cost-effectiveness of screening for fracture risk in a UK primary care setting: the SCOOP study. Osteoporos Int. 2020;31(8).

4. The Ministry of Health and Long-Term Care (Ontario). Schedule of Facility Fees for Independent Health Facilities Under the Independent Health Facilities Act. Vol. 2017. 2015.

5. Osteoporosis Canada. Osteoporosis Canada. Provincial Drug Coverage. 2017.

6. CADTH. Common Drug Review: Denosumab (Prolia - Amgen Canada) Indication: Osteoporosis in Men [Internet]. 2015 [cited 2021 Mar 26]. Available from: https://www.cadth.ca/sites/default/files/cdr/complete/SR0414_cdr_complete_Prolia-Men_Sept-21-15-e.pdf

7. Jaglal S, Cameron C, Croxford R, MacKay C, Yasmin F. Ontario Osteoporosis Strategy - Provincial Performance Data for Osteoporosis Management Technical Report [Internet]. 2020 [cited 2021 Dec 6]. Available from: https://osteostrategy.on.ca/wp-content/uploads/Final-OOS-Provincial-Performance-Data-Technical-Report-Nov-18-20.pdf

Appendix 8, as supplied by the authors. Appendix to: Thériault G, Limburg H, Klarenbach S, et al. Recommendations on screening for primary prevention of fragility fractures. *CMAJ* 2023. doi: 10.1503/cmaj.221219.
Copyright © 2023 The Author(s) or their employer(s). To receive this resource in an accessible format, please contact us at cmajgroup@cmaj.ca.

8. Public Health Agency of Canada. Canadian Chronic Disease Surveillance System (CCDSS) [Internet]. Vol. 2021. 2019 [cited 2021 Feb 22]. Available from: https://health-infobase.canada.ca/ccdss/data-tool/Age?V=32&M=3&S=B&Y=2016

9. CADTH. Guidelines for the Economic Evaluation of Health Technologies: Canada 4th Edition. Vol. 2021. 2017.

10. Statistics Canada. Canadian Community Health Survey 2009 [Internet]. 2012 [cited 2021 Jul 16]. Available from: https://www23.statcan.gc.ca/imdb/p3Instr.pl?Function=assembleInstr&a=1&&lang=en&Item_Id=76867

Appendix 8, as supplied by the authors. Appendix to: Thériault G, Limburg H, Klarenbach S, et al. Recommendations on screening for primary prevention of fragility fractures. *CMAJ* 2023. doi: 10.1503/cmaj.221219.
Copyright © 2023 The Author(s) or their employer(s). To receive this resource in an accessible format, please contact us at cmajgroup@cmaj.ca.
