# Supplementary material for: Recommendations on screening for primary prevention of fragility fractures
Source: CMAJ. 2023 May 8;195(18):E639–49. doi: 10.1503/cmaj.221219 (PMC10166624; doi:10.1503/cmaj.221219)
Supplement: Appendix 9: Analytical Framework [file 221219-guide-9-at.pdf]

Appendix 9: Analytical Framework

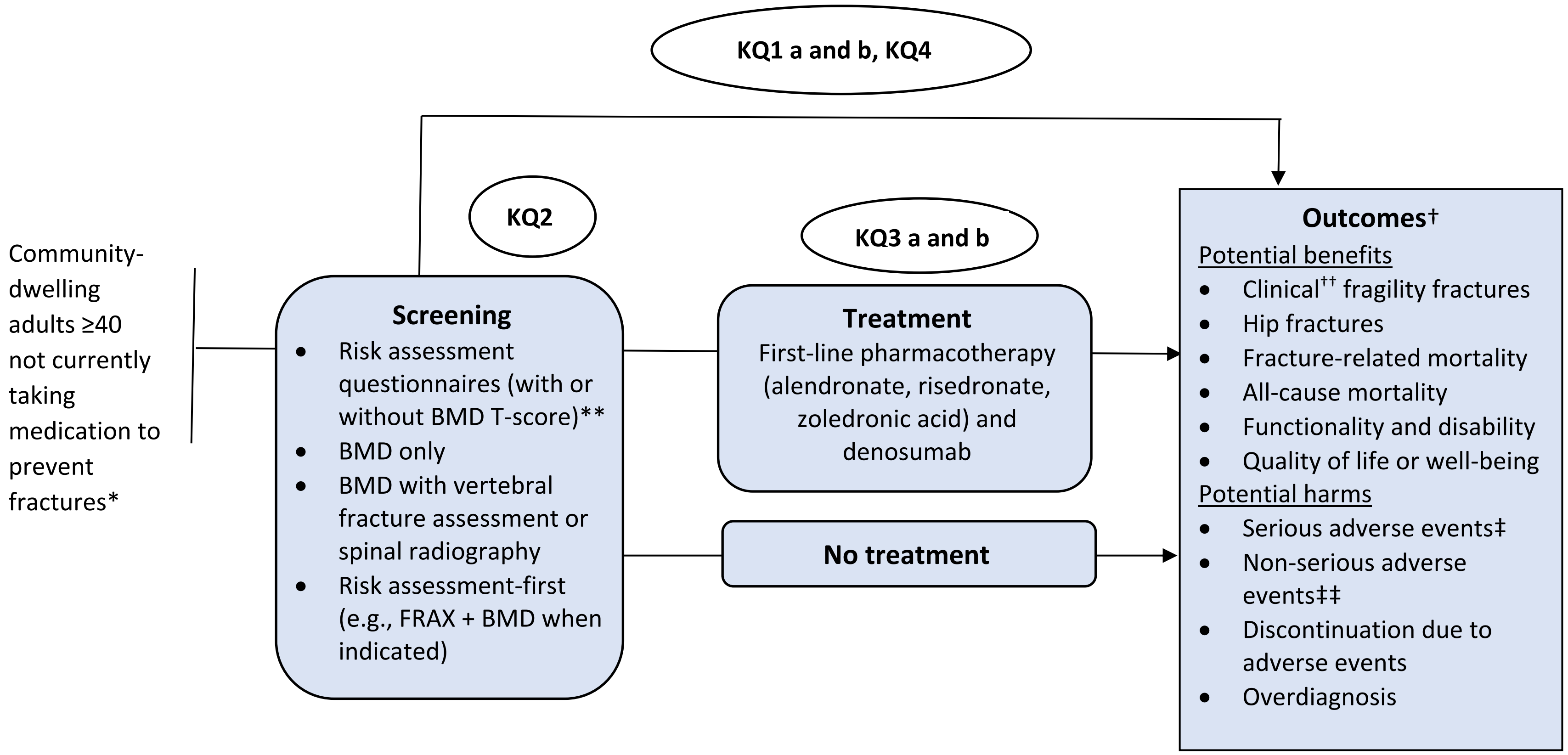

Abbreviations: BMD, bone mineral density test; FRAX, Fracture Risk Assessment Tool (https://frax.shef.ac.uk/); KQ, key question; T-score, standard deviations above or below the average bone density.

Appendix 9, as supplied by the authors. Appendix to: Thériault G, Limburg H, Klarenbach S, et al. Recommendations on screening for primary prevention of fragility fractures. *CMAJ* 2023. doi: 10.1503/cmaj.221219. Copyright © 2023 The Author(s) or their employer(s). To receive this resource in an accessible format, please contact us at cmajgroup@cmaj.ca.

Analytical framework: Key question (KQ) 1a: What are the benefits and harms of screening compared with no screening to prevent fragility fractures and related morbidity and mortality in primary care for adults ≥ 40 years? KQ1b: Does the effectiveness of screening to prevent fragility fractures vary by screening program type (i.e., BMD-first vs risk assessment-first) or by risk assessment tool? KQ2: How accurate are screening tests at predicting fracture risk among adults ≥ 40 years? KQ3a: What are the benefits of pharmacologic treatments to prevent fragility fractures among adults ≥ 40 years? KQ3b: What are the harms of pharmacologic treatments to prevent fragility fractures among adults ≥ 40 years? KQ4: For patients ≥40 years, what is the acceptability (i.e., positive attitudes, intentions, willingness, uptake) of screening and/or initiating treatment to prevent fragility fractures when considering the possible benefits and harms from screening and/or treatment?

* Main target population for guideline; inclusion and exclusion criteria for studies differ somewhat and are described in the protocol and systematic review (1,2).
** Any paper or electronic tool or set of questions using ≥ 2 demographic and/or clinical factors to assess risk for future fracture; must be externally validated for KQ2.
†These were all rated as critical or important by the Task Force, after considering input on their relative importance by patients, using surveys and focus groups conducted by the Knowledge Translation team at St. Michael's Hospital (Toronto). All benefits are considered critical (rated as ≥ 7 on 9-point scale) except for all-cause mortality which was important (4–6 on 9-point scale); for harms, serious adverse events are critical while the others are important. We acknowledge that some outcomes, should the direction of effect be the opposite of intended, may be considered harms versus benefits, and vice versa.
††Any symptomatic and radiologically confirmed fracture (sites per author definition; may be defined as major osteoporotic fracture).
‡The primary outcome will be total count of any serious adverse event, but individual outcomes of (a) serious cardiovascular, (b) serious cardiac rhythm disturbances, (c) serious gastrointestinal events (except cancers), (d) gastrointestinal cancers (i.e., colon, colorectal, gastric, esophageal), (e) atypical fractures, and (f) osteonecrosis of the jaw will also be included.
‡‡ Count of total number of participants experiencing one or more non-serious adverse event; the outcome of "any adverse event" will be used as a surrogate if necessary.

References

1. Gates M, Pillay J, Thériault G, Limburg H, Grad R, Klarenbach S, et al. Screening to prevent fragility fractures among adults 40 years and older in primary care: protocol for a systematic review. Syst Rev. 2019 Dec 23;8(1):216.

2. Gates M, Pillay J, Nuspl M, Wingert A, Vandermeer B, Hartling L. Screening for the primary prevention of fragility fractures among adults aged 40 years and older in primary care: systematic reviews of the effects and acceptability of screening and treatment, and the accuracy of risk prediction tools. Syst Rev. 2023;12(1):51.

Appendix 9, as supplied by the authors. Appendix to: Thériault G, Limburg H, Klarenbach S, et al. Recommendations on screening for primary prevention of fragility fractures. *CMAJ* 2023. doi: 10.1503/cmaj.221219. Copyright © 2023 The Author(s) or their employer(s). To receive this resource in an accessible format, please contact us at cmajgroup@cmaj.ca.
